# Thermally Induced Intramolecular Diels–Alder Reaction of Furan-Tethered Methylenecyclopropanes

**DOI:** 10.3390/molecules30204105

**Published:** 2025-10-16

**Authors:** Qi-Yun Huang, Xin-Tao Gu, Yin Wei, Min Shi

**Affiliations:** 1 Key Laboratory for Advanced Materials, Institute of Fine Chemicals, School of Chemistry & Molecular Engineering, East China University of Science and Technology, 130 Meilong Road, Shanghai 200237, China; y30230292@mail.ecust.edu.cn; 2 State Key Laboratory of Organometallic Chemistry, Center for Excellence in Molecular Synthesis, Shanghai Institute of Organic Chemistry, University of Chinese Academy of Sciences, Chinese Academy of Sciences, 345 Lingling Road, Shanghai 200032, China; xtgu@sioc.ac.cn

**Keywords:** Diels–Alder reaction, methylenecyclopropanes, cycloaddition, DFT calculation

## Abstract

The substantial ring strain and activated double bonds render methylenecyclopropanes (MCPs) potential substrates for Diels–Alder (DA) reactions. In this work, we present a thermally induced intramolecular Diels–Alder (IMDA) reaction utilizing furan-tethered MCPs. The reactions were carried out smoothly with respect to a wide variety of substrates with good functional group compatibility, affording the desired products in moderate to excellent yields. The synthetic utility of these products was successfully demonstrated. Mechanistic studies involving radical scavenger control experiments and density functional theory (DFT) calculations revealed a concerted mechanism involving an asynchronous one-step pathway.

## 1. Introduction

**Scheme 1:**
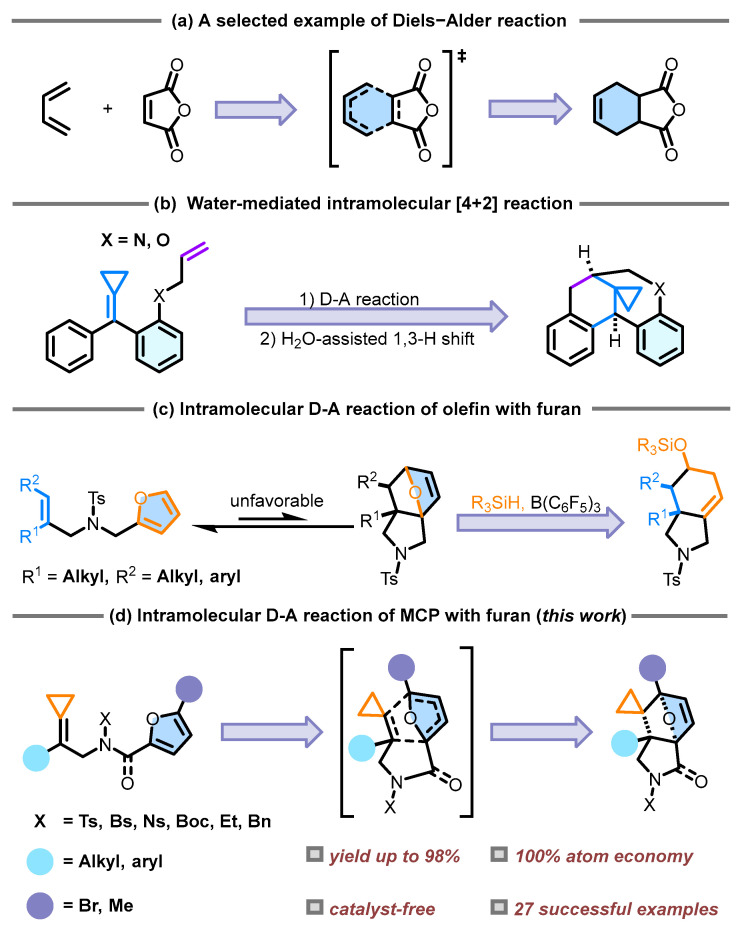
Backgrounds and this work.

The Diels–Alder (DA) reaction between dienes and alkene derivatives [1], recognized as the most iconic [4+2] cycloaddition in organic chemistry, was first reported in 1928. Mechanistic studies indicate that most DA reactions follow a polar mechanism, with nucleophilic or electrophilic interactions present in the transition state structure (TS) [2]. This reaction between a diene and a dienophile may undergo through a concerted mechanism involving a synchronous or an asynchronous TS, yielding a six-membered cyclohexene derivative, such as 1,3-butadiene and maleic anhydride (Scheme 1a). Characterized by its exceptional atom economy [3,4,5] and stereospecificity [6,7,8], this methodology remains the predominant approach for constructing cyclohexene frameworks. The DA reaction serves as a pivotal synthetic strategy for the precise construction of certain structurally complex natural products [9,10,11,12,13,14]. For example, it demonstrates exceptional efficacy [15,16,17,18] in assembling substituted quinolines and their tetrahydro derivatives and pyra-no[3,2-c]quinolines as well as indeno[2,1-c] quinolones.

Methylenecyclopropanes (MCPs) represent a unique class of cyclopropane derivatives characterized by exocyclic double bonds. Their substantial ring strain (40 kcal/mol) [19,20] and reactive exocyclic double bonds establish these compounds as privileged building blocks for accessing cyclopropane-containing molecular architectures [21,22] with conventionally inaccessible complexity. Ring strain has driven the development of diverse cycloaddition methodologies with MCPs, spanning thermally activated systems [23,24], Lewis or Brønsted acid catalysis [25,26], transition-metal-mediated processes [27,28,29], and radical-triggered pathways [30,31], even through electrocatalytic processes [32]. The pronounced reactivity of the exocyclic double bond also renders MCPs privileged substrates in DA reactions. The release of DA reactions employing MCPs has been documented within cyclopropane chemistry. In 2021, our research group published a paper [33] on allylamino- or allyloxy-tethered alkylidenecyclopropanes undergoing an intramolecular DA reaction (Scheme 1b). In this reaction, the substrate undergoes a DA reaction followed by a water-assisted 1,3-H shift leading to aromatization, yielding the final product; the proposed mechanism for this reaction has been confirmed through DFT calculations. Although significant progress has been made in the DA reaction of furan [34], most studies have focused on its reactions with strongly electrophilic alkenes, while DA reactions involving nucleophilic alkenes remain limited. The DA reaction between furan and nucleophilic alkenes faces two main challenges. Furan is highly nucleophilic and readily undergoes DA reactions with electrophilic alkenes, making it difficult to react with nucleophilic alkenes [35] and the aromaticity of furan reduces its reactivity toward electrophilic alkenes in DA reactions [36]. These two unfavorable factors can be overcome by the Intramolecular Diels–Alder (IMDA) reaction, which reduces the unfavorable activation entropy of the intermolecular process, thereby overcoming the high activation enthalpy resulting from the low polarity of the reaction and the aromaticity of furan [37]. In 2019, the Wang’s group reported a catalytic cascade reaction of *N*-allyl-*N*-furfurylamides [38]. This transformation proceeds through sequential steps: (1) initial DA cycloaddition; (2) B(C_6_F_5_)_3_-catalyzed ether bond cleavage mediated by R_3_Si-H species (Scheme 1c). Regrettably, the standalone DA reaction is not feasible and the addition of B(C_6_F_5_)_3_ and R_3_Si-H species is required to induce ether bond cleavage, thereby driving the DA reaction forward. Building upon past research, we hypothesize that strategic modification of the alkene substrate through terminal cyclopropane installation probably facilitates the DA reaction process, yielding the desired [4+2] adduct.

In this study, we developed a thermally induced IMDA reaction of furan-tethered MCPs (Scheme 1d). The transformation from a planar trigonal configuration to a tetrahedral one results in the release of ring strain in the MCP moiety, thereby reducing the strain energy and promoting the IMDA reaction. The spirocyclopropane-oxygen-bridged hexahydroisoindole is obtained in excellent yield (up to 98%) with complete atom economy (100%) under catalyst-free reaction conditions.

## 2. Results and Discussion

**Scheme 2:**
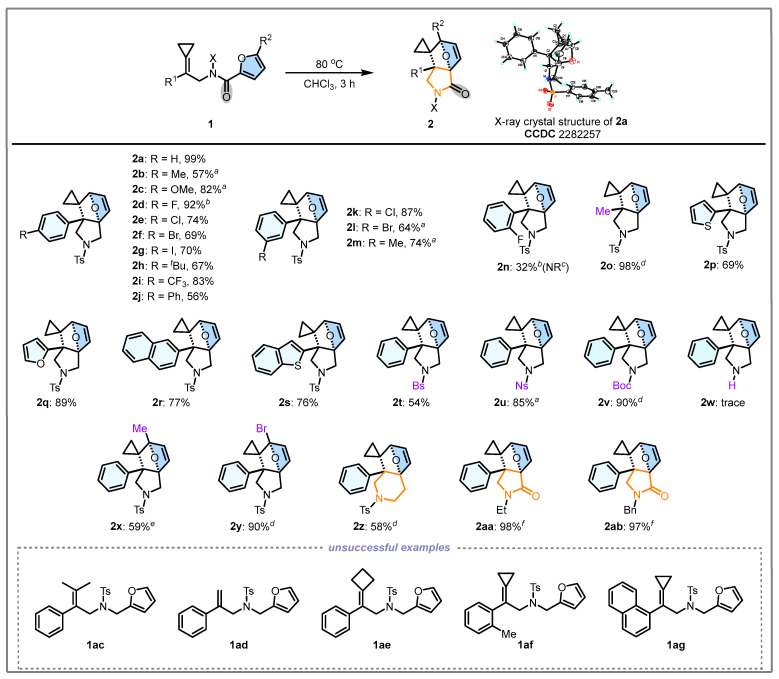
Scope of substrates for thermally induced intramolecular DA reaction of **1**. Reaction conditions: **1** (0.1 mmol), CHCl_3_ (2.0 mL), 80 °C for 3.0 h. *^a^* Reaction time was prolonged to 10 h. *^b^* Reaction conditions: **1** (0.1 mmol), PhMe (2.0 mL), 120 °C for 12 h. *^c^* Reaction time was prolonged to 24 h. *^d^* Reaction time was prolonged to 56 h. *^e^* Reaction time was prolonged to 96 h. *^f^* Reaction time was prolonged to 72 h.

The preliminary investigation (Table 1, Entry 1) demonstrated that substrate **1a** in CHCl_3_ upon heating at 40 °C for 12 h generated the target product **2a** in 38% yield. Its structure was unambiguously identified by X-ray diffraction. The ORTEP drawing is shown in Scheme 2 and the CIF data are summarized in the Supporting Information. Subsequent optimization of reaction time (Table 1, Entries 1–4) revealed that the prolonged reaction time significantly enhanced reaction efficiency, achieving >95% NMR yield after 96 h. Then, thermal activation strategies (Table 1, Entries 5–8) enabled substantial acceleration, where systematic parameter screening (CHCl_3_, 80 °C, 3.0 h) afforded **2a** in 98% isolated yield (Table 1, Entry 8). Notably, other solvents (Table 1, Entries 9–11) led to slightly diminished yields, confirming CHCl_3_ is the most optimal solvent. Based on the above experiments, the optimal reaction conditions have been determined as follows: **1a** (0.1 mmol), CHCl_3_ (2.0 mL) as the solvent, and reaction at 80 °C for 3.0 h, affording the product **2a** in an isolated yield of 98% (Table 1, Entry 8).

Under the optimized conditions, the substrate scope of the intramolecular DA reaction was investigated (Scheme 2). For aromatic positional effects (R), *para*-substitution (**2b**–**2n**) with diverse groups such as Me, OMe, F, Cl, Br, I, *^t^* Bu, CF_3_, and Ph afforded DA products in 56–92% yields, demonstrating broad electronic tolerance. *Meta*-substituents exhibited moderate efficiency (Cl: **2k**, 87%, Br: **2l**, 64%, Me: **2m**, 74%), while *ortho*-position modification caused severe steric inhibition, with only F-substitution enabling partial reactivity (32%) and Me groups (**1af**) completely suppressing the reaction (see page S16 in the SI for the detailed substrate scope). Notably, using substrate **1o** in which the Ph group was replaced with a Me group, the reaction was achieved to give **2o** in 98% yield; however, the reaction time was significantly prolonged. The reactions also proceeded smoothly when Ph was replaced with heteroaromatic ring or fused aromatic rings: 2-thienyl (**2p**, 69% yield), 2-furyl (**2q**, 95% yield), 2-naphthyl (**2r**, 77% yield), and 2-benzothienyl (**2s**, 76% yield). Notably, the 1-naphthyl-substituted substrate (**1ag**) failed to react, probably due to steric hindrance. Subsequently, we explored the scope of X substituents including Bs (**1t**), Ns (**1u**), and Boc (**1v**), and the corresponding DA reactions yielded the corresponding products **2t**, **2u**, **2v** in 54%, 85%, and 90% yields, respectively. Unfortunately, the reaction barely proceeded when X = H (**2w**, trace). Furthermore, the R^2^ group on the furan moiety was also explored. When R^2^ = Me (**1x**) and R^2^ = Br (**1y**), the reaction yielded the desired products **2x** and **2y** in 59% and 90% yields, respectively. The reaction can proceed smoothly even when substrate with a methylene-elongated carbon chain, affording **2z** in 58% yield. Finally, we investigated the reactivity of amide-linked substrates, with X = Et (**1aa**) and X = Bn (**1ab**), the corresponding products **2aa** and **2ab** were also accessed in 98% and 97% yields, respectively. Notably, the corresponding reaction time of **2aa** and **2ab** extended to 72 h, which may be attributed to the electron-donating effect of the amide group reducing the nucleophilicity of the furan ring, consequently leading to diminished reactivity. Furthermore, substrates **1ac**–**1ag** showed no reaction at either 80 °C or 120 °C, and were found to gradually decompose at the higher temperature.

**Scheme 3:**
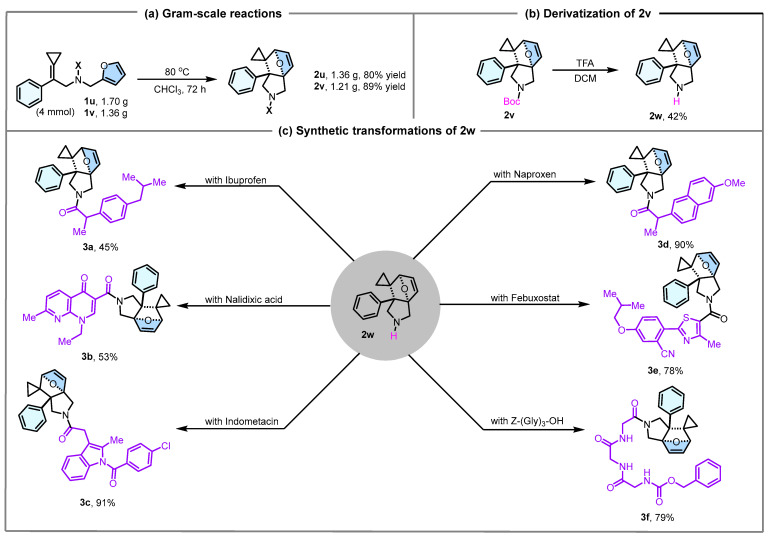
Large-scale synthesis and synthetic transformations.

Having established the substrate scope, we subsequently performed gram-scale reactions of **1u** and **1v**, which consistently afforded products **2u** and **2v** in 80% and 89% isolated yields, respectively (Scheme 3a), thereby confirming the scalability of this strategy. Subsequently, investigation on the transformation of **2v** demonstrated that *N*-Boc deprotection under TFA-mediated conditions using DCM as a solvent afforded product **2w** in 42% isolated yield (Scheme 3b). In contrast, the attempt to access **2w** via thermally-induced DA reaction failed. Noteworthy, compound **2w** demonstrated remarkable adaptability in HATU- and EDCI-activated condensations, effectively coupling with both therapeutic agents (**3a**, with ibuprofen, in 45% yield; **3b**, with nalidixic acid, in 53% yield; **3c**, with indometacin, in 91% yield; **3d**, with naproxen in 90% yield; **3e**, with febuxostat, in 78% yield) and the peptide (**3f**, with Z-Gly-Gly-Gly-OH, in 79% yield) (Scheme 3c).

**Scheme 4:**
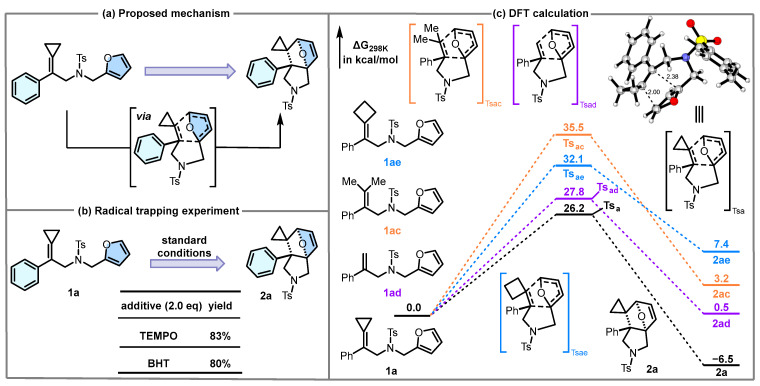
Mechanistic studies (the geometry of **Ts_a_** visualized with CYLview 2.0 [39]).

Here, we focus on the mechanistic studies. Based on the typically thermal DA reaction, we propose that the reaction proceeds via a concerted six-membered ring TS (Scheme 4a). To gain more mechanistic insights into this IMDA reaction, the control experiments in the presence of 2,2,6,6-tetramethylpiperidinooxy (TEMPO) and butylated hydroxytoluene (BHT) were conducted in Scheme 4b. The radical scavengers (TEMPO/BHT) have negligible suppression of product formation, effectively ruling out significant radical pathway involvement. To further elucidate the mechanism of this IMDA reaction, we conducted DFT calculations on the proposed mechanism (Scheme 4c). Theoretical calculations indicate that the IMDA of **1a** follows a concerted mechanism through **TSa** with an energy barrier of 26.1 kcal/mol, which can be overwhelmed under the standard reaction conditions. An asynchronous TS is indicated by the different distances of the two forming C-C bonds (Δd = 0.38) in the geometry of **TSa**. The high activation barrier 26.2 kcal/mol stems from the low polarity of the IMDA reaction and the aromaticity of furan, which precisely indicates that the reaction should proceed through an intramolecular pathway. The formation of **2a** is an exothermic process (ΔG_rxn_ = −6.5 kcal/mol). The calculated results demonstrate the non-reactive substrates (e.g., compounds **1ac**, **1ae**) exhibit significantly high energy barriers (ΔG^≠^ = 35.5 kcal/mol, 32.1 kcal/mol), indicating the reaction is difficult to occur under the standard reaction conditions. The calculation results are in line with experimental results that showed no reactions occurred using **1ac** and **1ae** as substrates. Although the activation barrier of **1ad** (ΔG^≠^ = 27.8 kcal/mol) is extremely close to that of **1a** (ΔG^≠^ = 26.2 kcal/mol), inertness of **1ad** for this reaction may stem from the energy of its product **2ad** being slightly higher than that of **1ad** by 0.5 kcal/mol, which drives cycloreversion to **1ad** (ΔG^≠^*_Re_* = 27.3 kcal/mol) to take place more easily. Therefore, the corresponding product **2ad** was not obtained in the experiment. Theoretical calculations reveal that the three-membered ring in MCPs serves as a pivotal structural unit driving the intramolecular DA reaction process. We postulate that this three-membered ring system facilitates the reaction by electronically activating the olefin component, thereby lowering the energy of TS and enhancing cyclization propensity. The possible stepwise ionic mechanism for this reaction is also investigated theoretically. The calculation results indicate the related zwitterionic intermediates are unstable (for details, see the page S5 of Supporting Information); thus, the possible stepwise ionic mechanism for DA reaction is excluded.

## 3. Materials and Methods

### 3.1. General Procedure for the Preparation of ***2***

To a 10.0 mL sealed tube were added substrate **1** (0.10 mmol, 1.0 eq), and commercially available CHCl_3_ (2.0 mL); then, the resulting mixture was stirred at 80 °C for 3.0 h. Then, the solvent was removed under vacuum and the residue was purified via silica gel column chromatography (PE:EA = 4:1) to give the desired products **2** in 32–99% yields. The common solvents (PE, EA, etc.) were obtained from Titan Company in Shanghai, China. All super-dry solvents were obtained from J&K Scientific in China.

### 3.2. General Procedure for Scale-Up Synthesis and Transformation

#### 3.2.1. Scale-Up Synthesis of **2u** and **2v**

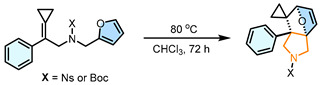



To a 100 mL flask were added substrate **1v** (4 mmol, 1.0 eq), and CHCl_3_ (40.0 mL), and the resulting mixture was stirred at 80 °C for 72 h. Then, the solvent was removed under vacuum and the residue was purified via silica gel column chromatography (PE:EA = 4:1) to give the desired products in 80% yield for **2u** and 89% yield for **2v**.

#### 3.2.2. Transformation of the Product

##### Deprotection of the Boc Group in Compound **2v**

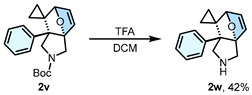



The reaction was conducted in a 15 mL round-bottom flask charged with compound **2v** (3.0 mmol). Then, 7.5 mL of CF_3_COOH was added. The reaction mixture was stirred for 4.0 h, and quenched with water, and neutralized with Et_3_N. The reaction solution was extracted three times with EA. The combined organic layers were dried over anhydrous Na_2_SO_4_, and the solvent was removed under reduced pressure, and purified via silica gel column chromatography (DCM:MeOH = 20:1). Compound **2w** was obtained in 42% yield. Reagents (CF_3_COOH and Et_3_N, etc.) were obtained from Leyan Company in Shanghai, China.

##### Condensation Reaction of Compound **2w**

***Condensation with ibuprofen***

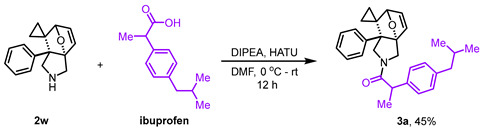

The reaction was conducted in a 25 mL round-bottom flask charged with ibuprofen (1.2 mmol, 1.2 eq) and HATU. Dry DMF was added to dissolve the mixture, followed by the addition of DIPEA. After pre-stirring for 30 min at 0 °C, **2w** (0.1 mmol, 1.0 eq) of 1.0 mL of dry DMF solution was introduced, and the reaction mixture was allowed to react for 12 h at room temperature. After completion of the reaction, the reaction was quenched with water and extracted three times with EA. The combined organic phases were washed three times with water, dried over anhydrous Na_2_SO_4_, and the solvent was removed under reduced pressure. The residue was purified via silica gel column chromatography (PE:EA = 3:1) to afford the product **3a** in 45% yield.

***Condensation with Nalidixic acid, Indometacin, Naproxen, Febuxostat and Z-Gly-Gly-Gly-OH***

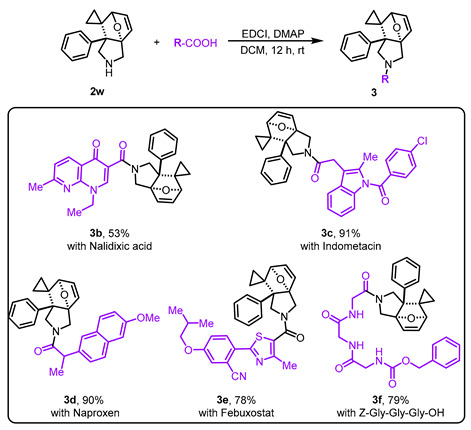

The reaction was conducted in a 25 mL round-bottom flask charged with RCOOH (1.2 mmol, 1.2 eq), EDCI (1.2 mmol, 1.2 eq) and DMAP (0.2 mmol, 0.2 eq). Then, dry THF was added to dissolve the mixture. The reaction mixture was allowed to react for 12 h at room temperature. After completion of the reaction, the reaction was quenched with water and extracted three times with DCM. The combined organic phases were dried over anhydrous Na_2_SO_4_, and the solvent was removed under reduced pressure. The residue was purified via silica gel column chromatography (PE:EA = 3:1) to afford the products **3b**, **3c**, **3d**, **3e** and **3f** in 53–91% yields.

### 3.3. General Procedure for the Preparation of ***1***

#### 3.3.1. General Procedure for the Synthesis of Compounds **1a**–**1u**, **1x**, **1z**, **1af**–**1ai**, and **1al**

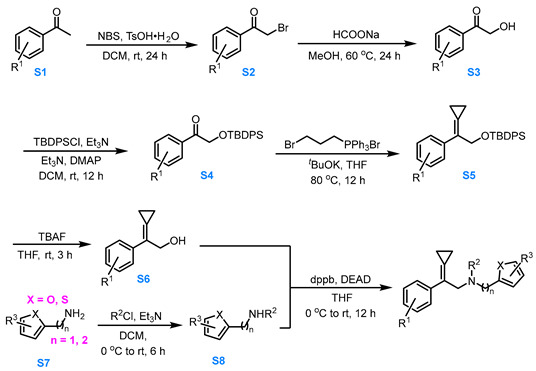

***Compound* S1 *to Compound* S2**

At room temperature, *N*-bromosuccinimide (1.1 eq, 44.0 mmol) was added dropwise to a solution of compound **S1** (1.0 eq, 40.0 mmol) and TsOH·H_2_O (0.1 eq, 4.0 mmol) in DCM (100.0 mL), and the reaction mixture was stirred for 24 h. After completion of the reaction, the reaction was quenched with water and extracted with DCM. The combined organic layers were washed twice with saturated NaCl solution, dried over anhydrous Na_2_SO_4_, and the solvent was removed under vacuum. The residue was purified via silica gel column chromatography (eluent: PE:EA = 10:1) to afford compound **S2** in moderate yield.

***Compound*** **S2 *to Compound* S3**

HCOONa (3.0 eq, 75.0 mmol) was added to the MeOH (80.0 mL) of compound **S2** (1.0 eq, 25.0 mmol) and refluxed at 60 °C for 24 h. After the reaction was completed, the reaction was quenched with water, extracted three times with DCM. The combined organic phases were washed two times with saturated NaCl solution, dried over anhydrous Na_2_SO_4_. The solvent was removed under vacuum and the residue was purified via silica gel column chromatography (eluent: PE:EA = 10:1) to afford compound **S3** in moderate yield.

***Compound* S3 *to Compound* S4**

Tert-Butylchlorodiphenylsilane (TBDPSCl, 1.1 eq, 11.0 mmol) was added to a solution containing compound **S3**, Et_3_N (1.2 eq, 12.0 mmol), and 4-dimethylaminopyridine (DMAP, 0.1 eq, 1.0 mmol) in DCM (16.0 mL). The reaction mixture was stirred for 12 h. After completion, the reaction was quenched with water, extracted three times with PE. The combined organic phases were dried over anhydrous Na_2_SO_4_, concentrated under reduced pressure, and the residue was purified via silica gel column chromatography (eluent: PE:EA = 20:1) and the compound S4 was obtained in about 90% yield.

***Compound*** **S4 *to Compound* S5**

A solution of 3-bromopropyltriphenylphosphonium bromide (1.5 eq, 15.0 mmol) and *^t^*BuOK (3.0 eq, 30.0 mmol) in dry THF (30.0 mL) was stirred at 80 °C under Ar for 0.5 h. Afterward, compound **S4** (1.0 eq, 10.0 mmol) in dry THF (15.0 mL) was added slowly and the reaction solution was stirred at 80 °C for 12 h. After the reaction was completed, the reaction mixture was cooled to room temperature, filtered through Celite, and the filtrate was concentrated under reduced pressure. The resulting residue was purified via silica gel column chromatography (pure PE) to afford compound **S5** in moderate yield.

***Compound*** **S5 *to Compound* S6**

At room temperature, a 1.0 M THF solution of TBAF (1.5 eq, 7.5 mmol, 7.5 mL) was added to a THF solution of compound **S5** (1.0 eq, 5.0 mmol), and the reaction mixture was stirred for 3.0 h. After the resulting solution had been stirred for 3.0 h, the reaction was quenched with water, and the aqueous layer was extracted with MTBE three times. The combined organic phases were dried over anhydrous Na_2_SO_4_, concentrated under reduced pressure, and the residue was purified via silica gel column chromatography (eluent: PE:EA = 4:1) to afford compound **S6** in about 90% yield.

***Compound*** **S7 *to Compound* S8**

Compound **S7** (1.0 eq, 10 mmol) and Et_3_N (1.3 eq, 13 mmol) were dissolved in DCM (40 mL). R^2^Cl (R^2^ = Ts, Bs, Ns, 1.1 eq, 11 mmol) was added portionwise at 0 °C. The reaction mixture was stirred at room temperature for 8.0 h and then concentrated under reduced pressure. The residue was purified via silica gel column chromatography (eluent: PE:Acetone = 4:1) to afford compound **S8** in moderate yield.

***Compound*** **S6 *and* S8 *to* 1a–1u, 1z, 1af–1ai, *and* 1al**

Compound **S6** (1.0 eq, 3.0 mmol), **S8** (1.0 eq, 3.0 mmol) and dppb (1.5 eq, 4.5 mmol) was dissolved in dry THF (15.0 mL). Under Ar protection, the reaction mixture was stirred while DEAD (1.5 eq, 4.5 mmol) was added dropwise to the system at 0 °C. The reaction was allowed to proceed for 12 h at room temperature. After the reaction was completed, the reaction mixture was filtered through Celite and the filtrate was concentrated under reduced pressure at 26 °C. The residue was purified via silica gel flash column chromatography (eluent: PE:MTBE = 8:1) to afford the products **1a**–**1s** in moderate yields.

#### 3.3.2. General Procedure for the Synthesis of Compounds **1w** and **1v**

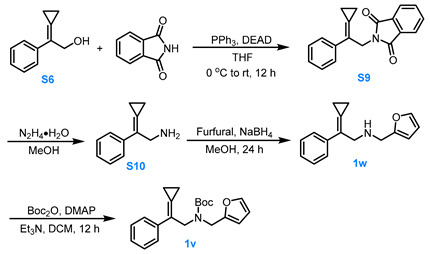

***Compound*** ***S6 to Compound* S9**

Compound **S6** (1.0 eq, 5.0 mmol), phthalimide (1.0 eq, 5.0 mmol) and PPh_3_ (1.5 eq, 7.5 mmol) was dissolved in dry THF (15.0 mL). Under Ar protection, the reaction mixture was stirred while DEAD was added dropwise to the system at 0 °C. The reaction was allowed to proceed for 12 h at room temperature. After the reaction was completed, the reaction mixture was filtered through Celite and the filtrate was concentrated under reduced pressure at 26 °C. The residue was purified via silica gel flash column chromatography (eluent: PE:EA = 10:1) to afford the compound **S9** in about 60% yield.

***Compound*** **S9 *to Compound* S10**

N_2_H_4_·H_2_O (10.0 eq, 30.0 mmol, 1.8 mL, 85%) was added to a methanol suspension (20 mL) of compound **S9** (1.0 eq, 3.0 mmol). After stirring for 3.0 h, TLC checking indicated complete consumption of the starting materials. A 2.0 M KOH solution (20 mL) was then added, and the reaction mixture was stirred for 0.5 h. The solution was extracted with DCM three times, and the combined organic phases were washed two times with saturated NaCl solution, dried over anhydrous Na_2_SO_4_, and concentrated under reduced pressure to afford compound **S10**.

***Compound*** **S10 *to Compound* 1w**

Furfural (1.1 eq, 2.0 mmol) was added to a methanol solution (10 mL) of compound **S10** (1.0 eq, 2.0 mmol), and the reaction mixture was stirred for 24 h. NaBH_4_ (2.0 eq, 4.0 mmol) was then added at 0 °C, and the reaction mixture was stirred under open conditions for 3.0 h. After completion, water was slowly added to quench the reaction. The reaction mixture was extracted with EA three times, and the combined organic phases were washed two with saturated NaCl solution, dried over anhydrous Na_2_SO_4_. The organic solvents were removed under reduced pressure, and the crude product was purified via silica gel flash column chromatography (eluent: PE:EA = 4:1) to afford the compound **1w** in about 75% yield.

***Compound*** **1w *to Compound* 1v**

Et_3_N (2.5 eq, 3.8 mmol) was added to a DCM solution (4.0 mL) containing compound **1w** (1.0 eq, 1.5 mmol and DMAP (0.2 eq, 0.3 mmol), followed by the addition of Boc_2_O (1.3 eq, 2.0 mol). The reaction mixture was stirred for 12 h, and then concentrated under reduced pressure at 26 °C, and the residue was purified by a silica gel column chromatography (eluent: PE:MTBE = 8:1) to afford compound **1v** in 61% yield.

#### 3.3.3. General Procedure for the Synthesis of Compound **1y**

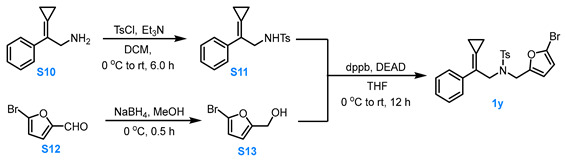

***Compound*** **S10 *to Compound* S11**

Compound **S10** (1.0 eq, 2.0 mmol) and Et3N (1.3 eq, 2.6 mmol) were dissolved in DCM (5.0 mL). TsCl was added portionwise at 0 °C. The reaction mixture was stirred at room temperature for 8.0 h, then concentrated under reduced pressure. The residue was purified via silica gel column chromatography (eluent: PE:Acetone = 4:1) to afford compound **S11** in 73% yield.

***Compound*** **S12 *to Compound* S13**

NaBH_4_ (0.55 eq, 2.75 mmol) was added to a methanol solution (8.0 mL) of compound **S12** (1.0 eq, 5.0 mmol) at 0 °C, and the reaction mixture was stirred for 0.5 h. The reaction was then quenched with saturated NH_4_Cl solution and extracted with diethyl ether three times. The combined organic phases were washed two times with saturated NaCl solution, dried over anhydrous Na_2_SO_4_, and 10 mL of anhydrous THF was added. The diethyl ether was removed under reduced pressure at 0 °C to obtain a THF solution (10 mL) of compound **S13** (0.5 mol/L).

***Compound*** **S11 *and* S13 *to Compound* 1y**

Compound **S11** (1.0 eq, 1.5 mmol), **S13** (1.0 eq, 1.5 mmol) and dppb (1.5 eq, 2.3 mmol) was dissolved in dry THF (7.5 mL). Under Ar protection, the reaction mixture was stirred while DEAD (1.5 eq, 2.3 mmol) was added dropwise to the system at 0 °C. The reaction was allowed to proceed for 12 h at room temperature. After the reaction was completed, the reaction mixture was filtered through a Celite and the filtrate was concentrated under reduced pressure at 26 °C. The residue was purified via silica gel flash column chromatography (eluent: PE:MTBE = 8:1) to afford the product **1y** in 20% yield.

#### 3.3.4. General Procedure for the Synthesis of Compounds **1aa**–**1ab**, and **1ak**

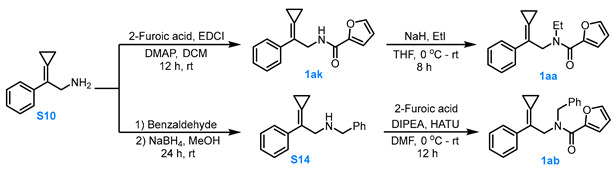

***Compound*** **S10 *to Compound* 1ak**

Under Ar atmosphere, a dry DCM (10 mL) solution containing compound **S10** (1.0 eq, 2.0 mmol), 2-furoic acid (1.1 eq, 2.2 mmol), EDCI (1.1 eq, 2.2 mmol) and DMAP (0.2 eq, 0.4 mmol) was stirred for 8.0 h. After completion of the reaction, the solvent was removed under reduced pressure at 26 °C. The residue was purified via silica gel column chromatography (eluent: PE:MTBE = 2:1) to afford compound **1ak** in 60% yield.

***Compound*** **1ak *to Compound* 1aa**

Under Ar atmosphere, EtI (2.0 eq, 2.0 mmol) was added to a dry THF solution containing NaH (1.2 eq, 1.2 mmol) and compound 1ak (1.0 eq, 1.0 mmol) that had been pre-stirred for 1.0 h, and the reaction was allowed to proceed for 8.0 h. After completion of the reaction, the reaction solution was extracted three times with EA, and the combined organic phases were concentrated under reduced pressure. Then, the solvent was removed under reduced pressure at 26 °C. The residue was purified via silica gel column chromatography (eluent: PE:MTBE = 4:1) to afford compound **1aa** in 77% yield.

***Compound*** **S10 *to Compound* S14**

Benzaldehyde (1.05 eq, 1.05 mmol) was added to a methanol solution (10 mL) of compound **S10** (1.0 eq, 1.0 mmol), and the reaction mixture was stirred for 24 h. NaBH_4_ (2.0 eq, 2.0 mmol) was then added at 0 °C, and the reaction mixture was stirred under open conditions for 3.0 h. After completion, water was slowly added to quench the reaction. The mixture was extracted with EA three times, and the combined organic phases were washed two times with saturated NaCl solution, dried over anhydrous Na_2_SO_4_. The organic solvents were removed under reduced pressure to afford crude compound **S14**. Compound **14** was carried forward to the next step without purification.

***Compound*** **S14 *to Compound* 1ab**

Under Ar atmosphere, DIPEA (2.0 eq, 2.0 mmol) was added to a solution of 2-furoic acid (1.2 eq, 1.2 mmol) and HATU (1.2 eq, 1.2 mmol) in anhydrous DMF (10 mL) at 0 °C. After stirring for 0.5 h, a solution of **S14** (1.0 eq, 1.0 mmol) in anhydrous DMF (3.0 mL) was introduced, and the reaction was allowed to proceed for 12 h. Upon completion, the reaction was quenched with water, extracted three times with MTBE. The combined organic phases were washed three times with water, concentrated under reduced pressure at 26 °C, and the residue was purified via silica gel column chromatography (eluent: PE:MTBE = 4:1) to afford Compound **1ab** in 50% yield.

#### 3.3.5. General Procedure for the Synthesis of Compound **1ac**

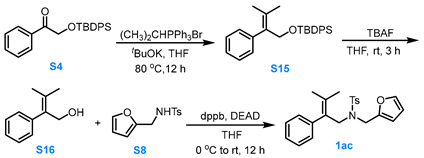

***Compound*** **S4 *to Compound* S15**

A solution of (CH_3_)_2_CHPPh_3_Br (1.5 eq, 15.0 mmol) and *^t^*BuOK (3.0 eq, 30.0 mmol) in dry THF (30.0 mL) was stirred at 80 °C under Ar for 0.5 h. Afterward compound **S4** (1.0 eq, 10.0 mmol) in dry THF (15.0 mL) was added slowly and the reaction solution was stirred at 80 °C for 12 h. After the reaction was completed, the mixture was cooled to room temperature, filtered through Celite, and the filtrate was concentrated under reduced pressure. The resulting residue was purified via silica gel column chromatography (pure PE) to afford compound **S15** in moderate yield.

***Compound*** **S15 *to Compound* S16**

At room temperature, a 1.0 M THF solution of TBAF (1.5 eq, 7.5 mmol, 7.5 mL) was added to a THF solution of compound **S15** (1.0 eq, 5.0 mmol), and the mixture was stirred for 3.0 h. The resulting solution was stirred for 3.0 h. The reaction was quenched with water, and the aqueous layer was extracted with MTBE three times. The combined organic phases were dried over anhydrous Na_2_SO_4_, concentrated under reduced pressure, and the residue was purified via silica gel column chromatography (eluent: PE:EA = 4:1) to afford compound **S16** in about 90% yield.

***Compound*** **S16 *to Compound* 1ac**

Compound **S16** (1.0 eq, 3.0 mmol), **S8** (1.0 eq, 3.0 mmol) and dppb (1.5 eq, 4.5 mmol) was dissolved in dry THF (15.0 mL). Under Ar protection, the reaction mixture was stirred while DEAD (1.5 eq, 4.5 mmol) was added dropwise to the system at 0 °C. The reaction was allowed to proceed for 12 h at room temperature. After the reaction was completed, the reaction mixture was filtered through Celite and the filtrate was concentrated under reduced pressure at 26 °C. The residue was purified via silica gel flash column chromatography (eluent: PE:MTBE = 8:1) to afford the product **1ac** in moderate yield.

#### 3.3.6. General Procedure for the Synthesis of Compound **1ad**

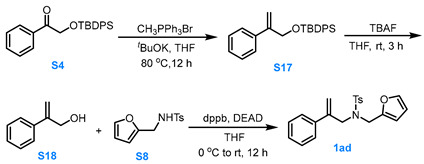

***Compound*** **S4 *to Compound* S17**

A solution of CH_3_PPh_3_Br (1.5 eq, 15.0 mmol) and *^t^*BuOK (3.0 eq, 30.0 mmol) in dry THF (30.0 mL) was stirred at 80 °C under Ar for 0.5 h. Afterward compound **S4** (1.0 eq, 10.0 mmol) in dry THF (15.0 mL) was added slowly and the reaction solution was stirred at 80 °C for 12 h. After the reaction was completed, the reaction mixture was cooled to room temperature, filtered through Celite, and the filtrate was concentrated under reduced pressure. The resulting residue was purified via silica gel column chromatography (pure PE) to afford compound **S17** in moderate yield.

***Compound*** **S17 *to Compound* S18**

At room temperature, a 1.0 M THF solution of TBAF (1.5 eq, 7.5 mmol, 7.5 mL) was added to a THF solution of compound **S15** (1.0 eq, 5.0 mmol), and the reaction mixture was stirred for 3.0 h. The resulting solution was stirred for 3.0 h. The reaction was quenched with water, and the aqueous layer was extracted with MTBE three times. The combined organic phases were dried over anhydrous Na_2_SO_4_, concentrated under reduced pressure, and the residue was purified via silica gel column chromatography (eluent: PE:EA = 4:1) to afford compound **S18** in about 90% yield.

***Compound*** **S18 *to Compound* 1ad**

Compound **S16** (1.0 eq, 3.0 mmol), **S8** (1.0 eq, 3.0 mmol) and dppb (1.5 eq, 4.5 mmol) was dissolved in dry THF (15.0 mL). Under Ar protection, the reaction mixture was stirred while DEAD (1.5 eq, 4.5 mmol) was added dropwise to the system at 0 °C. The reaction was allowed to proceed for 12 h at room temperature. After the reaction was completed, the reaction mixture was filtered through Celite and the filtrate was concentrated under reduced pressure at 26 °C. The residue was purified via silica gel flash column chromatography (eluent: PE:MTBE = 8:1) to afford the product **1ad** in moderate yield.

#### 3.3.7. General Procedure for the Synthesis of Compound **1ae**

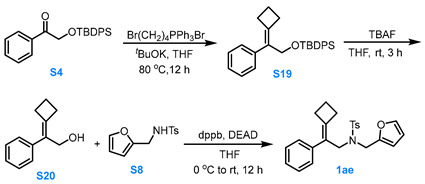

***Compound*** **S4 *to Compound* S19**

A solution of Br(CH_2_)_4_PPh_3_Br (1.5 eq, 15.0 mmol) and *^t^*BuOK (3.0 eq, 30.0 mmol) in dry THF (30.0 mL) was stirred at 80 °C under Ar for 0.5 h. Afterward compound **S4** (1.0 eq, 10.0 mmol) in dry THF (15.0 mL) was added slowly and the reaction solution was stirred at 80 °C for 12 h. After the reaction was completed, the reaction mixture was cooled to room temperature, filtered through Celite, and the filtrate was concentrated under reduced pressure. The resulting residue was purified via silica gel column chromatography (pure PE) to afford compound **S19** in moderate yield.

***Compound*** **S19 *to Compound* S20**

At room temperature, a 1.0 M THF solution of TBAF (1.5 eq, 7.5 mmol, 7.5 mL) was added to a THF solution of compound **S19** (1.0 eq, 5.0 mmol), and the reaction mixture was stirred for 3.0 h. After the resulting solution was stirred for 3.0 h, the reaction was quenched with water, and the aqueous layer was extracted with MTBE three times. The combined organic phases were dried over anhydrous Na_2_SO_4_, concentrated under reduced pressure, and the residue was purified via silica gel column chromatography (eluent: PE:EA = 4:1) to afford compound **S20** in about 90% yield.

***Compound*** **S20 *to Compound* 1ad**

Compound **S20** (1.0 eq, 3.0 mmol), **S8** (1.0 eq, 3.0 mmol) and dppb (1.5 eq, 4.5 mmol) was dissolved in dry THF (15.0 mL). Under Ar protection, the reaction mixture was stirred while DEAD (1.5 eq, 4.5 mmol) was added dropwise to the system at 0 °C. The reaction was allowed to proceed for 12 h at room temperature. After the reaction was completed, the mixture was filtered through Celite and the filtrate was concentrated under reduced pressure at 26 °C. The residue was purified via silica gel flash column chromatography (eluent: PE:MTBE = 8:1) to afford the product **1ad** in about 50% yield.

#### 3.3.8. General Procedure for the Synthesis of Compound **1aj**

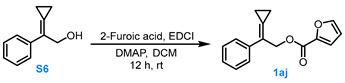

***Compound*** **S6 *to Compound* 1aj**

Under Ar atmosphere, a dry DCM (10 mL) solution containing compound **S6** (1.0 eq, 2.0 mmol), 2-furoic acid (1.1 eq, 2.2 mmol), EDCI (1.1 eq, 2.2 mmol) and DMAP (0.2 eq, 0.4 mmol) was stirred for 8.0 h. After completion of the reaction, the solvent was removed under reduced pressure at 26 °C. The residue was purified via silica gel column chromatography (eluent: PE:MTBE = 2:1) to afford compound **1aj** in 62% yield.

#### 3.3.9. Radical Trapping Experiment

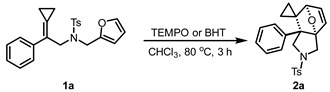



In a 10 mL sealed tube, TEMPO or BHT (0.2 mmol, 2.0 eq) were added to the CHCl_3_ (2.0 mL) solution containing **1a** (0.1 mmol, 1.0 eq), then the resulting mixture was stirred at 80 °C for 3.0 h. Then, the solvent was removed under vacuum and the residue was purified via silica gel column chromatography (PE:EA = 4:1) to give the desired product **2a** in 83% yield when TEMPO was added or in 80% yield when BHT was added. The result showed that 1a remained unchanged and the expected product **2a** was obtained.

### 3.4. Computational Methods

All quantum mechanical calculations were performed with Gaussian 16 [40]. The geometries of all species were optimized at ωB97X-D/6-311G(d,p), SMD (CHCl_3_) level [41,42,43,44]. The subsequent frequency calculations on the stationary points were carried out at the same level of theory to ascertain the nature of the stationary points as minima on the respective potential energy surfaces. All TSs were characterized by only one imaginary frequency pertaining to the desired reaction coordinate. The intrinsic reaction coordinate (IRC) calculations were carried out at the same level of theory to further authenticate the TSs. Thermochemical corrections to 298.15 K have been calculated for all minima from unscaled vibrational frequencies obtained at this same level. The Gibbs free energies (ΔG) in CHCl_3_ discussed in this work were computed at the ωB97X-D/6-311G(d,p) level with SMD solvent model, supplemented by single-point energy corrections at the ωB97X-D/def2-TZVPP level unless otherwise specified.

## 4. Conclusions

In summary, we successfully established a catalyst-free IMDA reaction of furan-tethered MCPs that enabled rapid synthesis of spirocyclopropane-oxygen-bridged hexahydroisoindole derivatives. Mechanistic studies with radical scavengers (TEMPO/BHT) confirmed the absence of radical intermediates, while DFT calculations revealed that this IMDA reaction followed a concerted mechanism involving an asynchronous TS. Furthermore, this cyclization reaction demonstrates high yields and straightforward derivatization capabilities, highlighting its promising potential for applications in the synthesis of complex natural products and pharmaceutical molecules. Studies are currently underway on the synthesis of biologically active substances from these bridged polycyclic compounds as well as the further extension of this cyclization strategy.

## Figures and Tables

**Table 1:** Optimization of the reaction conditions.

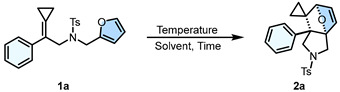				
Entry *^a^*	Solvent	Time	Temperature	Yield (%) *^b^*
1	CHCl_3_	12 h	40 °C	38
2	CHCl_3_	24 h	40 °C	65
3	CHCl_3_	48 h	40 °C	79
4	CHCl_3_	96 h	40 °C	>95
5	CHCl_3_	12 h	40 °C	88
6	CHCl_3_	16 h	60 °C	>95
7	CHCl_3_	12 h	80 °C	>95
8	CHCl_3_	3 h	80 °C	**>95/98 *^c^***
9	PhMe	3 h	80 °C	87
10	THF	3 h	80 °C	92
11	MeCN	3 h	80 °C	94

*^a^* Reaction conditions: **1a** (0.1 mmol), solvent (2.0 mL). *^b^* NMR yield using dimethyl terephthalate (DMT) as an internal standard. *^c^* Isolated yield. The bolded values represent the yields obtained under the optimal conditions.

## Data Availability

Data are contained within the article and Supplementary Materials.

## Supplementary Materials

The following supporting information can be downloaded at: https://www.mdpi.com/article/10.3390/molecules30204105/s1.
