# Supplementary material for: Thermally Induced Intramolecular Diels–Alder Reaction of Furan-Tethered Methylenecyclopropanes
Source: Molecules. 2025 Oct 16;30(20):4105. doi: 10.3390/molecules30204105 (PMC12566586; doi:10.3390/molecules30204105)
Supplement: Supplementary file 1 [file molecules-30-04105-s001.zip › molecules-3871130-supplementary.pdf]

Supporting Information

Thermally-Induced Intramolecular Diels-Alder Reaction of Furan-Tethered Methylenecyclopropanes

Qi-Yun Huang [1], Xin-Tao Gu [2], Yin Wei [2,*] and Min Shi [1,2,*]

1 Key Laboratory for Advanced Materials, Institute of Fine Chemicals, School of Chemistry & Molecular Engineering, East China University of Science and Technology, 130 Meilong Road, Shanghai 200237, China

2 State Key Laboratory of Organometallic Chemistry, Center for Excellence in Molecular Synthesis, Shanghai Institute of Organic Chemistry, University of Chinese Academy of Sciences, Chinese Academy of Sciences, 345 Lingling Road, Shanghai 200032, China

* Correspondence: weiyin@sioc.ac.cn (Y.W.); mshi@mail.sioc.ac.cn (M.S.)

Table of Contents

(1) General Information. S2

(2) Optimization of Reaction Conditions and Scope of Substrates. S3

(3) DFT calculation S5

(4) Spectroscopic Data of Substrates and Products. S6

(5) X-ray Crystal Data of 2a. S156

(6) Computational details S157

(7) References. S181

(1) General Information.

Melting points were determined on a digital melting point apparatus, and temperatures were uncorrected. NMR spectra were recorded with Bruker or Agilent equipment at 400 MHz (^{1}H NMR), 100 MHz (^{13}C NMR) and 376 MHz (^{19}F NMR) in $CDCl_3$, respectively. Data are presented as follows: chemical shift (ppm), multiplicity (s = singlet, d = doublet, t = triplet, q = quartet, sept = septet, m = multiplet), coupling constants in Hertz (Hz), and integration. Infrared spectra were recorded on a Perkin-Elmer PE-983 spectrometer with absorption in cm^{-1}. Mass and high-resolution mass spectra (HRMS) spectra were recorded by ESI method. The employed solvents were dry up by standard methods when necessary. Commercially obtained reagents were used without further purification. For thin-layer chromatography (TLC), silica gel plates (Huanghai GF254) were used. Flash column chromatography was carried out using 300-400 mesh silica gel at increased pressure.

(2) Optimization of Reaction Conditions and Scope of Substrates.

Table S1. Optimization of Reaction Conditions.

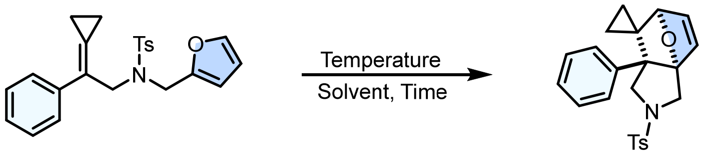

1a **2a**

Entry	Solvent	Time	Temperature	Yield (%)[b]
1	$CHCl_3$	12 h	40 °C	38
2	$CHCl_3$	24 h	40 °C	65
3	$CHCl_3$	48 h	40 °C	79
4	$CHCl_3$	96 h	40 °C	>95
5	$CHCl_3$	12 h	60 °C	88
6	$CHCl_3$	16 h	60 °C	>95
7	$CHCl_3$	12 h	80 °C	>95
8	$CHCl_3$	3 h	80 °C	>95/98[c]
9	PhMe	3 h	80 °C	87
10	THF	3 h	80 °C	92
11	MeCN	3 h	80 °C	94
12	THF	3 h	80 °C	86
13	DCE	3 h	80 °C	84

[a]Reaction conditions: **1a** (0.1 mmol), solvent (2.0 mL). [b]NMR yield using dimethyl terephthalate (DMT) as an internal standard. [c]Isolated yield.

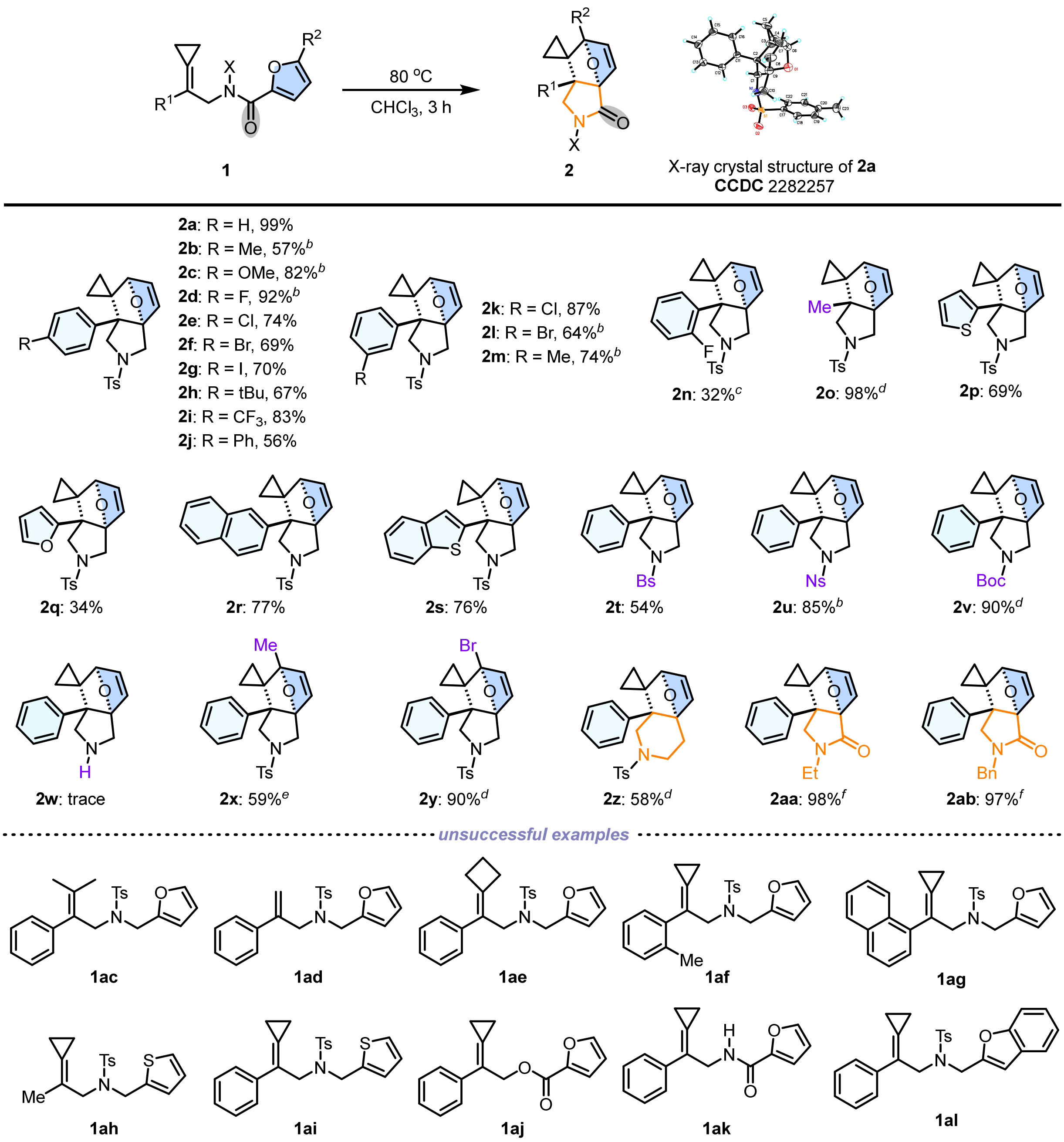

[a]Reaction conditions: **1** (0.1 mmol), $CHCl_3$ (2.0 mL), 80 °C for 3 h. [b]Reaction time was prolonged to 10 h. [c]Reaction conditions: **1** (0.1 mmol), PhMe (2.0 mL), 120 °C for 12 h. [d]Reaction time was prolonged to 96 h. [e]Reaction time was prolonged to 56 h. [f]Reaction time was prolonged to 72 h.

Scheme 2. Scope of Substrates

(3) DFT calculation

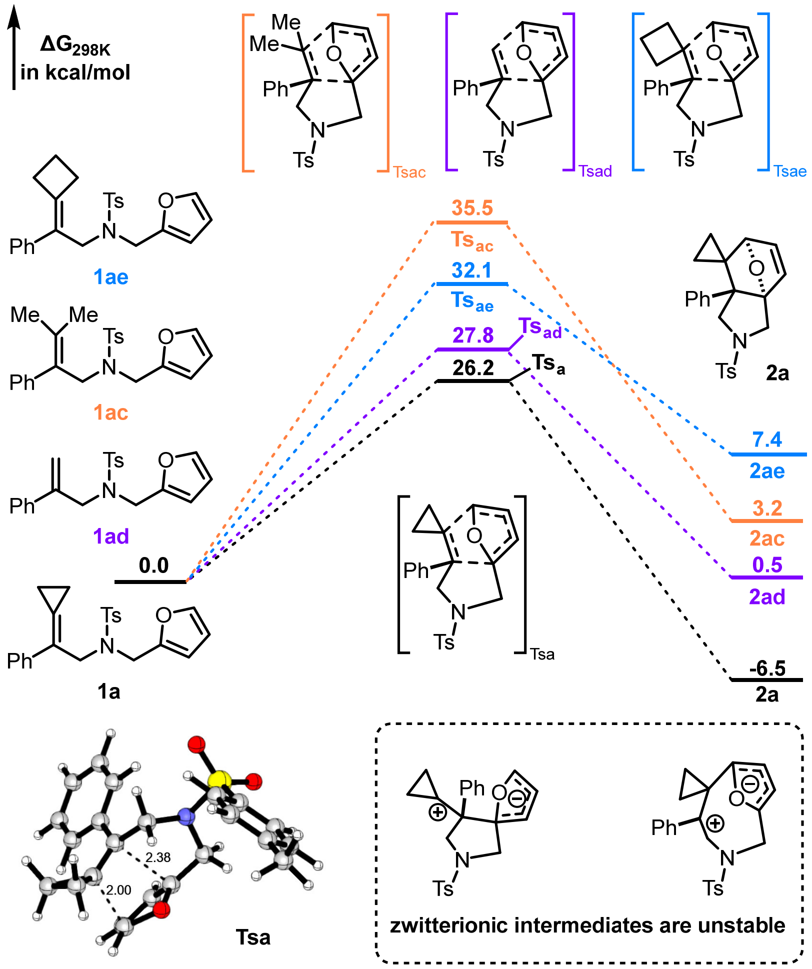

(4) Spectroscopic Data of Substrates and Products.

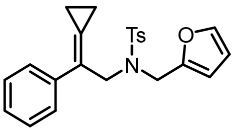

Compound 1a: Yield: 649 mg, 55%; A white solid; M.p.: 85 - 87 ºC; ^{1}H NMR (400 MHz, $CDCl_3$) δ 7.63 – 7.55 (m, 4H), 7.35 – 7.27 (m, 2H), 7.26 – 7.17 (m, 3H), 7.10 (d, *J* = 1.8 Hz, 1H), 6.15 (dd, *J* = 3.2, 1.8 Hz, 1H), 5.91 (d, *J* = 3.2 Hz, 1H), 4.44 (s, 2H), 4.25 (s, 2H), 2.40 (s, 3H), 1.42 – 1.34 (m, 2H), 1.14 – 1.03 (m, 2H); ^{13}C NMR (100 MHz, $CDCl_3$) δ 150.1, 142.9, 141.8, 137.6, 136.7, 129.3, 128.2, 127.4, 127.3, 127.1, 126.3, 121.8, 110.2, 108.8, 50.3, 42.5, 21.5, 4.9, 1.6; IR (neat): ν 2922, 1598, 1497, 1335, 1159, 1093, 1011, 815, 756, 741 cm^{-1}; HRMS (ESI) Calcd. for $C_{23}H_{23}NO_3SNa$ $[M+Na]^+$: 416.1291, Found: 416.1298.

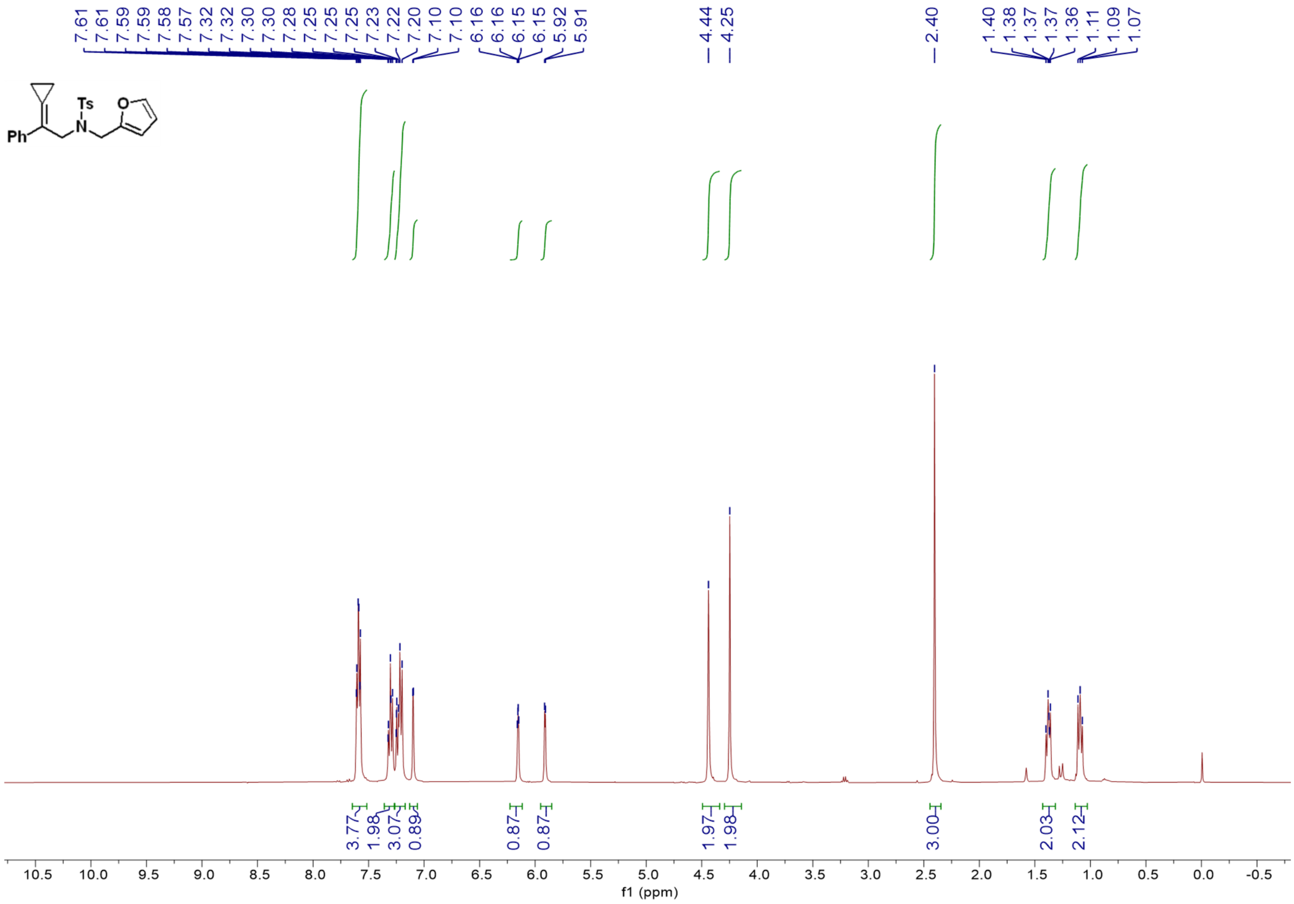
Ts
N
O
Ph
7.61 7.61 7.59 7.59 7.58 7.57 7.32 7.32 7.30 7.30 7.28 7.25 7.25 7.25 7.23 7.22 7.20 7.10 7.10 6.16 6.16 6.15 6.15 5.92 5.91
4.44 4.25
2.40
1.40 1.38 1.37 1.37 1.36 1.11 1.09 1.07
3.77 1.98 3.07 0.89 0.87 0.87 1.97 1.98 3.00 2.03 2.12
10.5 10.0 9.5 9.0 8.5 8.0 7.5 7.0 6.5 6.0 5.5 5.0 4.5 4.0 3.5 3.0 2.5 2.0 1.5 1.0 0.5 0.0 -0.5
f1 (ppm)

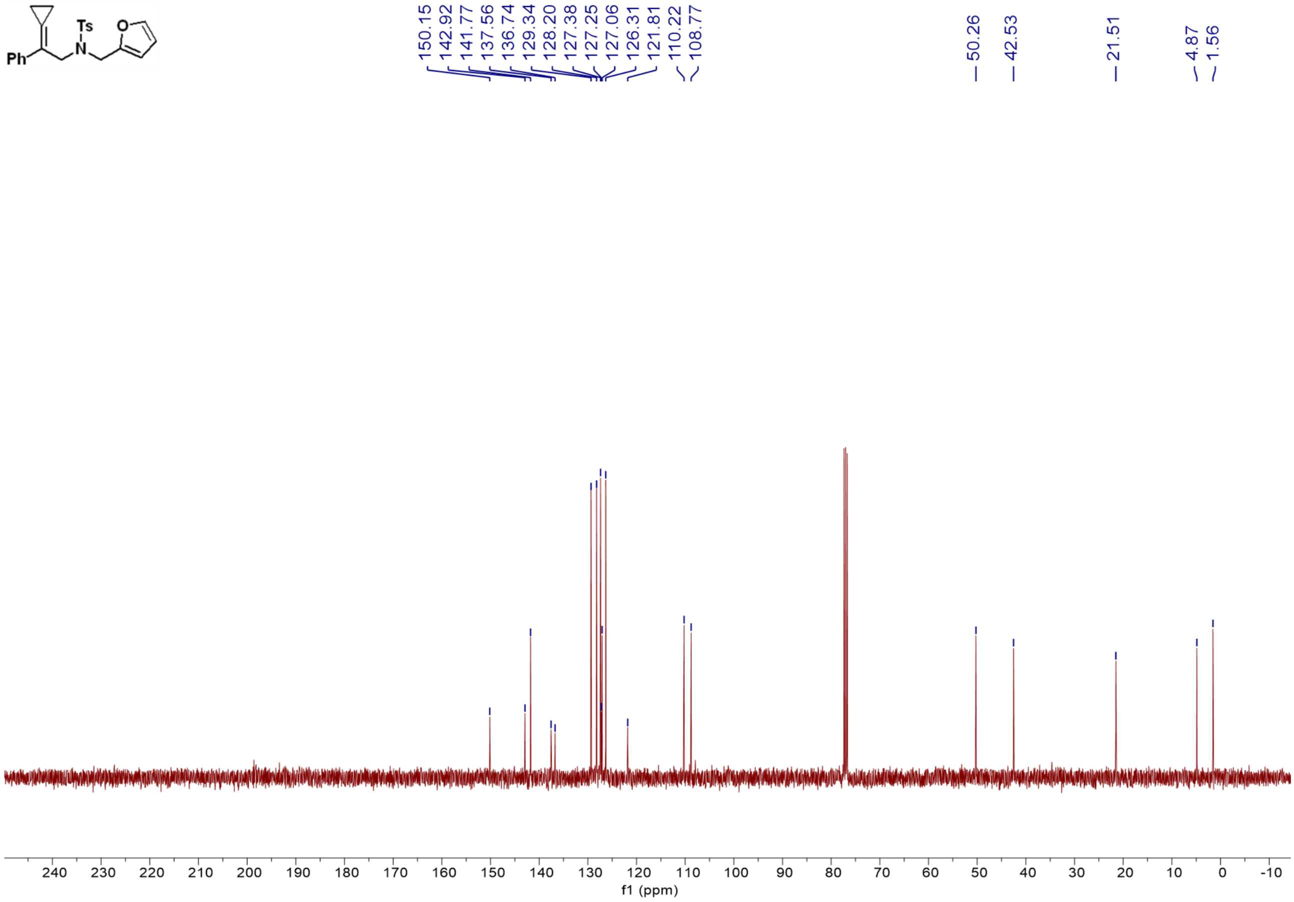
Ts
N
O
Ph
150.15 142.92 141.77 137.56 136.74 129.34 128.20 127.38 127.25 127.06 126.31 121.81 110.22 108.77
50.26 42.53
21.51
4.87 1.56
240 230 220 210 200 190 180 170 160 150 140 130 120 110 100 90 80 70 60 50 40 30 20 10 0 -10
f1 (ppm)

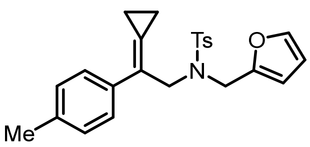

Compound 1b: Yield: 318 mg, 26%; A white solid; M.p.: 81 - 83 ºC; ^{1}H NMR (400 MHz, $CDCl_3$) δ 7.59 (d, *J* = 8.2 Hz, 2H), 7.50 (d, *J* = 8.2 Hz, 2H), 7.21 (d, *J* = 8.0 Hz, 1H), 7.15 - 7.07 (m, 2H), 6.16 (dd, *J* = 3.2, 1.8 Hz, 1H), 5.92 (d, *J* = 3.2 Hz, 1H), 4.42 (s, 2H), 4.25 (s, 2H), 2.41 (s, 3H), 2.34 (s, 3H), 1.40 - 1.32 (m, 2H), 1.11 - 1.04 (m, 2H); ^{13}C NMR (100 MHz, $CDCl_3$) δ 150.2, 142.8, 141.7, 136.84, 136.76, 134.7, 129.3, 128.9, 127.4, 126.2, 121.7, 110.2, 108.8, 50.3, 42.4, 21.5, 21.2, 4.8, 1.5; IR (neat): ν 2975, 1598, 1515, 1349, 1336, 1160, 1093, 1011, 815, 739 cm^{-1}; HRMS (ESI) Calcd. for $C_{24}H_{25}NO_3SNa$ $[M+Na]^+$: 430.1447, Found: 430.1446.

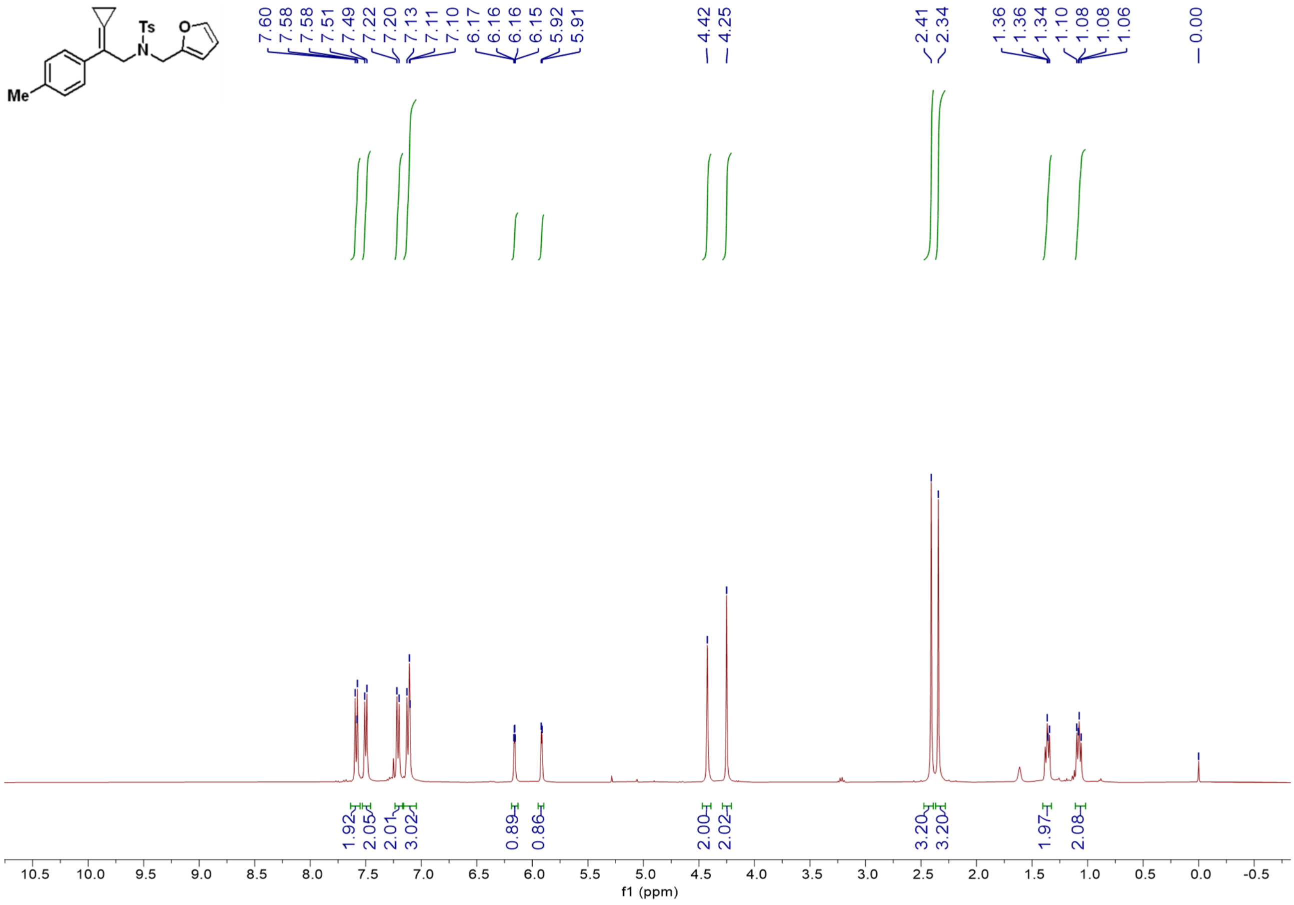
Ts
N
O
Me
7.60 7.58 7.58 7.51 7.49 7.22 7.20 7.13 7.11 7.10 6.17 6.16 6.16 6.15 5.92 5.91
4.42 4.25
2.41 2.34
1.36 1.36 1.34 1.10 1.08 1.08 1.06
0.00
1.92 2.05 2.01 3.02 0.89 0.86 2.00 2.02 3.20 3.20 1.97 2.08
f1 (ppm)

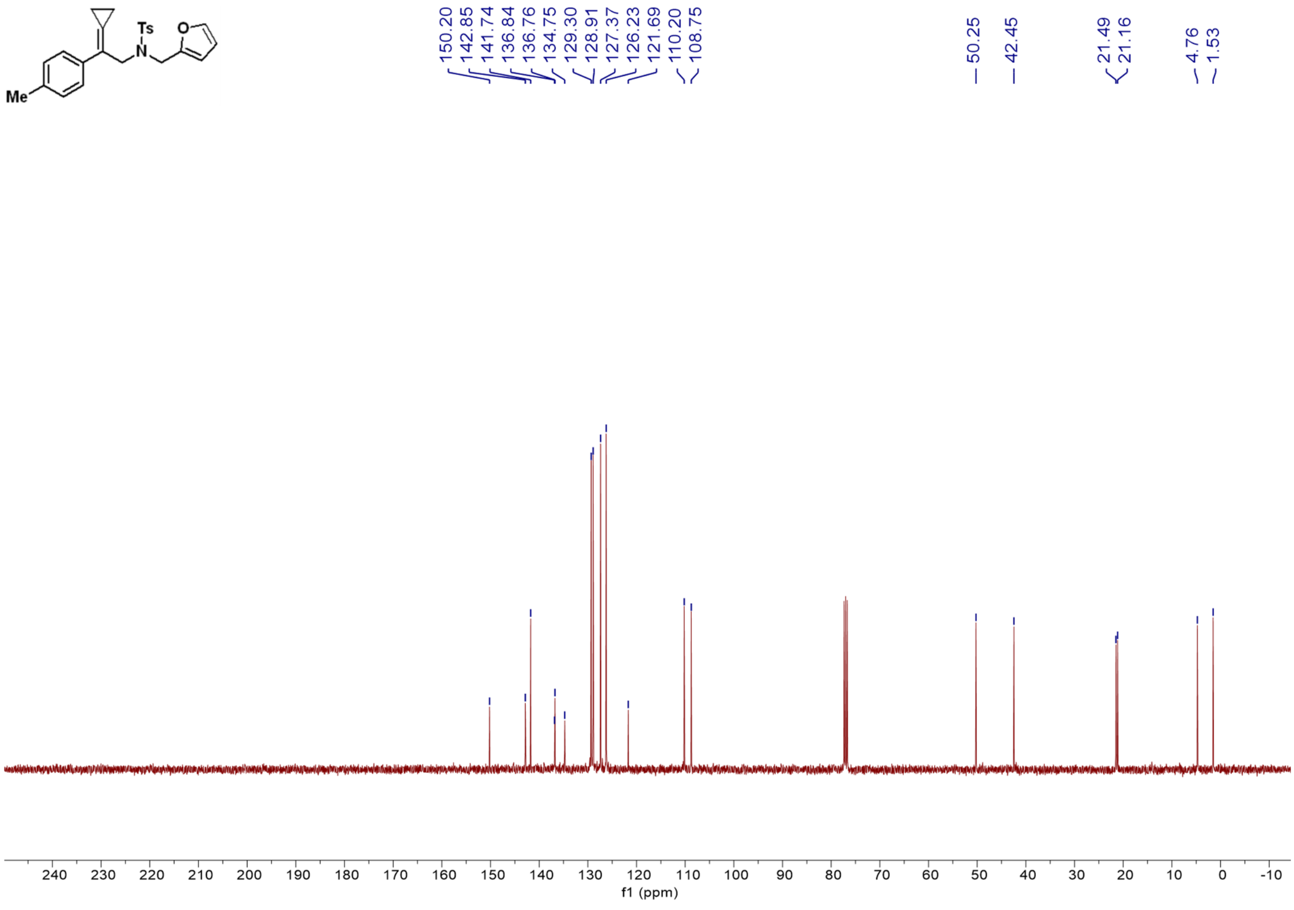
Ts
N
O
Me
150.20 142.85 141.74 136.84 136.76 134.75 129.30 128.91 127.37 126.23 121.69 110.20 108.75
50.25
42.45
21.49 21.16
4.76 1.53
f1 (ppm)

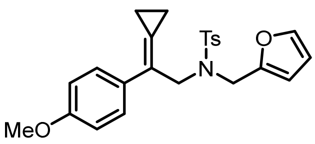

Compound 1c: Yield: 641 mg, 50%; A white solid; M.p.: 89 - 92 ºC; ^{1}H NMR (400 MHz, $CDCl_3$) δ 7.61 - 7.54 (m, 4H), 7.21 (d, *J* = 8.0 Hz, 2H), 7.12 - 7.07 (m, 1H), 6.86 (d, *J* = 8.8 Hz, 1H), 6.15 (dd, *J* = 3.2, 1.8 Hz, 1H), 5.90 (d, *J* = 3.2 Hz, 1H), 4.41 (s, 1H), 4.24 (s, 1H), 3.82 (s, 1H), 2.41 (s, 1H), 1.40 - 1.32 (m, 2H), 1.11 - 1.03 (m, 2H); ^{13}C NMR (100 MHz, $CDCl_3$) δ 158.7, 150.2, 142.9, 141.7, 136.8, 130.2, 129.3, 127.5, 127.4, 125.2, 121.2, 113.6, 110.2, 108.7, 55.2, 50.5, 42.5, 21.5, 4.8, 1.5; IR (neat): ν 2963, 2511, 1598, 1513, 1345, 1258, 1159, 1091, 1024, 802, 744 cm^{-1}; HRMS(ESI) Calcd. for $C_{24}H_{25}NO_4SNa$ $[M+Na]^+$: 446.1397, Found: 446.1392.

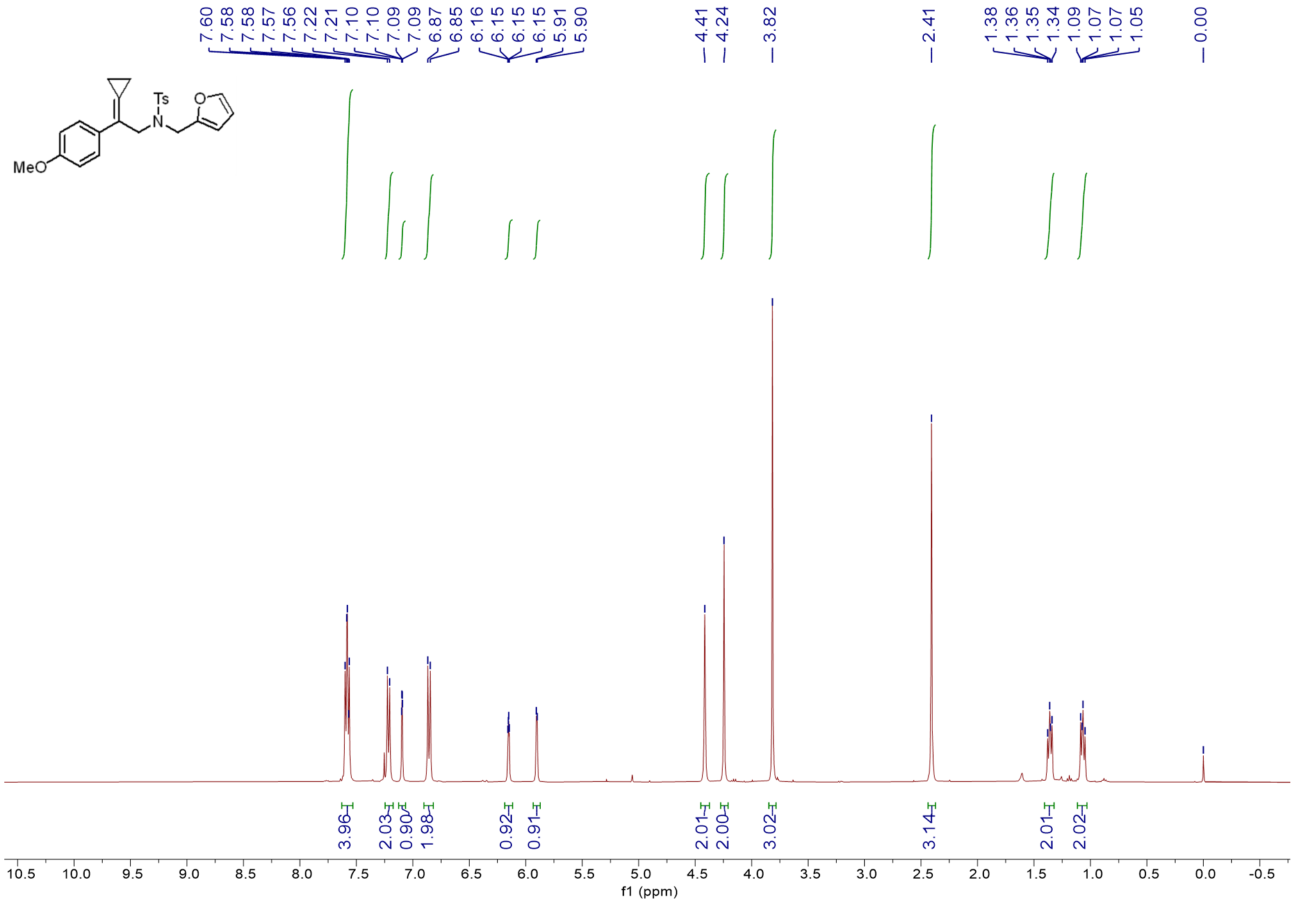
7.60 7.58 7.58 7.57 7.56 7.22 7.21 7.10 7.10 7.09 7.09 6.87 6.85 6.16 6.15 6.15 6.15 5.91 5.90
4.41 4.24 3.82 2.41 1.38 1.36 1.35 1.34 1.09 1.07 1.07 1.05 0.00
Ts
N
O
MeO
3.96 2.03 0.90 1.98 0.92 0.91 2.01 2.00 3.02 3.14 2.01 2.02
f1 (ppm)

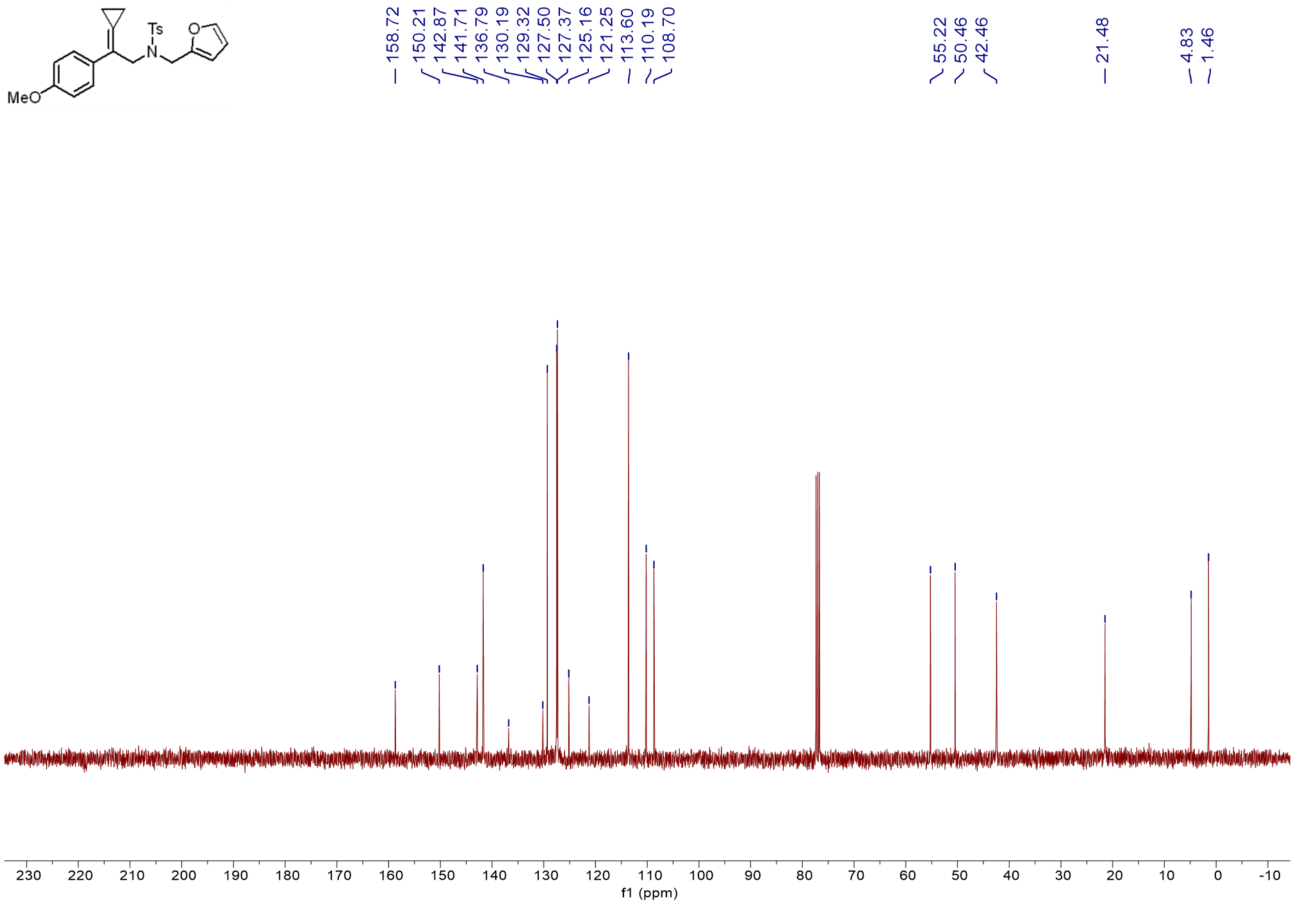
158.72 150.21 142.87 141.71 136.79 130.19 129.32 127.50 127.37 125.16 121.25 113.60 110.19 108.70
55.22 50.46 42.46 21.48 4.83 1.46
Ts
N
O
MeO
f1 (ppm)

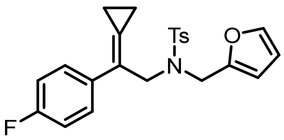

Compound 1d: Yield: 447 mg, 36%; A white solid; M.p.: 85 -87 ºC; ^{1}H NMR (400 MHz, $CDCl_3$) δ 7.66 (d, *J* = 7.9 Hz, 2H), 7.62 - 7.57 (m, 2H), 7.49 - 7.41 (m, 6H), 7.40 - 7.33 (m, 3H), 7.31 - 7.24 (m, 3H), 7.21 - 7.15 (m, 2H), 4.28 (s, 4H), 2.43 (s, 3H), 1.07 - 0.97 (m, 4H), 0.97 - 0.89 (m, 4H); ^{13}C NMR (100 MHz, $CDCl_3$) δ 162.0 (d, *J* = 246.1 Hz), 150.1, 143.0, 141.8, 136.6, 133.6, 129.4, 128.0 (d, *J* = 8.0 Hz), 127.4, 127.0, 121.1, 115.0 (d, *J* = 21.2 Hz), 110.2, 108.8, 50.6, 42.6, 21.5, 4.9, 1.6; ^{19}F NMR (376 MHz, $CDCl_3$) δ -115.3; IR (neat): ν 2978, 1600, 1509, 1336, 1229, 1160, 1092, 813, 739 cm^{-1}; HRMS(ESI) Calcd. for $C_{23}H_{22}NO_3SNaF$ $[M+Na]^+$: 434.1197, Found: 434.1193.

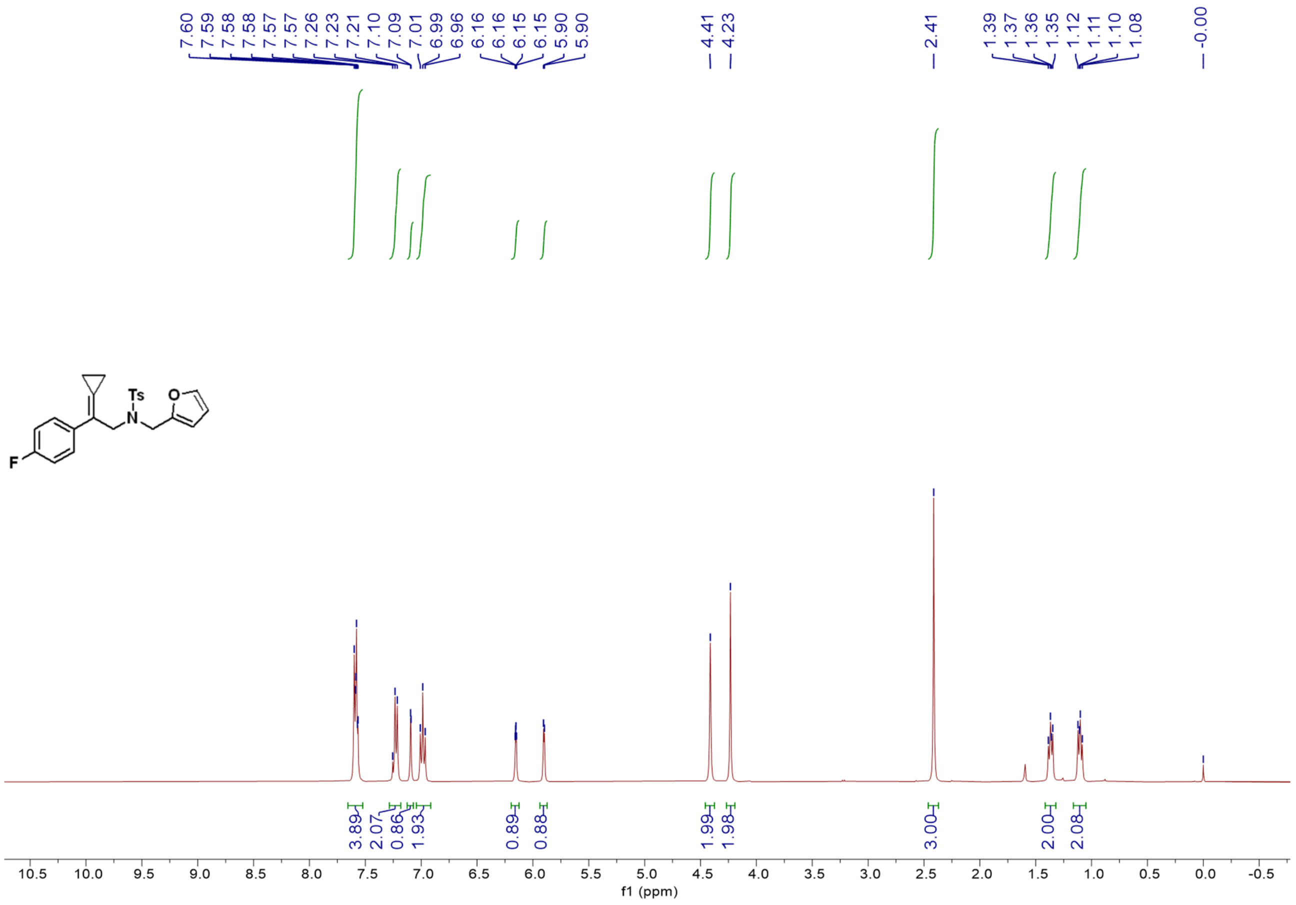
7.60 7.59 7.58 7.58 7.57 7.57 7.26 7.23 7.21 7.10 7.09 7.01 6.99 6.96 6.16 6.16 6.15 6.15 5.90 5.90
4.41 4.23
2.41
1.39 1.37 1.36 1.35 1.12 1.11 1.10 1.08
-0.00
Ts
N
O
F
3.89 2.07 0.86 1.93 0.89 0.88 1.99 1.98 3.00 2.00 2.08
f1 (ppm)

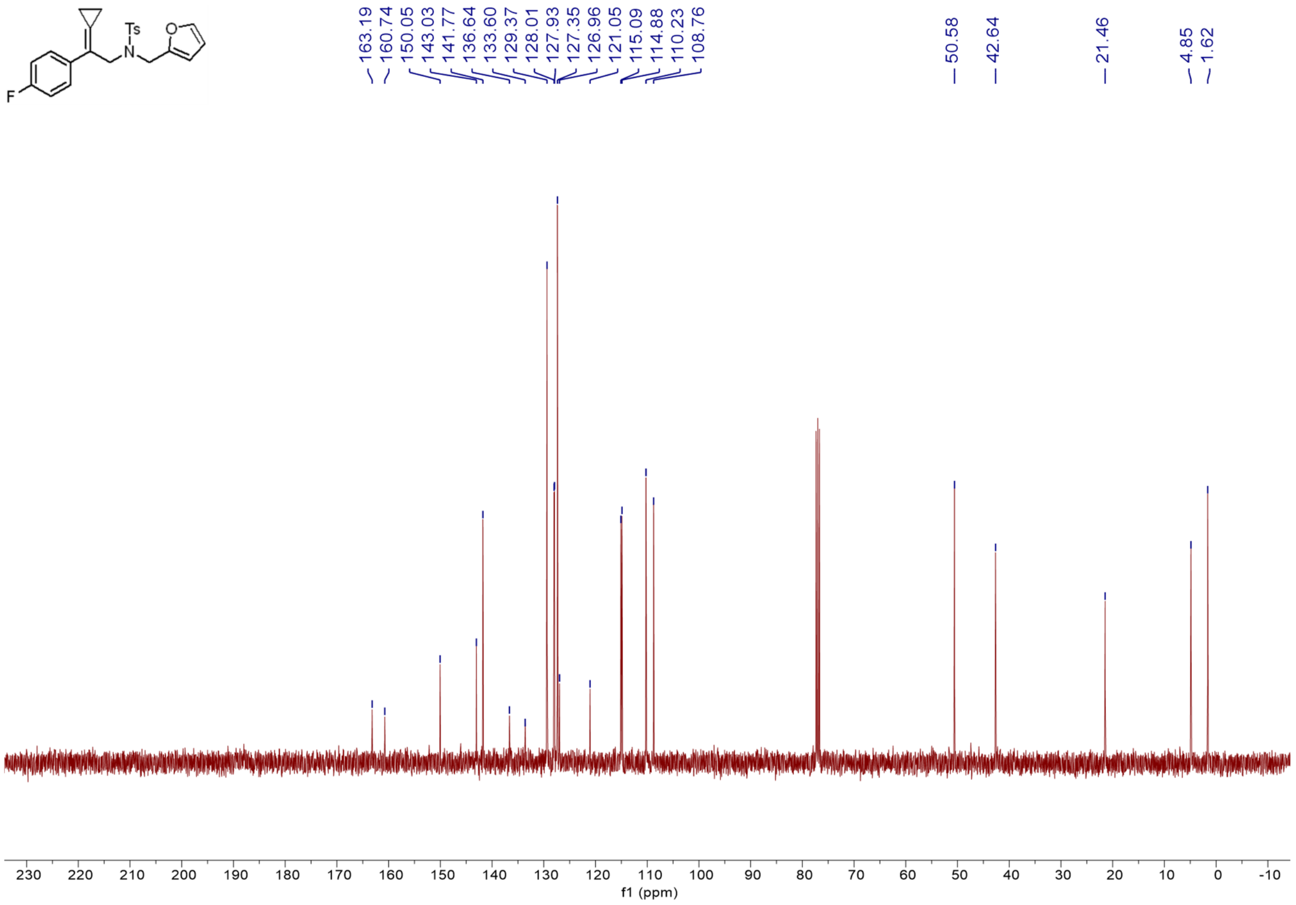
Ts
N
O
F
163.19 160.74 150.05 143.03 141.77 136.64 133.60 129.37 128.01 127.93 127.35 126.96 121.05 115.09 114.88 110.23 108.76
50.58 42.64
21.46
4.85 1.62
f1 (ppm)

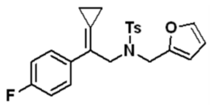

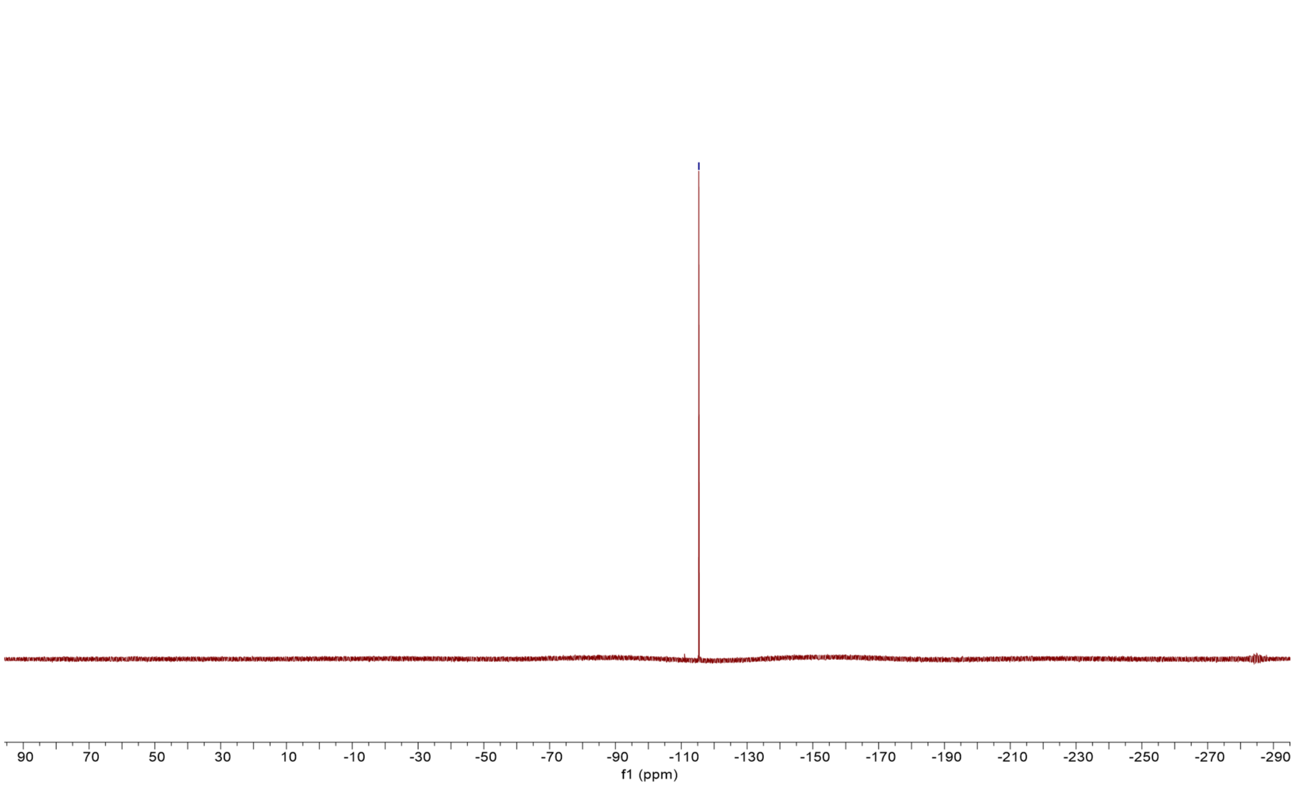

-115.34
90
70
50
30
10
-10
-30
-50
-70
-90
-110
-130
-150
-170
-190
-210
-230
-250
-270
-290
f1 (ppm)

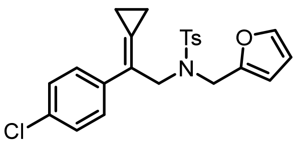

Compound 1e: Yield: 423 mg, 66%; A white solid; M.p.: 83 -86 ºC; ^{1}H NMR (400 MHz, $CDCl_3$) 7.62 - 7.50 (m, 4H), 7.29 - 7.18 (m, 4H), 7.10 (dd, *J* = 2.0, 0.8 Hz, 1H), 6.16 (dd, *J* = 3.2, 2.0 Hz, 1H), 5.91 (d, *J* = 3.2 Hz, 1H), 4.41 (s, 2H), 4.23 (s, 2H), 2.42 (s, 3H), 1.41 - 1.29 (m, 2H), 1.15 - 1.07 (m, 2H); ^{13}C NMR (100 MHz, $CDCl_3$) 150.0, 143.1, 141.8, 136.6, 136.0, 132.8, 129.4, 128.3, 127.9, 127.6, 127.3, 121.1, 110.3, 108.8, 50.4, 42.7, 21.5, 4.9, 1.6; IR (neat): ν 2975, 1597, 1493, 1335, 1160, 1092, 1011,813, 724, cm^{-1}; HRMS (ESI) Calcd. for $C_{23}H_{22}NO_3SNaCl$ $[M+Na]^+$: 450.0901, Found: 450.0906.

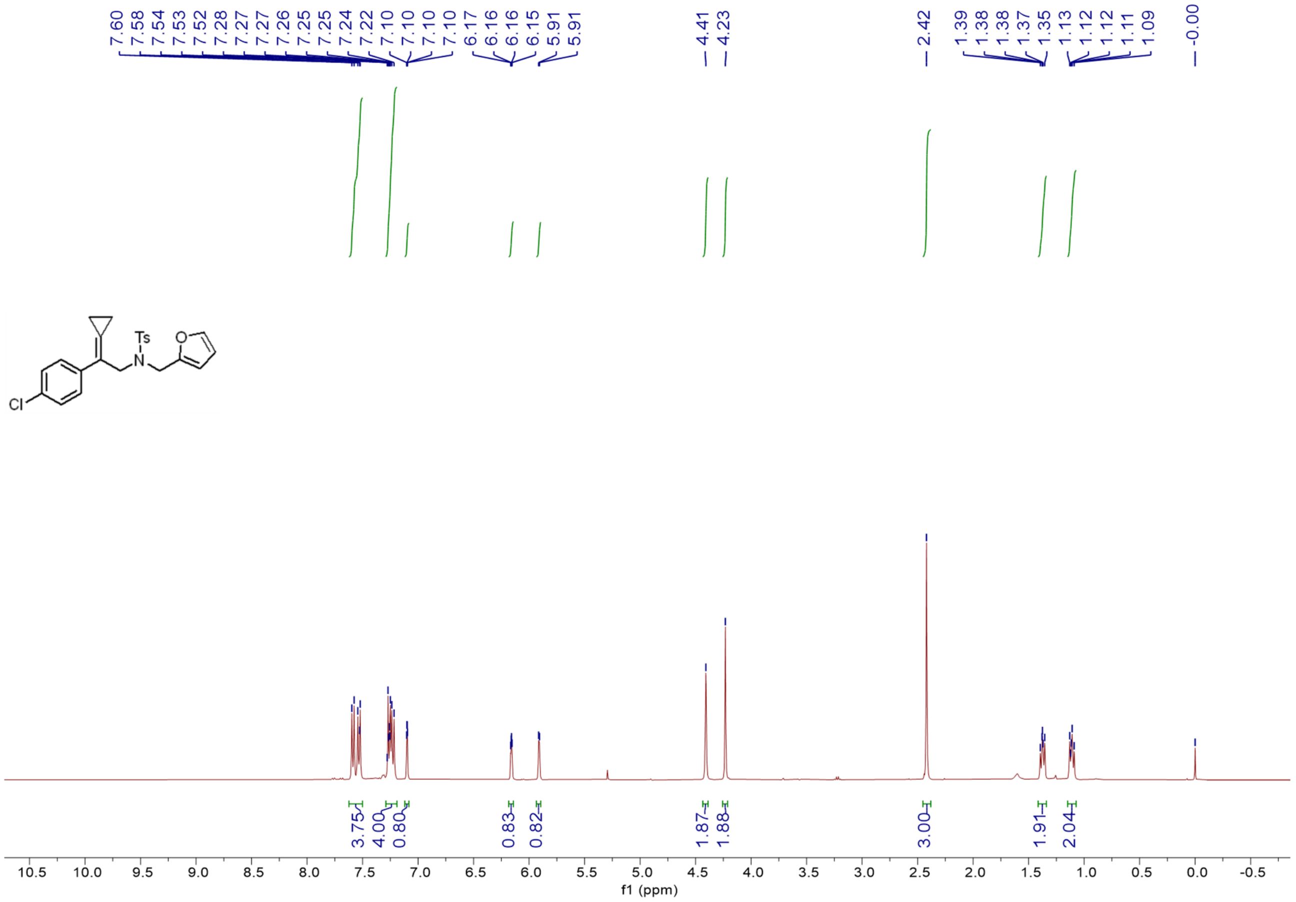
7.60 7.58 7.54 7.53 7.52 7.28 7.27 7.27 7.26 7.25 7.25 7.24 7.22 7.10 7.10 7.10 7.10 6.17 6.16 6.16 6.15 5.91 5.91
4.41 4.23
2.42
1.39 1.38 1.38 1.37 1.35 1.13 1.12 1.12 1.11 1.09
-0.00
Ts
N
O
Cl
3.75 4.00 0.80 0.83 0.82 1.87 1.88 3.00 1.91 2.04
f1 (ppm)

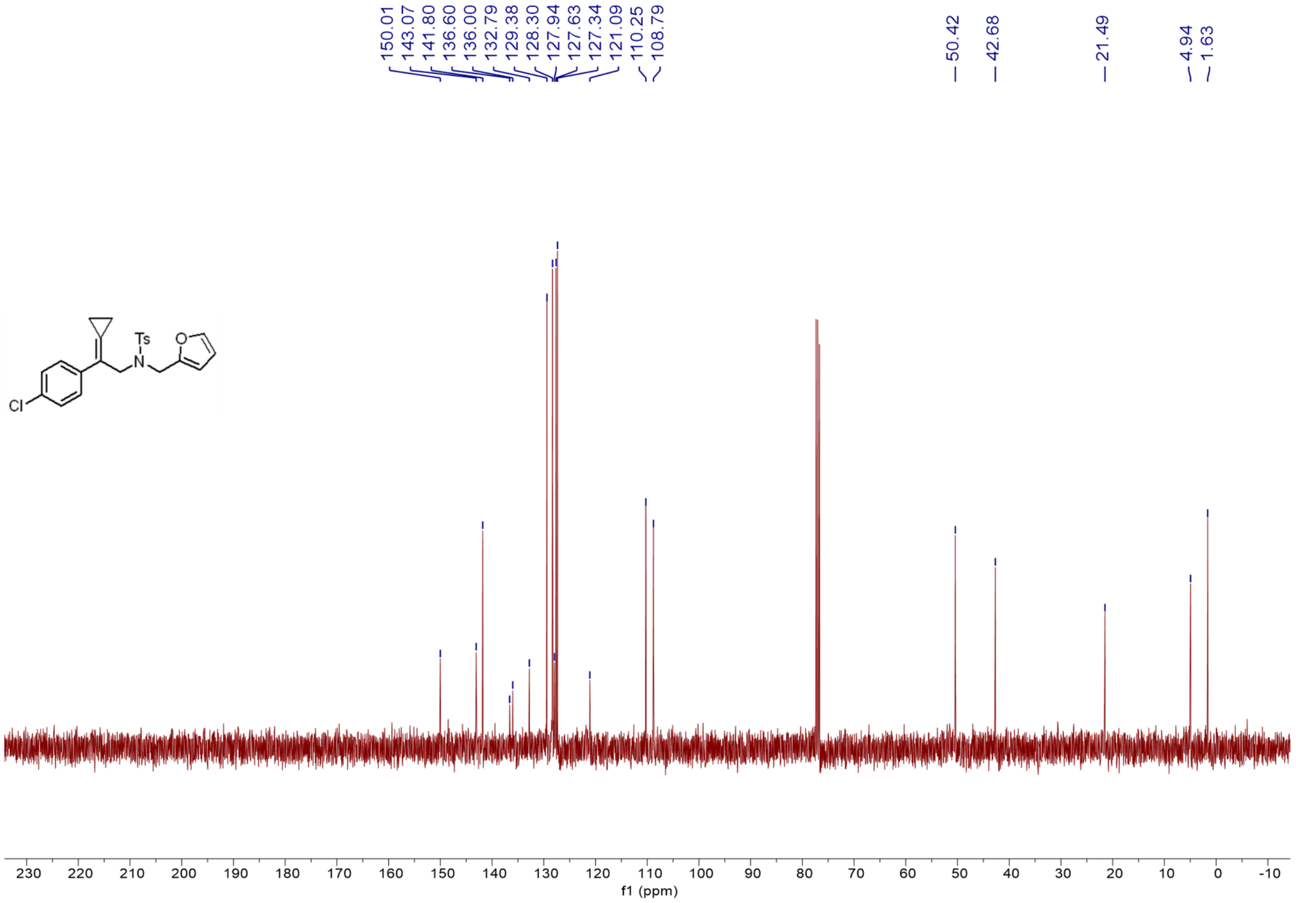
150.01 143.07 141.80 136.60 136.00 132.79 129.38 128.30 127.94 127.63 127.34 121.09 110.25 108.79
50.42 42.68
21.49
4.94 1.63
Ts
N
O
Cl
f1 (ppm)

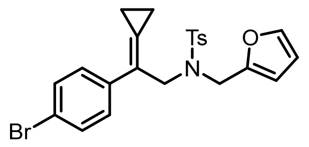

Compound 1f: Yield: 603 mg, 43%; A white solid; M.p.: 85 - 88 °C; ^{1}H NMR (400 MHz, $CDCl_3$) δ 7.58 (d, *J* = 8.0 Hz, 2H), 7.48 (d, *J* = 8.4 Hz, 2H), 7.41 (d, *J* = 8.4 Hz, 2H), 7.22 (d, *J* = 8.0 Hz, 2H), 7.10 (d, *J* = 1.8 Hz, 1H), 6.16 (dd, *J* = 3.4, 1.8 Hz, 1H), 5.91 (d, *J* = 3.4 Hz, 1H), 4.40 (s, 1H), 4.23 (s, 1H), 2.42 (s, 2H), 1.40 - 1.32 (m, 2H), 1.15 - 1.05 (m, 2H); ^{13}C NMR (100 MHz, $CDCl_3$) δ 150.0, 143.1, 141.8, 136.5, 136.4, 131.2, 129.4, 128.1, 128.0, 127.3, 121.1, 121.0, 110.3, 108.8, 50.4, 42.7, 21.5, 5.0, 1.6; IR (neat): ν 2922, 1711, 1490, 1336, 1159, 1091, 1007, 813, 745, 717 cm^{-1}; HRMS (ESI) Calcd. for $C_{23}H_{22}NO_3SNaBr$ $[M+H]^+$: 494.0396, Found: 494.0393.

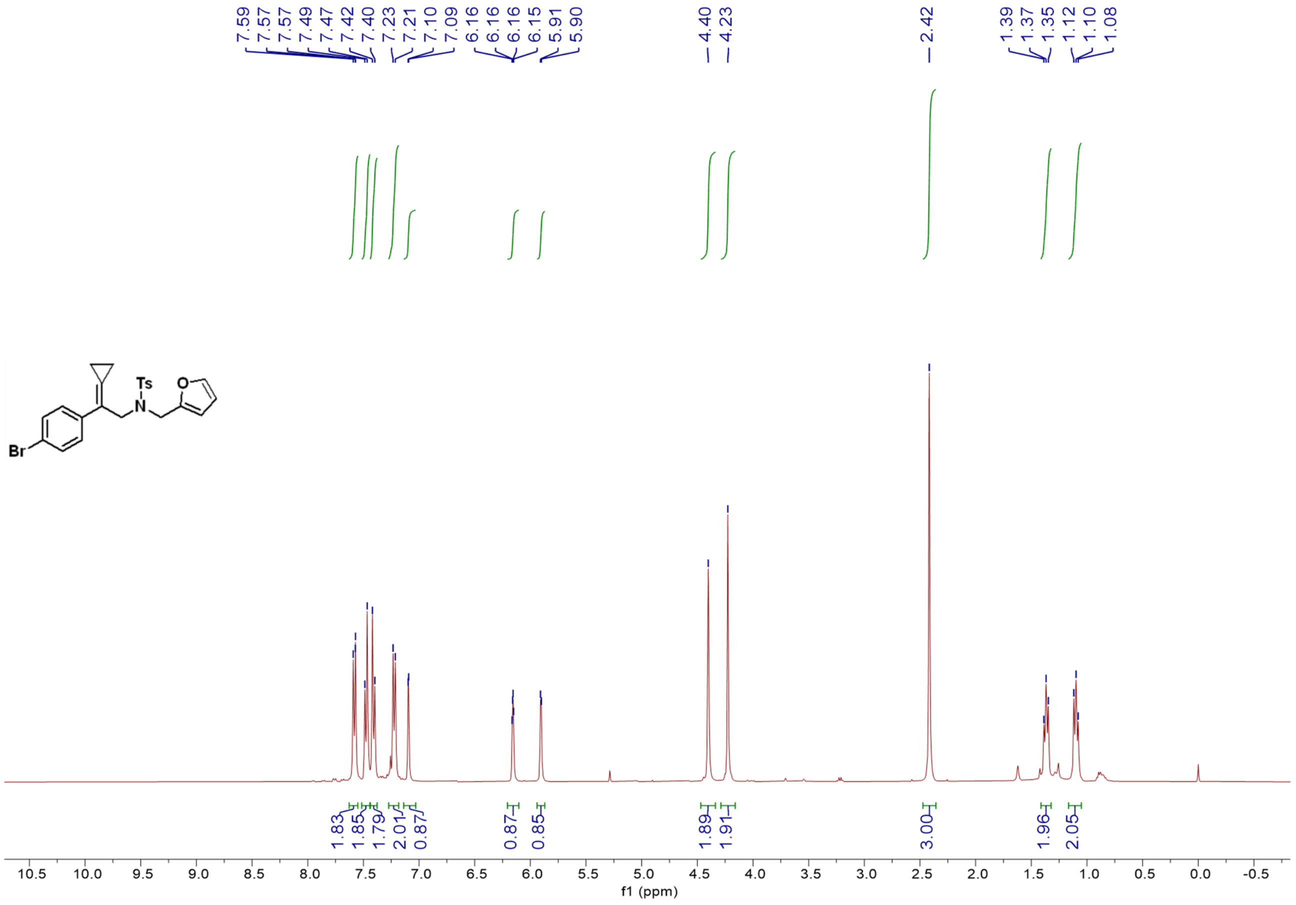
7.59
7.57
7.57
7.49
7.47
7.42
7.40
7.23
7.21
7.10
7.09
6.16
6.16
6.16
6.15
5.91
5.90
4.40
4.23
2.42
1.39
1.37
1.35
1.12
1.10
1.08
Ts
N
O
Br
1.83
1.85
1.79
2.01
0.87
0.87
0.85
1.89
1.91
3.00
1.96
2.05
f1 (ppm)

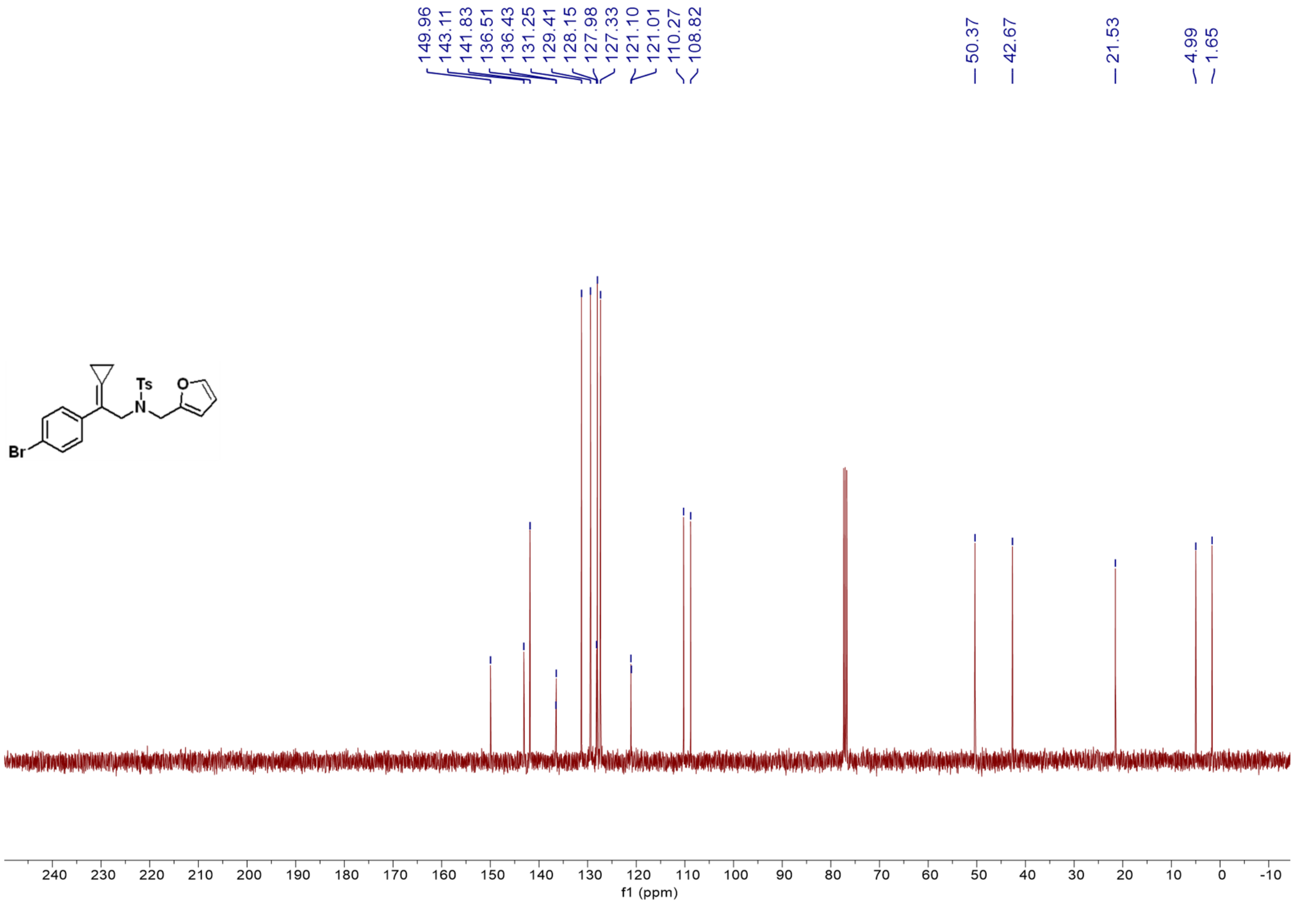
149.96
143.11
141.83
136.51
136.43
131.25
129.41
128.15
127.98
127.33
121.10
121.01
110.27
108.82
50.37
42.67
21.53
4.99
1.65
Ts
N
O
Br
f1 (ppm)

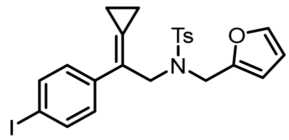

Compound 1g: Yield: 707 mg, 45%; A white solid; M.p.: 89 - 92 ºC; ^{1}H NMR (400 MHz, $CDCl_3$) δ 7.65 - 7.55 (m, 4H), 7.34 (d, *J* = 8.6 Hz, 2H), 7.22 (d, *J* = 8.0 Hz, 2H), 7.10 (d, *J* = 1.8 Hz, 1H), 6.19 – 6.14 (m, 1H), 5.91 (d, *J* = 3.2 Hz, 1H), 4.40 (s, 2H), 4.23 (s, 2H), 2.42 (s, 3H), 1.40 - 1.32 (m, 2H), 1.13 - 1.05 (m, 2H); ^{13}C NMR (100 MHz, $CDCl_3$) δ 150.0, 143.1, 141.8, 137.2, 137.1, 136.6, 129.4, 128.2, 127.3, 121.3, 110.3, 108.8, 97.4, 92.6, 50.3, 42.7, 21.5, 4.9, 1.6; IR (neat): ν 2964, 1488, 1336, 1260, 1159, 1092, 1030, 1003, 811, 745 cm^{-1}; HRMS (ESI) Calcd. for $C_{23}H_{22}NO_3SNaI$ $[M+Na]^+$: 542.0257, Found: 542.0266.

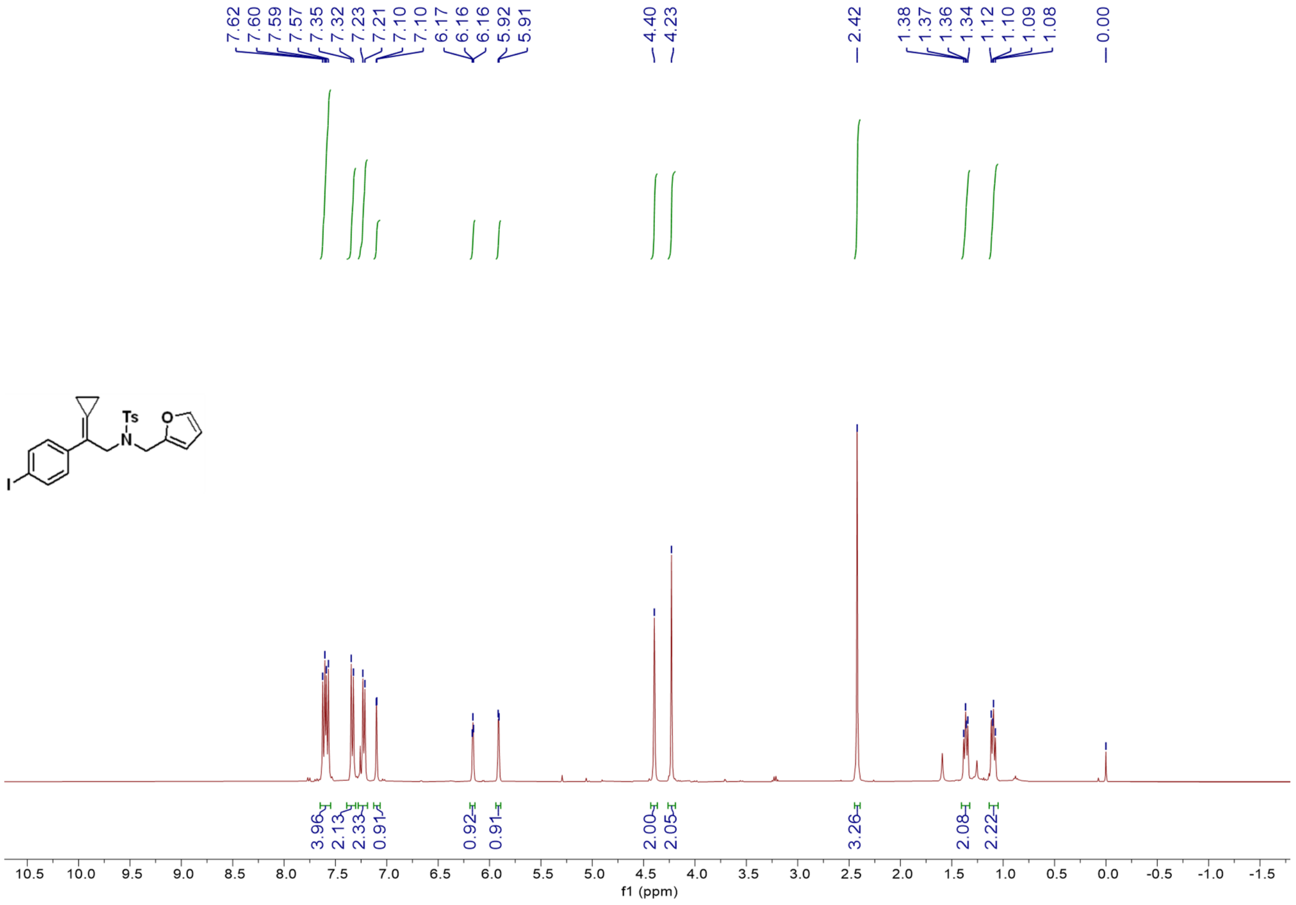

7.62 7.60 7.59 7.57 7.35 7.32 7.23 7.21 7.10 7.10 6.17 6.16 6.16 5.92 5.91
4.40 4.23
2.42
1.38 1.37 1.36 1.34 1.12 1.10 1.09 1.08
0.00
Ts
N
O
I
3.96 2.13 2.33 0.91 0.92 0.91 2.00 2.05 3.26 2.08 2.22
10.5 10.0 9.5 9.0 8.5 8.0 7.5 7.0 6.5 6.0 5.5 5.0 4.5 4.0 3.5 3.0 2.5 2.0 1.5 1.0 0.5 0.0 -0.5 -1.0 -1.5
f1 (ppm)

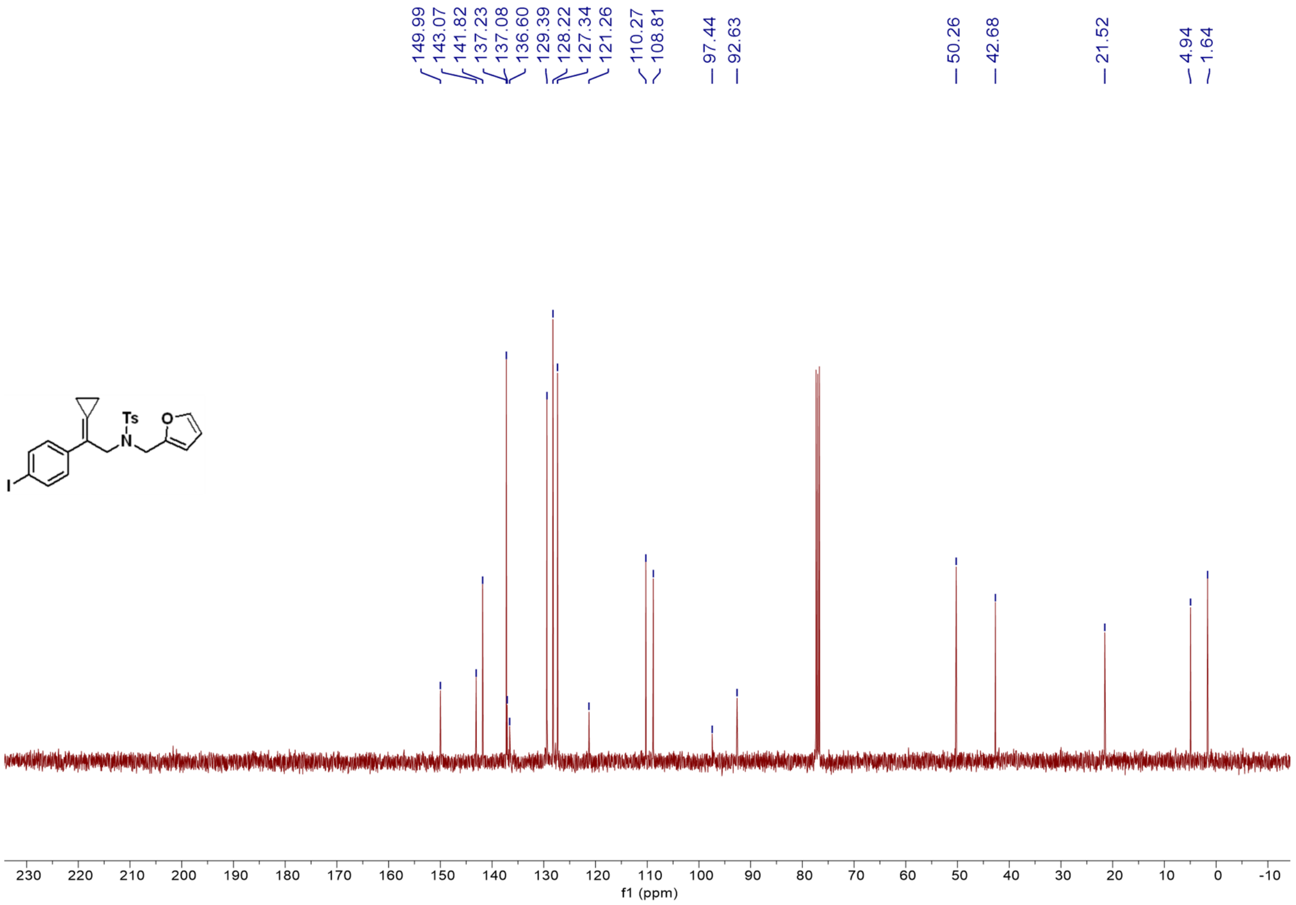

149.99 143.07 141.82 137.23 137.08 136.60 129.39 128.22 127.34 121.26
110.27 108.81
97.44 92.63
50.26 42.68
21.52
4.94 1.64
Ts
N
O
I
230 220 210 200 190 180 170 160 150 140 130 120 110 100 90 80 70 60 50 40 30 20 10 0 -10
f1 (ppm)

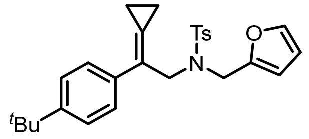

Compound 1h: Yield: 402 mg, 30%; A white solid; M.p.: 86 - 89 ºC; ^{1}H NMR (400 MHz, $CDCl_3$) δ 7.57 (dd, *J* = 8.4, 6.2 Hz, 4H), 7.34 (d, *J* = 8.4 Hz, 2H), 7.21 (d, *J* = 8.0 Hz, 1H), 7.12 - 7.08 (m, 1H), 6.16 (dd, *J* = 3.2, 1.8 Hz, 1H), 5.92 (d, *J* = 3.2 Hz, 1H), 4.44 (s, 2H), 4.26 (s, 2H), 2.41 (s, 3H), 1.41 - 1.35 (m, 2H), 1.33 (s, 9H), 1.11 - 1.04 (m, 2H); ^{13}C NMR (100 MHz, $CDCl_3$) δ 150.2, 149.9, 142.8, 141.7, 136.9, 134.7, 129.3, 127.4, 126.3, 125.9, 125.1, 121.5, 110.2, 108.7, 50.2, 42.4, 34.5, 31.3, 21.5, 4.8, 1.5; IR (neat): ν 2963, 1458 , 1337, 1261, 1093, 1032, 814, 804, 719 cm^{-1}; HRMS (ESI) Calcd. for $C_{27}H_{31}NO_3SNa$ $[M+H]^+$: 472.1917, Found: 472.1919.

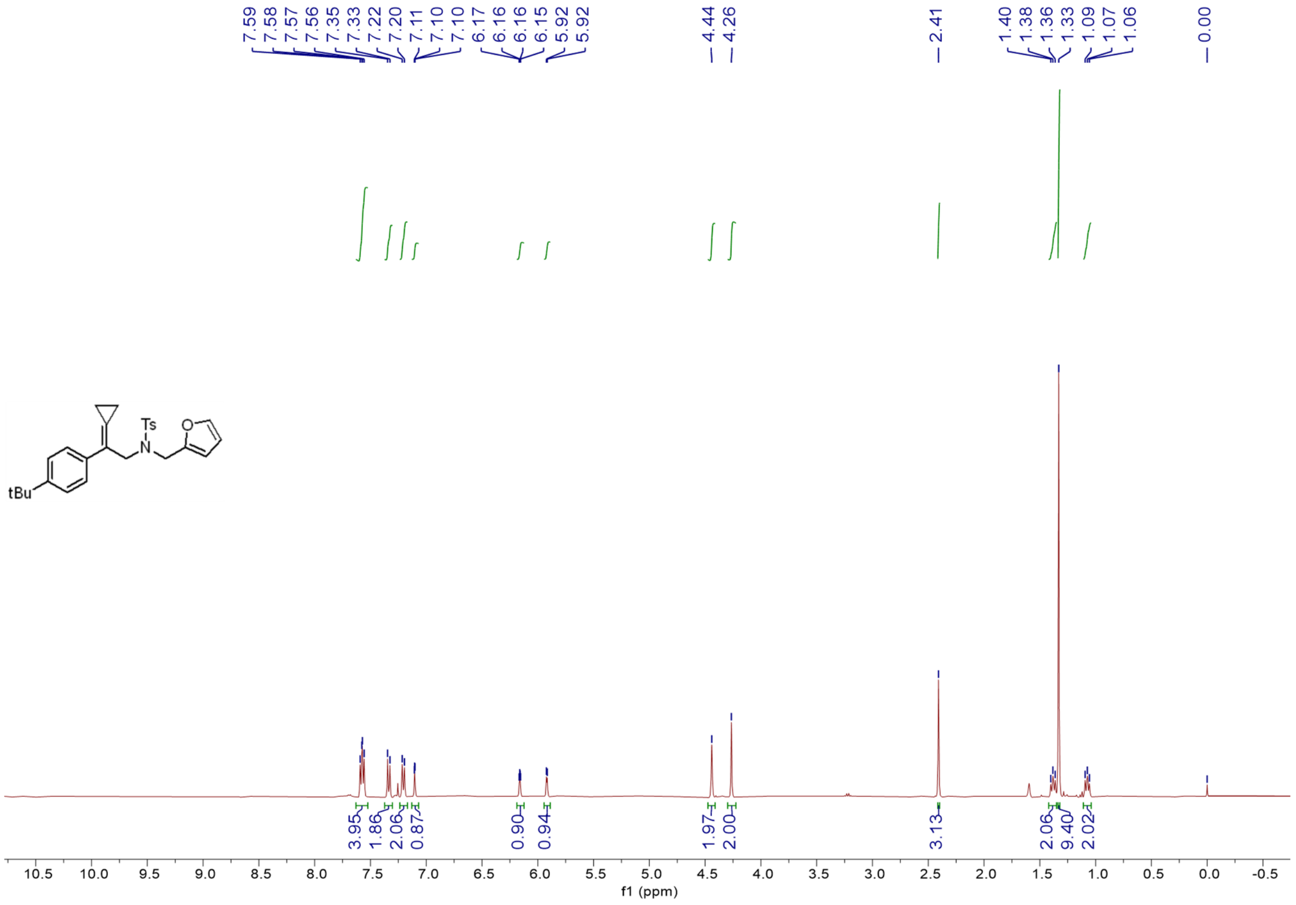
7.59 7.58 7.57 7.56 7.35 7.33 7.22 7.20 7.11 7.10 7.10 6.17 6.16 6.16 6.15 5.92 5.92
4.44 4.26
2.41
1.40 1.38 1.36 1.33 1.09 1.07 1.06
0.00
Ts
N
O
tBu
3.95 1.86 2.06 0.87 0.90 0.94 1.97 2.00 3.13 2.06 9.40 2.02
f1 (ppm)

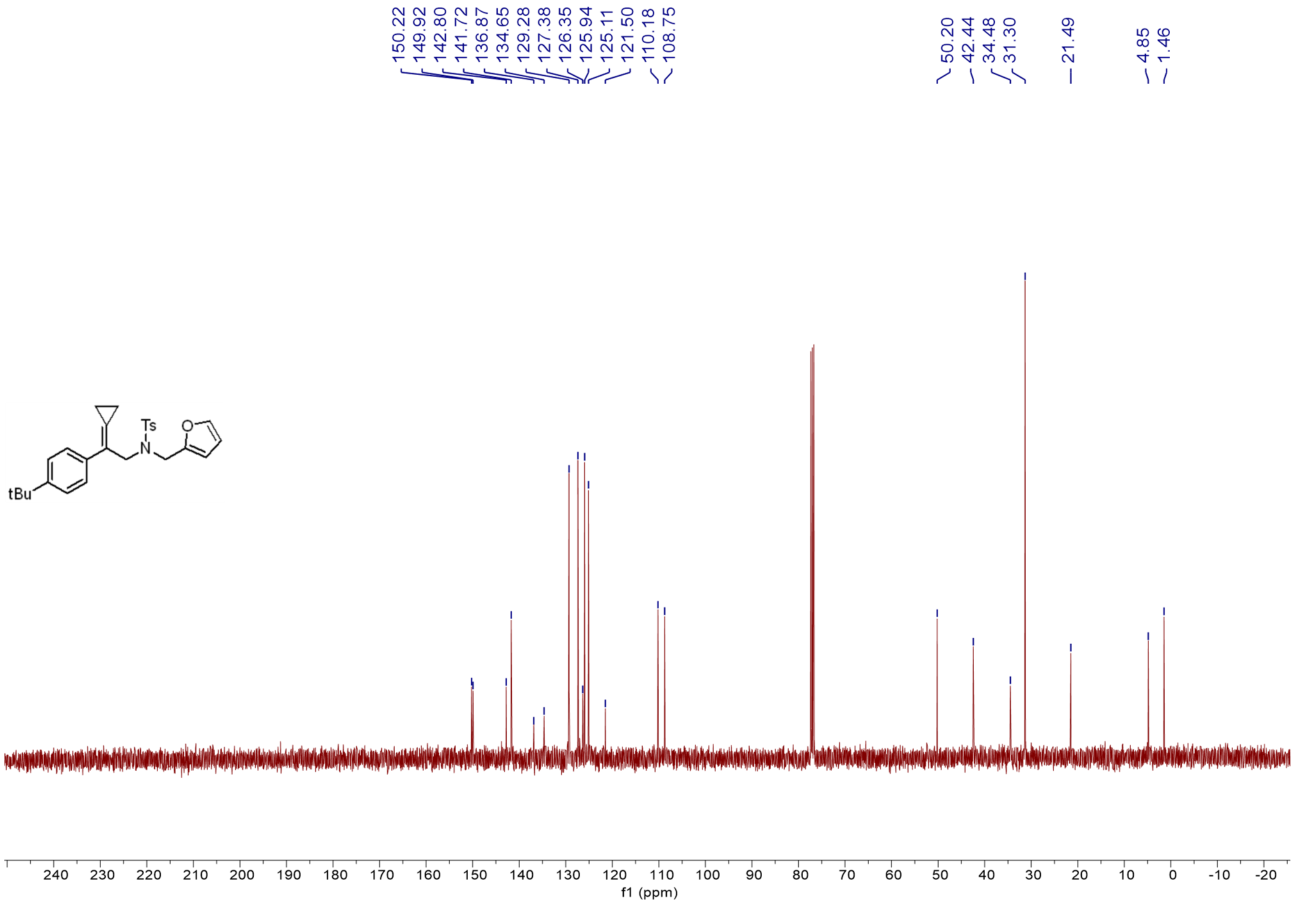
150.22 149.92 142.80 141.72 136.87 134.65 129.28 127.38 126.35 125.94 125.11 121.50 110.18 108.75
50.20 42.44 34.48 31.30
21.49
4.85 1.46
Ts
N
O
tBu
f1 (ppm)

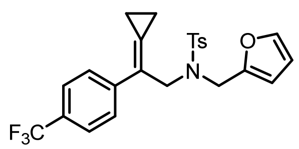

Compound 1i: Yield: 591 mg, 43%; A white solid; M.p.: 90 - 94 ºC; ^{1}H NMR (400 MHz, $CDCl_3$) δ 7.69 (d, *J* = 8.0 Hz, 2H), 7.59 (d, *J* = 8.0 Hz, 2H), 7.54 (d, *J* = 8.0 Hz, 2H), 7.23 (d, *J* = 8.0 Hz, 2H), 7.13 - 7.07 (m, 1H), 6.19 - 6.13 (m, 1H), 5.92 (d, *J* = 3.2 Hz, 1H), 4.45 (s, 2H), 4.24 (s, 2H), 2.42 (s, 3H), 1.58 (s, 3H), 1.46 - 1.38 (m, 2H), 1.20 - 1.12 (m, 2H); ^{13}C NMR (100 MHz, $CDCl_3$) δ 149.9, 143.2, 141.9, 141.1, 136.5, 130.0, 129.4, 128.8 (q, *J* = 32.5 Hz), 127.4, 126.5, 125.1 (q, *J* = 3.8 Hz), 124.3 (q, *J* = 271.6 Hz), 121.3, 110.3, 108.9, 50.4, 42.8, 21.5, 5.1, 1.8; ^{19}F NMR (376 MHz, $CDCl_3$) δ -62.48; IR (neat): ν 2963, 1617, 1326, 1160, 1120, 1074, 1014, 846, 804, 747 cm^{-1}; HRMS (ESI) Calcd. for $C_{24}H_{22}NO_3SNaF_3$ $[M+Na]^+$: 484.1165, Found: 484.1163.

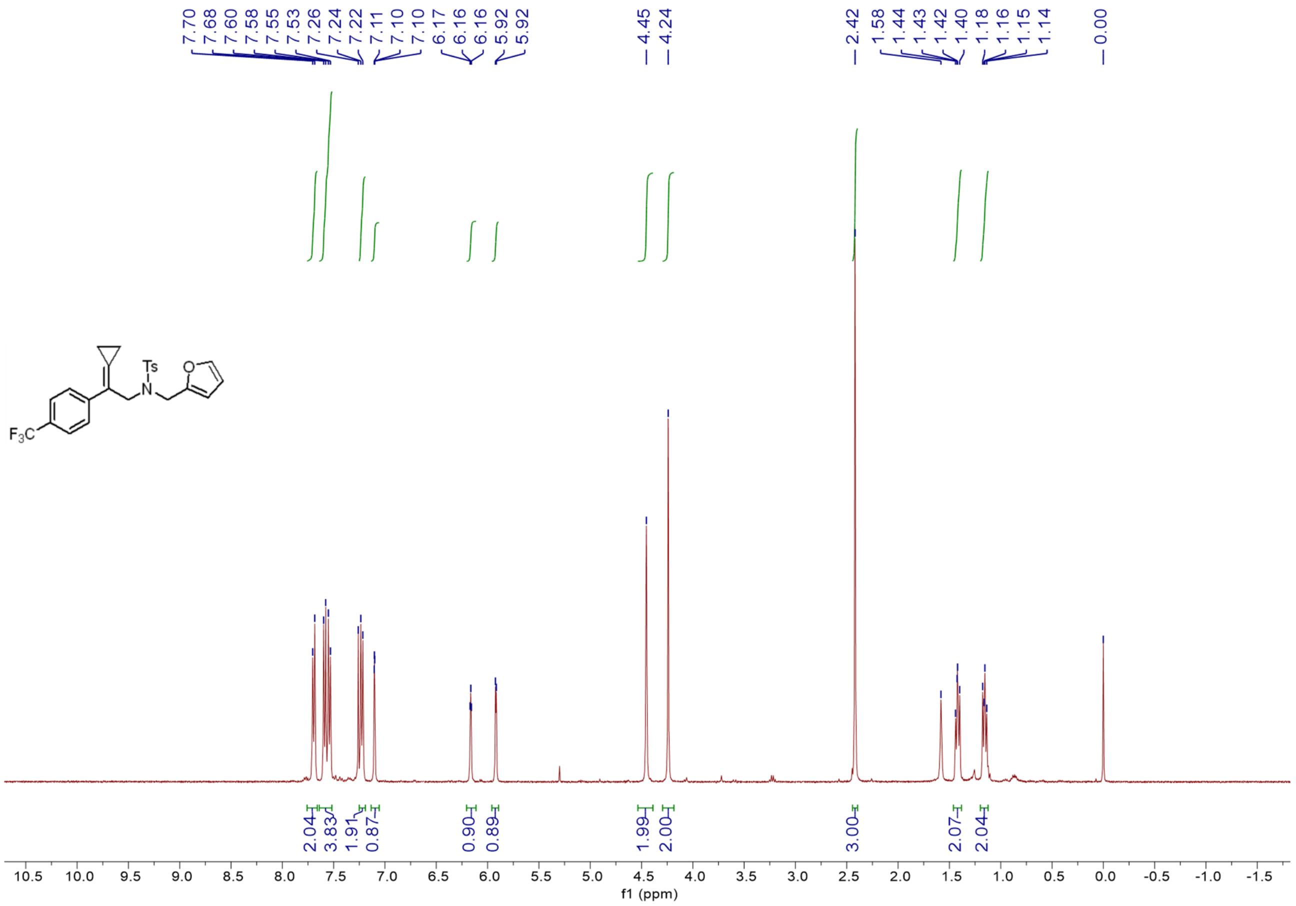
7.70
7.68
7.60
7.58
7.55
7.53
7.26
7.24
7.22
7.11
7.10
7.10
6.17
6.16
6.16
5.92
5.92
4.45
4.24
2.42
1.58
1.44
1.43
1.42
1.40
1.18
1.16
1.15
1.14
0.00
Ts
N
O
F3C
2.04
3.83
1.91
0.87
0.90
0.89
1.99
2.00
3.00
2.07
2.04
f1 (ppm)

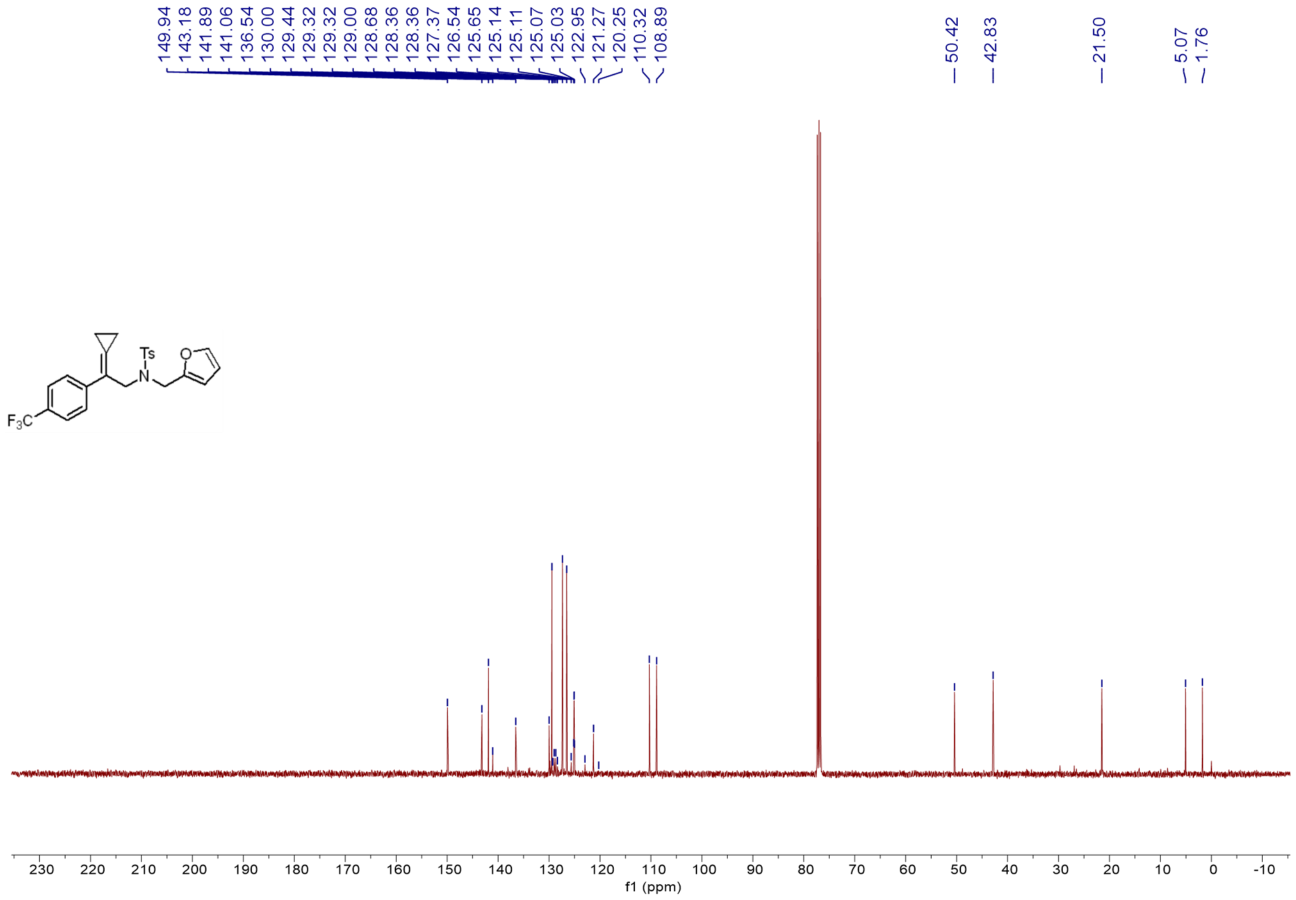
149.94
143.18
141.89
141.06
136.54
130.00
129.44
129.32
129.32
129.00
128.68
128.36
128.36
127.37
126.54
125.65
125.14
125.11
125.07
125.03
122.95
121.27
120.25
110.32
108.89
50.42
42.83
21.50
5.07
1.76
Ts
N
O
F3C
f1 (ppm)

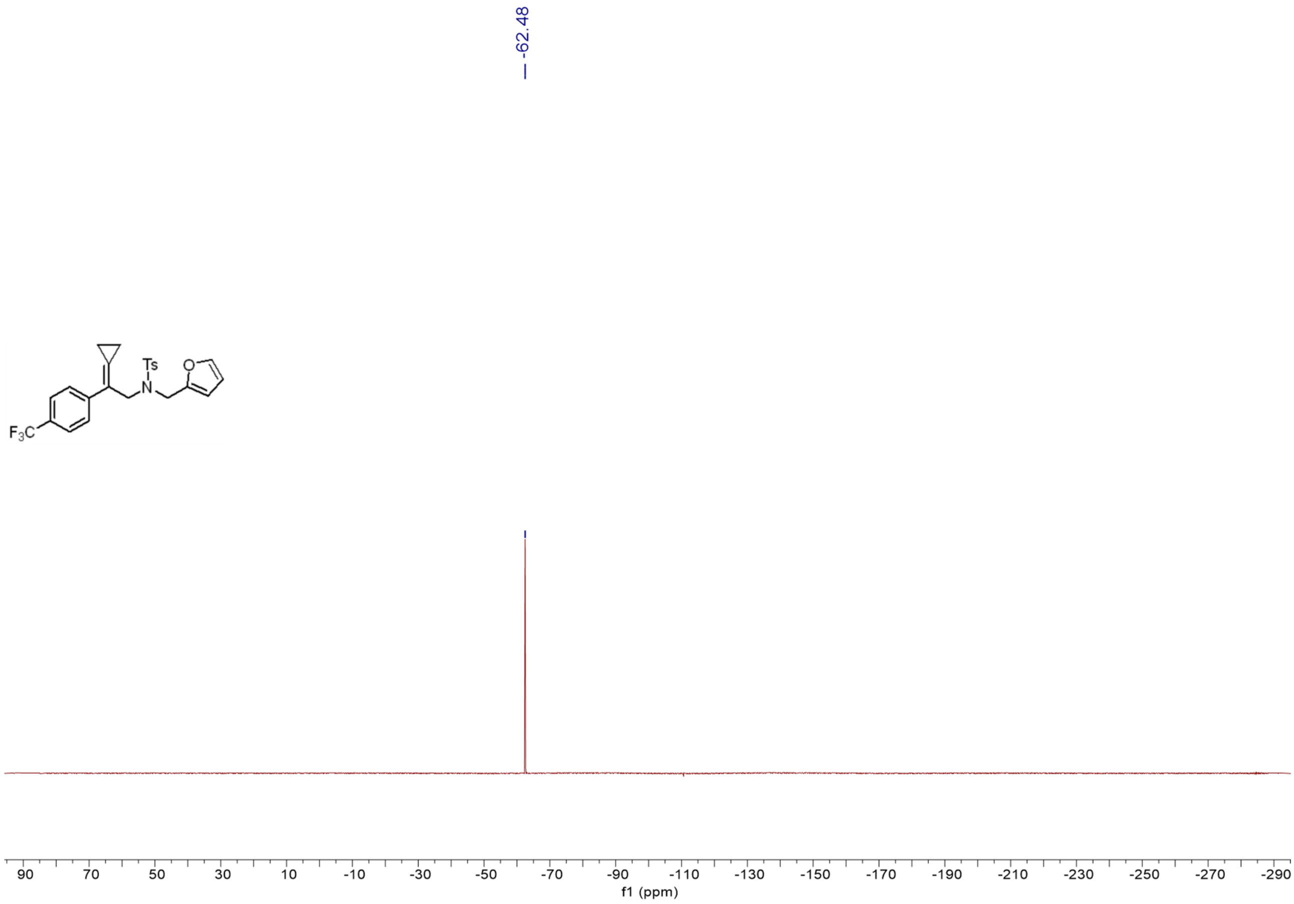

-62.48
Ts
N
O
F3C
90 70 50 30 10 -10 -30 -50 -70 -90 -110 -130 -150 -170 -190 -210 -230 -250 -270 -290
f1 (ppm)

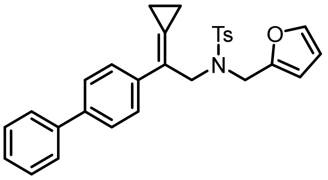

Compound 1j: Yield: 423 mg, 30%; A white solid; M.p.: 83 - 85 ºC; ^{1}H NMR (400 MHz, $CDCl_3$) δ 7.73 - 7.69 (m, 2H), 7.65 - 7.59 (m, 4H), 7.58 - 7.54 (m, 2H), 7.48 - 7.42 (m, 1H), 7.38 - 7.32 (m, 1H), 7.22 (d, J = 8.2 Hz, 2H), 7.13 - 7.10 (m, 1H), 6.17 (dd, J = 3.2, 1.6 Hz, 1H), 5.94 (d, J = 3.2 Hz, 1H), 4.49 (s, 2H), 4.29 (s, 2H), 2.40 (s, 3H), 1.48 - 1.39 (m, 2H), 1.67 - 1.09 (m, 2H); ^{13}C NMR (100 MHz, $CDCl_3$) δ 150.2, 143.0, 141.8, 140.8, 139.7, 136.7, 136.6, 129.6, 129.4, 128.8, 127.2, 126.9, 126.9, 126.7, 121.5, 110.3, 108.8, 50.4, 42.6, 40.1, 21.5, 5.0, 1.6; IR (neat): ν 2924, 1598, 1488, 1331, 1159, 1093, 1011, 814, 730 cm^{-1}; HRMS (ESI) Calcd. for $C_{29}H_{27}NO_3SNa$ $[M+Na]^+$: 492.1604, Found: 492.1609.

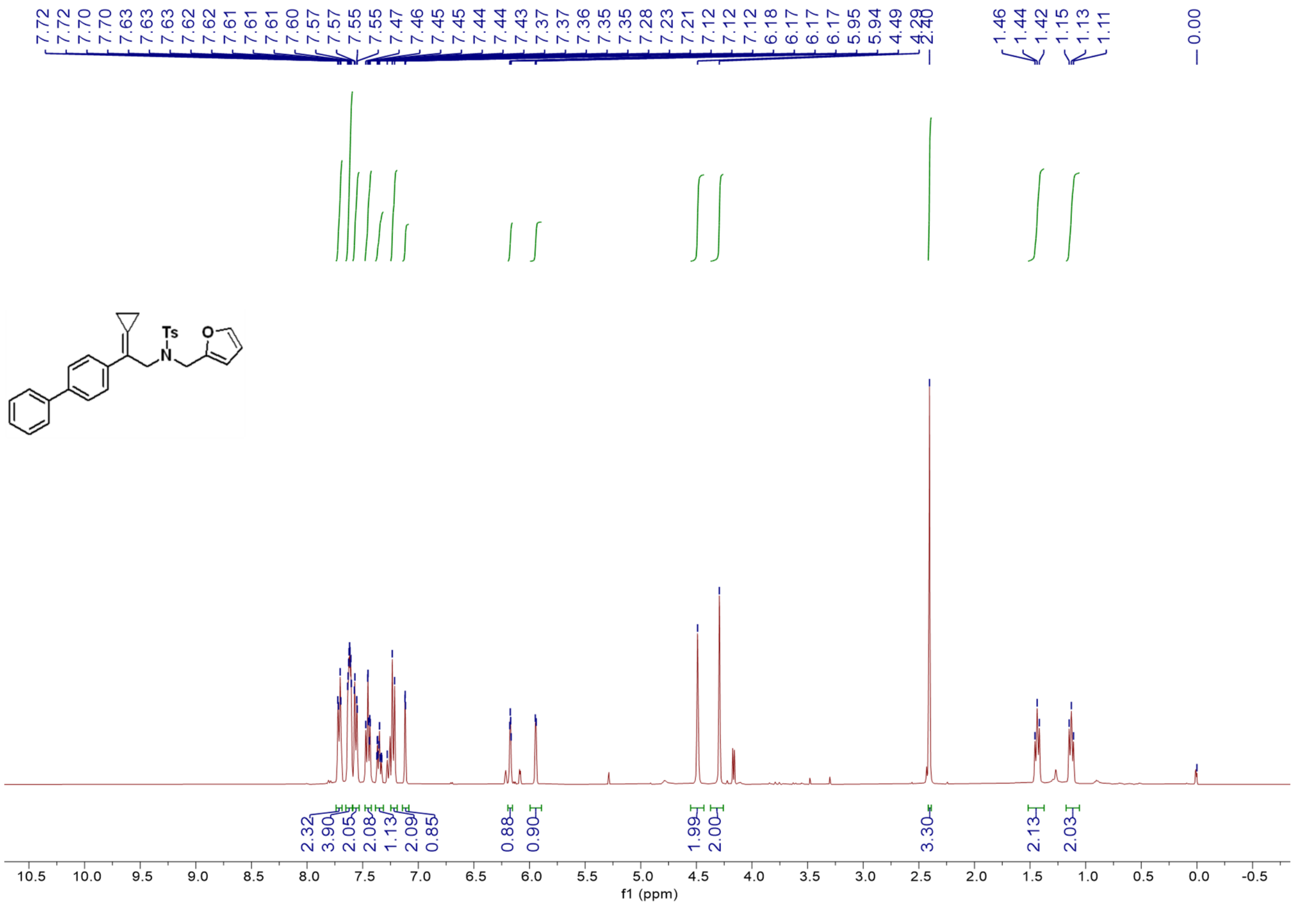
7.72 7.72 7.70 7.70 7.63 7.63 7.63 7.62 7.62 7.61 7.61 7.61 7.60 7.57 7.57 7.55 7.55 7.47 7.46 7.45 7.45 7.44 7.44 7.43 7.37 7.37 7.36 7.35 7.35 7.28 7.23 7.21 7.12 7.12 7.12 6.18 6.17 6.17 6.17 5.95 5.94 4.49 4.29 2.40
1.46 1.44 1.42 1.15 1.13 1.11
0.00
Ts
N
O
2.32 3.90 2.05 2.08 1.13 2.09 0.85 0.88 0.90 1.99 2.00 3.30 2.13 2.03
f1 (ppm)

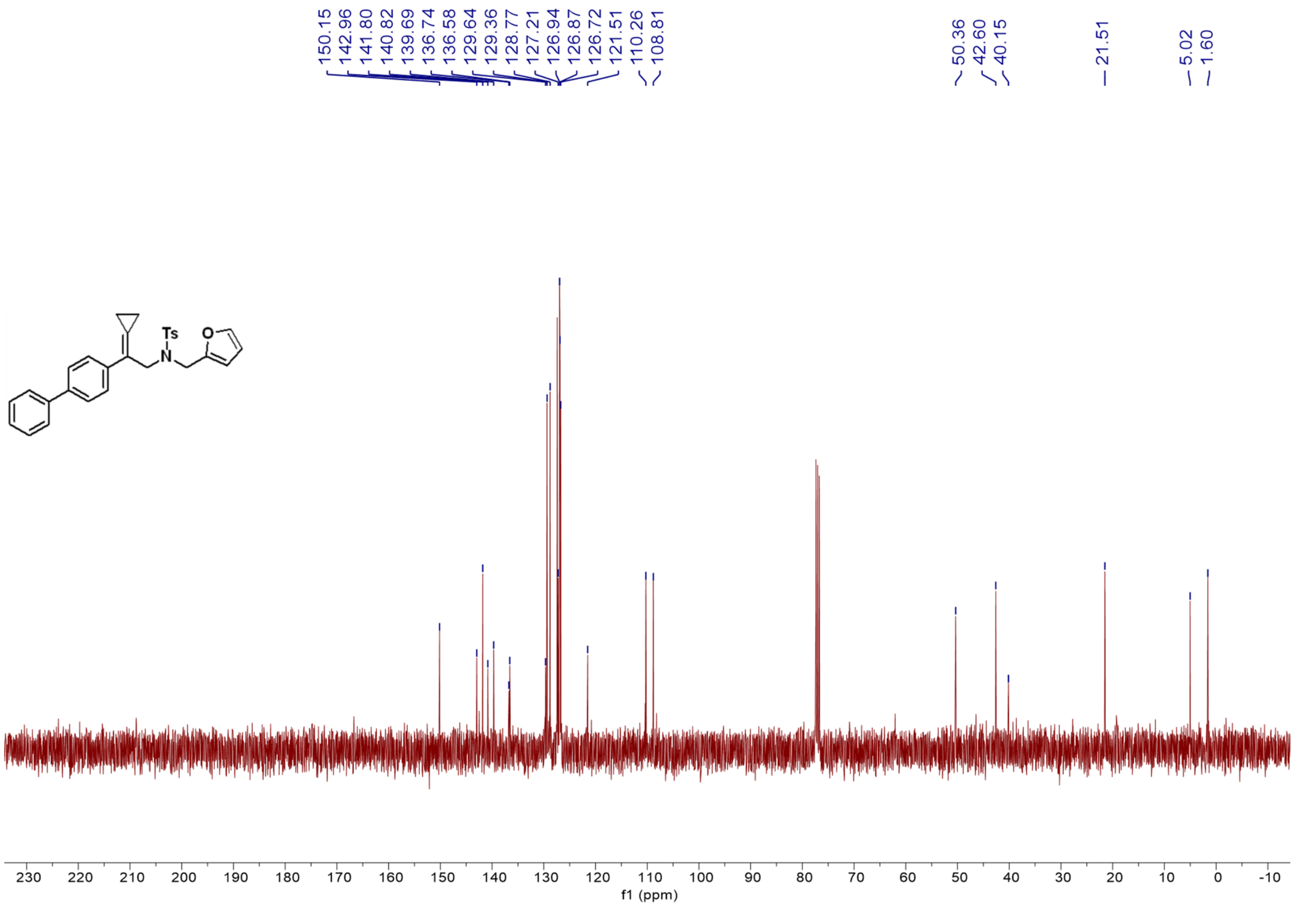
150.15 142.96 141.80 140.82 139.69 136.74 136.58 129.64 129.36 128.77 127.21 126.94 126.87 126.72 121.51 110.26 108.81
50.36 42.60 40.15
21.51
5.02 1.60
Ts
N
O
f1 (ppm)

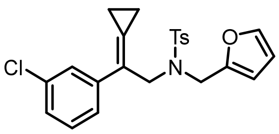

Compound 1k: Yield: 624 mg, 52%; A white solid; M.p.: 83 - 85 ºC; ^{1}H NMR (400 MHz, $CDCl_3$) δ 77.59 (d, J = 8.2 Hz, 2H), 7.52 - 7.45 (m, 2H), 7.25 - 7.18 (m, 4H), 7.14 - 7.09 (m, 1H), 6.20 - 6.15 (m, 1H), 5.94 (d, J = 3.2 Hz, 1H), 4.40 (s, 2H), 4.25 (s, 2H), 2.42 (s, 3H), 1.43 - 1.35 (m, 2H), 1.17 - 1.09 (m, 2H); ^{13}C NMR (100 MHz, $CDCl_3$) δ 142.8, 140.9, 137.9, 136.6, 132.2, 131.2, 129.4, 128.3, 128.0, 127.5, 127.3, 126.8, 126.8, 126.2, 126.1, 125.1, 122.6, 122.2, 52.0, 51.4, 21.4, 4.9, 3.5, 2.5, 1.6; IR (neat): ν 2965, 1594, 1339, 1260, 1160, 1093, 1030, 799, 724 cm^{-1}; HRMS (ESI) Calcd. for $C_{23}H_{22}NO_3SNaCl$ $[M+Na]^+$: 450.0901, Found: 450.0910.

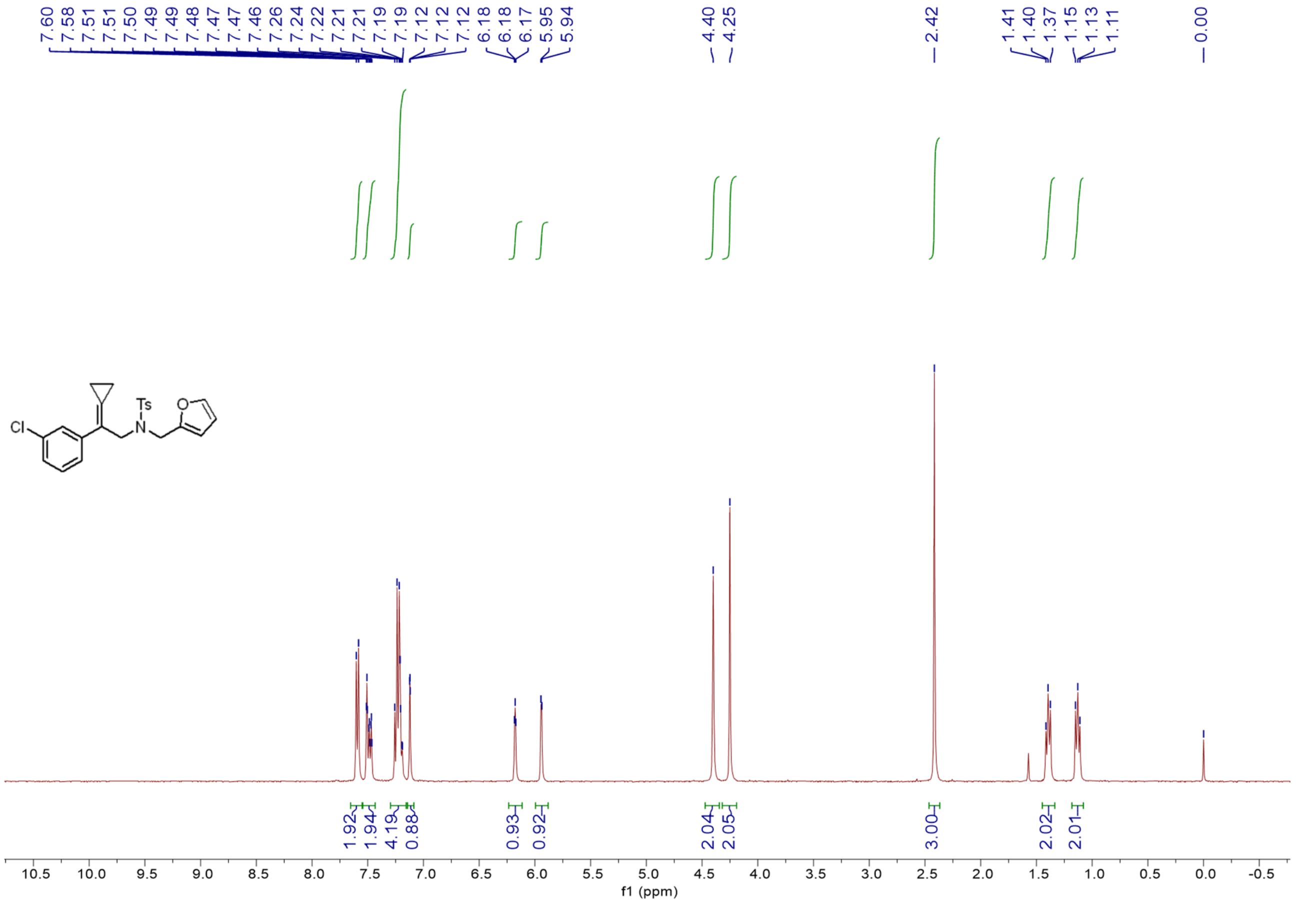
7.60
7.58
7.51
7.51
7.50
7.49
7.49
7.48
7.47
7.47
7.46
7.26
7.24
7.22
7.21
7.21
7.19
7.19
7.12
7.12
7.12
6.18
6.18
6.17
5.95
5.94
4.40
4.25
2.42
1.41
1.40
1.37
1.15
1.13
1.11
0.00
Cl
Ts
N
O
1.92
1.94
4.19
0.88
0.93
0.92
2.04
2.05
3.00
2.02
2.01
f1 (ppm)

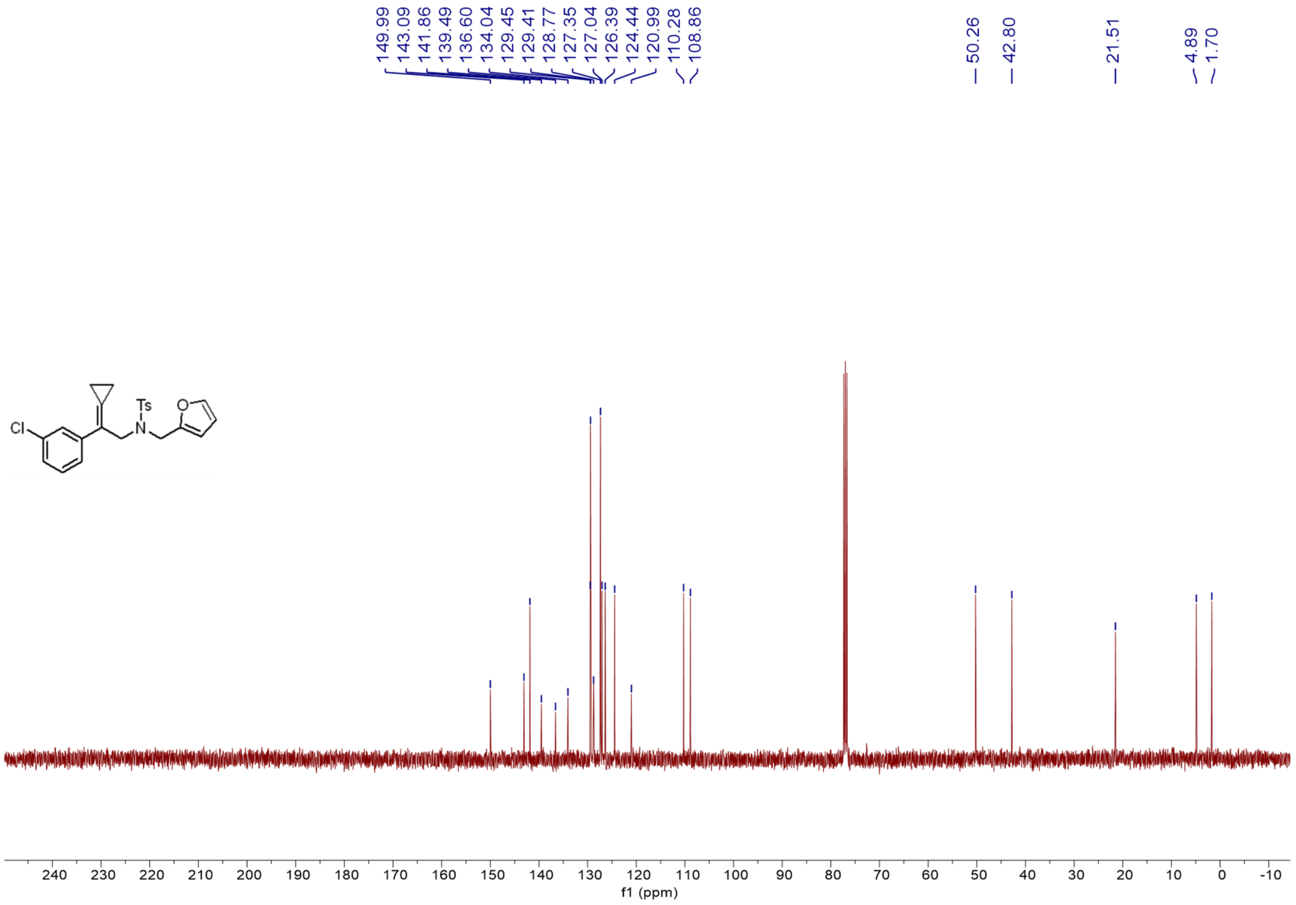
149.99
143.09
141.86
139.49
136.60
134.04
129.45
129.41
128.77
127.35
127.04
126.39
124.44
120.99
110.28
108.86
50.26
42.80
21.51
4.89
1.70
Cl
Ts
N
O
f1 (ppm)

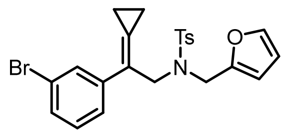

Compound 1l: Yield: 918 mg, 65%; A white solid; M.p.: 84 - 86 ºC; ^{1}H NMR (400 MHz, $CDCl_3$) 7.70 - 7.64 (m, 1H), 7.59 (d, *J* = 8.0 Hz, 2H), 7.52 (d, *J* = 8.0 Hz, 1H), 7.36 (d, *J* = 8.0 Hz, 1H), 7.21 (dt, *J* = 25.0, 8.0 Hz, 3H), 7.12 (dd, *J* = 1.8, 0.8 Hz, 1H), 6.18 (dd, *J* = 3.2, 1.8 Hz, 1H), 5.94 (d, *J* = 3.2 Hz, 1H), 4.40 (s, 2H), 4.25 (s, 2H), 2.42 (s, 3H), 1.44 - 1.35 (m, 2H), 1.18 - 1.08 (m, 2H); ^{13}C NMR (100 MHz, $CDCl_3$) δ 150.0, 143.1, 141.9, 139.8, 136.6, 130.0, 129.8, 129.4, 129.3, 128.8, 127.3, 124.9, 122.3, 120.9, 110.3, 108.9, 50.2, 42.8, 21.5, 4.9, 1.7; IR (neat): ν 2963, 1977, 1595, 1344, 1162, 1093, 891, 813, 715 cm^{-1}; HRMS (ESI) Calcd. for $C_{23}H_{22}NO_3SNaBr$ $[M+Na]^+$: 494.0396, Found: 494.0398.

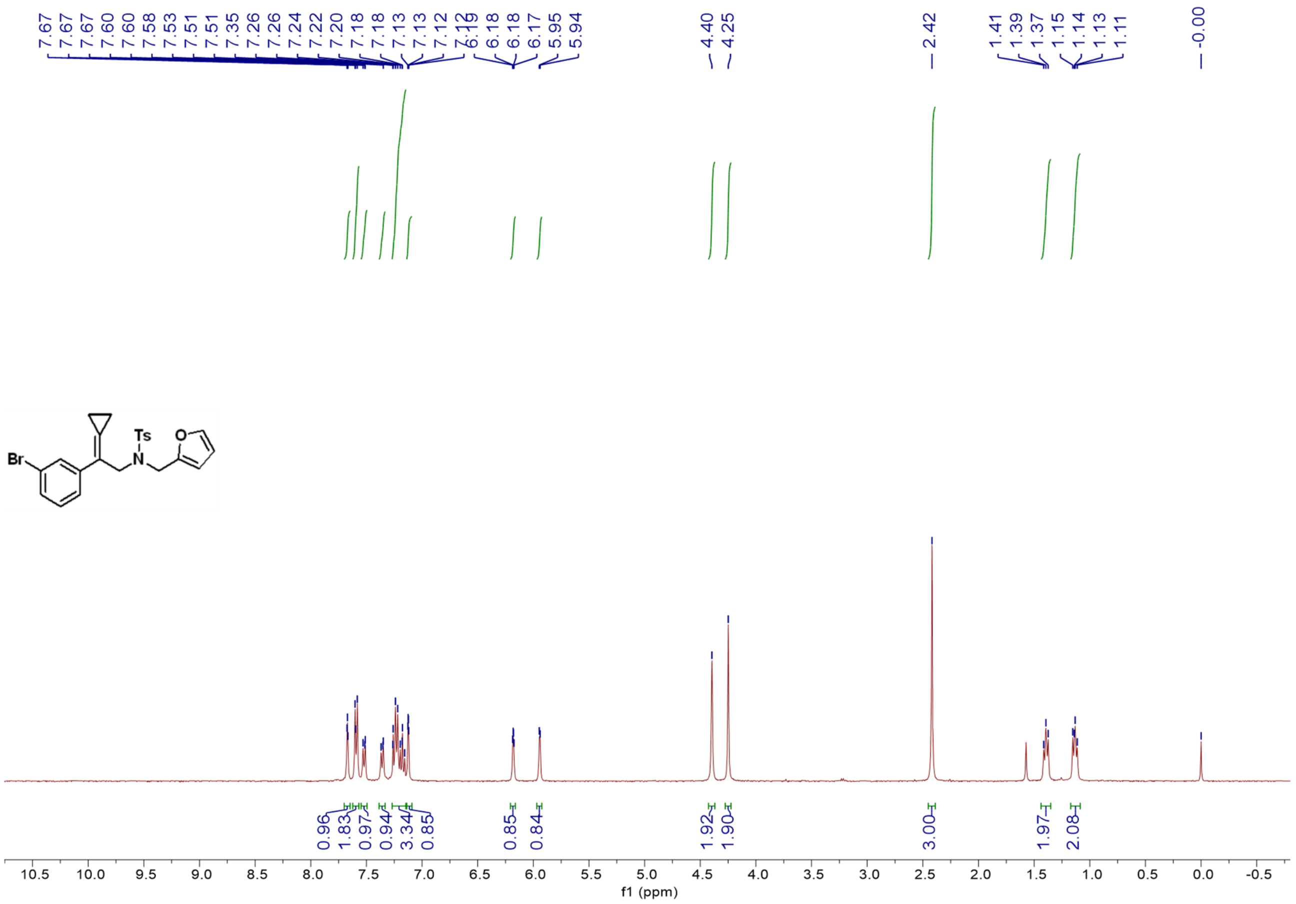
7.67
7.67
7.67
7.60
7.60
7.58
7.53
7.51
7.51
7.35
7.26
7.26
7.24
7.22
7.20
7.18
7.18
7.13
7.13
7.12
7.12
6.19
6.18
6.18
6.17
5.95
5.94
4.40
4.25
2.42
1.41
1.39
1.37
1.15
1.14
1.13
1.11
-0.00
Br
Ts
N
O
0.96
1.83
0.97
0.94
3.34
0.85
0.85
0.84
1.92
1.90
3.00
1.97
2.08
10.5 10.0 9.5 9.0 8.5 8.0 7.5 7.0 6.5 6.0 5.5 5.0 4.5 4.0 3.5 3.0 2.5 2.0 1.5 1.0 0.5 0.0 -0.5
f1 (ppm)

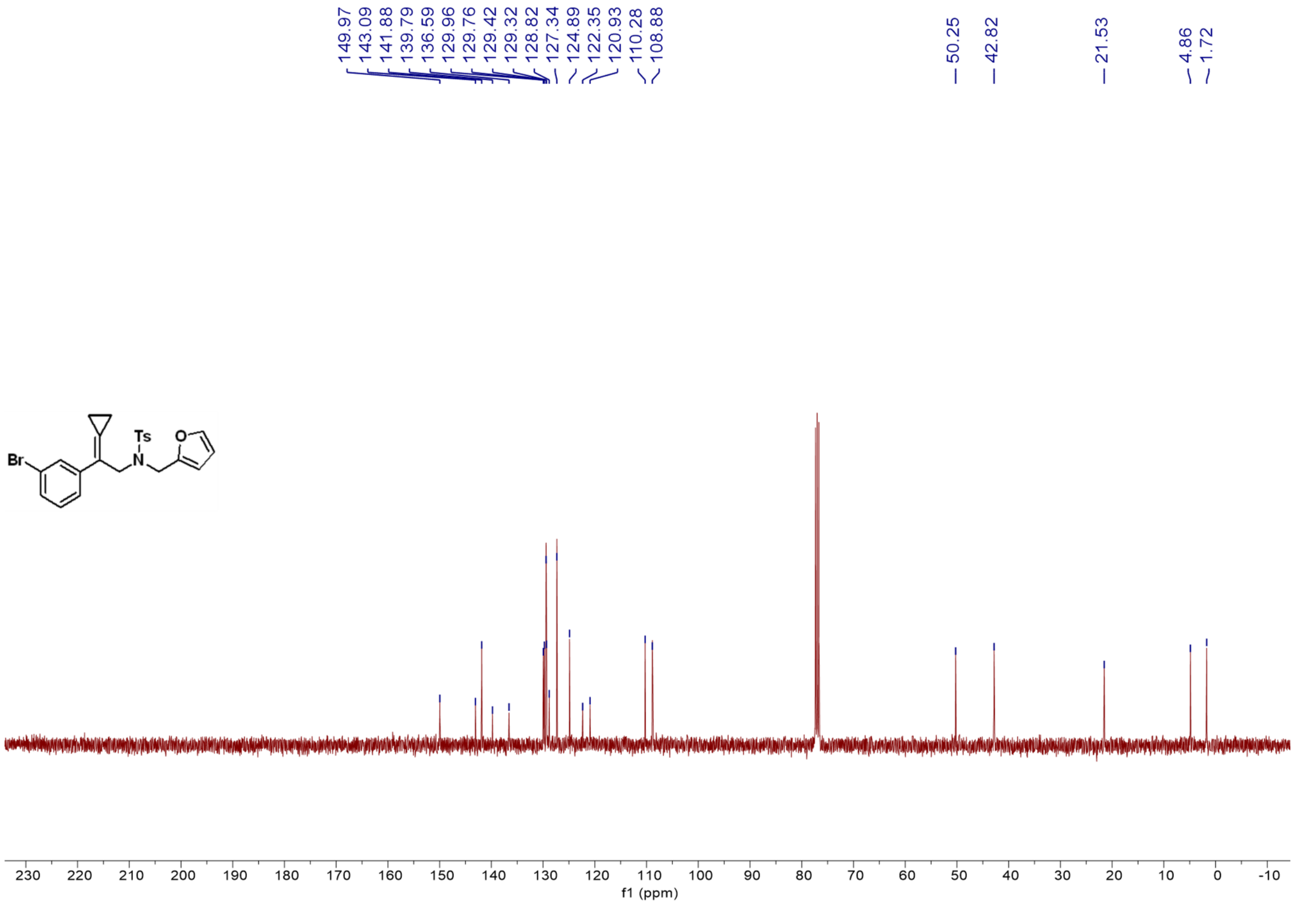
149.97
143.09
141.88
139.79
136.59
129.96
129.76
129.42
129.32
128.82
127.34
124.89
122.35
120.93
110.28
108.88
50.25
42.82
21.53
4.86
1.72
Br
Ts
N
O
230 220 210 200 190 180 170 160 150 140 130 120 110 100 90 80 70 60 50 40 30 20 10 0 -10
f1 (ppm)

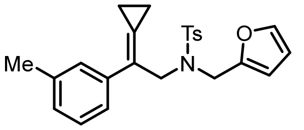

Compound 1m: Yield: 787 mg, 64%; A white solid; M.p.: 82 - 84 °C; ^{1}H NMR (400 MHz, $CDCl_3$) δ 7.59 (d, *J* = 8.0 Hz, 2H), 7.38 (d, *J* = 6.0 Hz,2H), 7.20 (t, *J* = 8.0 Hz, 3H), 7.12 (d, *J* = 1.8 Hz, 1H), 7.06 (d, *J* = 7.4 Hz, 1H), 6.17 (dd, *J* = 3.2, 1.8 Hz,1H), 4.43 (s, 2H), 4.26 (s, 2H), 2.41 (s, 3H), 2.34 (s, 3H), 1.37 (t, *J* = 7.8 Hz, 2H), 1.09 (t, *J* = 7.8 Hz, 2H); ^{13}C NMR (100 MHz, $CDCl_3$) δ 150.2, 142.8, 141.8, 137.6, 136.9, 129.3, 128.1, 127.9, 127.4, 127.04, 126.96, 123.5, 121.9, 110.2, 108.8, 50.2, 42.5, 21.6, 21.5, 4.8, 1.6; IR (neat): ν 2924, 1776, 1599, 1493, 1335, 1158, 1090, 1011, 814, 741 cm^{-1}; HRMS (ESI) Calcd. for $C_{24}H_{25}NO_3NaS$ $[M+Na]^+$ 430.1447, Found: 430.1450.

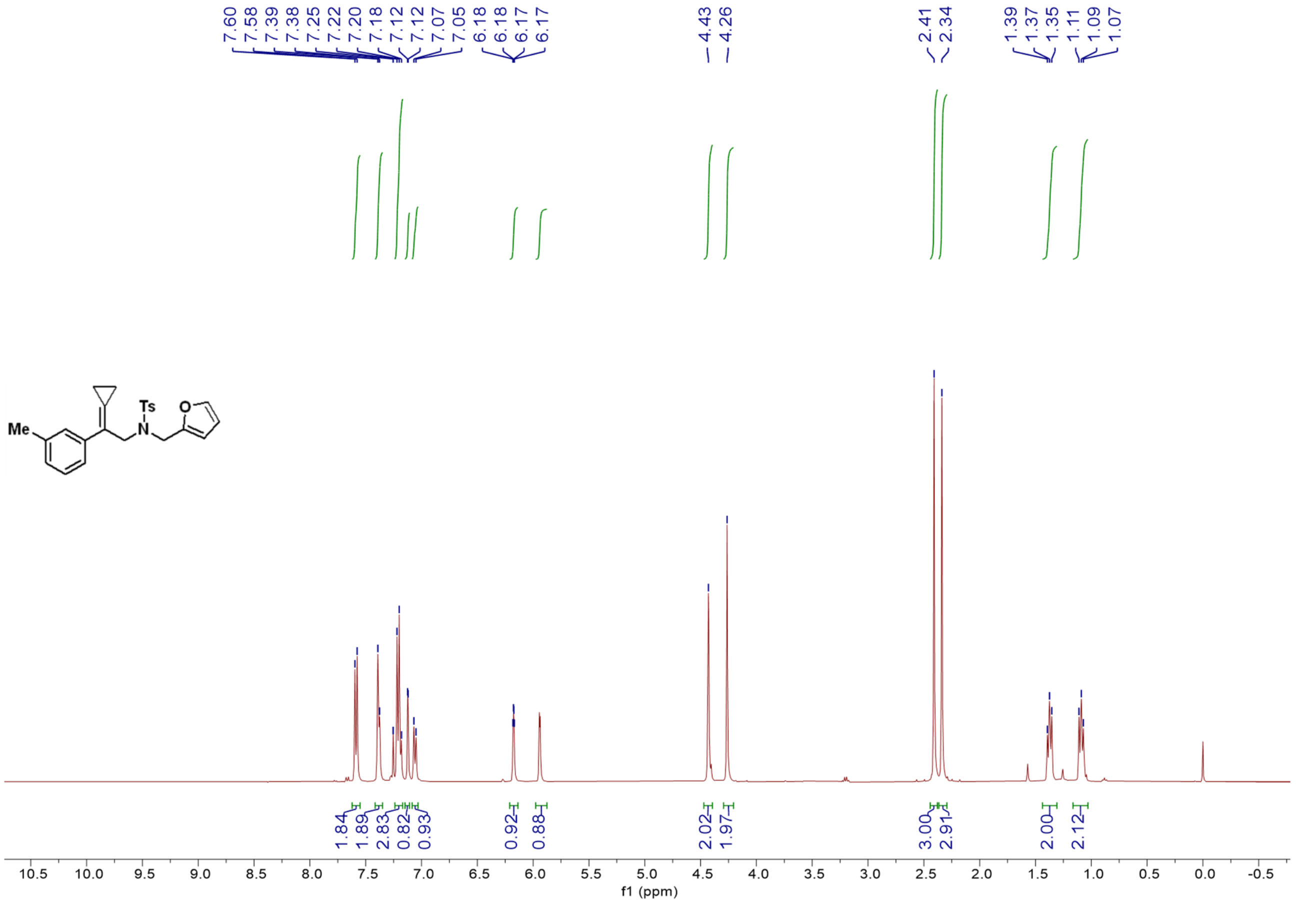
7.60 7.58 7.39 7.38 7.25 7.22 7.20 7.18 7.12 7.12 7.07 7.05 6.18 6.18 6.17 6.17
4.43 4.26
2.41 2.34
1.39 1.37 1.35 1.11 1.09 1.07
Me
Ts
N
O
1.84 1.89 2.83 0.82 0.93 0.92 0.88 2.02 1.97 3.00 2.91 2.00 2.12
f1 (ppm)

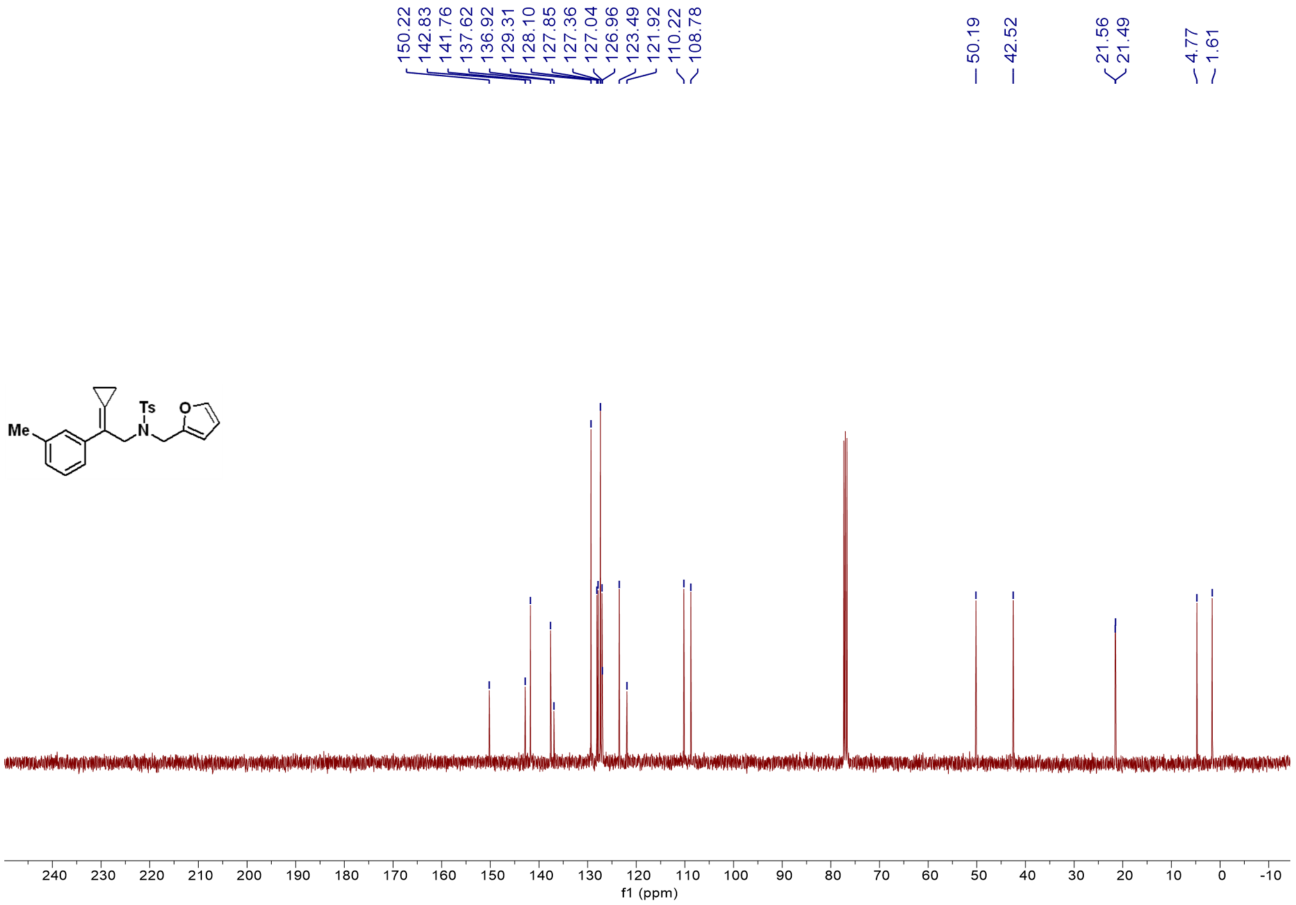
150.22 142.83 141.76 137.62 136.92 129.31 128.10 127.85 127.36 127.04 126.96 123.49 121.92 110.22 108.78
50.19
42.52
21.56 21.49
4.77 1.61
Me
Ts
N
O
f1 (ppm)

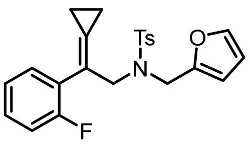

Compound 1n: Yield: 708 mg, 52%; A white solid; M.p.: 86 - 90 °C; ^{1}H NMR (400 MHz, $CDCl_3$) δ 7.52 (d, *J* = 8.2 Hz, 2H), 7.25 - 7.13 (m, 5H), 7.06 (td, *J* = 7.4, 1.2 Hz, 1H), 7.02 - 6.94 (m, 1H), 6.22 (dd, *J* = 3.2, 1.8 Hz, 1H), 6.04 (d, *J* = 3.2 Hz, 1H), 4.38 (s, 2H), 4.32 (s, 2H), 2.39 (s, 3H), 1.28 - 1.14 (m, 4H). ^{13}C NMR (100 MHz, $CDCl_3$) δ 160.2 (d, *J* = 247.4 Hz), 150.0, 142.8, 142.1, 137.2, 130.23 (d, *J* = 4.2 Hz), 130.15, 129.3, 128.7 (d, *J* = 8.3 Hz), 127.2, 126.5 (d, *J* = 14.5 Hz), 123.9 (d, *J* = 3.5 Hz), 119.0, 115.7 (d, *J* = 22.8 Hz), 110.3, 109.1, 50.6 (d, *J* = 3.1 Hz), 42.7, 21.5, 3.7 (d, *J* = 3.1 Hz), 3.4; ^{19}F NMR (376 MHz, $CDCl_3$) δ -114.26; IR (neat): ν 2980, 1598, 1489, 1450, 1334, 1158, 1092, 1010, 920, 814, 756 cm^{-1}; HRMS (ESI) Calcd. for $C_{23}H_{22}NO_3SNaF$ $[M+Na]^+$: 434.1197, Found: 434.1207.

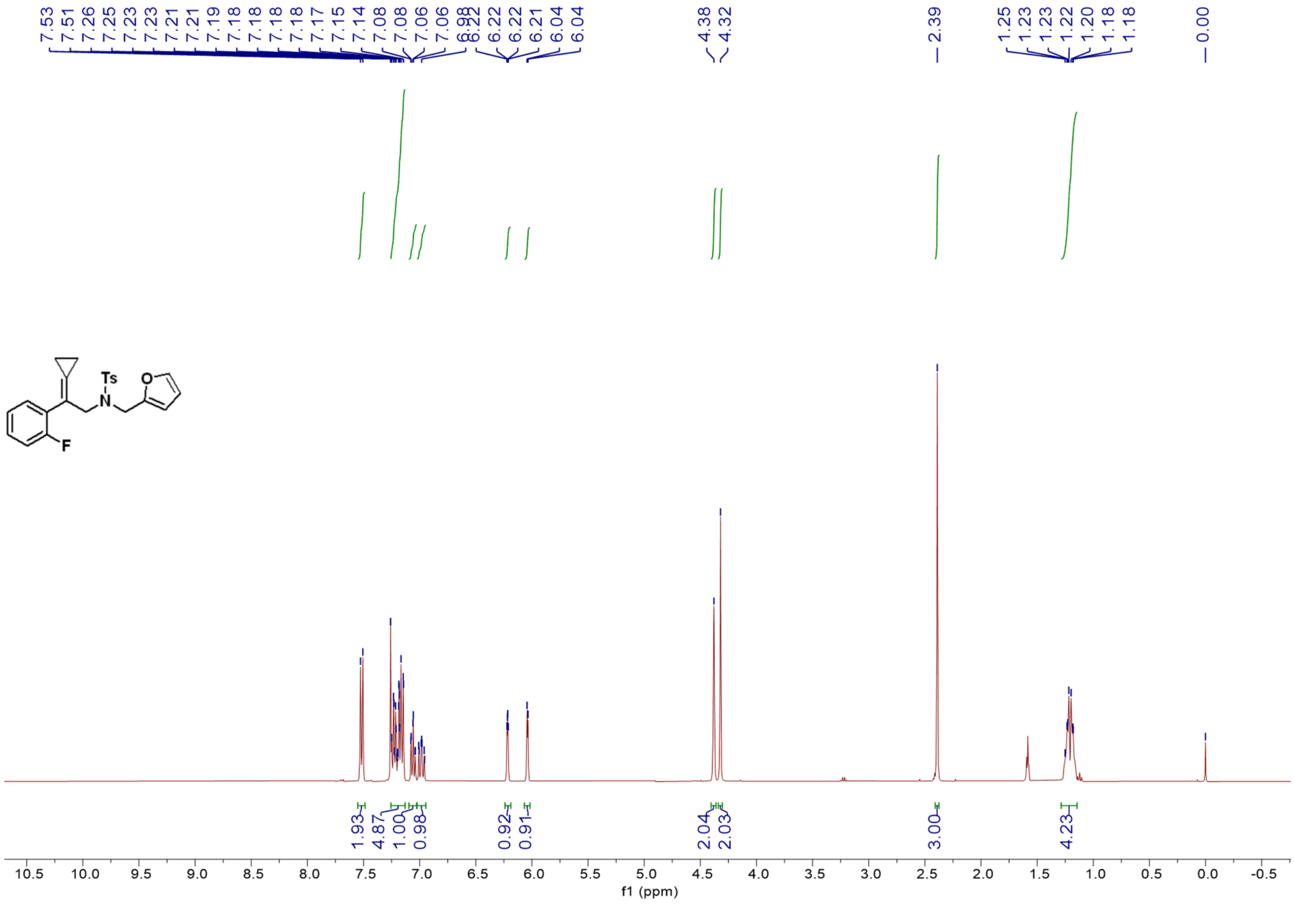
7.53 7.51 7.26 7.25 7.23 7.23 7.21 7.21 7.19 7.18 7.18 7.18 7.18 7.17 7.15 7.14 7.08 7.06 7.06 6.98 6.22 6.22 6.22 6.21 6.04 6.04
4.38 4.32
2.39
1.25 1.23 1.23 1.22 1.20 1.18 1.18
0.00
Ts
N
O
F
1.93 4.87 1.00 0.98 0.92 0.91 2.04 2.03 3.00 4.23
10.5 10.0 9.5 9.0 8.5 8.0 7.5 7.0 6.5 6.0 5.5 5.0 4.5 4.0 3.5 3.0 2.5 2.0 1.5 1.0 0.5 0.0 -0.5
f1 (ppm)

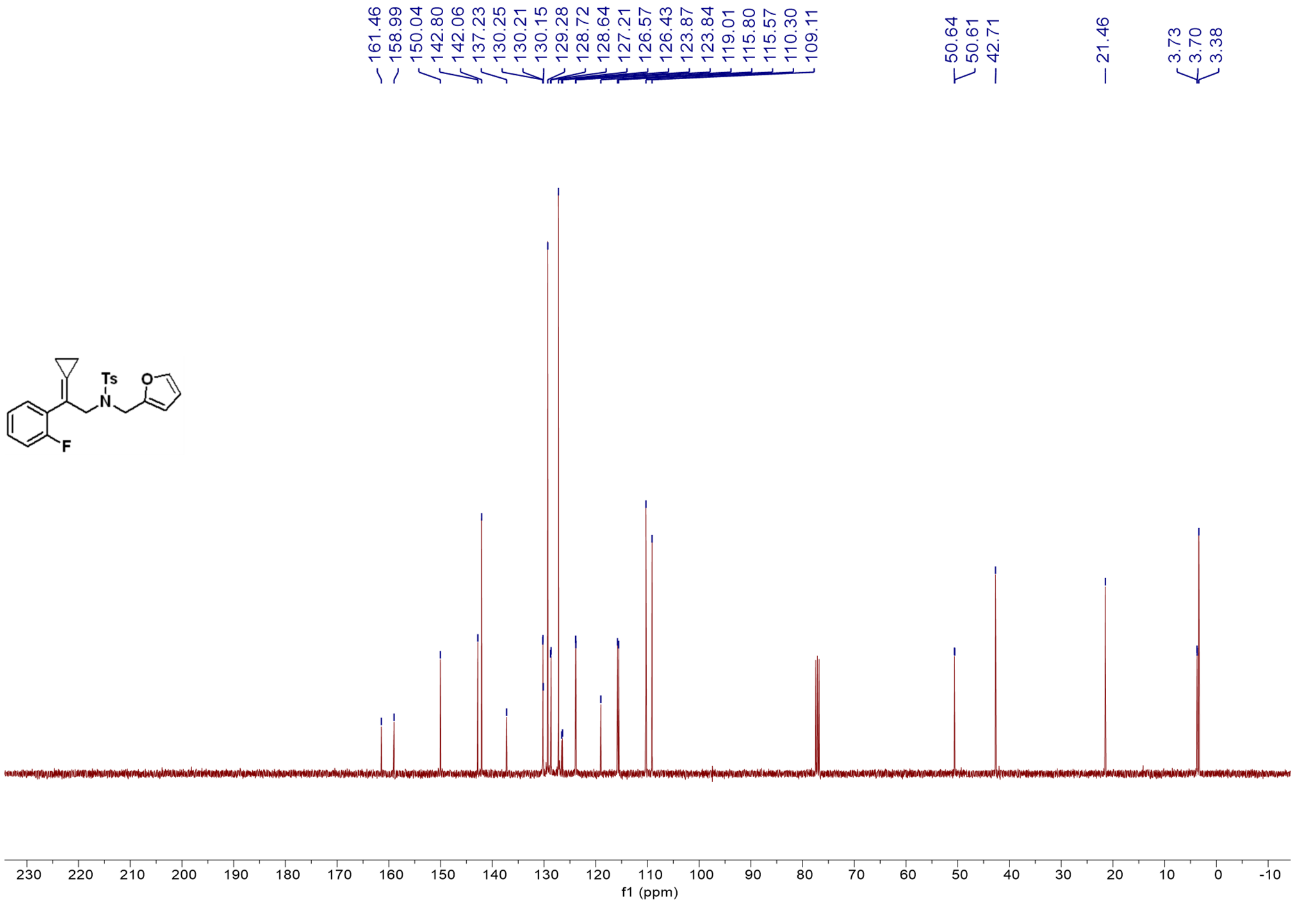
161.46 158.99 150.04 142.80 142.06 137.23 130.25 130.21 130.15 129.28 128.72 128.64 127.21 126.57 126.43 123.87 123.84 119.01 115.80 115.57 110.30 109.11
50.64 50.61 42.71
21.46
3.73 3.70 3.38
Ts
N
O
F
230 220 210 200 190 180 170 160 150 140 130 120 110 100 90 80 70 60 50 40 30 20 10 0 -10
f1 (ppm)

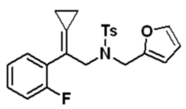

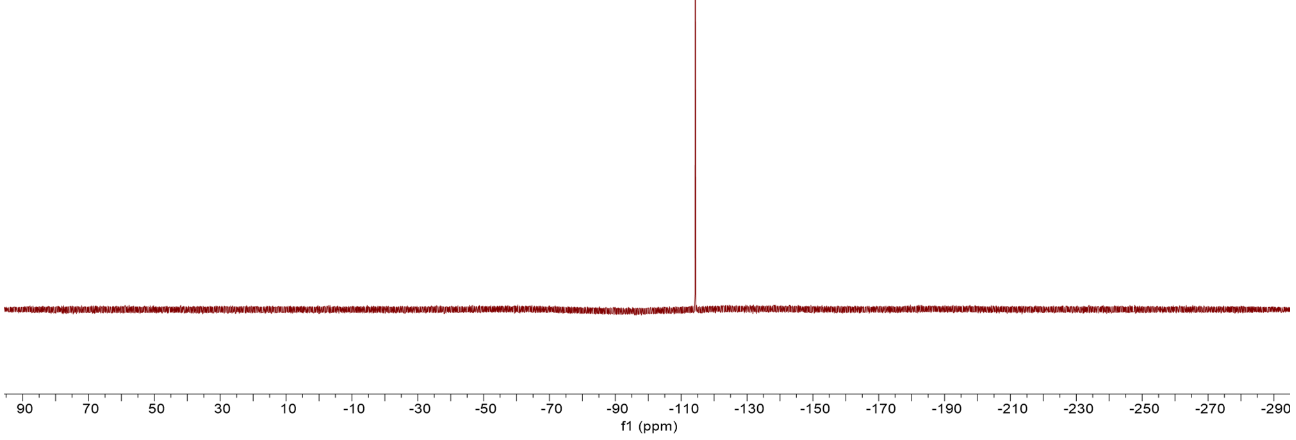
-114.26
90
70
50
30
10
-10
-30
-50
-70
-90
-110
-130
-150
-170
-190
-210
-230
-250
-270
-290
f1 (ppm)

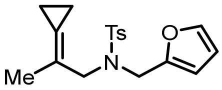

Compound 1m: Yield: 298 mg, 15%; A colorless oil; ^{1}H NMR (400 MHz, $CDCl_3$) δ 7.62 (d, *J* = 8.2 Hz, 2H), 7.23 (d, *J* = 8.2 Hz, 2H), 7.19 – 7.14 (m, 1H), 6.20 (dd, *J* = 3.2, 1.8 Hz, 1H), 6.02 (d, *J* = 3.2 Hz, 1H), 4.31 (s, 2H), 3.89 (s, 2H), 2.40 (s, 4H), 1.76 (t, *J* = 1.6 Hz, 3H), 1.00 (q, *J* = 1.6 Hz, 4H); ^{13}C NMR (100 MHz, $CDCl_3$) δ 150.1, 142.8, 142.0, 137.4, 129.3, 127.2, 121.8, 119.2, 110.2, 108.9, 52.6, 42.6, 21.4, 18.1, 2.7, 2.0; IR (neat): ν 2920, 1597, 1335, 1155, 1092, 1057, 893, 812, 742, 677 cm^{-1}; HRMS (ESI) Calcd. for $C_{18}H_{22}NO_3S$ $[M+H]^+$: 332.1315, Found: 332.1318.

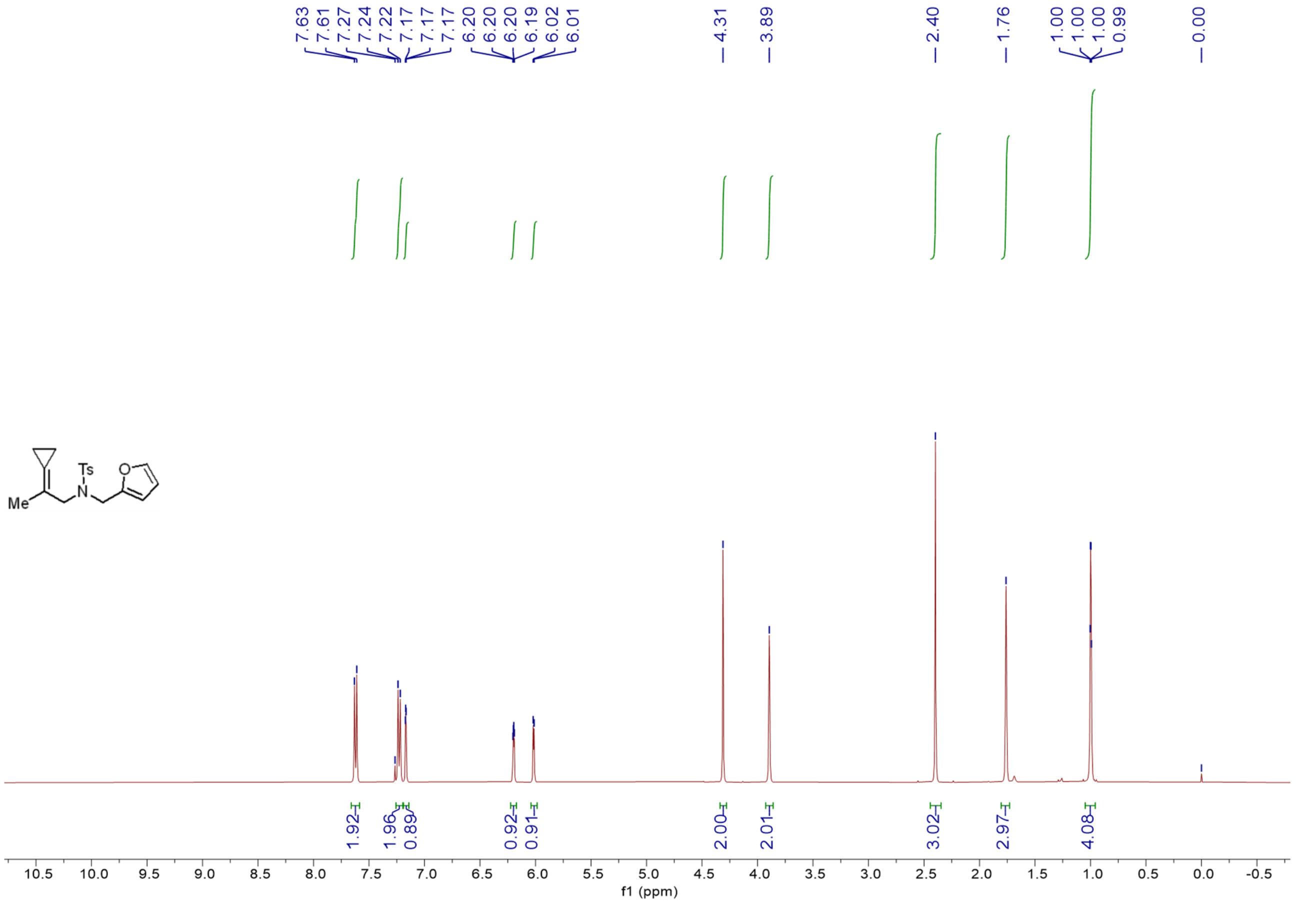
7.63
7.61
7.27
7.24
7.22
7.17
7.17
7.17
6.20
6.20
6.20
6.19
6.02
6.01
4.31
3.89
2.40
1.76
1.00
1.00
1.00
0.99
0.00
Ts
N
O
Me
1.92
1.96
0.89
0.92
0.91
2.00
2.01
3.02
2.97
4.08
f1 (ppm)

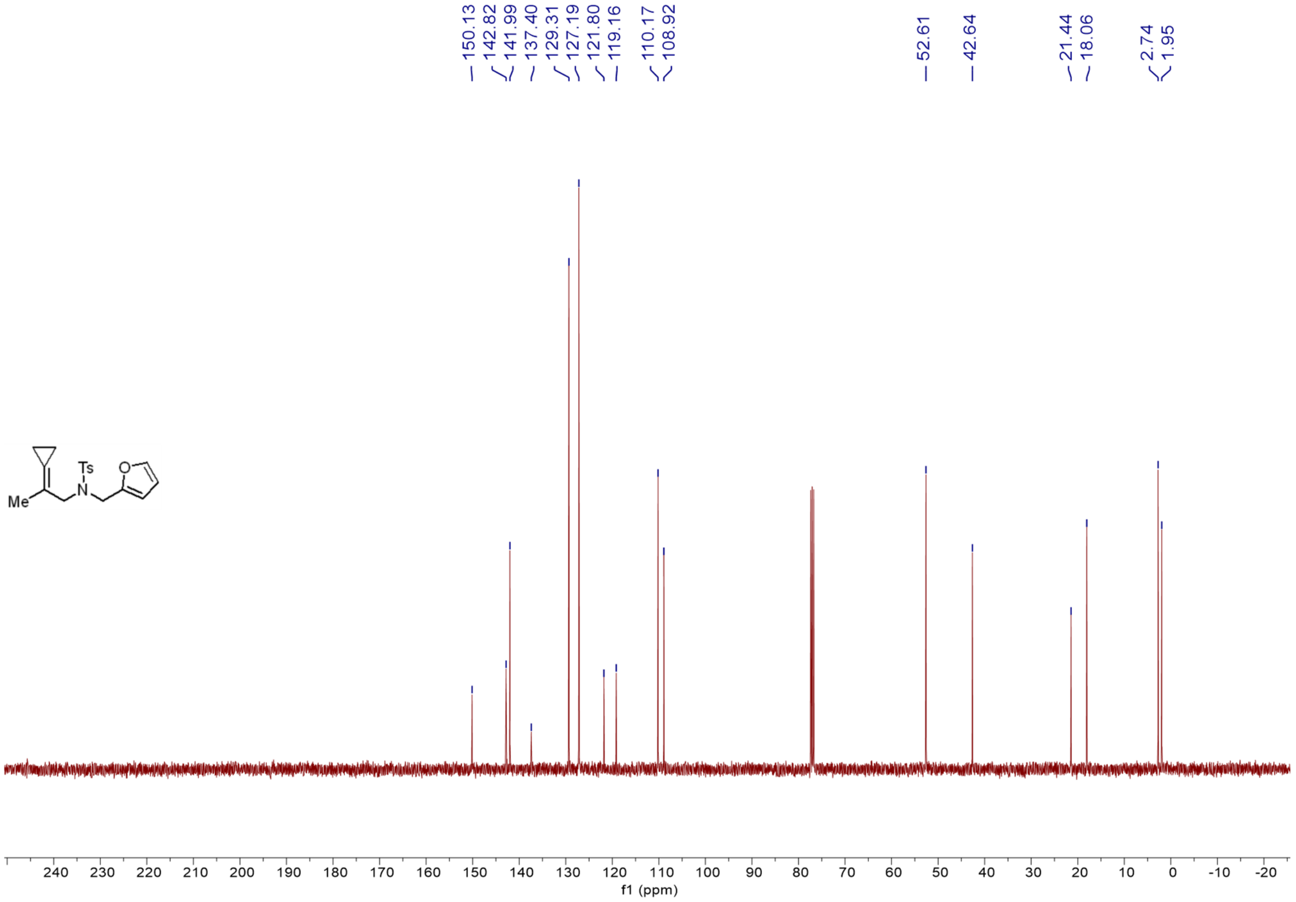
150.13
142.82
141.99
137.40
129.31
127.19
121.80
119.16
110.17
108.92
52.61
42.64
21.44
18.06
2.74
1.95
Ts
N
O
Me
f1 (ppm)

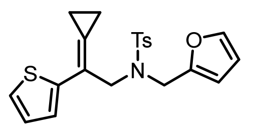

Compound 1p: Yield: 692 mg, 58%; A white solid; M.p.: 110 - 112 ºC; ^{1}H NMR (400 MHz, $CDCl_3$) δ 7.61 (d, *J* = 8.0 Hz, 2H), 7.36 (d, *J* = 3.6 Hz, 1H), 7.23 (d, *J* = 8.0 Hz, 2H), 7.17 (d, *J* = 5.2 Hz, 1H), 7.11 - 7.08 (m, 1H), 7.00 (dd, *J* = 5.2, 3.6 Hz, 1H), 6.15 (t, *J* = 2.5 Hz, 1H), 5.92 (d, *J* = 3.2 Hz, 1H), 4.39 (s, 2H), 4.33 (s, 2H), 2.41 (s, 3H), 1.35 (dd, *J* = 9.2, 6.0 Hz, 2H), 1.22 (dd, *J* = 9.2, 6.0 Hz, 2H); ^{13}C NMR (100 MHz, $CDCl_3$) δ 150.0, 143.0, 142.8, 141.8, 136.7, 129.4, 127.4, 127.3, 125.9, 124.3, 123.8, 117.8, 110.2, 108.8, 50.3, 42.7, 21.5, 5.3, 3.3; IR (neat): ν 2962, 1780, 1343, 1260, 1159, 1089, 1021, 799, 654 cm^{-1}; HRMS (ESI) Calcd. for $C_{21}H_{21}NO_3S_2Na$ $[M+Na]^+$: 422.0855, Found: 422.0860.

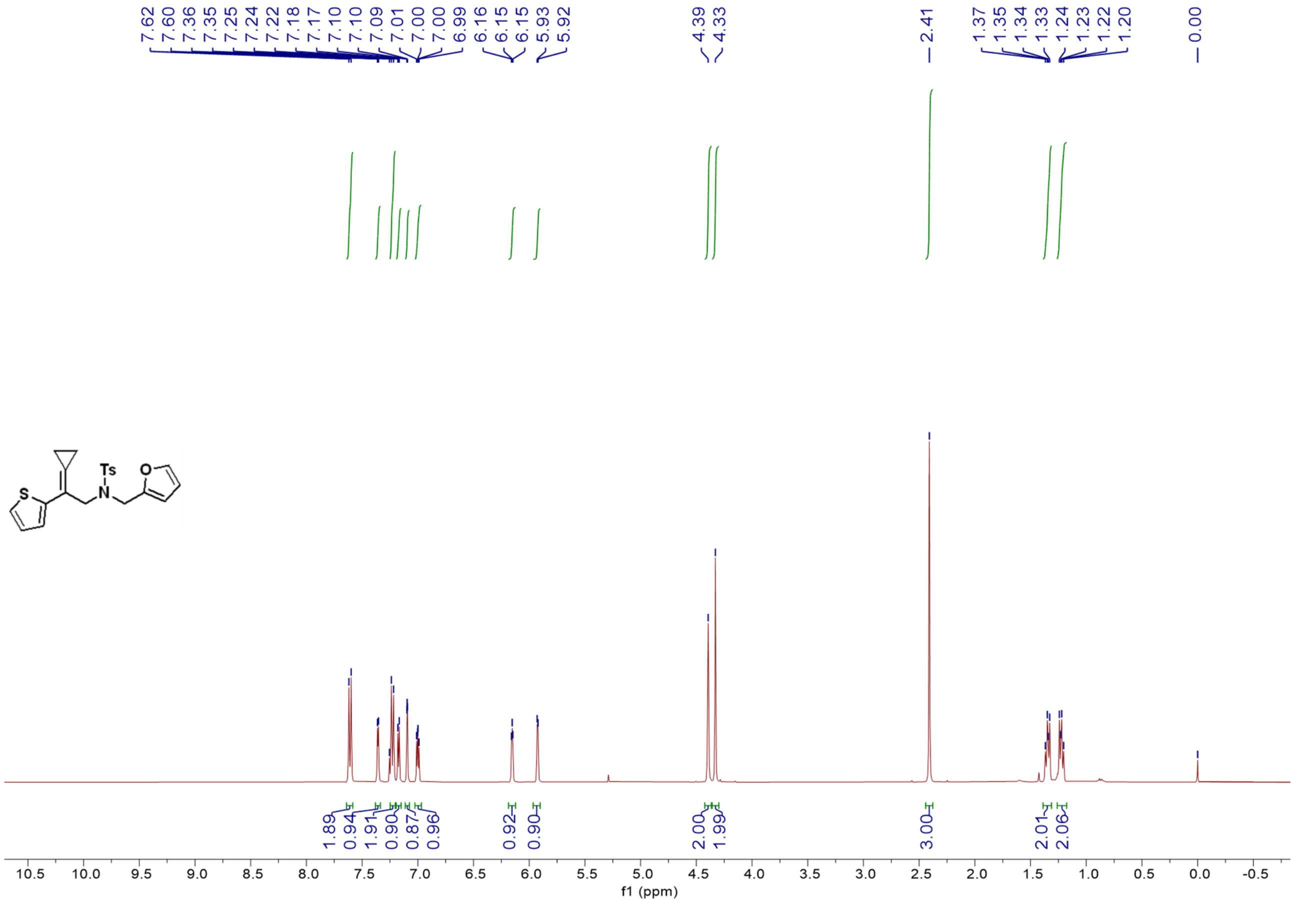
7.62 7.60 7.36 7.35 7.25 7.24 7.22 7.18 7.17 7.10 7.10 7.09 7.01 7.00 7.00 6.99 6.16 6.15 6.15 5.93 5.92
4.39 4.33
2.41
1.37 1.35 1.34 1.33 1.24 1.23 1.22 1.20
0.00
Ts
N
S
O
1.89 0.94 1.91 0.90 0.87 0.96
0.92 0.90
2.00 1.99
3.00
2.01 2.06
10.5 10.0 9.5 9.0 8.5 8.0 7.5 7.0 6.5 6.0 5.5 5.0 4.5 4.0 3.5 3.0 2.5 2.0 1.5 1.0 0.5 0.0 -0.5
f1 (ppm)

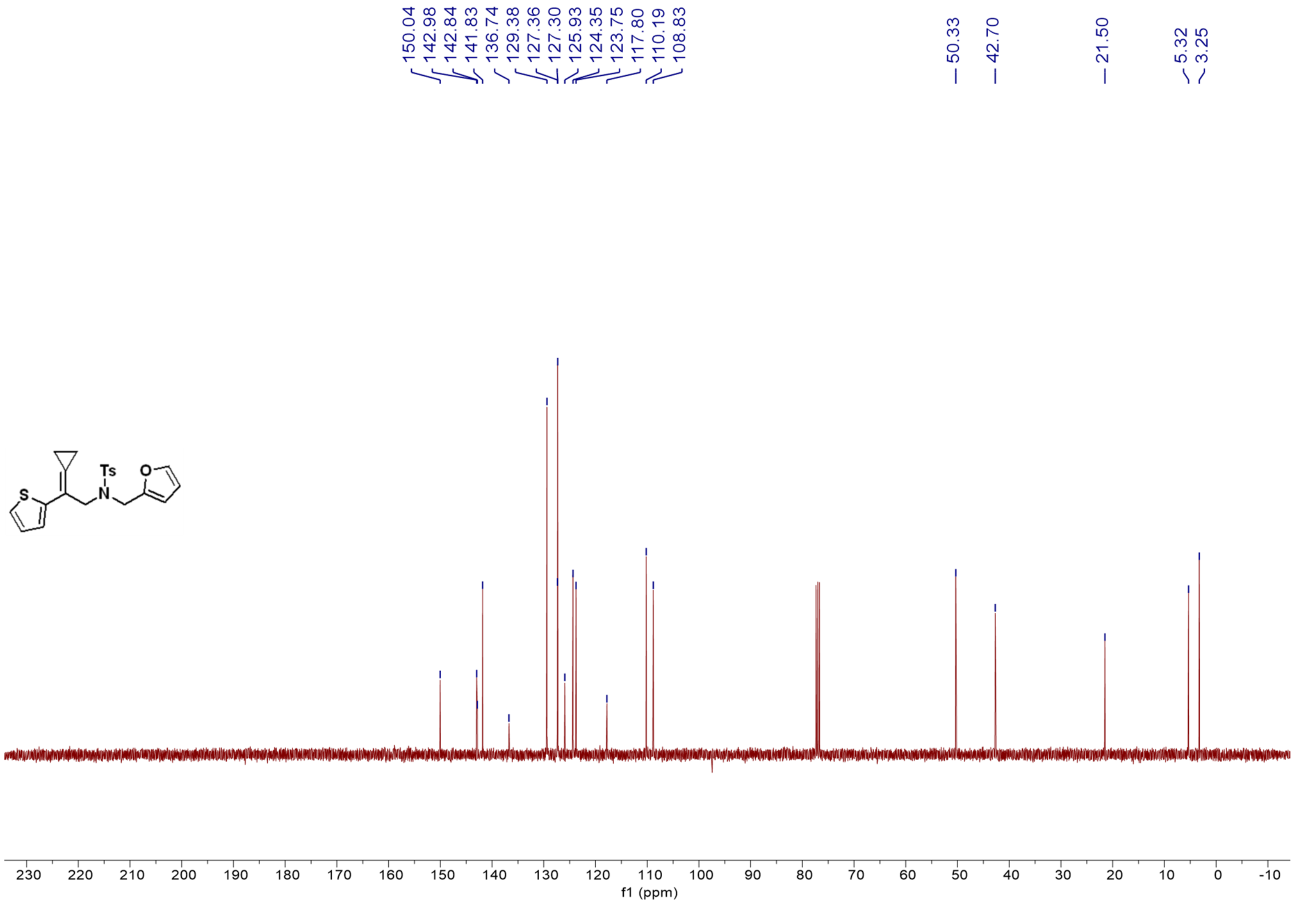
150.04 142.98 142.84 141.83 136.74 129.38 127.36 127.30 125.93 124.35 123.75 117.80 110.19 108.83
50.33
42.70
21.50
5.32 3.25
Ts
N
S
O
230 220 210 200 190 180 170 160 150 140 130 120 110 100 90 80 70 60 50 40 30 20 10 0 -10
f1 (ppm)

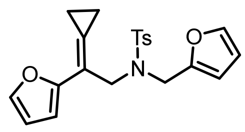

Compound 1q: Yield: 324 mg, 22%; A white solid; M.p.: 97 - 99 ºC; ^{1}H NMR (400 MHz, $CDCl_3$) δ 7.60 (d, *J* = 8.2 Hz, 2H), 7.34 (d, *J* = 1.8 Hz, 1H), 7.22 (d, *J* = 8.2 Hz, 2H), 7.12 (d, *J* = 1.8 Hz, 1H), 6.62 (d, *J* = 3.4 Hz, 1H), 6.39 (dd, *J* = 3.4, 1.8 Hz, 1H), 6.17 (dd, *J* = 3.2, 1.8 Hz, 1H), 5.96 (d, *J* = 3.2 Hz, 1H), 4.35 (s, 2H), 4.31 (s, 2H), 2.41 (s, 2H), 1.40 - 1.32 (m, 2H), 1.20 - 1.13 (m, 2H); ^{13}C NMR (100 MHz, $CDCl_3$) δ 152.8, 150.1, 142.9, 141.9, 141.5, 137.1, 129.3, 127.2, 125.5, 114.3, 111.2, 110.2, 108.9, 106.5, 48.9, 42.7, 21.5, 4.4, 2.0; IR (neat): ν 2962, 2152, 1787, 1338, 1160, 1011, 817, 745, 669 cm^{-1}; HRMS (ESI) Calcd. for $C_{21}H_{21}NO_4SNa$ $[M+Na]^+$: 406.1084, Found: 406.1091.

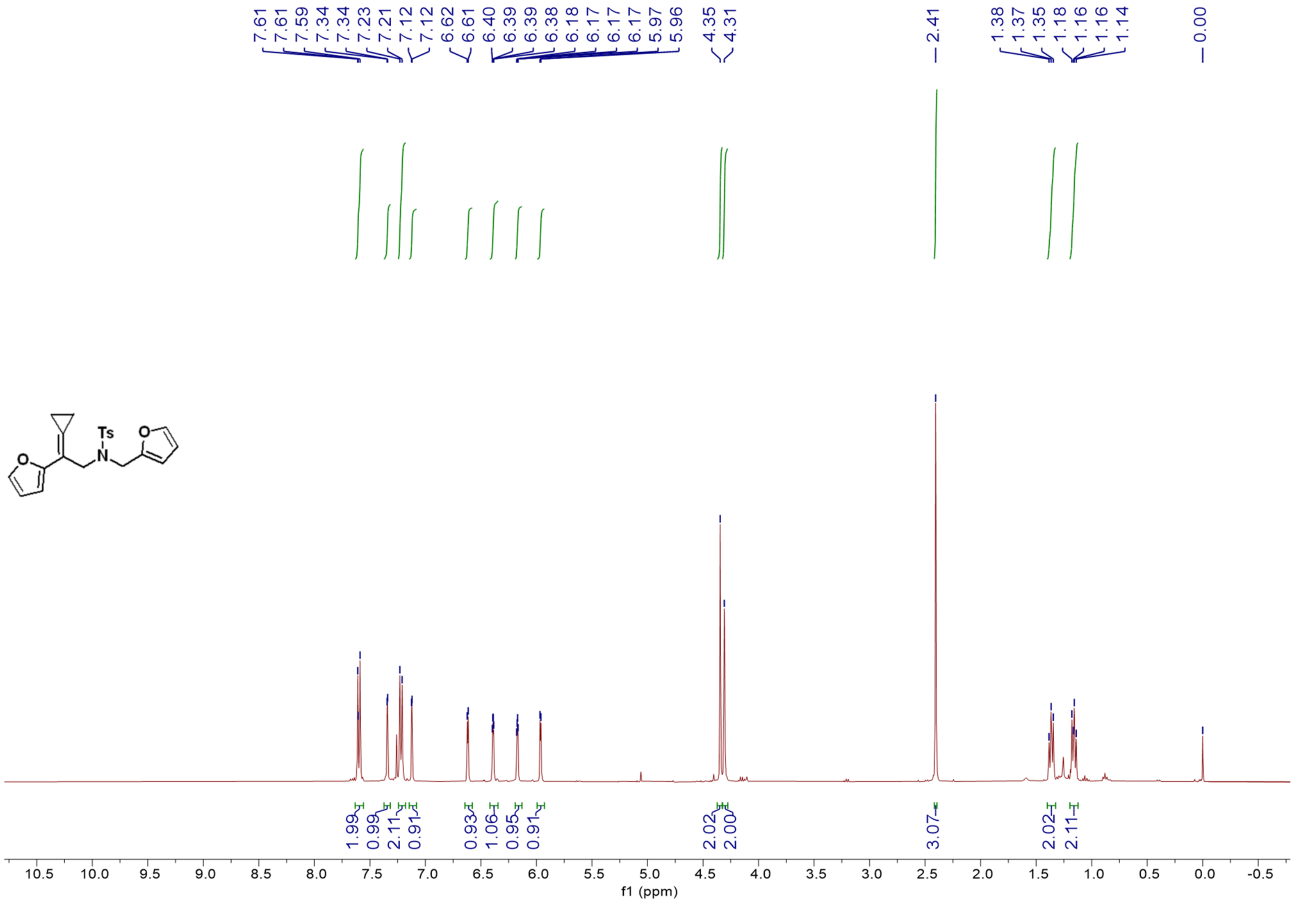

7.61 7.61 7.59 7.34 7.34 7.23 7.21 7.12 7.12 6.62 6.61 6.40 6.39 6.39 6.38 6.18 6.17 6.17 6.17 5.97 5.96 4.35 4.31
2.41
1.38 1.37 1.35 1.18 1.16 1.14
0.00
Ts
N
O
O
1.99 0.99 2.11 0.91 0.93 1.06 0.95 0.91 2.02 2.00 3.07 2.02 2.11
10.5 10.0 9.5 9.0 8.5 8.0 7.5 7.0 6.5 6.0 5.5 5.0 4.5 4.0 3.5 3.0 2.5 2.0 1.5 1.0 0.5 0.0 -0.5
f1 (ppm)

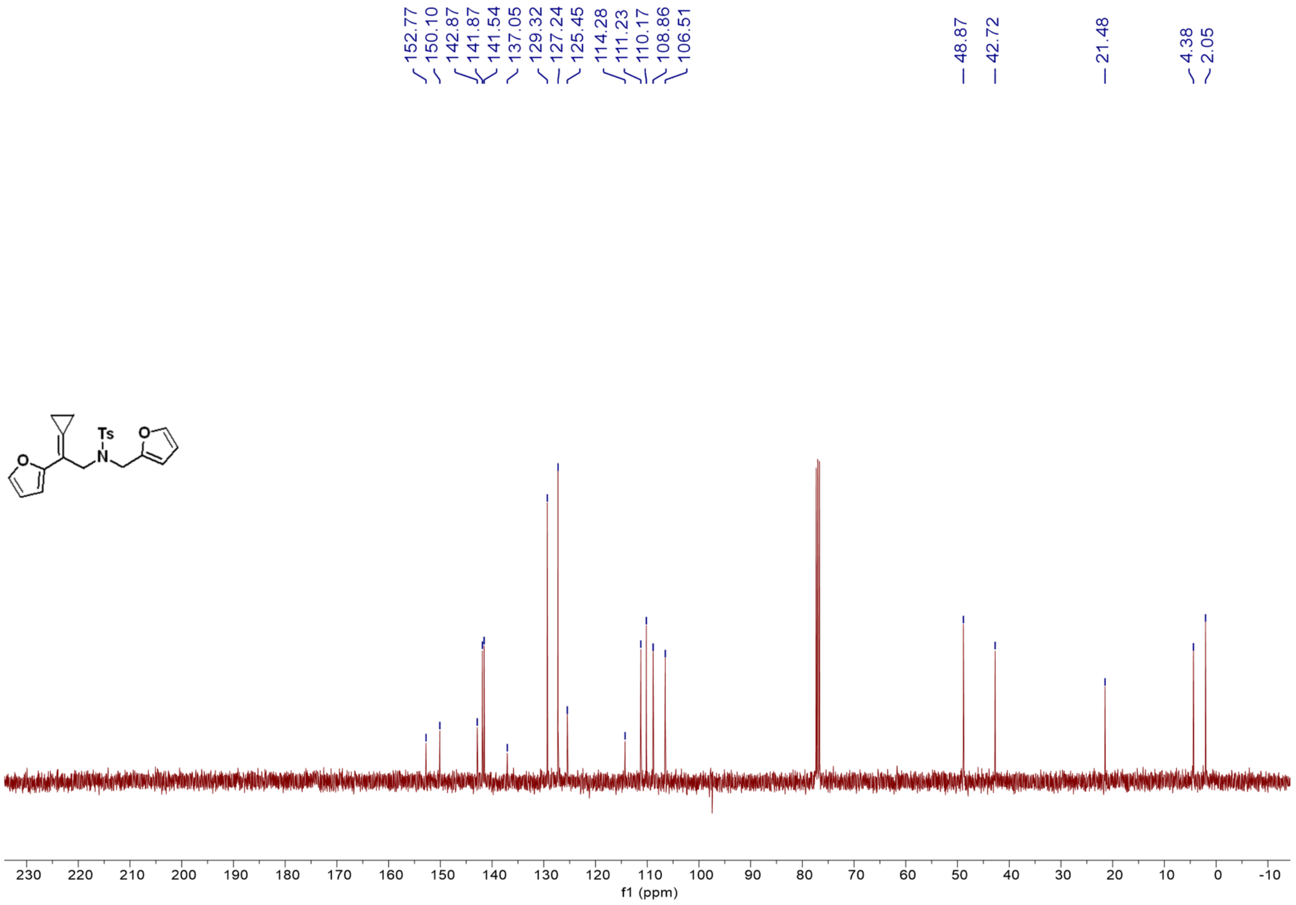

152.77 150.10 142.87 141.87 141.54 137.05 129.32 127.24 125.45 114.28 111.23 110.17 108.86 106.51
48.87 42.72 21.48 4.38 2.05
Ts
N
O
O
230 220 210 200 190 180 170 160 150 140 130 120 110 100 90 80 70 60 50 40 30 20 10 0 -10
f1 (ppm)

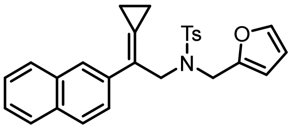

Compound 1r: Yield: 546 mg, 41%; A white solid; M.p.: 130 - 134 ºC; ^{1}H NMR (400 MHz, $CDCl_3$) δ 8.06 - 7.97 (m, 1H), 7.85 - 7.73 (m, 4H), 7.64 - 7.57 (m, 2H), 7.50 -7.40 (m, 2H), 7.18 (d, *J* = 8.0 Hz, 2H), 7.13 - 7.09 (m, 1H), 6.17 (dq, *J* = 3.2, 1.6 Hz, 1H), 5.95 (d, *J* = 3.2 Hz, 1H), 4.56 (s, 2H), 4.29 (s, 2H), 2.39 (s, 3H), 1.47 (t, *J* = 7.8 Hz, 2H), 1.15 (t, *J* = 7.8 Hz, 2H); ^{13}C NMR (100 MHz, $CDCl_3$) δ 150.2, 143.0, 141.8, 136.9, 135.0, 133.4, 132.5, 129.4, 128.5, 128.1, 127.6, 127.43, 127.37, 125.9, 125.8, 125.1, 124.7, 121.9, 110.3, 108.8, 50.4, 42.7, 21.5, 5.2, 1.7; IR (neat): ν 2963, 1597, 1505, 1344, 1260, 1159, 1092, 1011, 803, 746 cm^{-1}; HRMS (ESI) Calcd. for $C_{27}H_{25}NO_3SNa$ $[M+Na]^+$: 466.1447, Found: 466.1448.

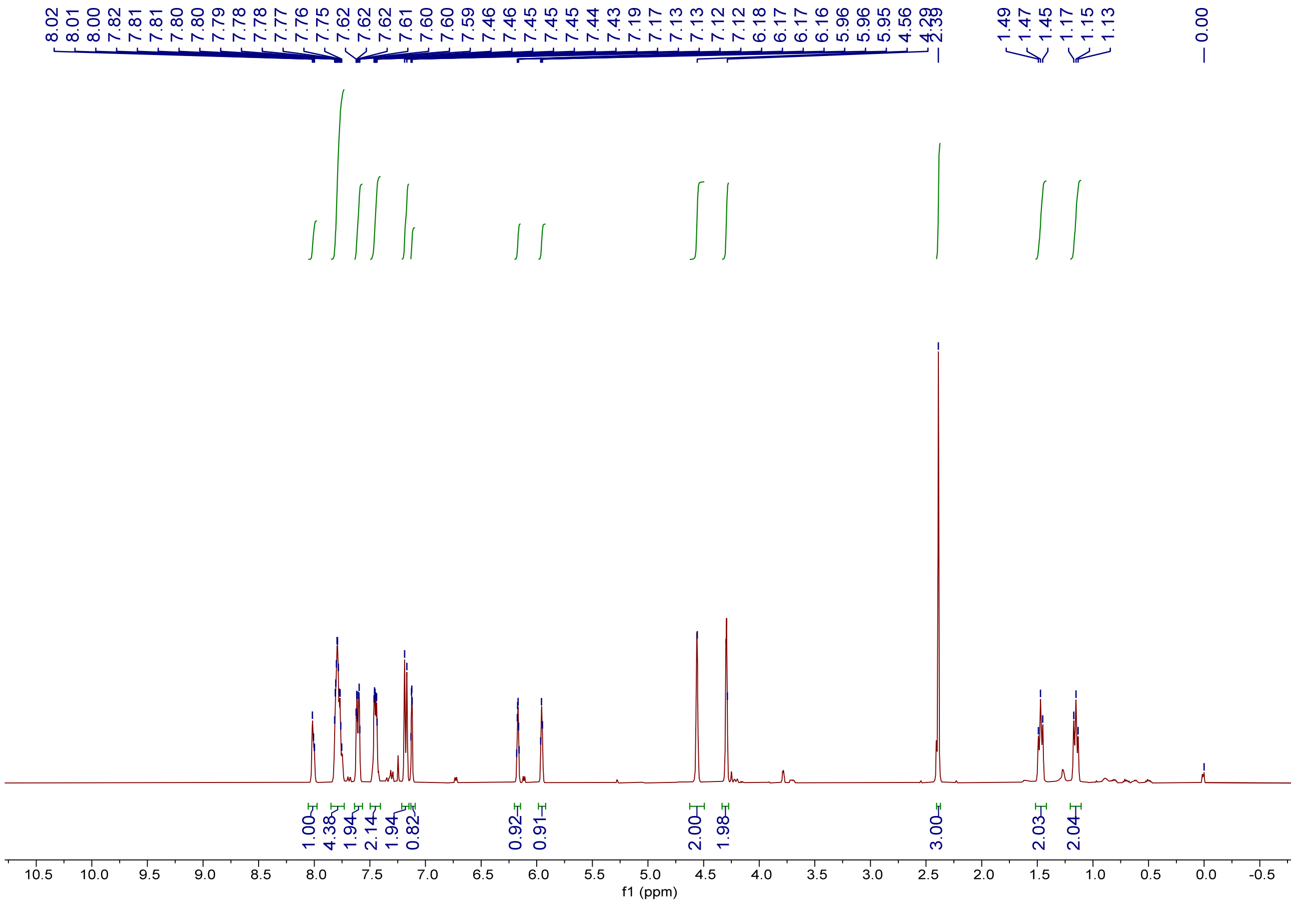
8.02 8.01 8.00 7.82 7.81 7.81 7.80 7.80 7.79 7.78 7.78 7.77 7.76 7.75 7.62 7.62 7.62 7.61 7.60 7.59 7.46 7.46 7.45 7.45 7.45 7.44 7.43 7.19 7.17 7.13 7.13 7.12 7.12 6.18 6.17 6.17 6.16 5.96 5.96 5.95 4.56 4.29 2.39
1.49 1.47 1.45 1.17 1.15 1.13
0.00
1.00 4.38 1.94 2.14 1.94 0.82 0.92 0.91 2.00 1.98 3.00 2.03 2.04
f1 (ppm)

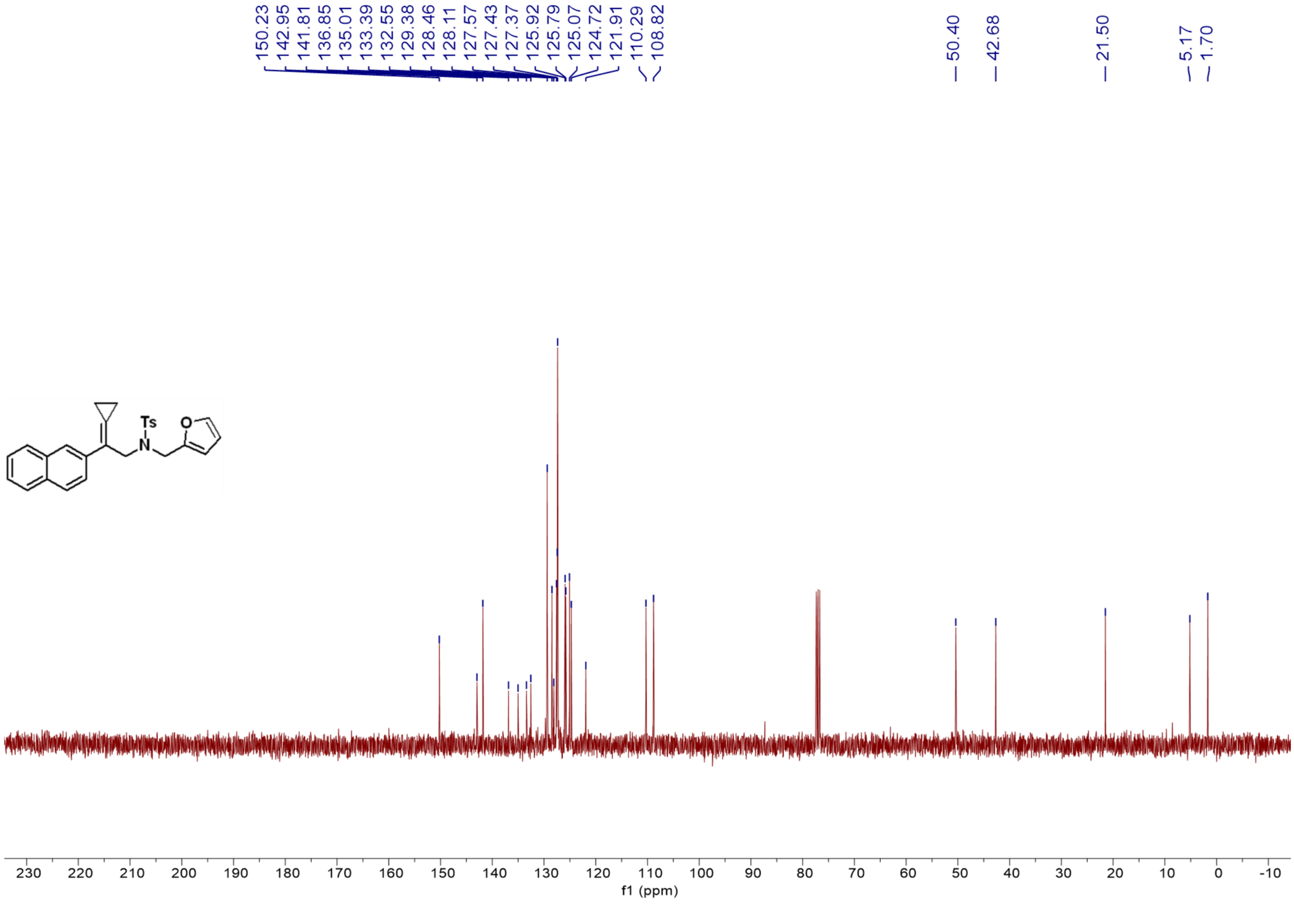
150.23 142.95 141.81 136.85 135.01 133.39 132.55 129.38 128.46 128.11 127.57 127.43 127.37 125.92 125.79 125.07 124.72 121.91 110.29 108.82
50.40 42.68 21.50 5.17 1.70
f1 (ppm)
Ts
N
O

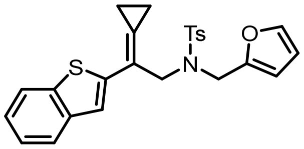

Compound 1s: Yield: 1025 mg, 78%; A white solid; M.p.: 116 - 120 ºC; ^{1}H NMR (400 MHz, $CDCl_3$) δ 7.72 (dd, *J* = 13.4, 7.6 Hz, 2H), 7.64 (d, *J* = 7.8 Hz, 2H), 7.48 (s, 1H), 7.34 - 7.19 (m, 4H), 7.10 (s, 1H), 6.20 - 6.11 (m, 1H), 5.96 (d, *J* = 3.2 Hz, 1H), 4.45 (s, 2H), 4.35 (s, 2H), 2.41 (s, 3H), 1.44 (dd, *J* = 9.6, 6.4 Hz, 2H), 1.25 (dd, *J* = 9.6, 6.4 Hz, 2H); ^{13}C NMR (100 MHz, $CDCl_3$) δ 150.0, 143.1, 142.9, 141.9, 140.3, 139.2, 136.6, 129.53, 129.48, 127.4, 124.3, 124.2, 123.9, 121.7, 120.3, 118.2, 110.3, 108.9, 50.2, 42.8, 21.5, 5.8, 3.4; IR (neat): ν 2962, 1337, 1260, 1159, 1090, 1024, 797, 746 cm^{-1}; HRMS (ESI) Calcd. for $C_{25}H_{22}NO_3S_2Na$ $[M+Na]^+$: 472.1012, Found: 472.1007.

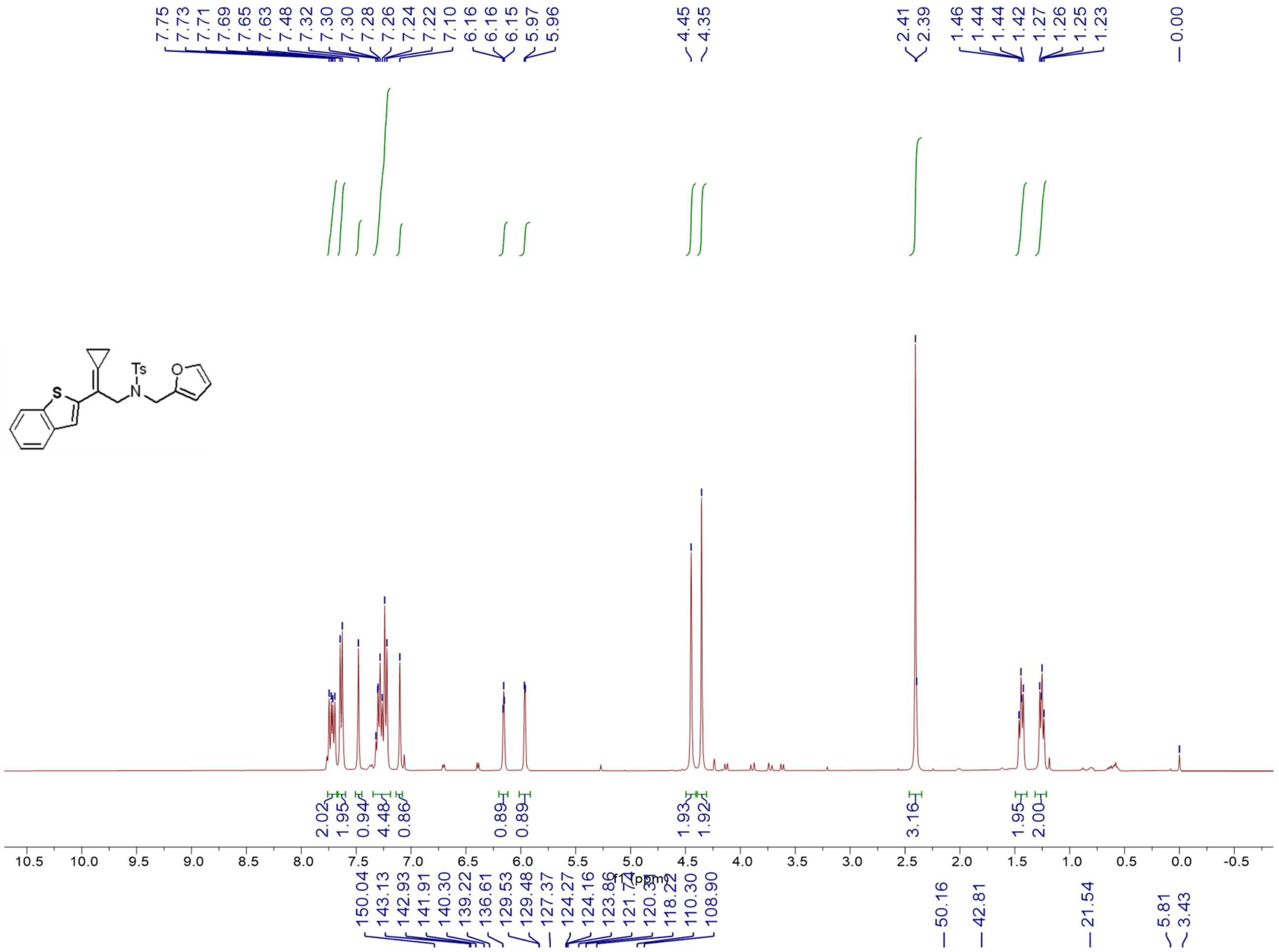

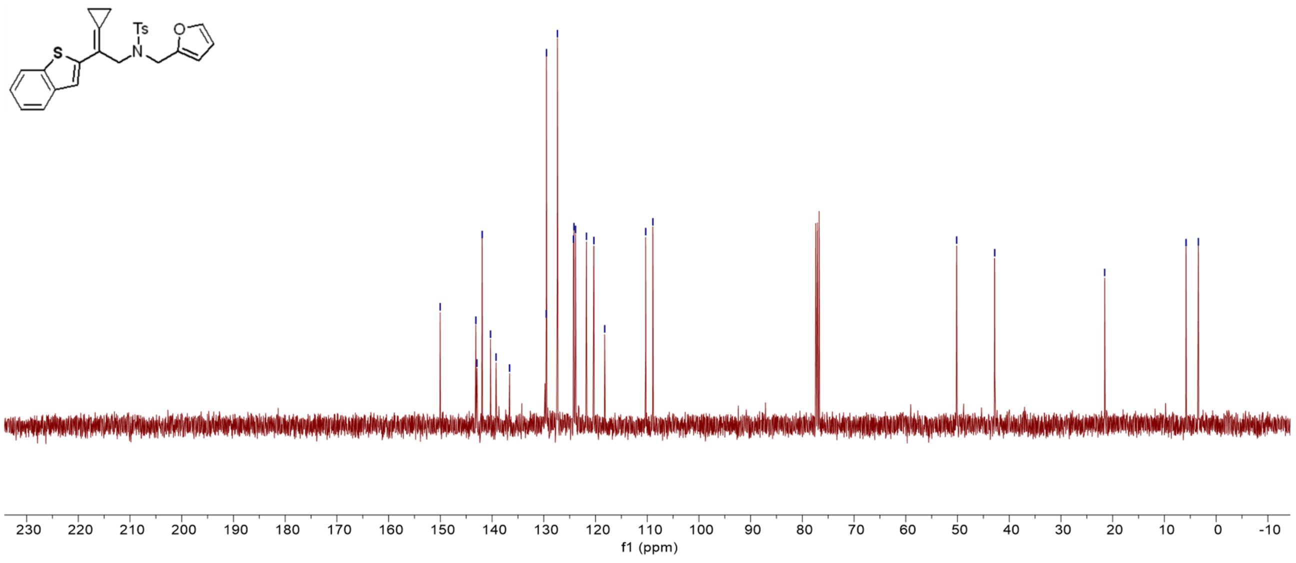

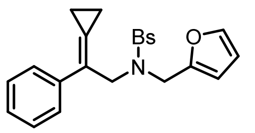

Compound 1t: Yield: 626 mg, 46%; A white solid; M.p.: 82 - 84 ºC; ^{1}H NMR (400 MHz, $CDCl_3$) δ 7.63 - 7.57 (m, 2H), 7.52 (d, J = 1.2 Hz, 4H), 7.36 - 7.29 (m, 2H), 7.29 - 7.22 (m, 1H), 6.19 (dd, J = 3.2, 1.8Hz, 1H), 5.99 (d, J = 3.2 Hz, 1H), 4.47 (s, 2H), 4.27 (s, 2H), 1.41 (dd, J = 9.2, 6.4 Hz, 2H), 1.14 (dd, J = 9.2, 6.4 Hz, 2H); ^{13}C NMR (100 MHz, $CDCl_3$) δ 149.8, 142.0, 138.8, 137.4, 131.9, 128.8, 128.3, 127.7, 127.2, 127.0, 126.3, 121.7, 110.2, 109.1, 50.3, 42.4, 4.9, 1.7; IR (neat): ν 1574, 1349, 1162, 1091, 1068, 1009, 758, 739, 697 cm^{-1}; HRMS (ESI) Calcd. for $C_{22}H_{20}NO_3SNaBr$ $[M+Na]^+$: 480.240, Found: 480.248.

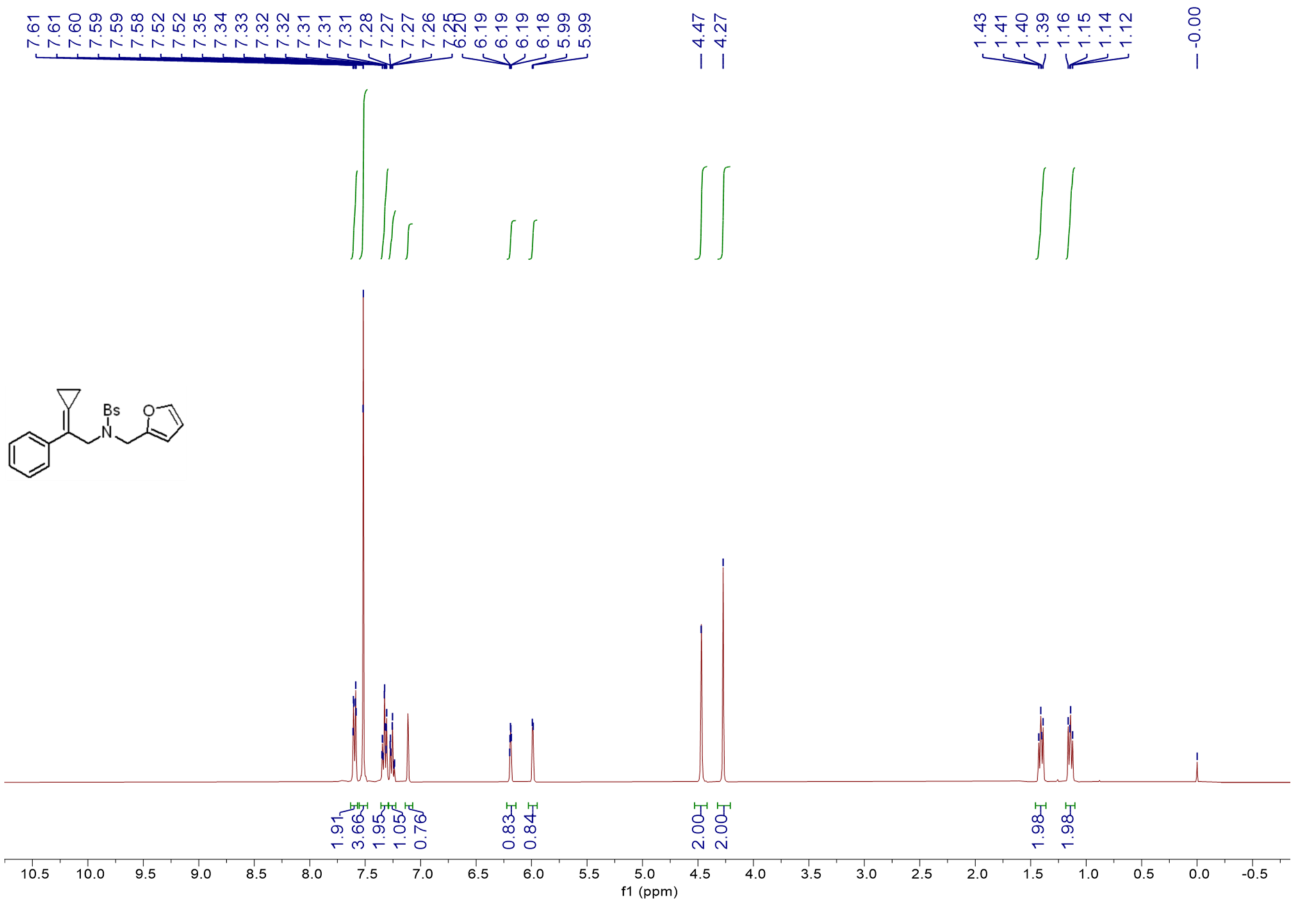
7.61 7.61 7.60 7.59 7.59 7.58 7.52 7.52 7.35 7.34 7.33 7.32 7.32 7.31 7.31 7.31 7.28 7.27 7.27 7.26 7.25 6.20 6.19 6.19 6.19 6.18 5.99 5.99
4.47 4.27
1.43 1.41 1.40 1.39 1.16 1.15 1.14 1.12
-0.00
Bs
N
O
1.91 3.66 1.95 1.05 0.76 0.83 0.84 2.00 2.00 1.98 1.98
10.5 10.0 9.5 9.0 8.5 8.0 7.5 7.0 6.5 6.0 5.5 5.0 4.5 4.0 3.5 3.0 2.5 2.0 1.5 1.0 0.5 0.0 -0.5
f1 (ppm)

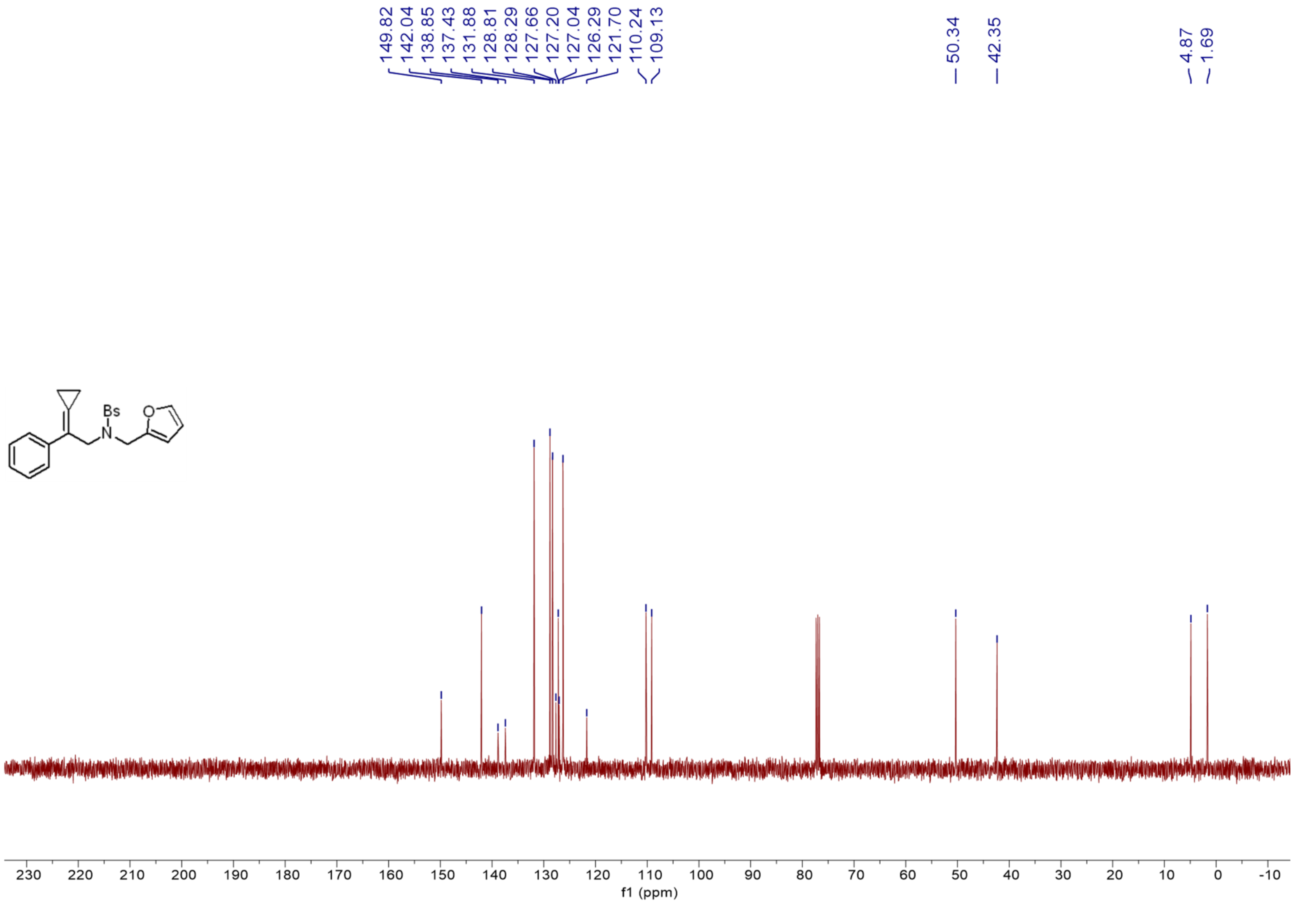
149.82 142.04 138.85 137.43 131.88 128.81 128.29 127.66 127.20 127.04 126.29 121.70 110.24 109.13
50.34 42.35
4.87 1.69
Bs
N
O
230 220 210 200 190 180 170 160 150 140 130 120 110 100 90 80 70 60 50 40 30 20 10 0 -10
f1 (ppm)

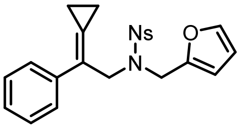

Compound 1u: Yield: 820 mg, 64%; A white solid; M.p.: 87 - 90 ºC; ^{1}H NMR (400 MHz, $CDCl_3$) δ 8.19 (d, *J* = 8.8 Hz, 2H), 7.79 (d, *J* = 8.8 Hz, 2H), 7.60 (d, *J* = 7.6 Hz, 2H), 7.33 (t, *J* = 7.6 Hz, 2H), 7.29 – 7.24 (m, 1H), 7.07 (d, *J* = 1.8 Hz, 1H), 6.18 (dd, *J* = 3.2, 1.8 Hz, 1H), 6.04 (d, *J* = 3.2 Hz, 1H), 4.54 (s, 2H), 4.33 (s, 2H), 1.43 (dd, *J* = 9.4, 6.5 Hz, 2H), 1.18 (dd, *J* = 9.3, 6.6 Hz, 2H); ^{13}C NMR (100 MHz, $CDCl_3$) δ 149.5, 145.6, 142.2, 137.3, 128.39, 128.36, 128.1, 127.4, 126.3, 123.8, 121.5, 110.3, 109.5, 50.6, 42.3, 4.9, 1.8; IR (neat): ν 2963, 1528, 1348, 1260, 1162, 1091, 1025, 1012, 798, 742 cm^{-1}; HRMS (ESI) Calcd. for $C_{22}H_{20}N_2O_5SNa$ $[M+Na]^+$: 447.0985, Found: 447.0987.

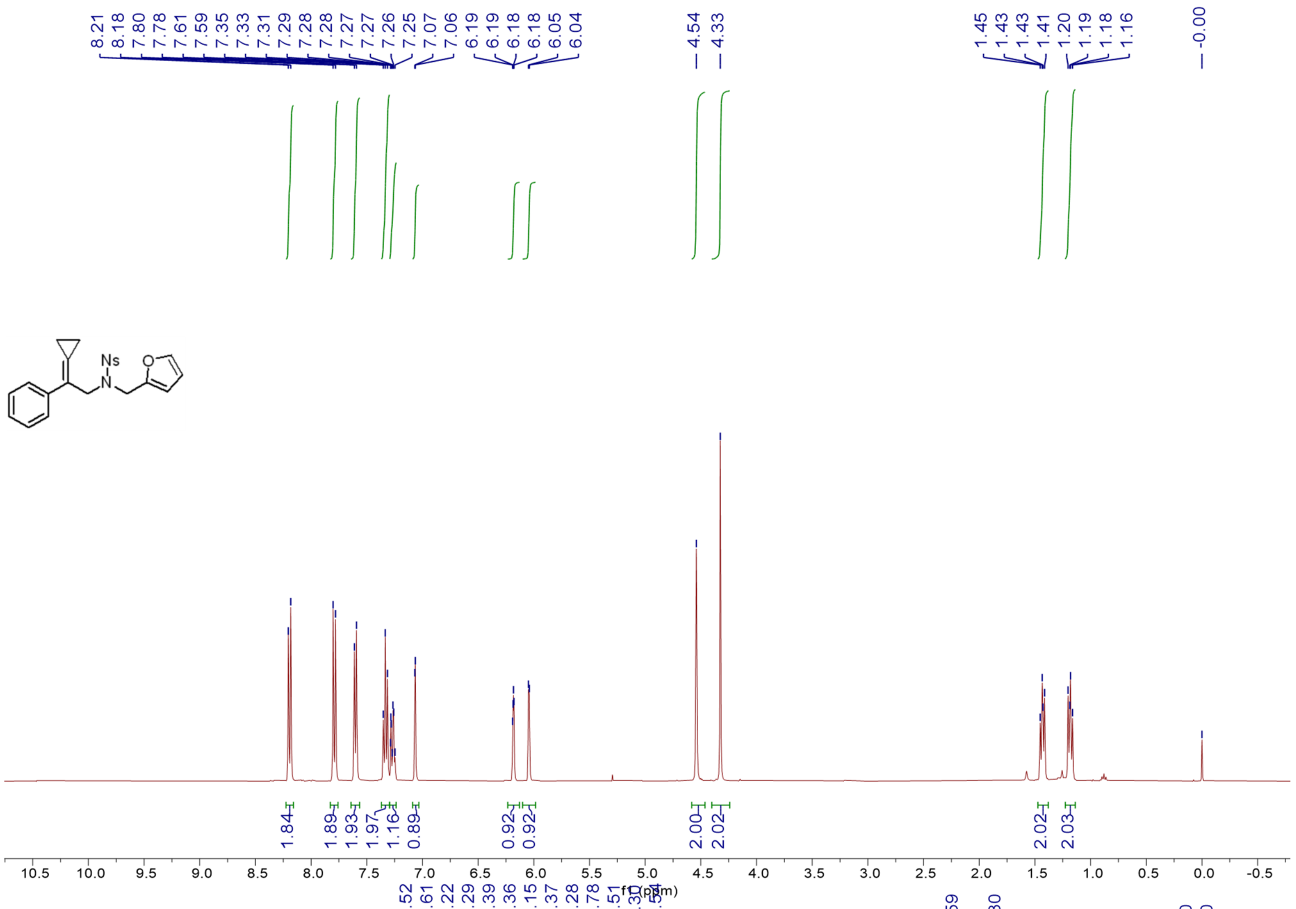

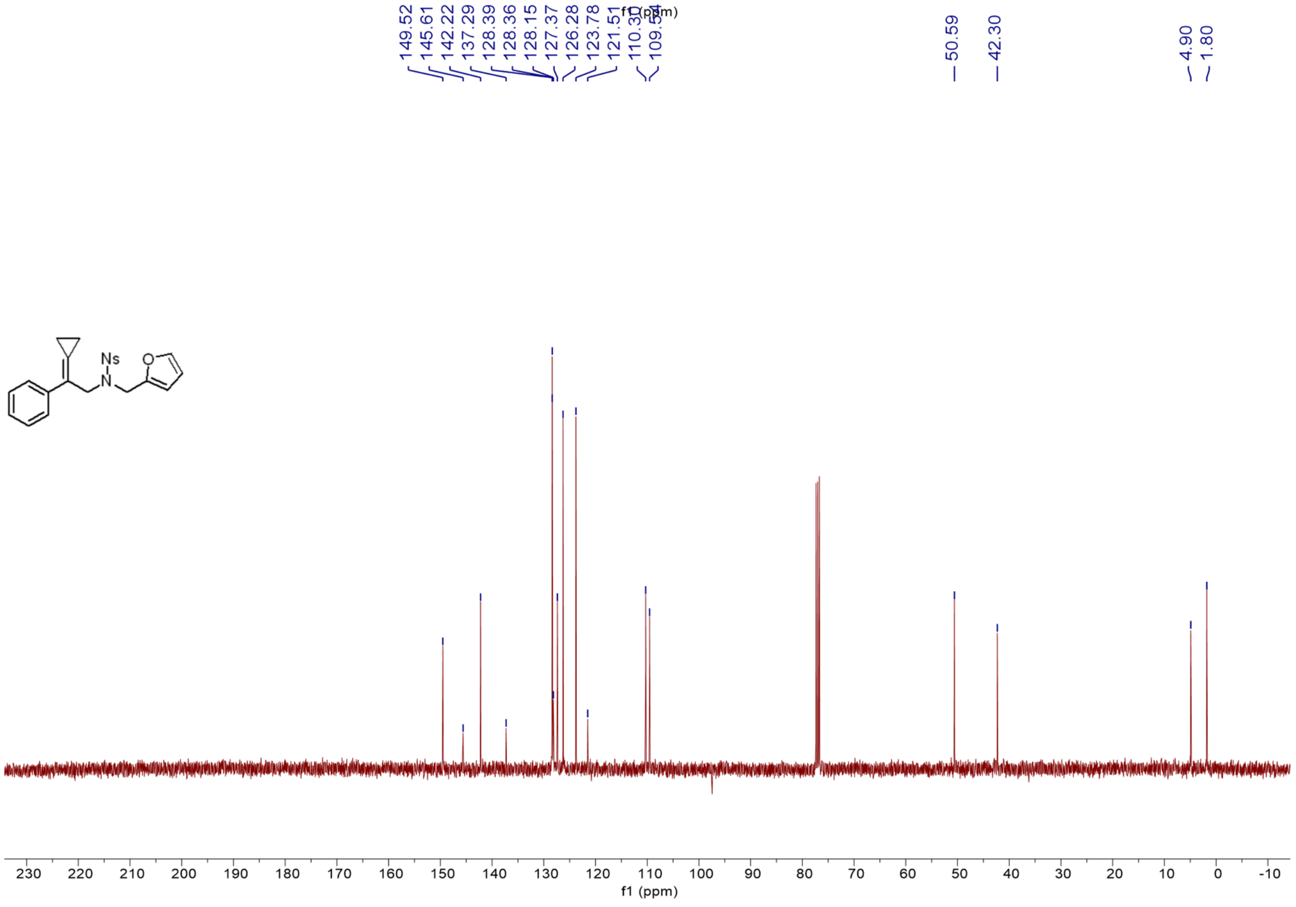

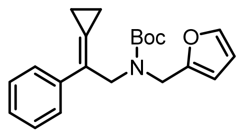

Compound 1v: Yield: 310 mg, 61%; A yellow oil; ^{1}H NMR (400 MHz, $CDCl_3$) (Mixture of two rotamers 66 : 34) δ 7.71 - 7.53 (m, 2H), 7.36 - 7.25 (m, 3H), 7.25 - 7.15 (m, 1H), 6.26 (dd, *J* = 3.2, 1.8 Hz, 1H), 6.13 (s, 0.4H), 6.06 (s, 0.6H), 4.57 (s, 1.2H), 4.48 (s, 0.8H), 4.32 (s, 1.2H), 4.19 (s, 0.8H), 1.47 (s, 9H), 1.43 - 1.35 (m, 2H), 1.16 (t, *J* = 7.6 Hz, 2H); ^{13}C NMR (100 MHz, $CDCl_3$) δ 155.4, 152.2, 152.0, 141.8, 141.7, 138.6, 138.1, 128.2, 127.0, 126.1, 124.3, 123.7, 123.4, 123.2, 110.2, 107.7, 107.2, 79.9, 48.8, 48.0, 41.8, 41.6, 28.4, 4.4, 3.8, 1.5, 1.1; IR (neat): ν 2976, 2931, 1691, 1413, 1367, 1251, 1159, 1011, 884, 761, 698 cm^{-1}; HRMS (ESI) Calcd. for $C_{21}H_{25}NO_3Na$ $[M+Na]^+$: 362.1727, Found: 362.1721.

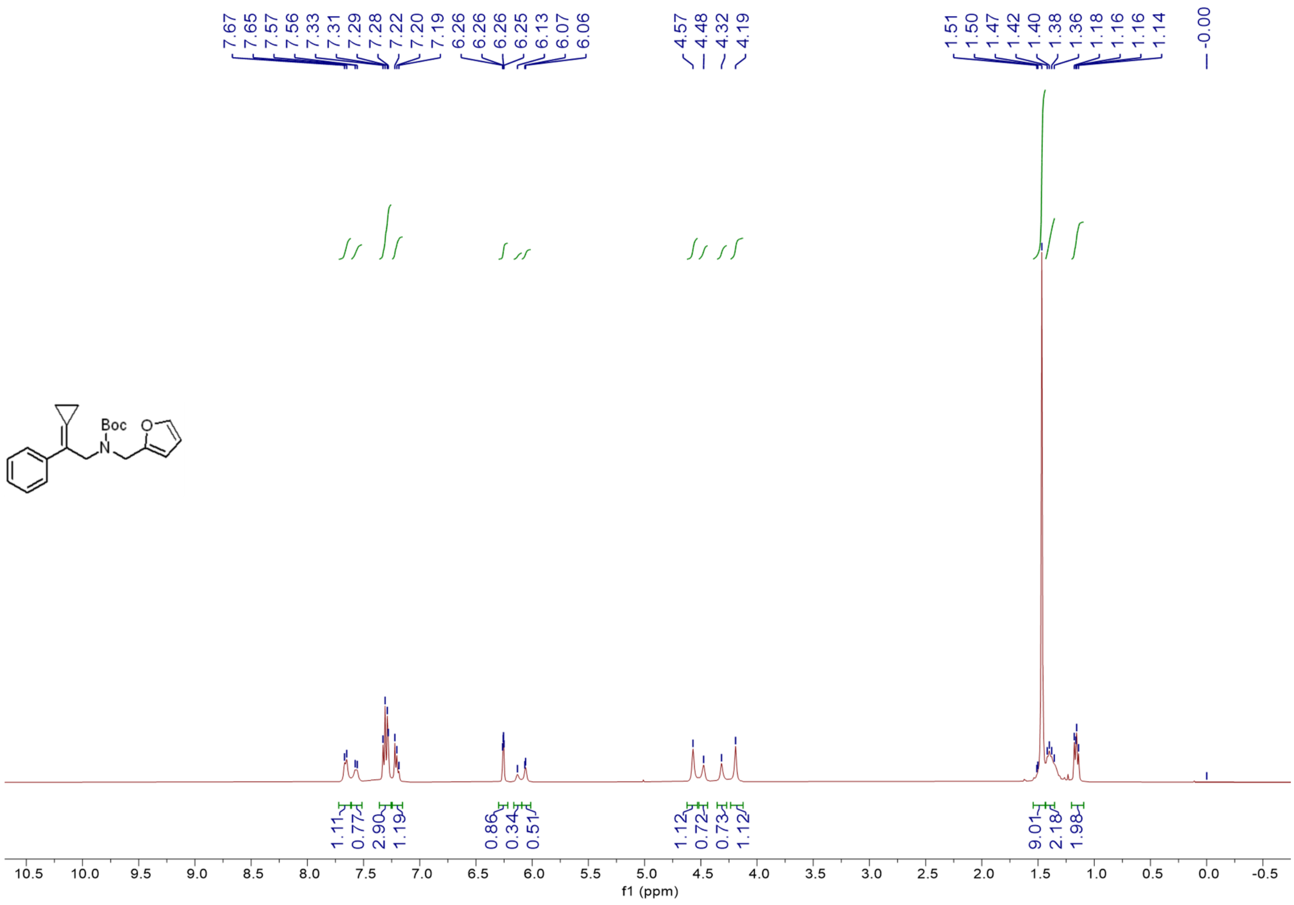
7.67 7.65 7.57 7.56 7.33 7.31 7.29 7.28 7.22 7.20 7.19 6.26 6.26 6.25 6.13 6.07 6.06
4.57 4.48 4.32 4.19
1.51 1.50 1.47 1.42 1.40 1.38 1.36 1.18 1.16 1.16 1.14
-0.00
Boc
N
O
1.11 0.77 2.90 1.19 0.86 0.34 0.51 1.12 0.72 0.73 1.12 9.01 2.18 1.98
f1 (ppm)

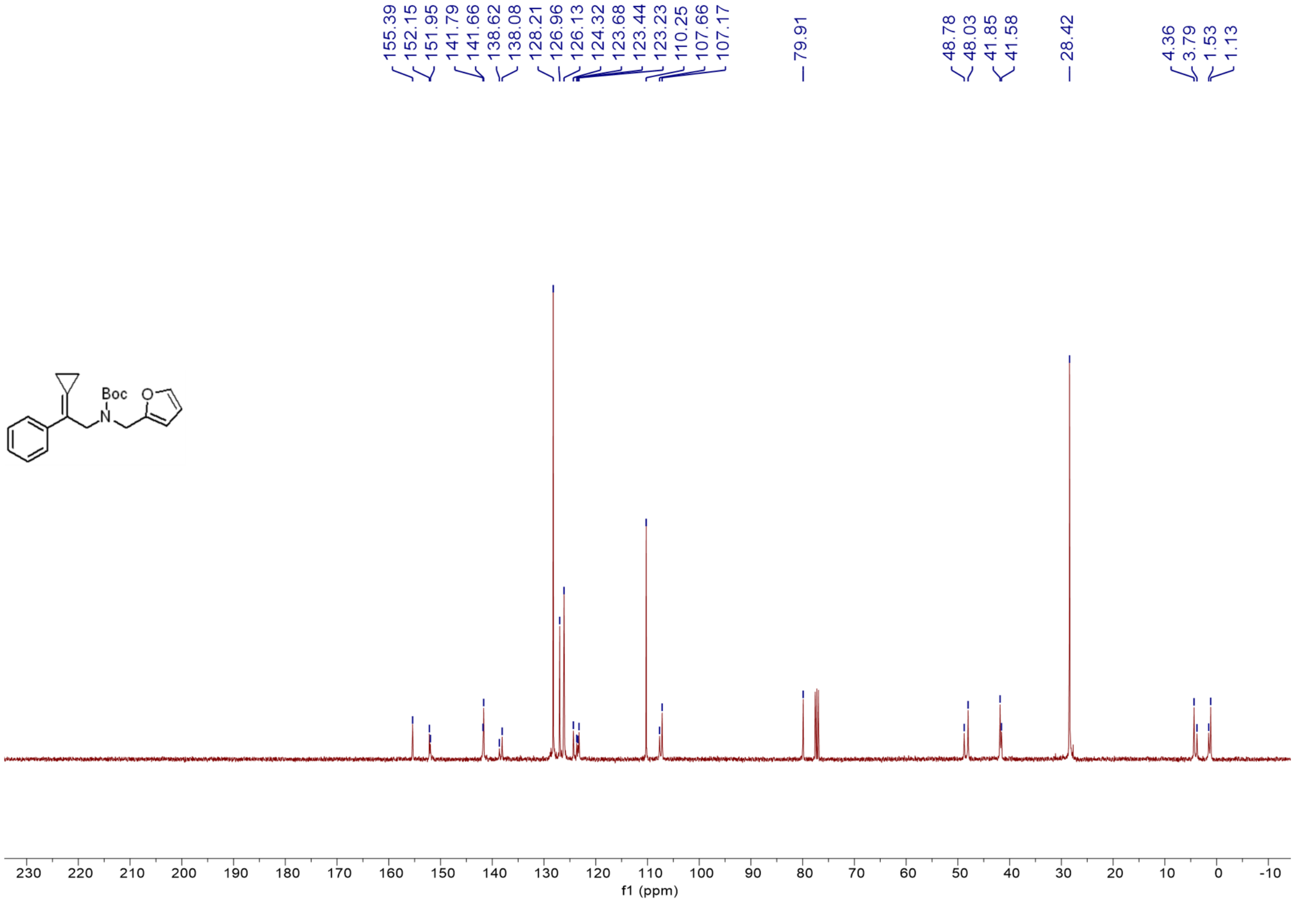
155.39 152.15 151.95 141.79 141.66 138.62 138.08 128.21 126.96 126.13 124.32 123.68 123.44 123.23 110.25 107.66 107.17
79.91
48.78 48.03 41.85 41.58
28.42
4.36 3.79 1.53 1.13
Boc
N
O
f1 (ppm)

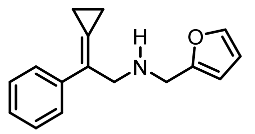

Compound 1w: Yield: 366 mg, 75%; A yellow oil; ^{1}H NMR (400 MHz, $CDCl_3$) δ 7.60 (d, *J* = 8.3 Hz, 2H), 7.38 - 7.30 (m, 3H), 7.28 - 7.20 (m, 1H), 6.36 - 6.28 (m, 1H), 6.20 - 6.13 (m, 1H), 3.84 (s, 2H), 3.78 (s, 2H), 1.47 - 1.38 (m, 2H), 1.24 - 1.17 (m, 2H); ^{13}C NMR (100 MHz, $CDCl_3$) δ 154.0, 141.7, 138.8, 128.4, 126.8, 126.0, 125.2, 123.3, 110.1, 106.9, 51.4, 45.4, 4.5, 1.2; IR (neat): ν 2963, 1496, 1445, 1075, 1026, 798, 732, 693, cm^{-1}; HRMS (ESI) Calcd. for $C_{16}H_{18}NO$ $[M+H]^+$: 240.1383, Found: 240.1379.

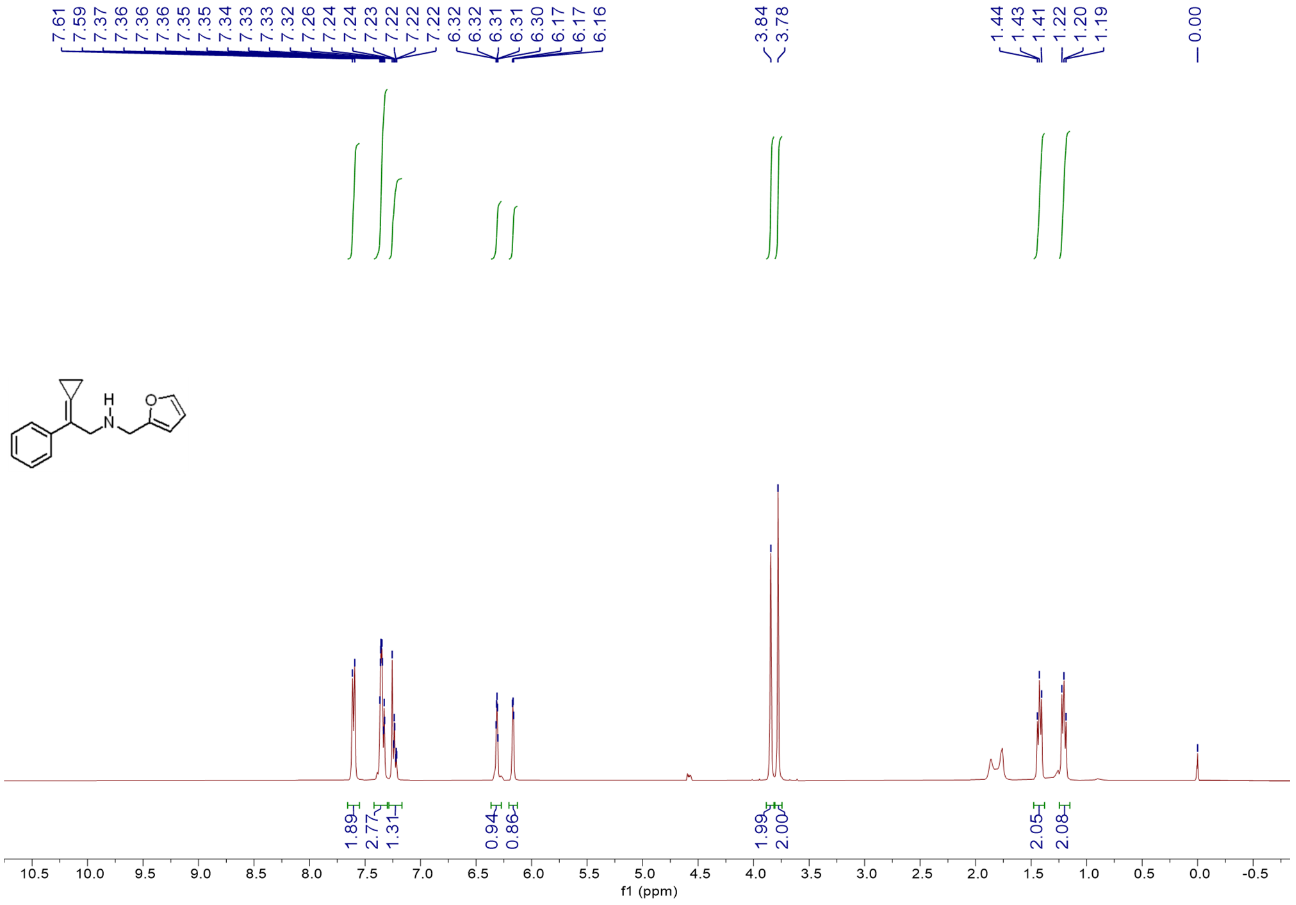

7.61
7.59
7.37
7.36
7.36
7.36
7.35
7.35
7.34
7.33
7.33
7.32
7.26
7.24
7.24
7.23
7.22
7.22
7.22
6.32
6.32
6.31
6.31
6.30
6.17
6.17
6.16
3.84
3.78
1.44
1.43
1.41
1.22
1.20
1.19
0.00
H
N
O
1.89
2.77
1.31
0.94
0.86
1.99
2.00
2.05
2.08
f1 (ppm)

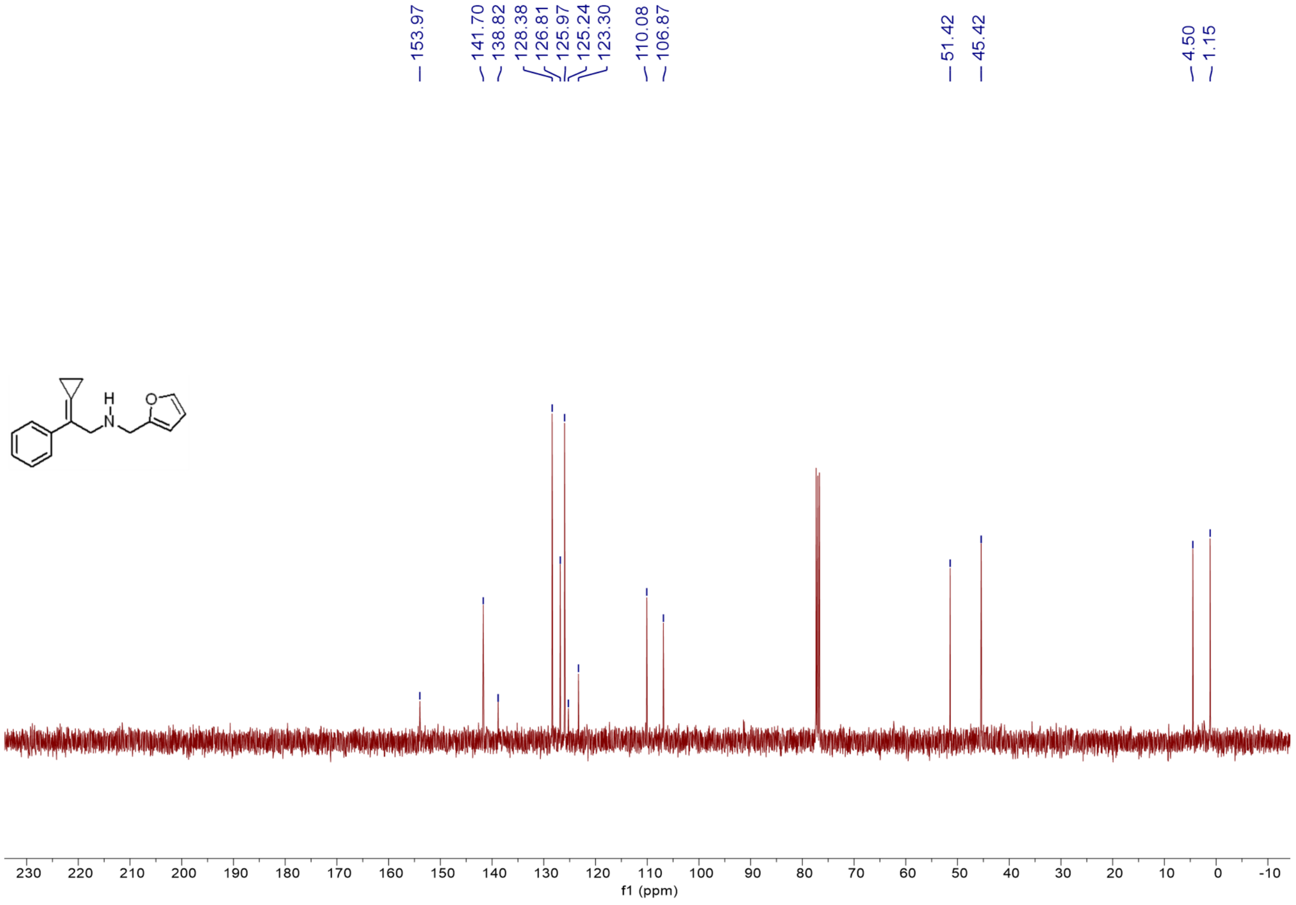

153.97
141.70
138.82
128.38
126.81
125.97
125.24
123.30
110.08
106.87
51.42
45.42
4.50
1.15
H
N
O
f1 (ppm)

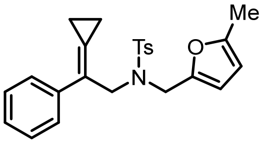

Compound 1x: Yield: 930 mg, 76%; A white solid; M.p.: 58 - 60 ºC; ^{1}H NMR (400 MHz, $CDCl_3$) δ 7.69 - 7.55 (m, 4H), 7.38 - 7.28 (m, 2H), 7.27 - 7.15 (m, 3H), 5.78 (t, J = 2.6 Hz, 1H), 5.73 - 5.68 (m, 1H), 4.44 (s, 2H), 4.19 (s, 2H), 2.40 (s, 3H), 2.00 (s, 3H), 1.43 - 1.33 (m, 2H), 1.17 – 1.05 (m, 2H); ^{13}C NMR (100 MHz, $CDCl_3$) δ 151.4, 148.1, 142.7, 137.6, 136.9, 129.2, 128.2, 127.4, 127.0, 126.9, 126.3, 121.9, 109.7, 105.9, 50.2, 42.6, 21.5, 13.2, 4.8, 1.6; IR (neat): ν 2962, 1698, 1597, 1345, 1260, 1159, 1091, 1021, 798, 662 cm^{-1}; HRMS (ESI) Calcd. for $C_{24}H_{25}NO_3SNa$ $[M+Na]^+$: 430.1447, Found: 430.1443.

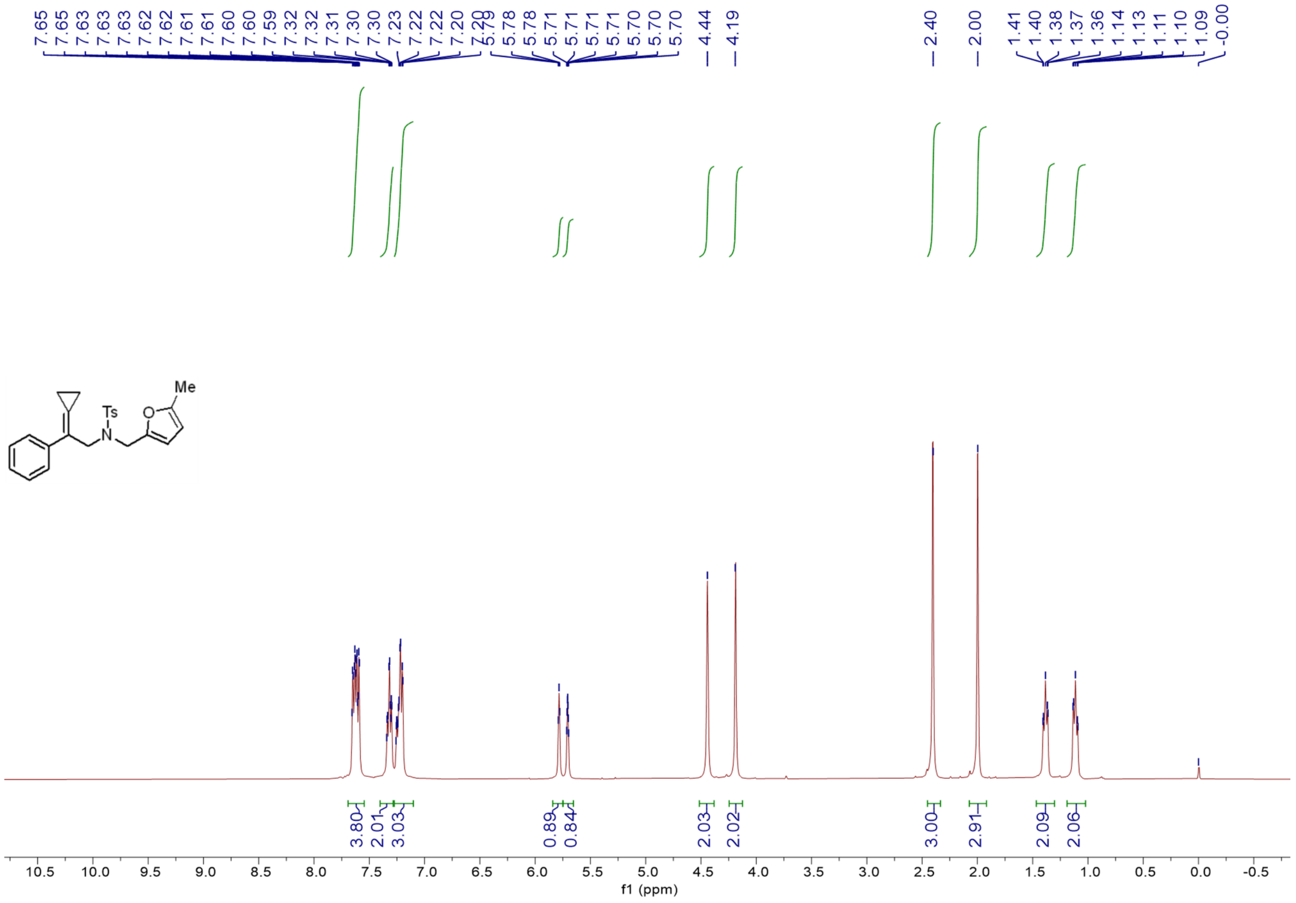
7.65 7.65 7.63 7.63 7.63 7.62 7.62 7.61 7.61 7.60 7.60 7.59 7.32 7.32 7.31 7.30 7.30 7.23 7.22 7.22 7.20 7.20 5.79 5.78 5.78 5.71 5.71 5.71 5.71 5.70 5.70 5.70
4.44
4.19
2.40
2.00
1.41 1.40 1.38 1.37 1.36 1.14 1.13 1.11 1.10 1.09 -0.00
Me
Ts
O
N
3.80 2.01 3.03 0.89 0.84 2.03 2.02 3.00 2.91 2.09 2.06
10.5 10.0 9.5 9.0 8.5 8.0 7.5 7.0 6.5 6.0 5.5 5.0 4.5 4.0 3.5 3.0 2.5 2.0 1.5 1.0 0.5 0.0 -0.5
f1 (ppm)

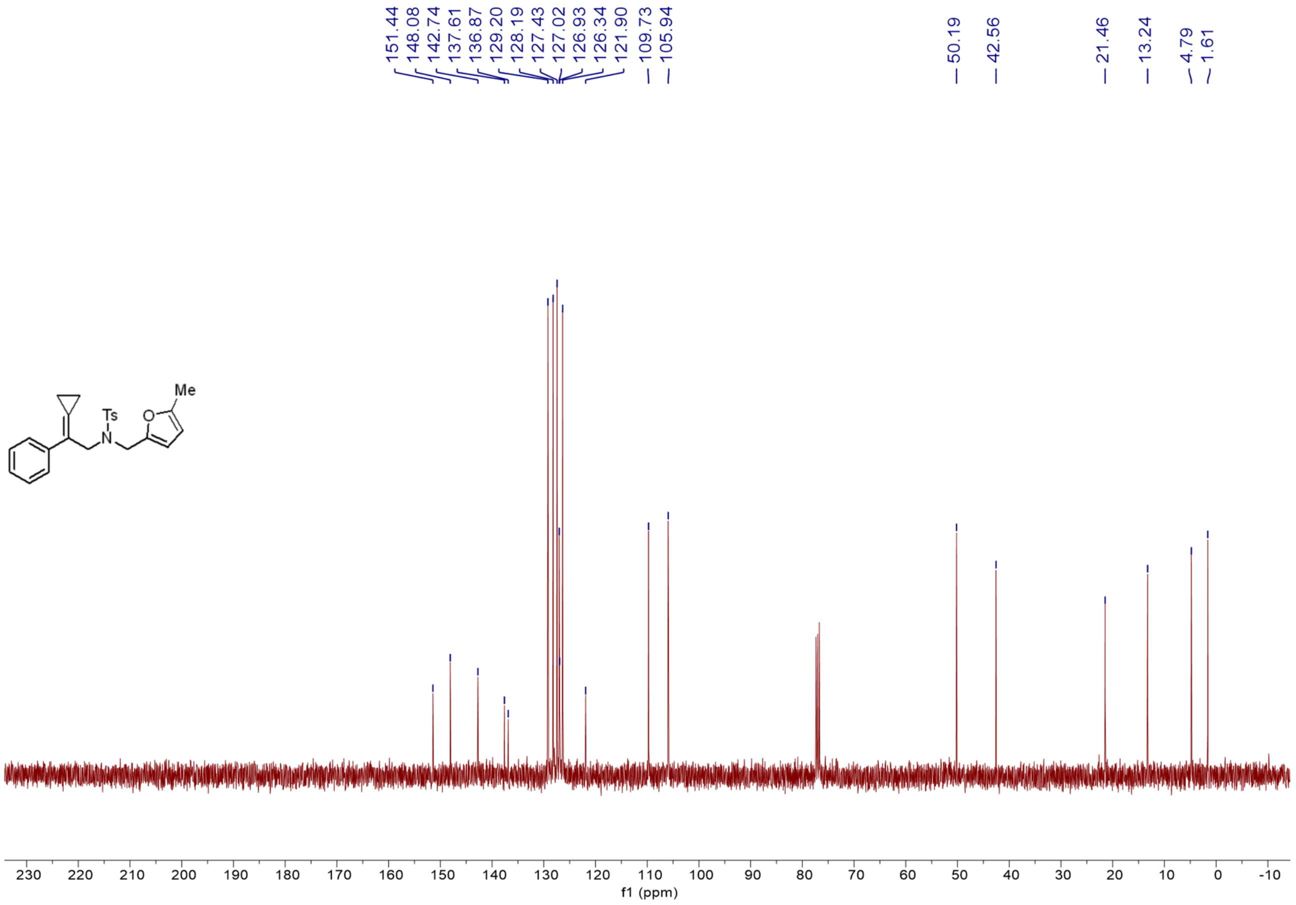
151.44 148.08 142.74 137.61 136.87 129.20 128.19 127.43 127.02 126.93 126.34 121.90
109.73
105.94
50.19
42.56
21.46
13.24
4.79
1.61
Me
Ts
O
N
230 220 210 200 190 180 170 160 150 140 130 120 110 100 90 80 70 60 50 40 30 20 10 0 -10
f1 (ppm)

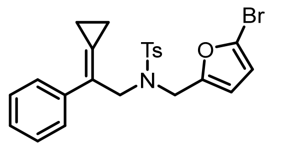

Compound 1y: Yield: 282 mg, 20%; A white solid; M.p.: 88 - 91 ºC; ^{1}H NMR (400 MHz, $CDCl_3$) δ 7.61 (t, *J* = 7.8 Hz, 4H), 7.32 (t, *J* = 7.6 Hz, 2H), 7.28 - 7.19 (m, 3H), 6.04 (d, *J* = 3.2 Hz, 1H), 5.90 (d, *J* = 3.2 Hz, 1H), 4.46 (s, 2H), 4.18 (s, 2H), 2.42 (s, 3H), 1.42 (t, *J* = 7.8 Hz, 2H), 1.15 (t, *J* = 7.8 Hz, 2H); ^{13}C NMR (100 MHz, $CDCl_3$) δ 1152.2, 143.2, 137.3, 136.3, 129.5, 128.2, 127.5, 127.3, 127.1, 126.3, 121.7, 120.8, 111.8, 111.7, 50.6, 42.4, 21.5, 5.0, 1.6; IR (neat): ν 2962, 1598, 1497, 1337, 1306, 1160, 1011, 798, 734, 709 cm^{-1}; HRMS (ESI) Calcd. for $C_{23}H_{22}NO_3SNaBr$ $[M+Na]^+$: 494.0396, Found: 494.0400.

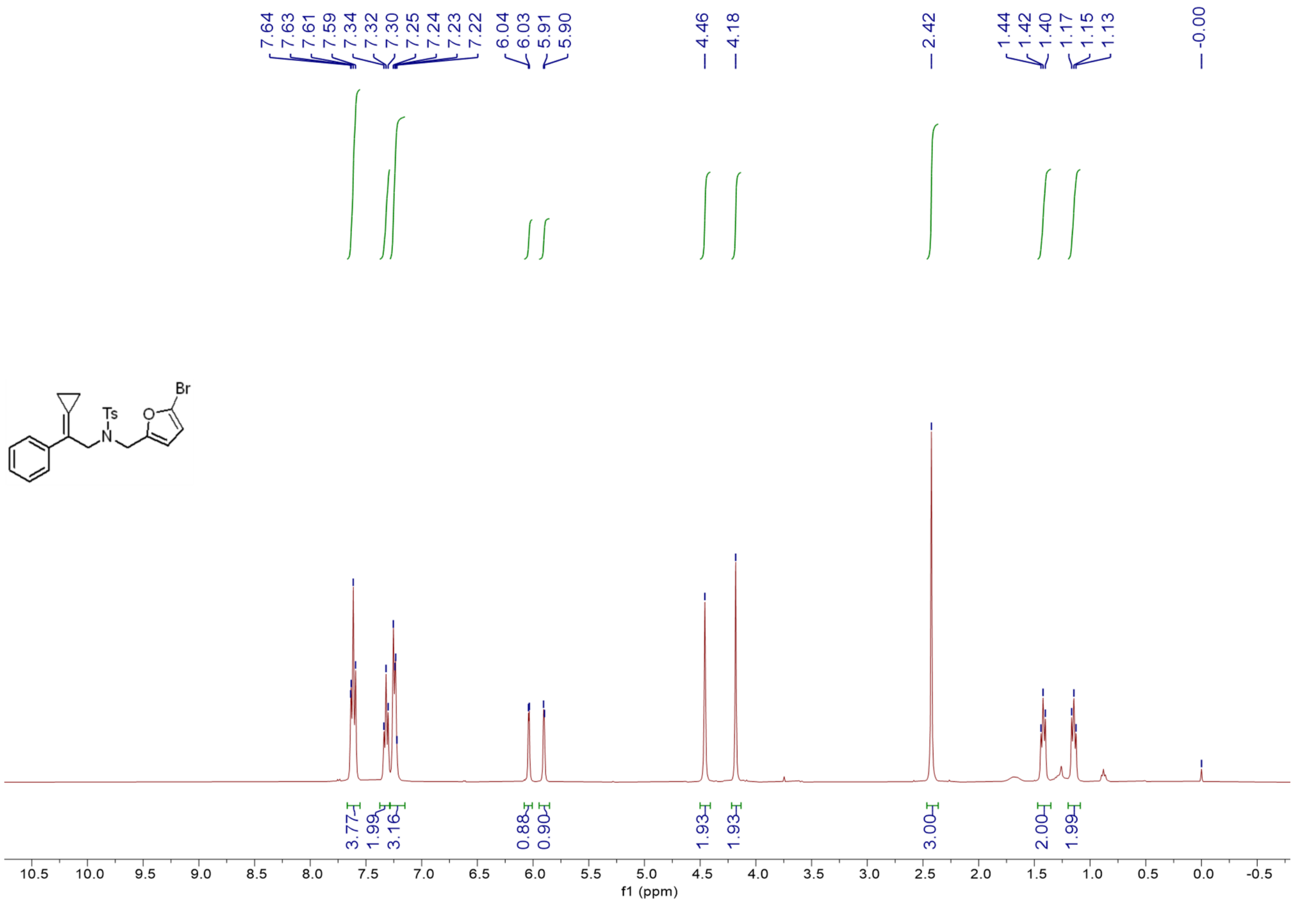
7.64 7.63 7.61 7.59 7.34 7.32 7.30 7.25 7.24 7.23 7.22 6.04 6.03 5.91 5.90
4.46 4.18
2.42
1.44 1.42 1.40 1.17 1.15 1.13
-0.00
Ts
Br
3.77 1.99 3.16 0.88 0.90 1.93 1.93 3.00 2.00 1.99
f1 (ppm)

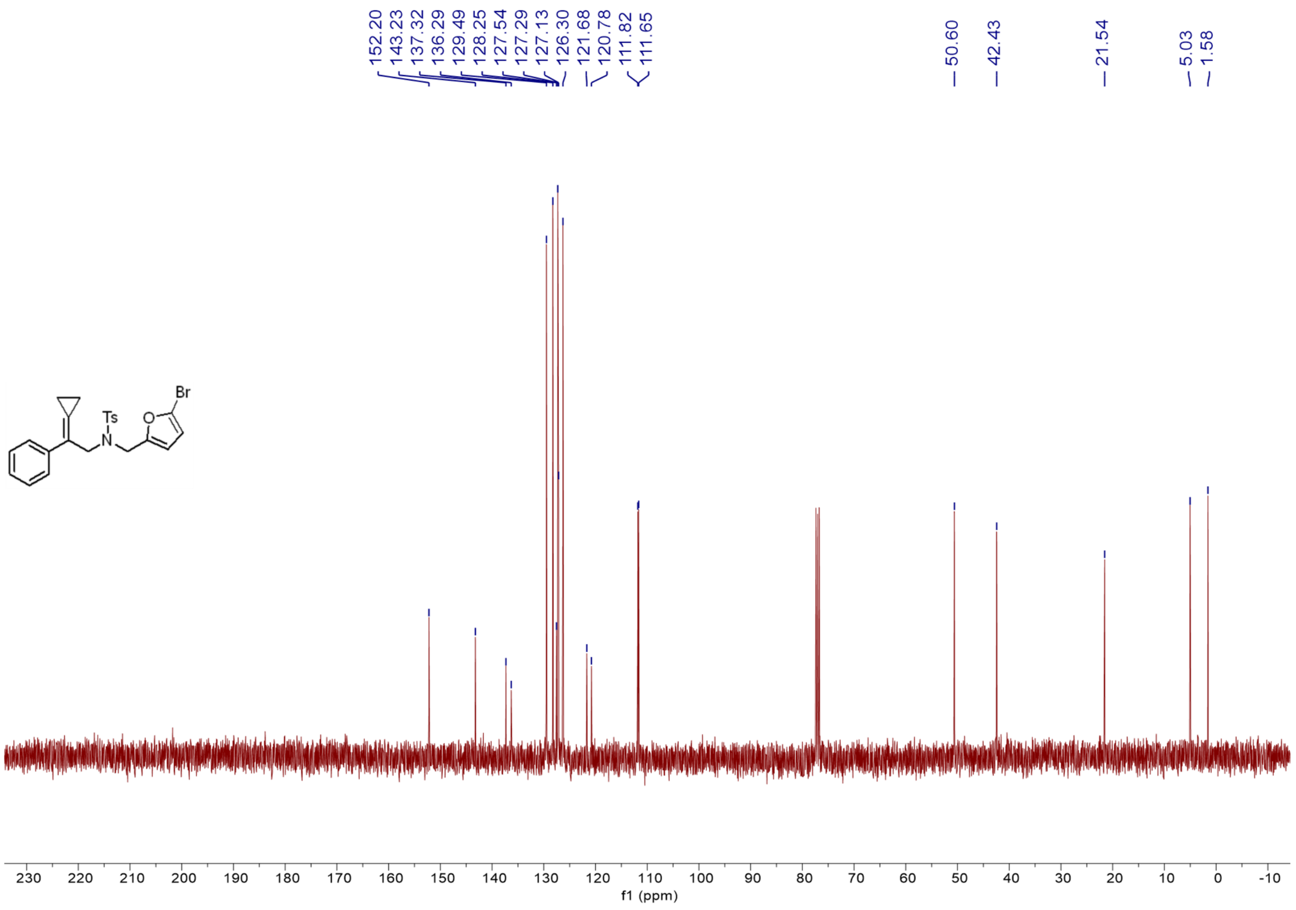
152.20 143.23 137.32 136.29 129.49 128.25 127.54 127.29 127.13 126.30 121.68 120.78 111.82 111.65
50.60 42.43
21.54
5.03 1.58
Ts
Br
f1 (ppm)

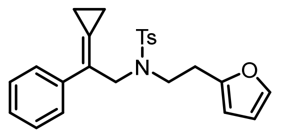

Compound 1z: Yield: 838 mg, 68%; A white solid; M.p.: 85 - 88 °C; ^{1}H NMR (400 MHz, $CDCl_3$) δ 7.71 (t, J = 9.0 Hz, 4H), 7.40 - 7.22 (m, 5H), 7.20 (d, J = 1.8 Hz, 1H), 6.18 (dd, J = 3.2, 1.8 Hz, 1H), 5.81 (d, J = 3.2 Hz, 1H), 4.38 (s, 2H), 3.30 - 3.16 (m, 2H), 2.74 - 2.61 (m, 2H), 2.43 (s, 3H), 1.49 (t, J = 7.8 Hz, 2H), 1.24 - 1.13 (m, 2H); ^{13}C NMR (100 MHz, $CDCl_3$) δ 152.6, 143.3, 141.3, 137.5, 136.1, 129.7, 128.4, 127.4, 127.3, 127.2, 126.3, 122.2, 110.1, 105.9, 51.8, 46.2, 27.6, 21.5, 5.5, 1.5; IR (neat): ν 2966, 1598, 1449, 1260, 1159, 1092, 1019, 920, 802, 695 cm^{-1}; HRMS (ESI) Calcd. for $C_{24}H_{25}NO_3SNa$ $[M+Na]^+$: 430.1447, Found: 430.1447.

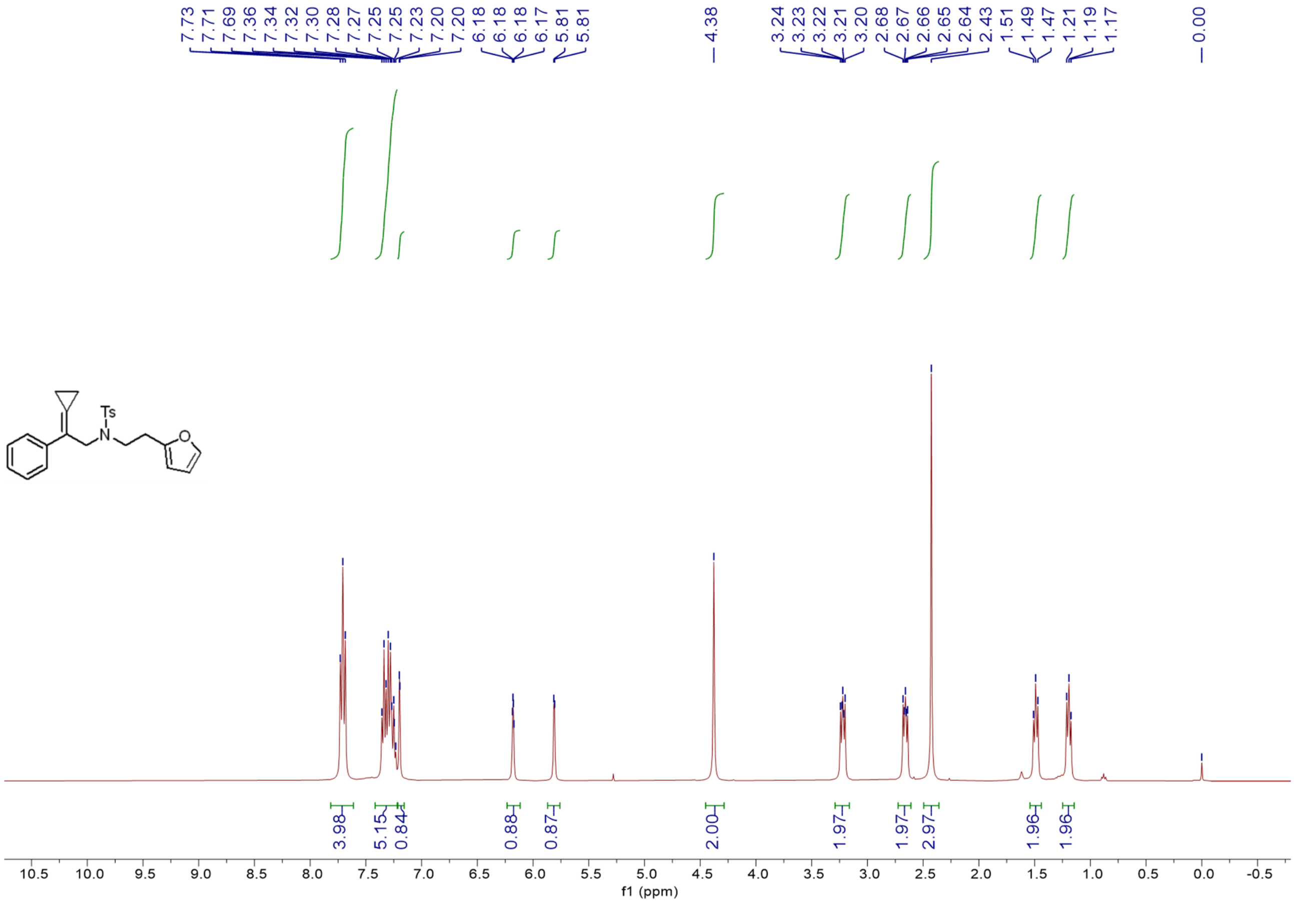
7.73 7.71 7.69 7.36 7.34 7.32 7.30 7.28 7.27 7.25 7.25 7.23 7.20 7.20 6.18 6.18 6.18 6.17 5.81 5.81
4.38
3.24 3.23 3.22 3.21 3.20 2.68 2.67 2.66 2.65 2.64 2.43 1.51 1.49 1.47 1.21 1.19 1.17
0.00
Ts
N
O
3.98 5.15 0.84 0.88 0.87 2.00 1.97 1.97 2.97 1.96 1.96
f1 (ppm)

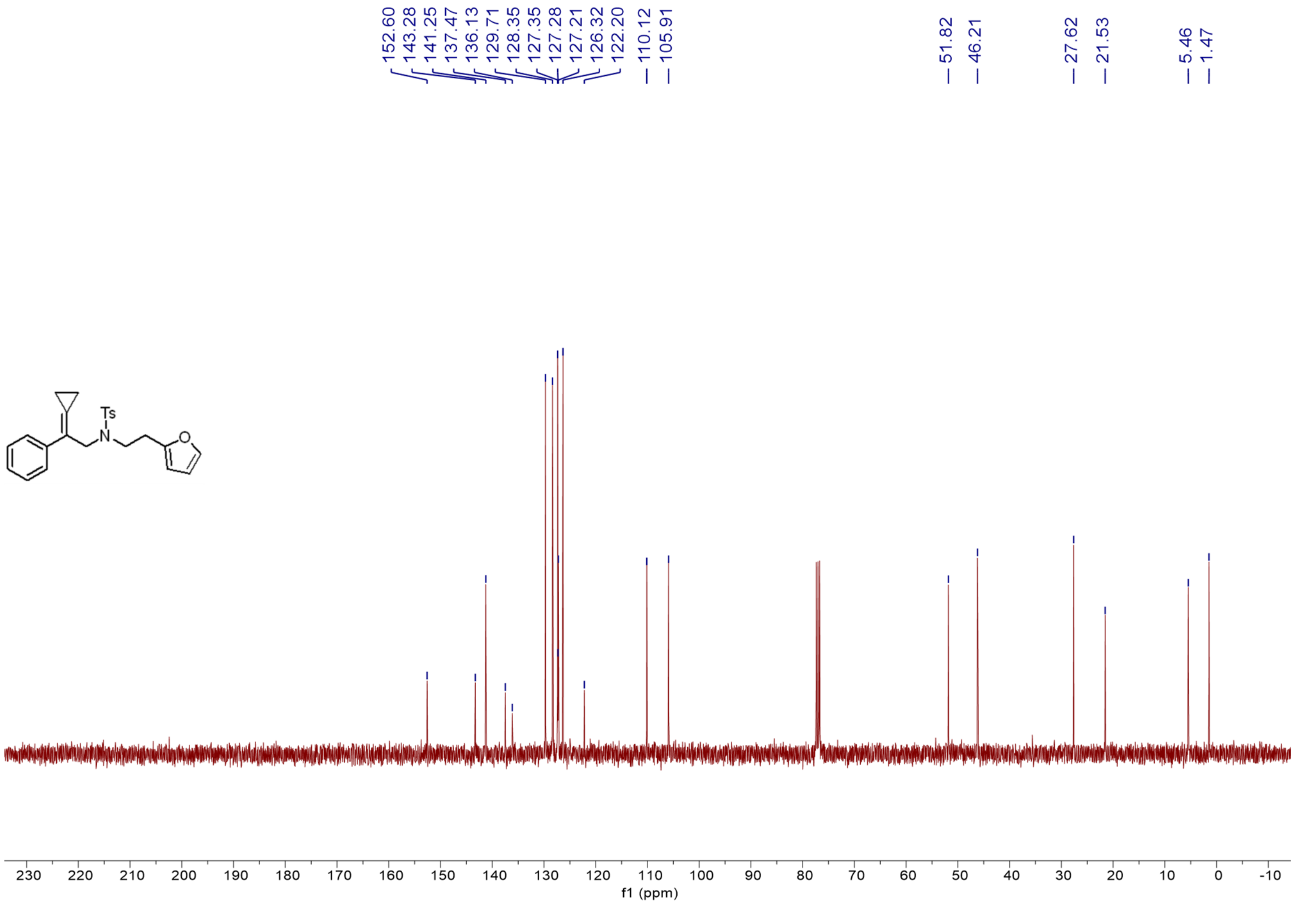
152.60 143.28 141.25 137.47 136.13 129.71 128.35 127.35 127.28 127.21 126.32 122.20
110.12
105.91
51.82
46.21
27.62
21.53
5.46
1.47
Ts
N
O
f1 (ppm)

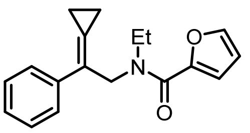

Compound 1aa : Yield: 217 mg, 77%; A white solid; M.p.: 75 - 78 °C; ^{1}H NMR (400 MHz, $CDCl_3$) δ 7.62 (s, 2H), 7.45 (s, 1H), 7.35 (t, *J* = 7.6 Hz, 2H), 7.27 (d, *J* = 6.4 Hz, 1H), 6.96 (s, 1H), 6.43 (s, 1H), 4.80 (s, 2H), 3.51 (q, *J* = 7.2 Hz, 2H), 1.39 (s, 2H), 1.24 -1.10 (m, 5H); ^{13}C NMR (100 MHz, $CDCl_3$) δ 158.5, 146.6, 142.2, 136.7, 126.8, 126.2, 125.6, 124.2, 121.1, 114.1, 109.6, 48.7, 45.5, 40.2, 12.4, 11.0, 2.7, 1.3, 0.0; IR (neat): ν 2973, 1621, 1571, 1489, 1427, 1288, 1073, 1012, 756, 695 cm^{-1}; HRMS (ESI) Calcd. for $C_{18}H_{19}NO_2Na$ $[M+Na]^+$: 304.1308, Found: 304.1304.

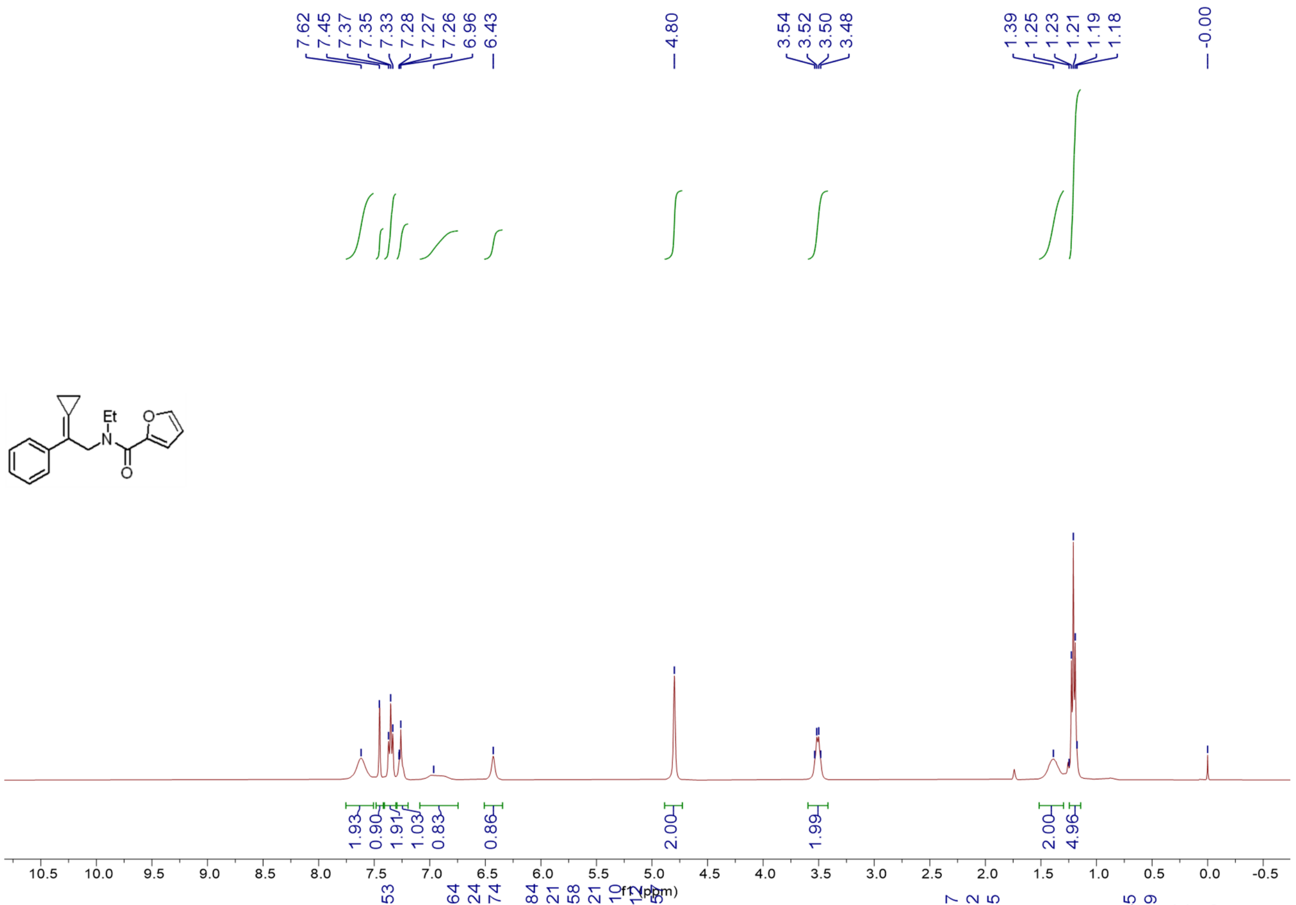

7.62
7.45
7.37
7.35
7.33
7.28
7.27
7.26
6.96
6.43
4.80
3.54
3.52
3.50
3.48
1.39
1.25
1.23
1.21
1.19
1.18
-0.00
Et
N
O
1.93
0.90
1.91
1.03
0.83
0.86
2.00
1.99
2.00
4.96
f1 (ppm)

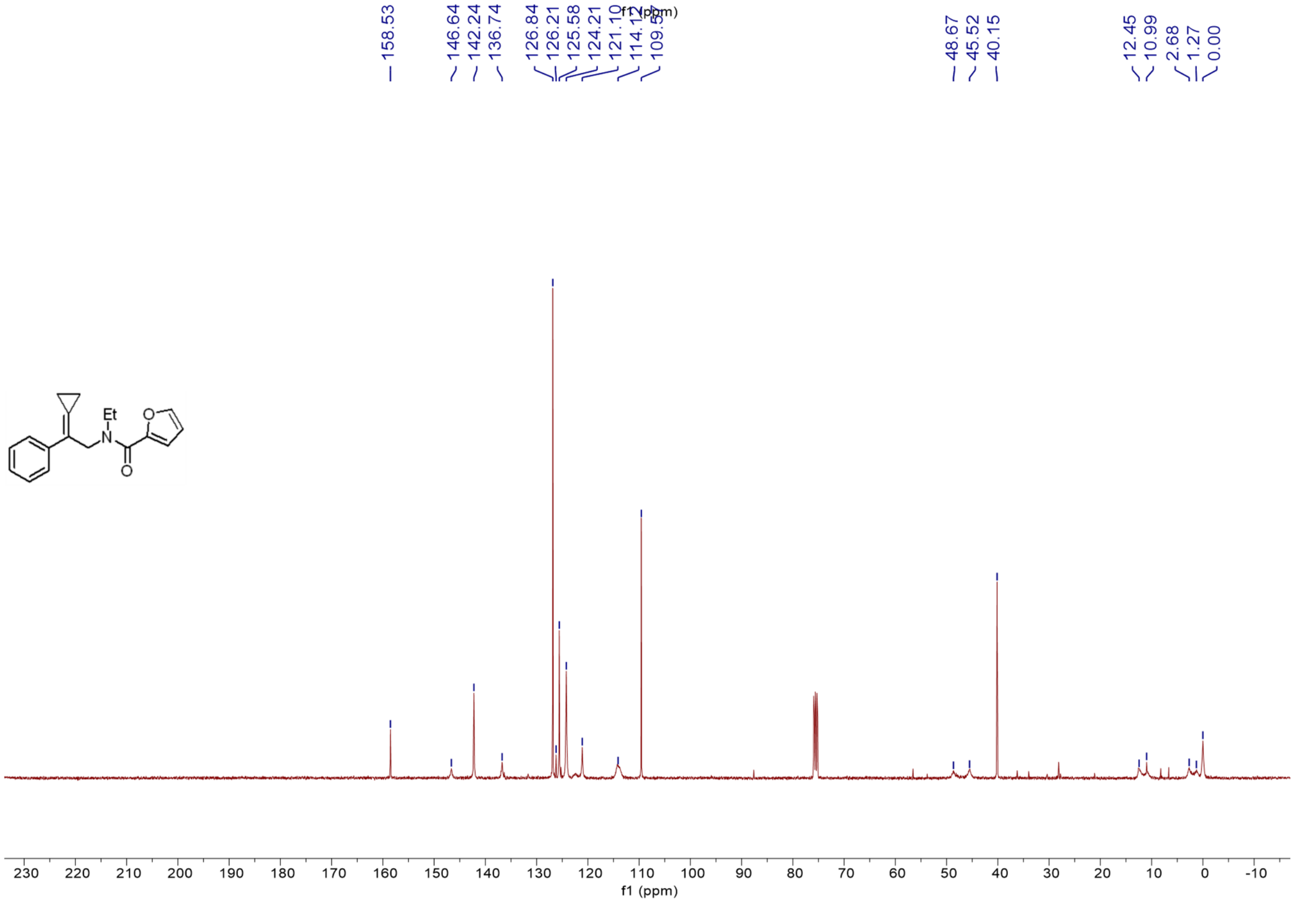

158.53
146.64
142.24
136.74
126.84
126.21
125.58
124.21
121.10
114.17
109.57
48.67
45.52
40.15
12.45
10.99
2.68
1.27
0.00
Et
N
O
f1 (ppm)

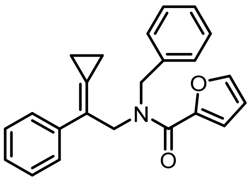

Compound 1ab: Yield: 172 mg, 50%; A yellow oil; ^{1}H NMR (400 MHz, $CDCl_3$) δ 7.57 (s, 2H), 7.44 (s, 1H), 7.38 - 7.08 (m, 8H), 6.94 (d, *J* = 3.6 Hz, 1H), 6.42 (dd, *J* = 3.6, 1.8 Hz, 1H), 4.81 - 4.62 (m, 2H), 1.35 (t, *J* = 7.8 Hz, 2H), 1.22 - 0.82 (m, 2H); ^{13}C NMR (100 MHz, $CDCl_3$) δ 159.3, 146.2, 142.6, 136.7, 135.6, 127.1, 126.9, 126.7, 125.9, 125.7, 125.4, 124.2, 120.5, 114.5, 109.7, 48.5, 45.8, 2.6, 1.4, 0.0; IR (neat): ν 3033, 2973, 1624, 1486, 1421, 1262, 1028, 755, 695 cm^{-1}; HRMS (ESI) Calcd. for $C_{23}H_{22}NO_2$ $[M+H]^+$: 344.1645, Found: 344.1638.

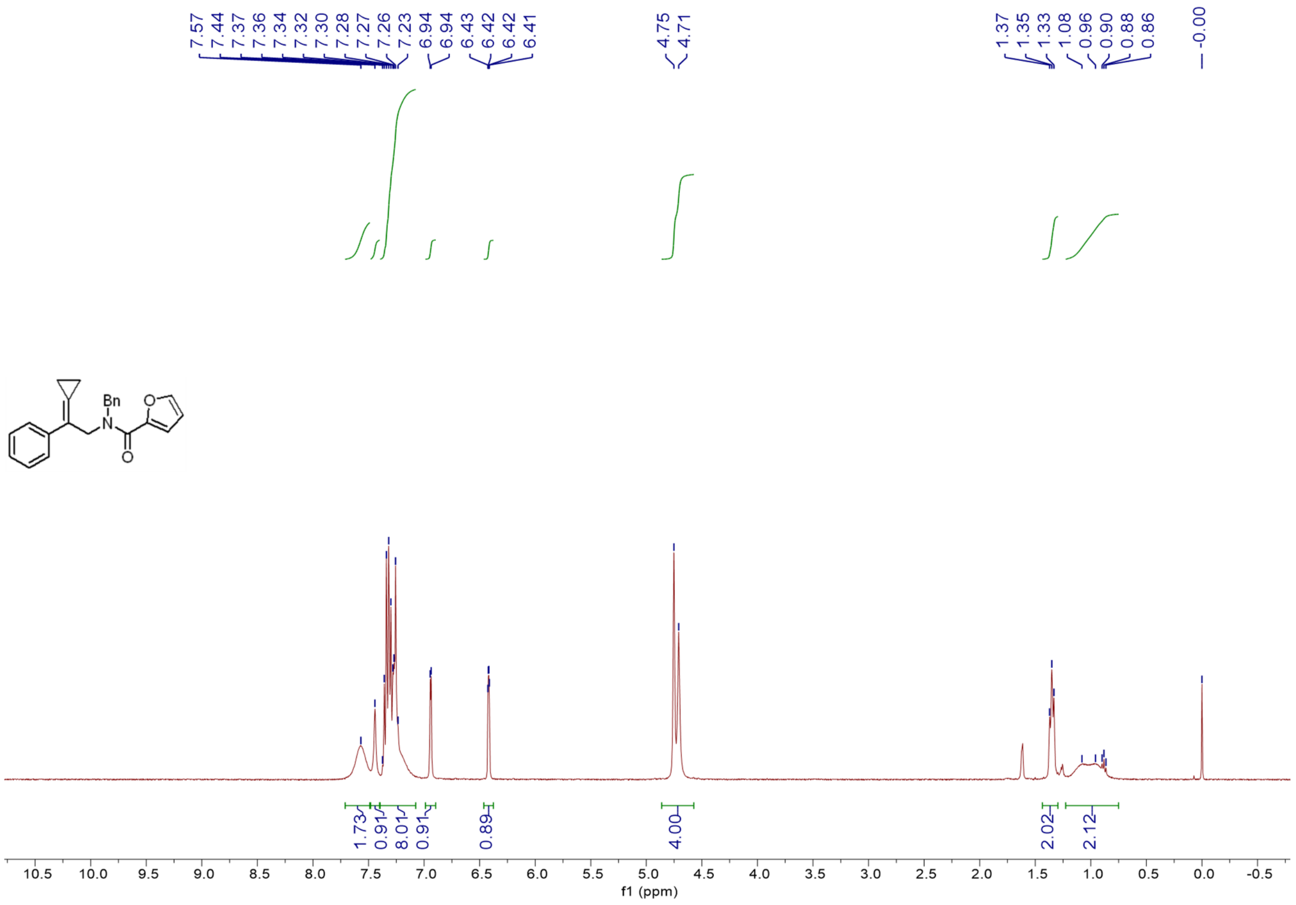
7.57
7.44
7.37
7.36
7.34
7.32
7.30
7.28
7.27
7.26
7.23
6.94
6.94
6.43
6.42
6.42
6.41
4.75
4.71
1.37
1.35
1.33
1.08
0.96
0.90
0.88
0.86
-0.00
Bn
N
O
O
1.73
0.91
8.01
0.91
0.89
4.00
2.02
2.12
f1 (ppm)

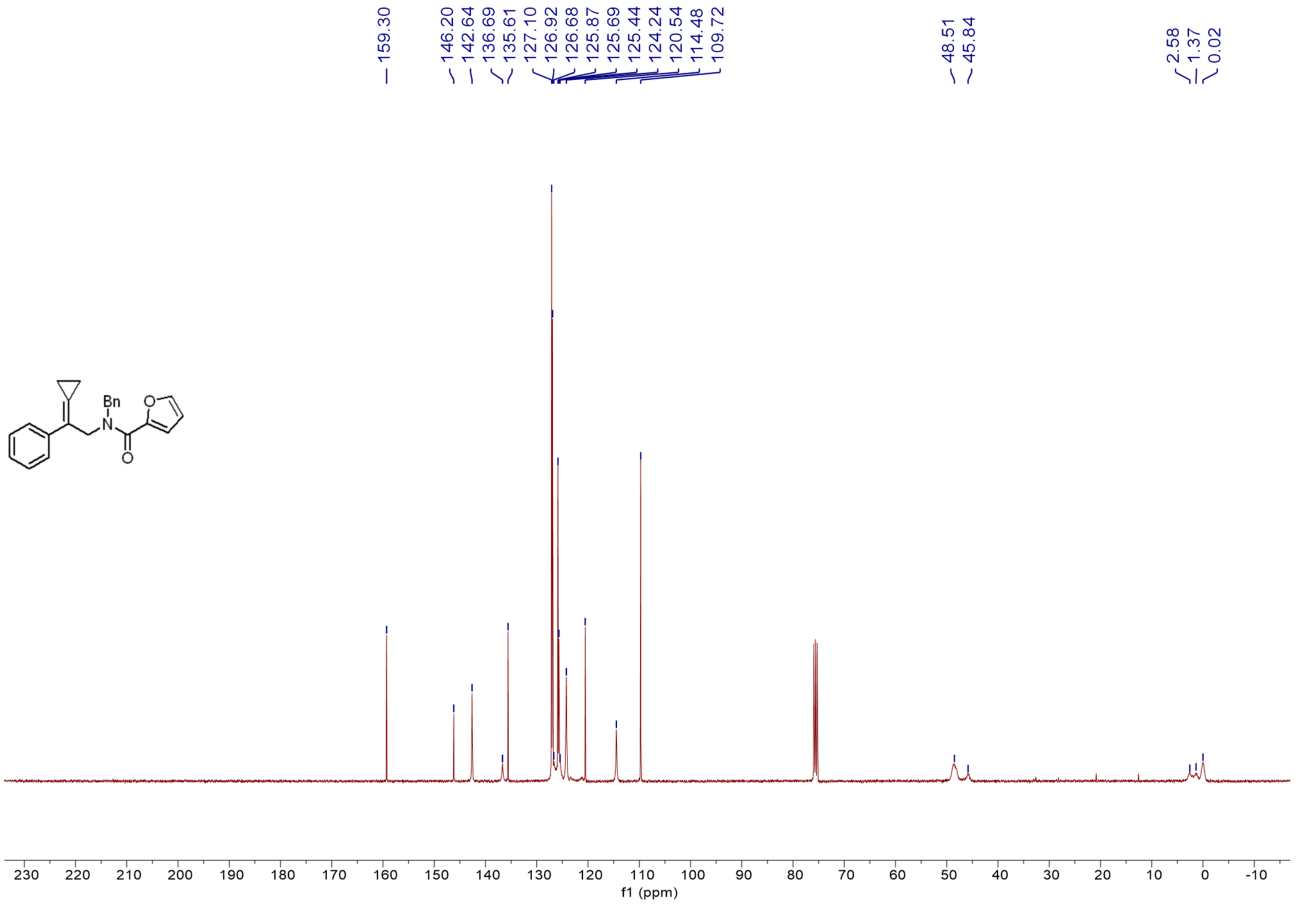
159.30
146.20
142.64
136.69
135.61
127.10
126.92
126.68
125.87
125.69
125.44
124.24
120.54
114.48
109.72
48.51
45.84
2.58
1.37
0.02
Bn
N
O
O
f1 (ppm)

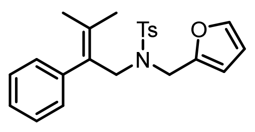

Compound 1ac: Yield: 290.0 mg, 77%; A colorless oil; ^{1}H NMR (400 MHz, $CDCl_3$) δ 7.35 (d, *J* = 8.0 Hz, 2H), 7.25 - 7.17 (m, 4H), 7.09 (d, *J* = 8.0 Hz, 2H), 6.96 - 6.87 (m, 2H), 6.25 (dd, *J* = 3.2, 1.8 Hz, 1H), 6.09 (d, *J* = 3.2 Hz, 1H), 4.22 (s, 2H), 4.17 (s, 2H), 2.37 (s, 3H), 1.79 (s, 3H), 1.54 (s, 3H); ^{13}C NMR (100 MHz, $CDCl_3$) δ 150.4, 142.6, 142.0, 140.8, 137.2, 134.8, 129.4, 129.2, 129.1, 128.0, 127.3, 126.4, 110.4, 109.0, 48.4, 42.5, 22.7, 21.5, 20.2; IR (neat): ν 1591, 1493, 1438, 1330, 1156, 1092, 1010, 927, 893, 758 cm^{-1}; HRMS (ESI) Calcd. for $C_{23}H_{26}NSO_3$ $[M+H]^+$: 396.1628, Found: 396.1631.

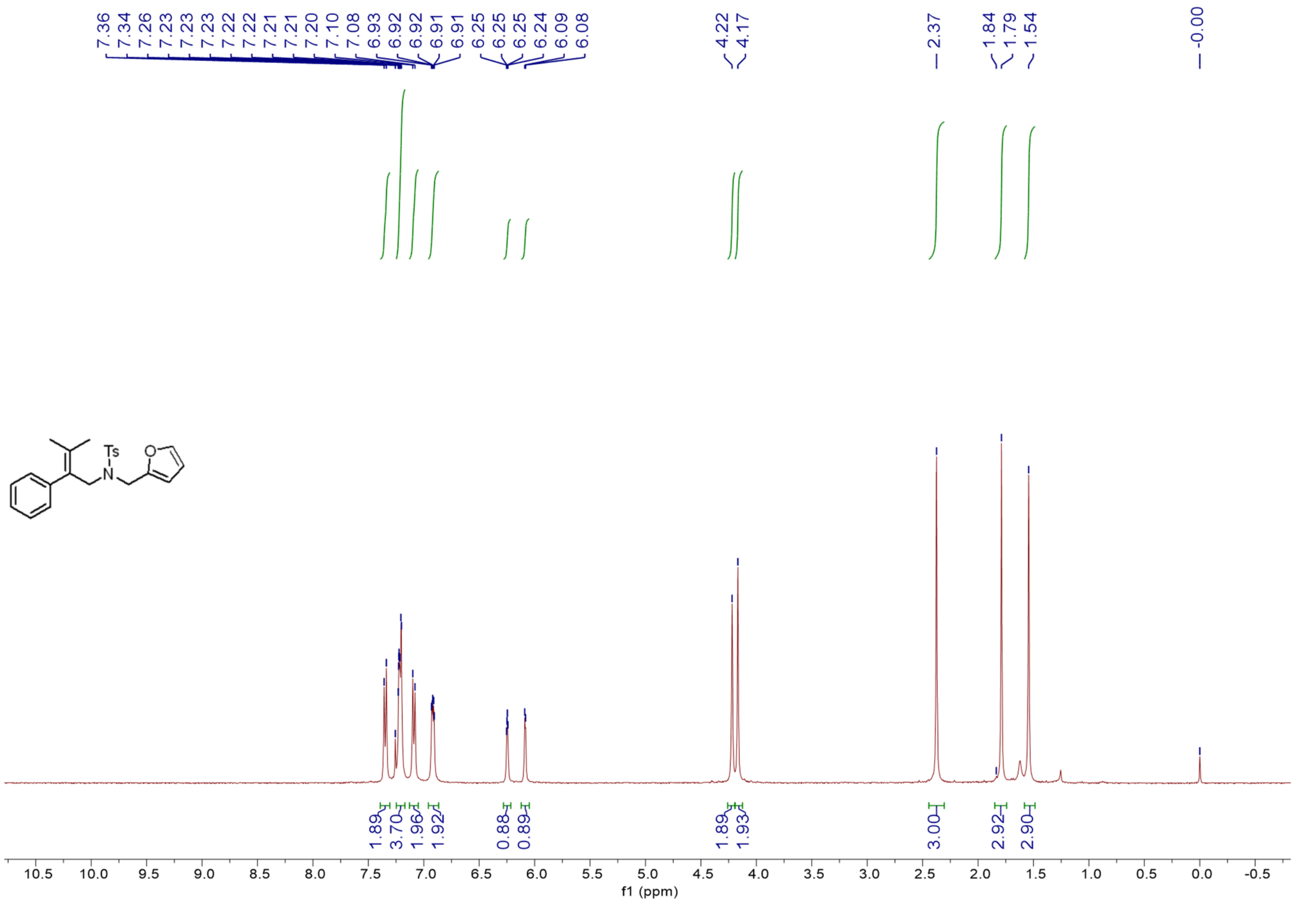
7.36
7.34
7.26
7.23
7.23
7.23
7.22
7.22
7.21
7.21
7.20
7.10
7.08
6.93
6.92
6.92
6.91
6.91
6.25
6.25
6.25
6.24
6.09
6.08
4.22
4.17
2.37
1.84
1.79
1.54
-0.00
Ts
N
O
1.89
3.70
1.96
1.92
0.88
0.89
1.89
1.93
3.00
2.92
2.90
f1 (ppm)

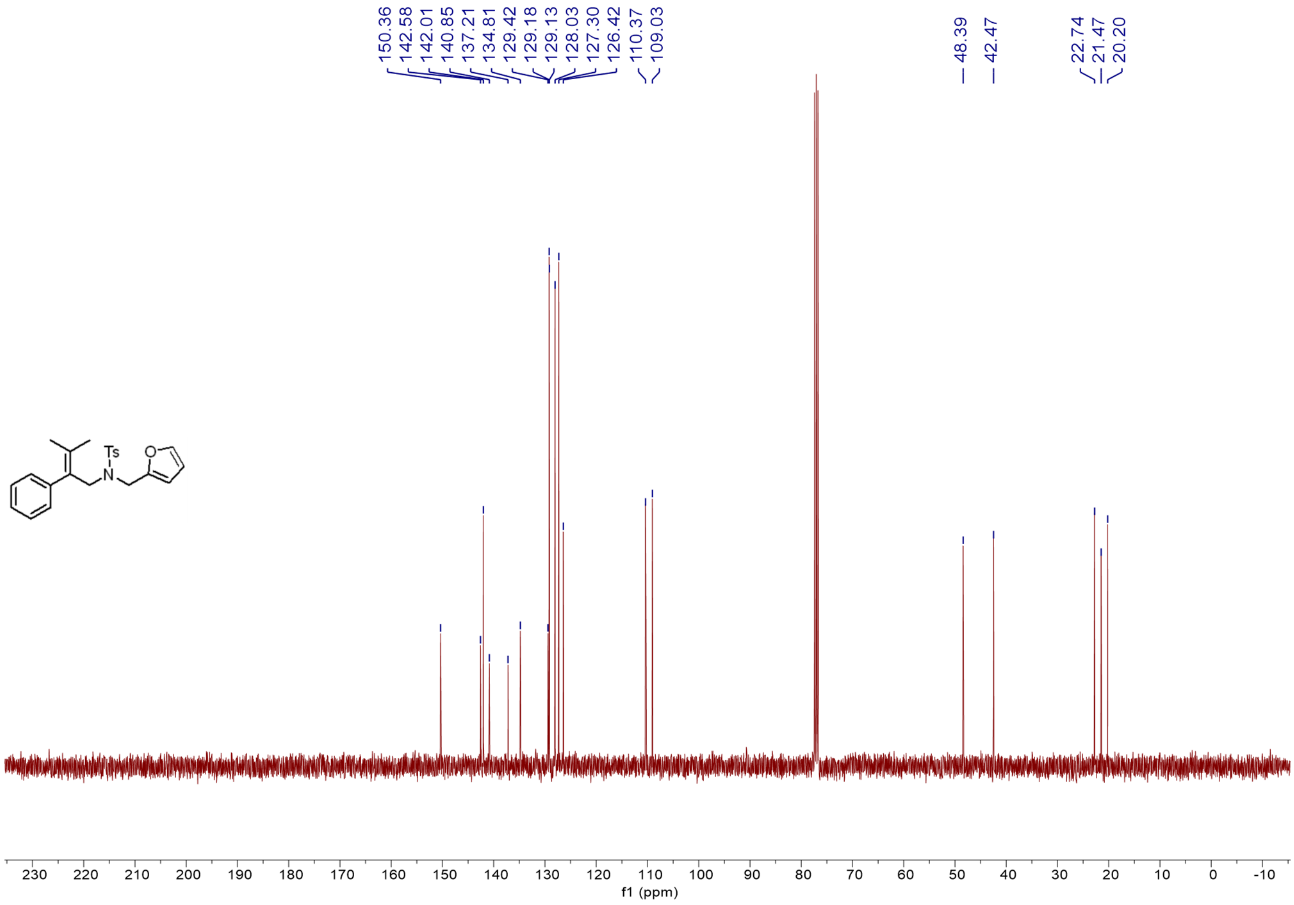
150.36
142.58
142.01
140.85
137.21
134.81
129.42
129.18
129.13
128.03
127.30
126.42
110.37
109.03
48.39
42.47
22.74
21.47
20.20
Ts
N
O
f1 (ppm)

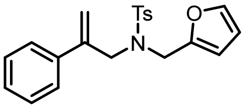

Compound 1ad: Yield: 290.0 mg, 79%; A white solid; This is a known compound and its spectroscopic data are consistent with those reported in the literature;[1] ^{1}H NMR (400 MHz, $CDCl_3$) δ 7.61 - 7.55 (m, 2H), 7.39 - 7.34 (m, 2H), 7.33 - 7.26 (m, 3H), 7.23 - 7.15 (m, 3H), 6.20 (s, 1H), 6.05 (s, 1H), 5.46 (s, 1H), 5.27 (s, 1H), 4.34 (s, 2H), 4.23 (s, 2H), 2.40 (s, 3H); ^{13}C NMR (100 MHz, $CDCl_3$) δ 149.6, 143.0, 142.4 142.2, 138.5, 136.8, 129.3, 128.3, 127.9, 127.4, 126.4, 116.3, 110.2, 109.6, 50.7, 42.6, 21.4.

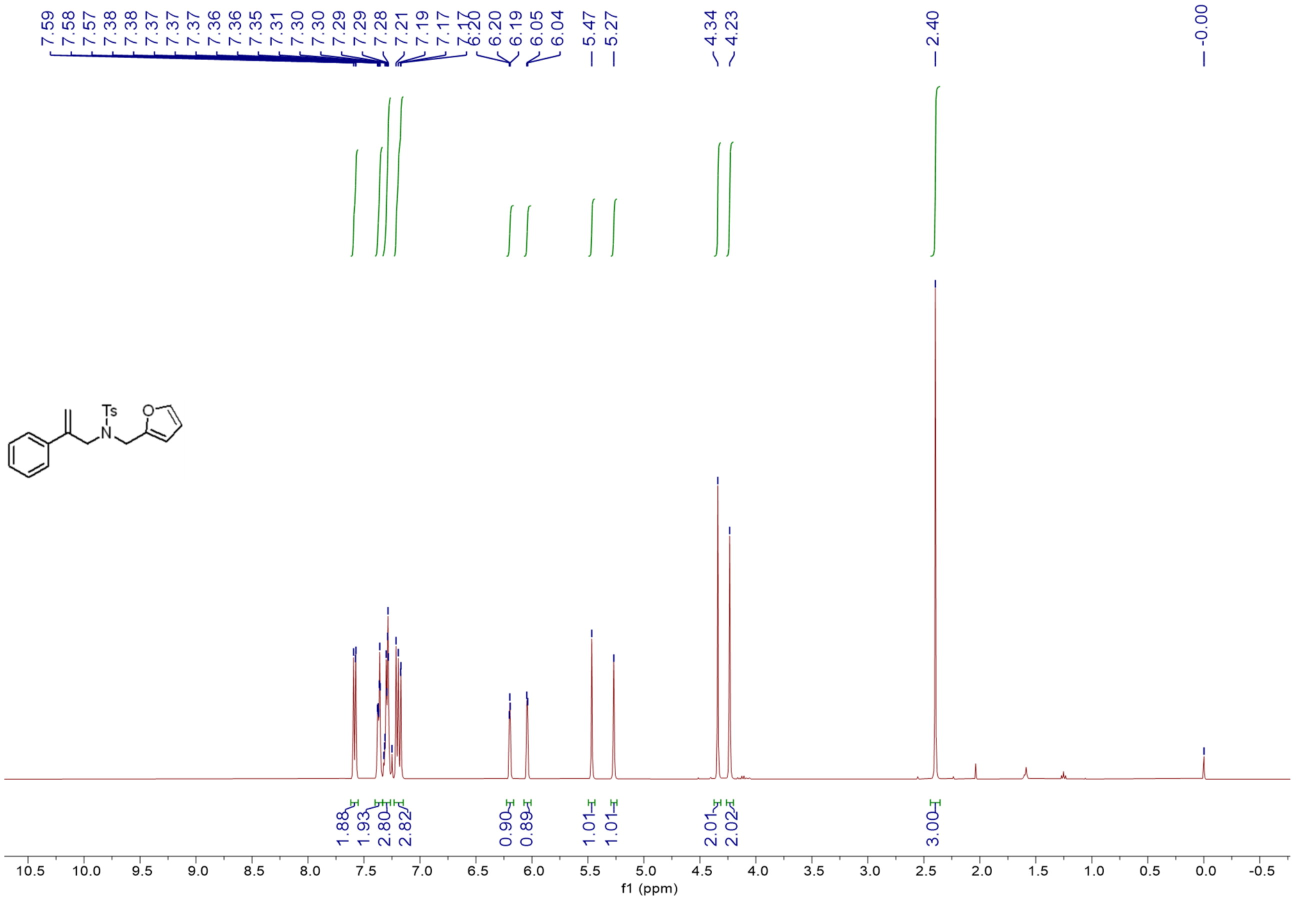

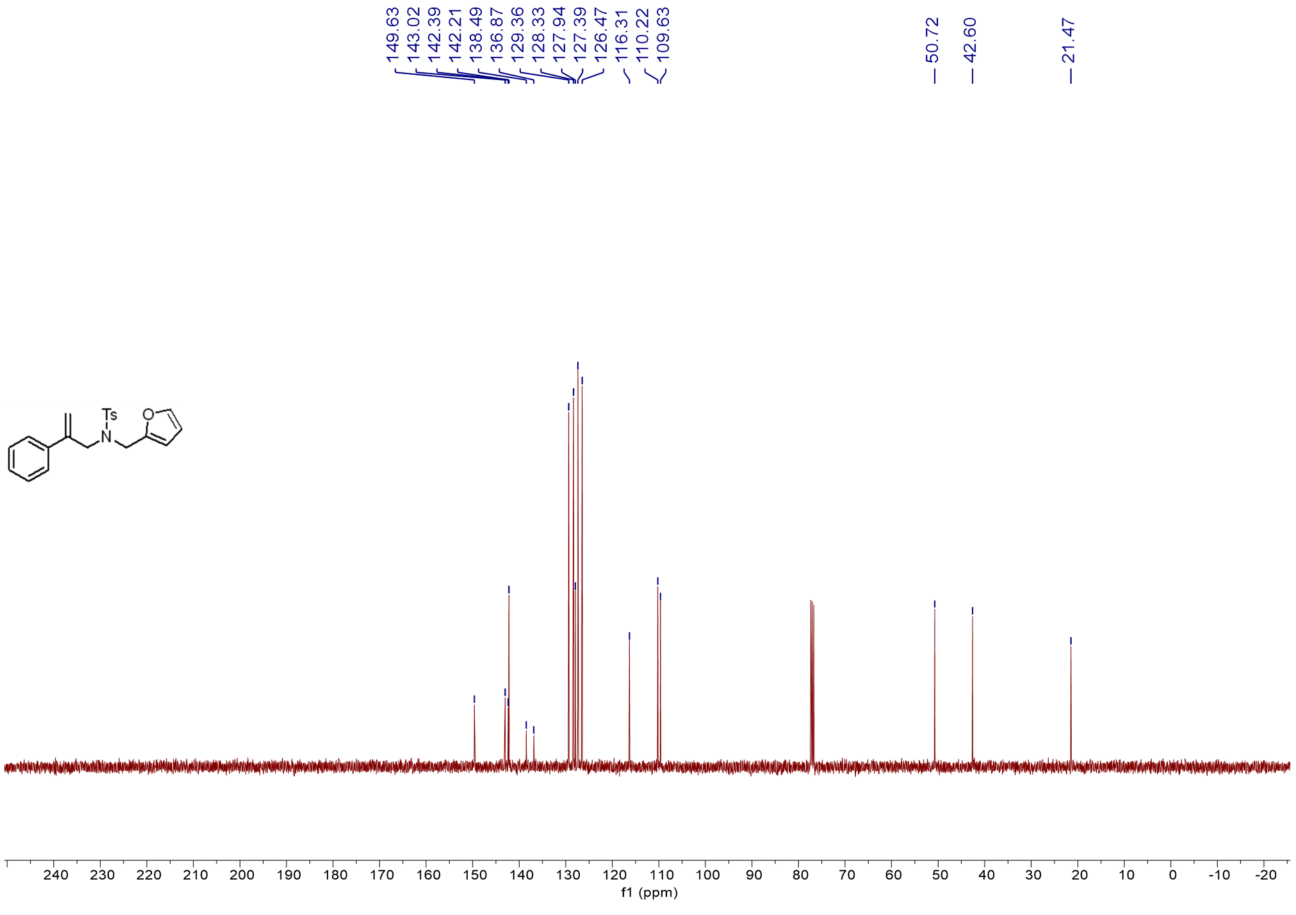

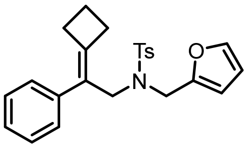

Compound 1ae: Yield: 177.0 mg, 43%; A yellow oil; ^{1}H NMR (400 MHz, $CDCl_3$) δ 7.52 - 7.46 (m, 2H), 7.29 - 7.10 (m, 8H), 6.23 (dd, *J* = 3.2, 1.8 Hz, 1H), 6.03 (dd, *J* = 3.2, 0.8 Hz, 1H), 4.21 (s, 2H), 4.15 (s, 2H), 2.81 - 2.74 (m, 2H), 2.74 - 2.67 (m, 2H), 2.39 (s, 3H), 1.99 - 1.87 (m, 2H); ^{13}C NMR (100 MHz, $CDCl_3$) δ 150.3, 145.1, 142.8, 141.9, 137.3, 137.0, 129.3, 128.0, 127.6, 127.4, 126.5, 125.1, 110.3, 108.9, 46.2, 42.2, 32.0, 30.5, 21.5, 16.8; IR (neat): ν 2954, 1600, 1493, 1334, 1158, 1096, 1015, 813, 759, 652 cm^{-1}; HRMS (ESI) Calcd. for $C_{18}H_{22}NO_3S$ $[M+H]^+$: 332.1315, Found: 332.1318.

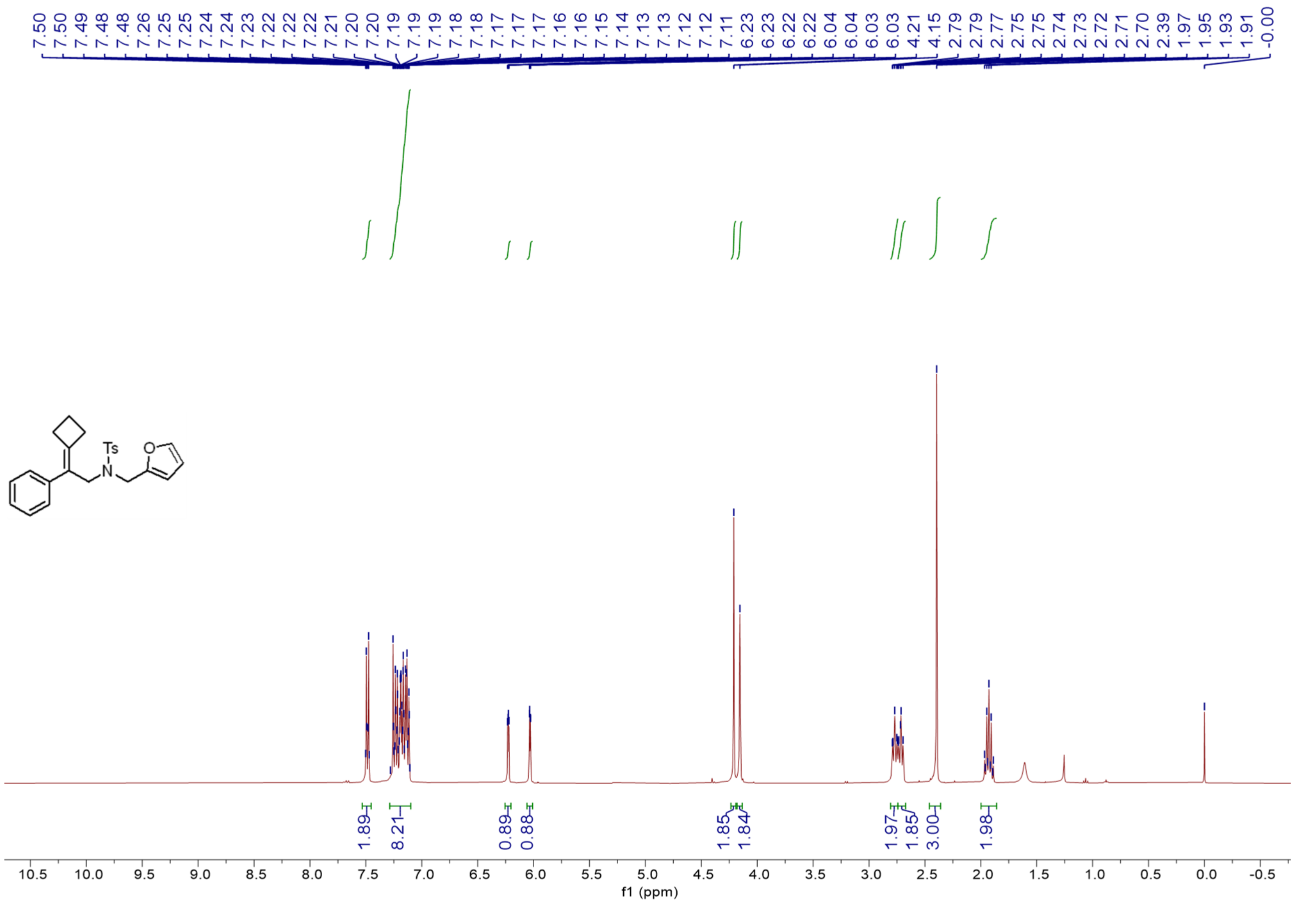
7.50 7.50 7.49 7.48 7.26 7.25 7.25 7.24 7.24 7.23 7.22 7.22 7.22 7.21 7.20 7.19 7.19 7.19 7.18 7.17 7.17 7.17 7.16 7.16 7.15 7.14 7.13 7.13 7.12 7.12 7.11 6.23 6.23 6.22 6.22 6.04 6.04 6.03 6.03 4.21 4.15 2.79 2.79 2.77 2.75 2.75 2.74 2.73 2.72 2.71 2.70 2.39 1.97 1.95 1.93 1.91 -0.00
Ts
O
N
1.89
8.21
0.89
0.88
1.85
1.84
1.97
1.85
3.00
1.98
10.5 10.0 9.5 9.0 8.5 8.0 7.5 7.0 6.5 6.0 5.5 5.0 4.5 4.0 3.5 3.0 2.5 2.0 1.5 1.0 0.5 0.0 -0.5
f1 (ppm)

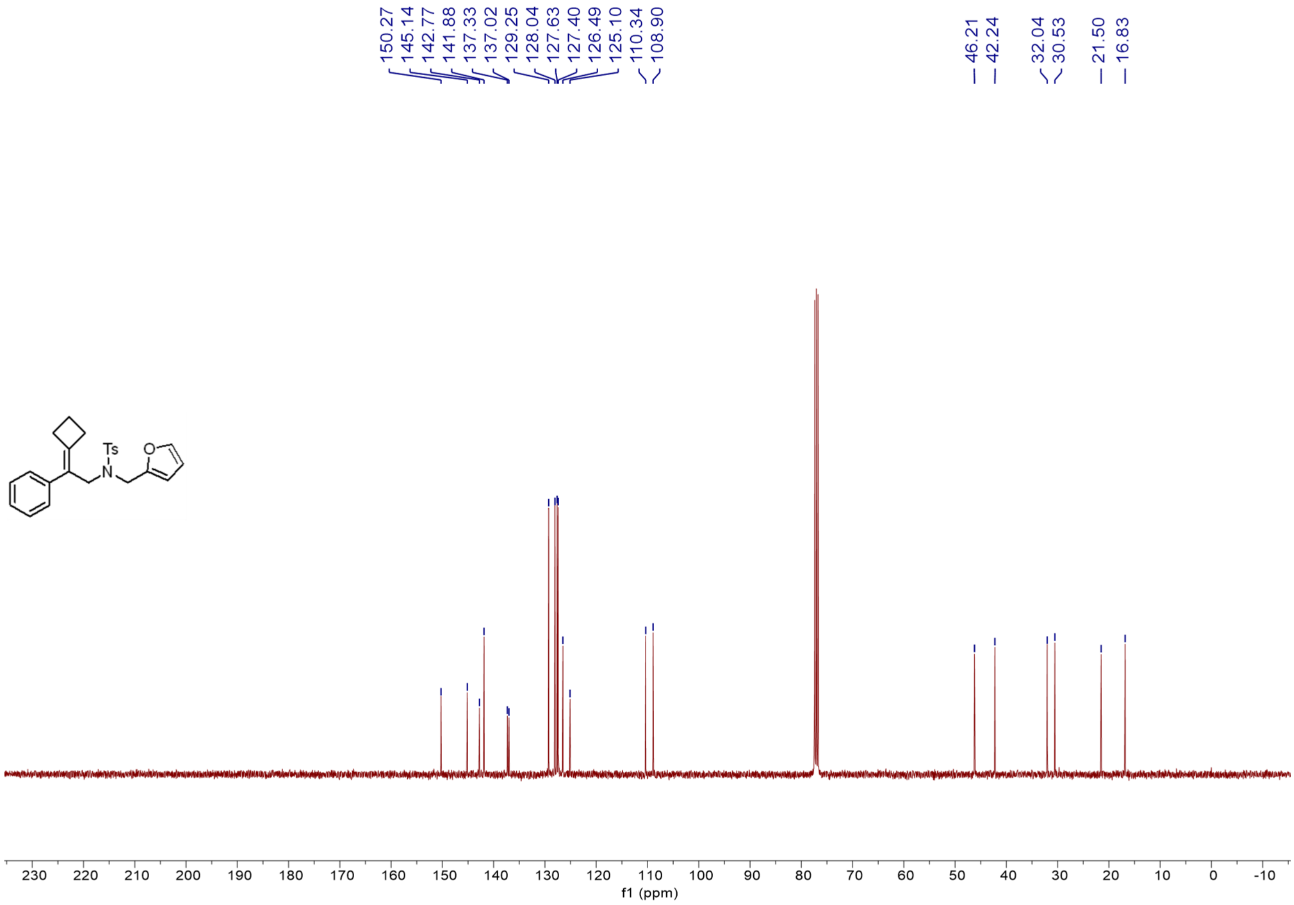
150.27 145.14 142.77 141.88 137.33 137.02 129.25 128.04 127.63 127.40 126.49 125.10 110.34 108.90
46.21 42.24 32.04 30.53 21.50 16.83
Ts
O
N
230 220 210 200 190 180 170 160 150 140 130 120 110 100 90 80 70 60 50 40 30 20 10 0 -10
f1 (ppm)

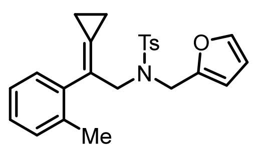

Compound 1af: Yield: 774.0 mg, 63%; A colorless oil; ^{1}H NMR (400 MHz, $CDCl_3$) δ 7.45 (d, J = 8.2 Hz, 2H), 7.23 -7.06 (m, 6H), 7.02 (dd, J = 7.6, 1.8 Hz, 1H), 6.23 (dd, J = 3.2, 1.8 Hz, 1H), 6.06 (d, J = 3.2 Hz, 1H), 4.35 (s, 2H), 4.25 (s, 2H), 2.37 (s, 3H), 2.19 (s, 3H), 1.30 - 1.24 (m, 2H), 1.06 - 0.99 (m, 2H); ^{13}C NMR (100 MHz, $CDCl_3$) δ 150.1, 142.6, 142.1, 139.1, 137.5, 135.9, 130.2, 129.2, 128.9, 127.14, 127.11, 126.2, 125.5, 124.0, 110.3, 109.2, 51.2, 42.9, 21.4, 19.8, 3.1, 2.9; IR (neat): ν 2976, 1494, 1444, 1346, 1158, 1010, 919, 813, 727 cm^{-1}; HRMS (ESI) Calcd. for $C_{24}H_{25}NO_3SNa$ $[M+Na]^+$: 430.1447, Found: 430.1450.

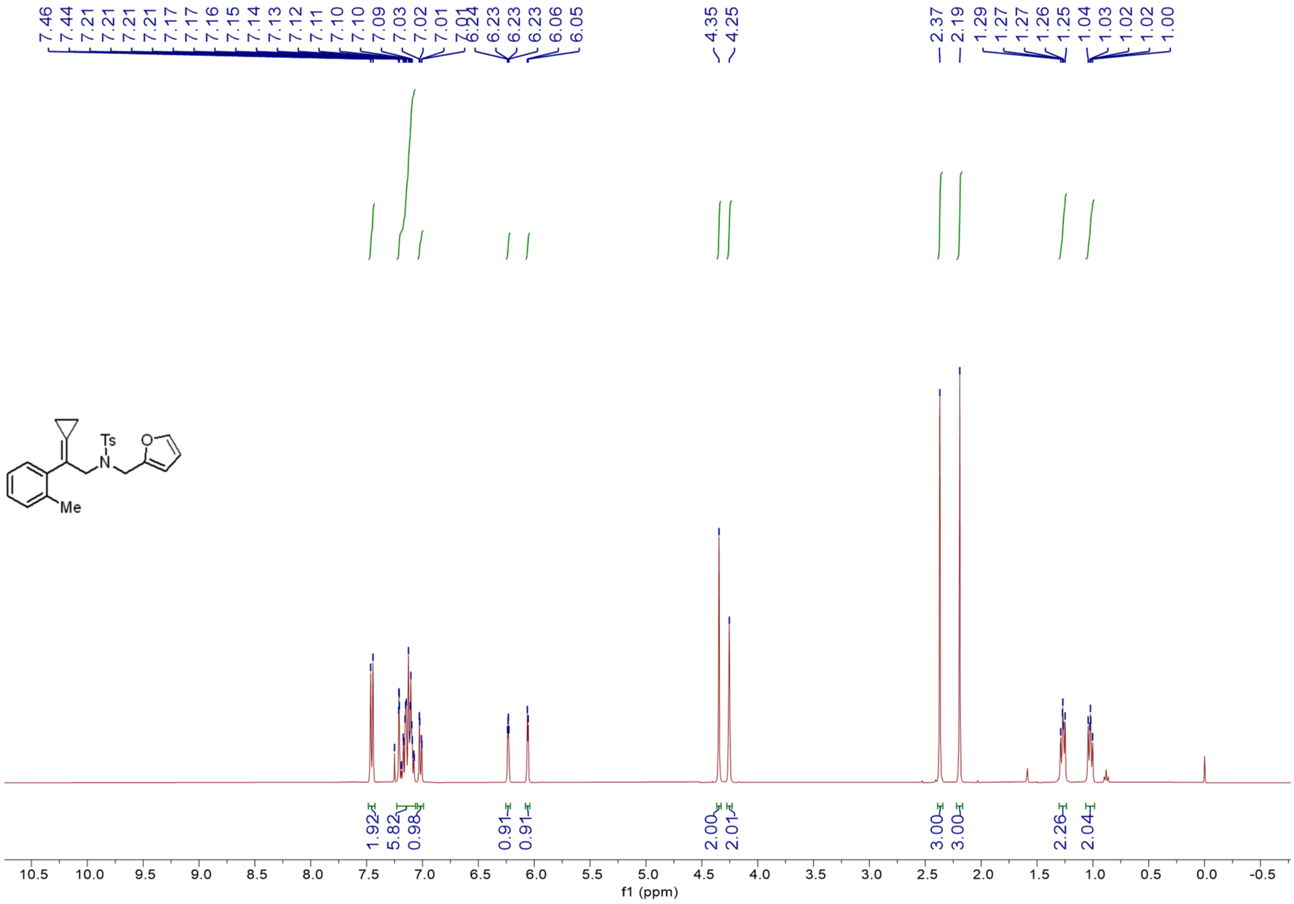

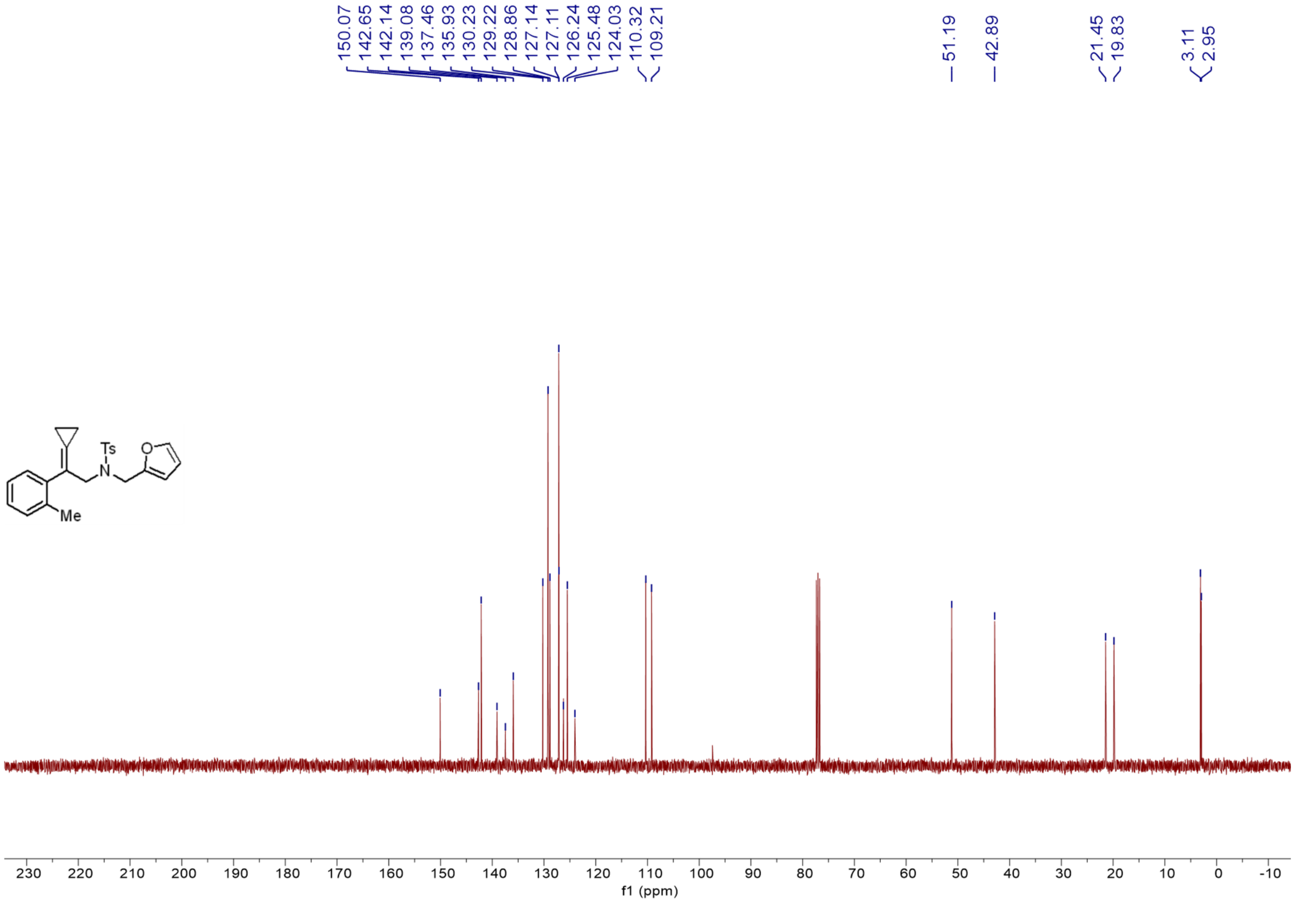

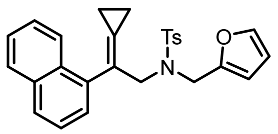

Compound 1ag: Yield: 984.0 mg, 74%; A white solid; M.p.: 128 - 130 ºC; ^{1}H NMR (400 MHz, $CDCl_3$) δ 7.82 (d, *J* = 7.7 Hz, 1H), 7.74 (d, *J* = 8.0 Hz, 2H), 7.49 - 7.30 (m, 5H), 7.25 - 7.18 (m, 2H), 6.97 (d, *J* = 8.0 Hz, 2H), 6.23 (dt, *J* = 3.2, 1.6 Hz, 1H), 6.05 (d, *J* = 3.2 Hz, 1H), 4.40 (s, 4H), 2.31 (s, 3H), 1.45 - 1.33 (m, 2H), 1.03 - 0.95 (m, 2H); ^{13}C NMR (100 MHz, $CDCl_3$) δ150.1, 142.5, 142.2, 137.4, 137.3, 133.8, 131.4, 129.0, 128.4, 128.0, 127.4, 127.0, 125.9, 125.7, 125.5, 125.4, 125.2, 122.7, 110.3, 109.3, 51.7, 43.0, 21.4, 3.5, 3.2; IR (neat): ν 2979, 1597, 1505, 1339, 1157, 1091, 1011, 804, 781, 736 cm^{-1}; HRMS (ESI) Calcd. for $C_{27}H_{25}NO_3S$ $[M+Na]^+$: 466.1447, Found: 466.1445.

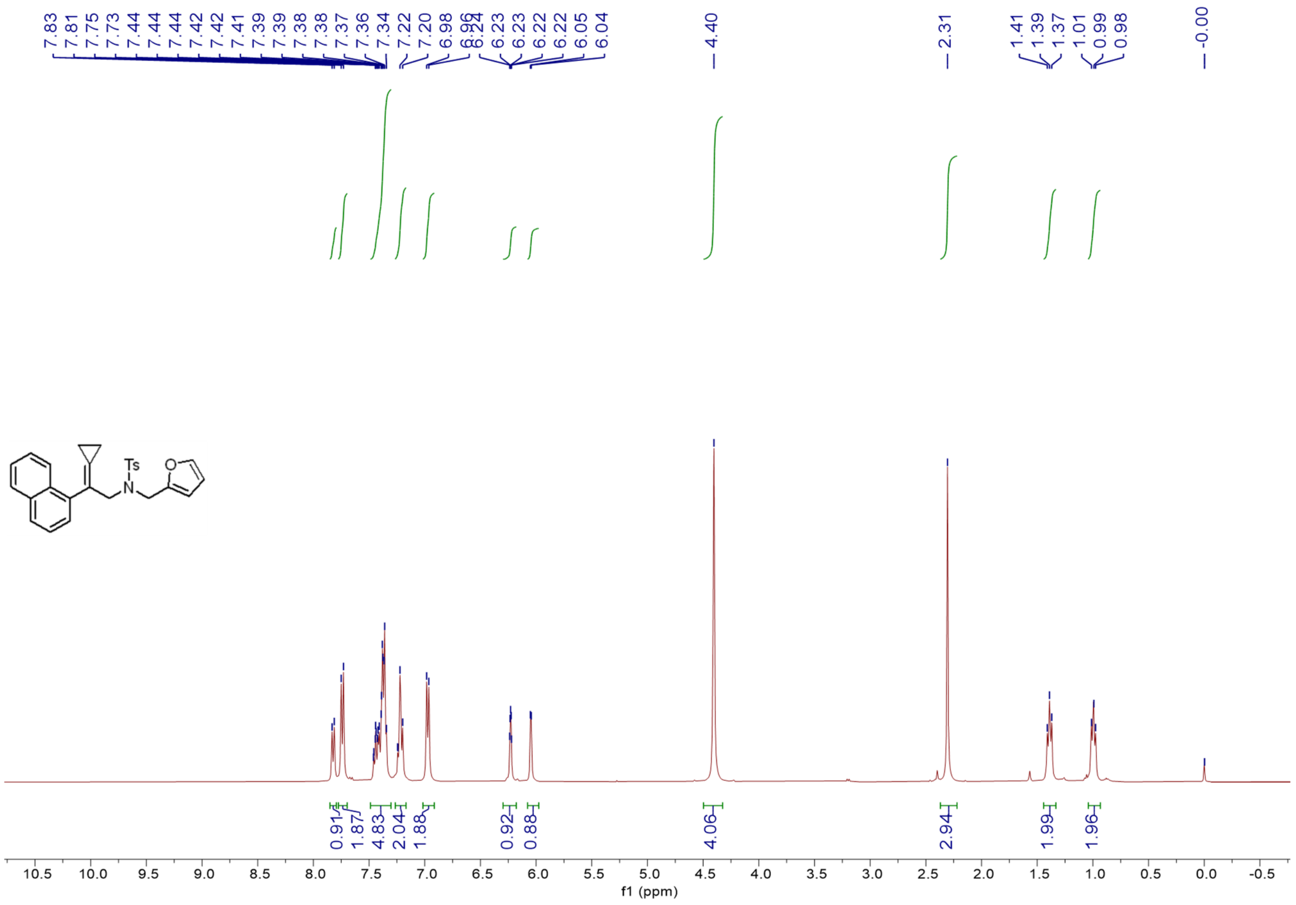
7.83
7.81
7.75
7.73
7.44
7.44
7.44
7.42
7.42
7.41
7.39
7.39
7.38
7.38
7.37
7.36
7.34
7.22
7.20
6.98
6.96
6.24
6.23
6.23
6.22
6.22
6.05
6.04
4.40
2.31
1.41
1.39
1.37
1.01
0.99
0.98
-0.00
Ts
N
O
0.91
1.87
4.83
2.04
1.88
0.92
0.88
4.06
2.94
1.99
1.96
f1 (ppm)

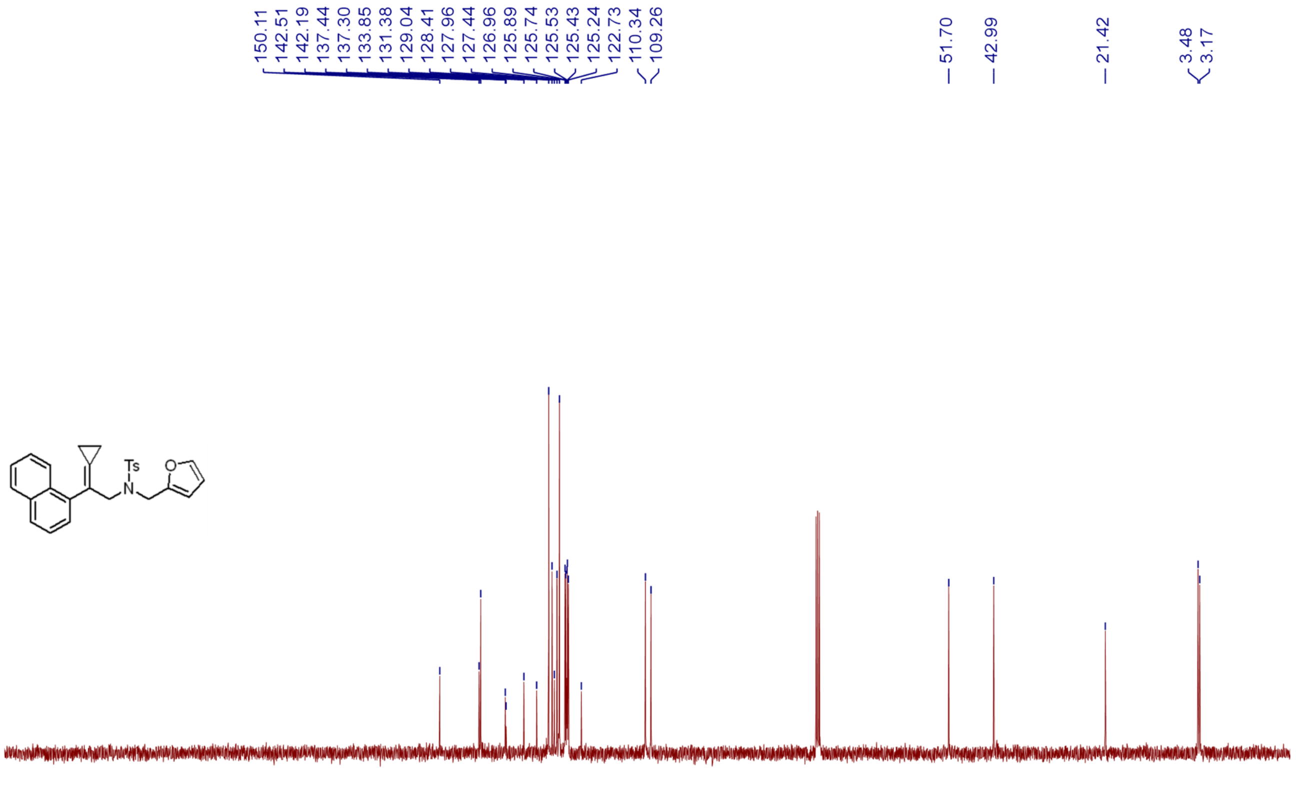
150.11
142.51
142.19
137.44
137.30
133.85
131.38
129.04
128.41
127.96
127.44
126.96
125.89
125.74
125.53
125.43
125.24
122.73
110.34
109.26
51.70
42.99
21.42
3.48
3.17
Ts
N
O
f1 (ppm)

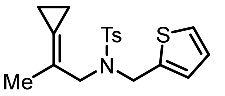

Compound 1ah: Yield: 750 mg, 72%; A white solid; M.p.: 58 - 60 °C; ^{1}H NMR (400 MHz, $CDCl_3$) δ 7.65 (d, J = 8.2 Hz, 2H), 7.25 (d, J = 8.2 Hz, 2H), 7.13 (dt, J = 5.2, 1.2 Hz, 1H), 6.85 (dd, J = 5.2, 3.4 Hz, 1H), 6.78 (d, J = 3.4 Hz, 1H), 4.48 (s, 2H), 3.92 (s, 2H), 2.41 (s, 3H), 1.71 (s, 3H), 0.98 (s, 4H); ^{13}C NMR (100 MHz, $CDCl_3$) δ 143.0, 139.0, 137.5, 129.5, 127.3, 127.2, 126.4, 125.6, 122.1, 119.3, 52.5, 44.9, 21.5, 18.3, 2.9, 2.0; IR (neat): ν 2975, 1598, 1442, 1333, 1143, 1091, 1022, 903, 814, 704 cm^{-1}; HRMS (ESI) Calcd. for $C_{18}H_{21}NO_2S_2Na$ $[M+Na]^+$: 370.0906, Found: 470.0904.

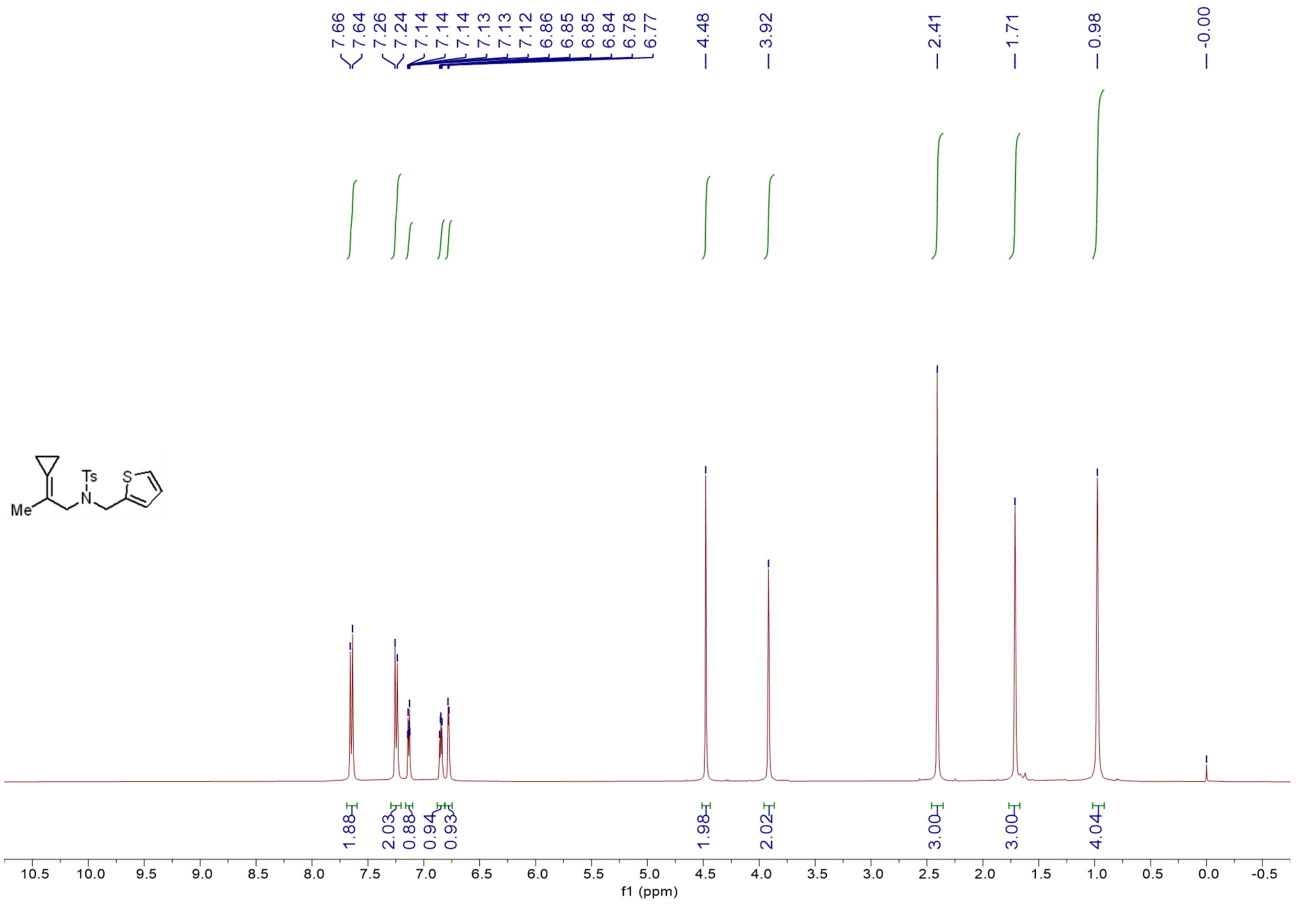

7.66
7.64
7.26
7.24
7.14
7.14
7.14
7.13
7.13
7.12
6.86
6.85
6.85
6.84
6.78
6.77
4.48
3.92
2.41
1.71
0.98
-0.00
Ts
N
S
Me
1.88
2.03
0.88
0.94
0.93
1.98
2.02
3.00
3.00
4.04
f1 (ppm)

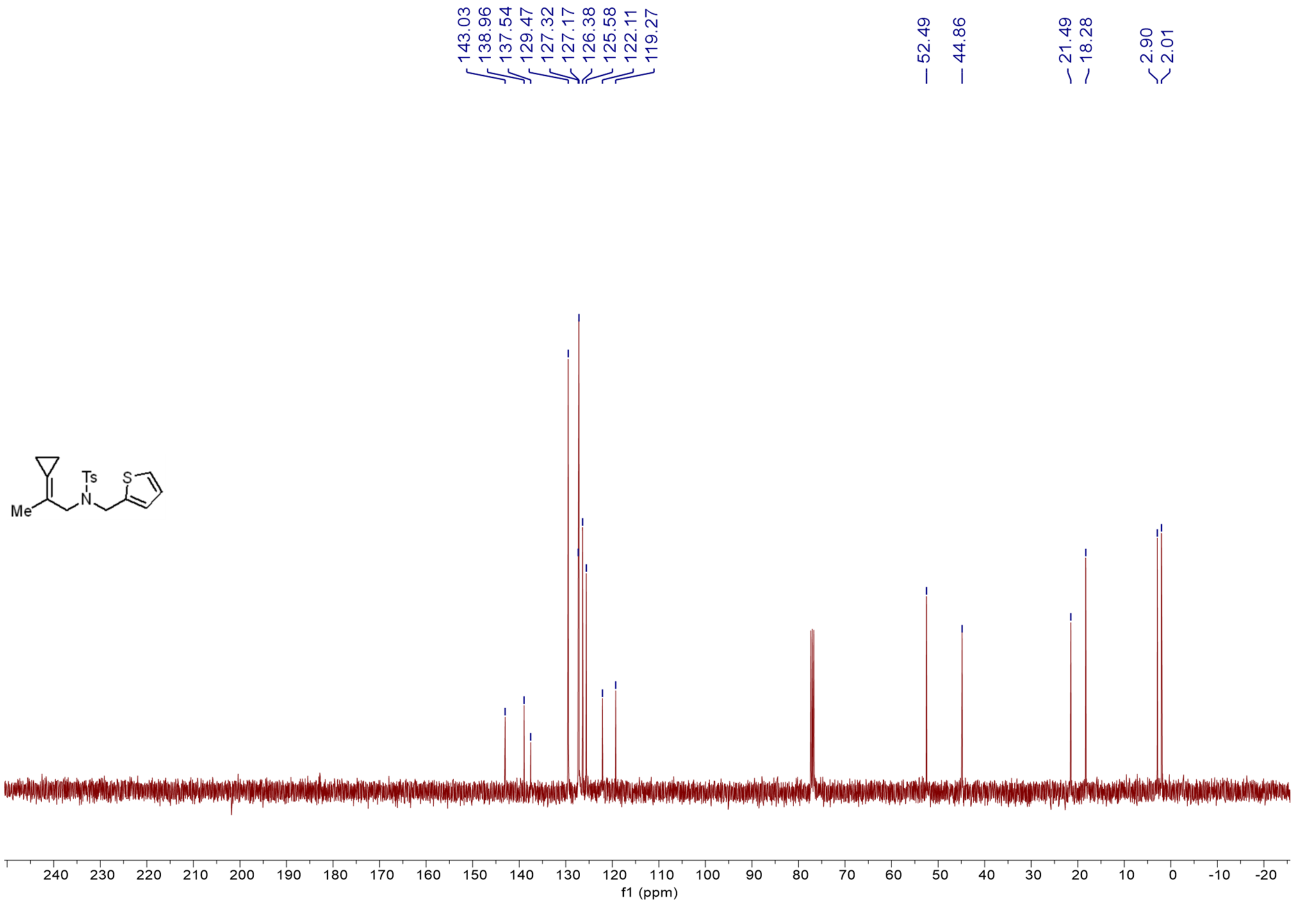

143.03
138.96
137.54
129.47
127.32
127.17
126.38
125.58
122.11
119.27
52.49
44.86
21.49
18.28
2.90
2.01
Ts
N
S
Me
f1 (ppm)

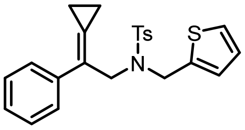

Compound 1ai: Yield: 860 mg, 70%; A white solid; M.p.: 127 - 130 °C; ^{1}H NMR (400 MHz, $CDCl_3$) δ 7.64 - 7.59 (m, 2H), 7.54 - 7.48 (m, 2H), 7.32 - 7.19 (m, 5H), 7.07 (dd, *J* = 5.0, 1.2 Hz, 1H), 6.79 (dd, *J* = 5.0, 3.4 Hz, 1H), 6.67 (dd, *J* = 3.4, 1.2 Hz, 1H), 4.46 (s, 2H), 4.42 (s, 2H), 2.43 (s, 3H), 1.57 (s, 6H), 1.38 - 1.31 (m, 2H), 1.06 - 0.98 (m, 2H); ^{13}C NMR (100 MHz, $CDCl_3$) δ 143.0, 139.0, 137.6, 129.5, 127.3, 127.2, 126.4, 125.5, 122.1, 119.3, 52.5, 44.9, 21.5, 18.3, 2.9, 2.0; IR (neat): ν 2970, 1597, 1336, 1158, 1091, 1023, 902, 815, 750, 696 cm^{-1}; HRMS (ESI) Calcd. for $C_{23}H_{23}NO_2S_2Na$ $[M+Na]^+$: 432.1062, Found: 432.1065.

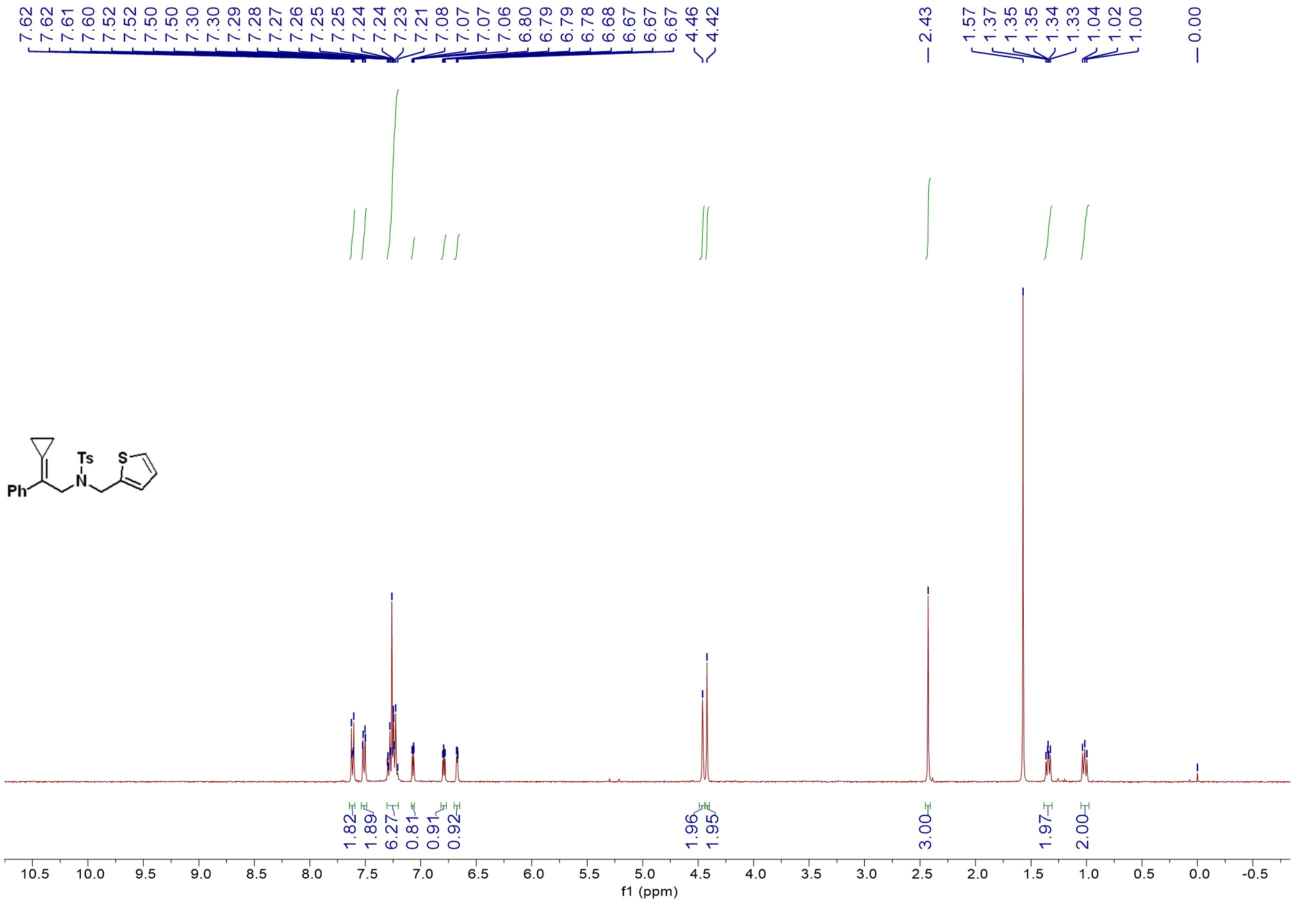
7.62 7.62 7.60 7.52 7.52 7.50 7.30 7.30 7.29 7.28 7.27 7.26 7.25 7.24 7.24 7.23 7.21 7.08 7.07 7.06 6.80 6.79 6.79 6.78 6.68 6.67 6.67 6.67 4.46 4.42
2.43 1.57 1.37 1.35 1.35 1.34 1.33 1.04 1.02 1.00 0.00
Ts
N
S
Ph
1.82 1.89 6.27 0.81 0.91 0.92 1.96 1.95 3.00 1.97 2.00
f1 (ppm)

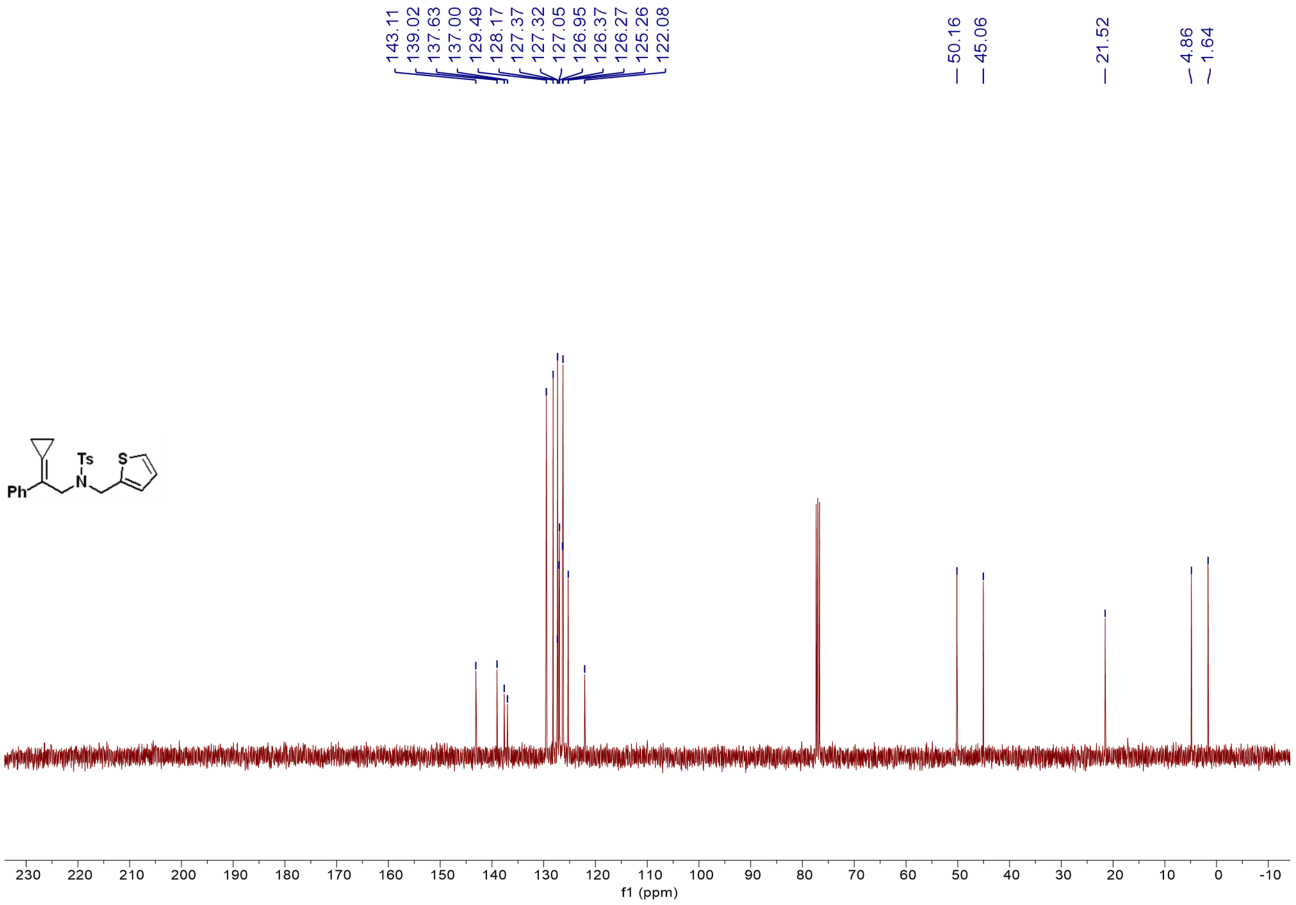
143.11 139.02 137.63 137.00 129.49 128.17 127.37 127.32 127.05 126.95 126.37 126.27 125.26 122.08
50.16 45.06 21.52 4.86 1.64
Ts
N
S
Ph
f1 (ppm)

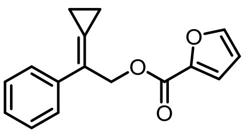

Compound 1aj: Yield: 475.0 mg, 62%; A white solid; M.p.: 95 - 98 °C; ^{1}H NMR (400 MHz, $CDCl_3$) δ 7.68 (d, *J* = 7.6 Hz, 2H), 7.58 - 7.51 (m, 1H), 7.36 (t, *J* = 7.6 Hz, 2H), 7.30 - 7.20 (m, 1H), 7.12 (d, *J* = 3.4 Hz, 1H), 6.45 (dd, *J* = 3.4, 1.8 Hz, 1H), 5.38 (s, 2H), 1.52 (t, *J* = 9.6 Hz, 2H), 1.30 (t, *J* = 9.6 Hz, 2H); ^{13}C NMR (100 MHz, $CDCl_3$) δ 158.7, 146.3, 144.6, 137.5, 128.4, 128.3, 127.1, 125.7, 122.1, 118.0, 111.8, 66.3, 5.3, 1.2; IR (neat): ν 2975, 1721, 1497, 1473, 1396, 1293, 1178, 1115, 761, 697 cm^{-1}; HRMS (ESI) Calcd. for $C_{16}H_{14}NO_3Na$ $[M+Na]^+$: 277.0835, Found: 277.0831.

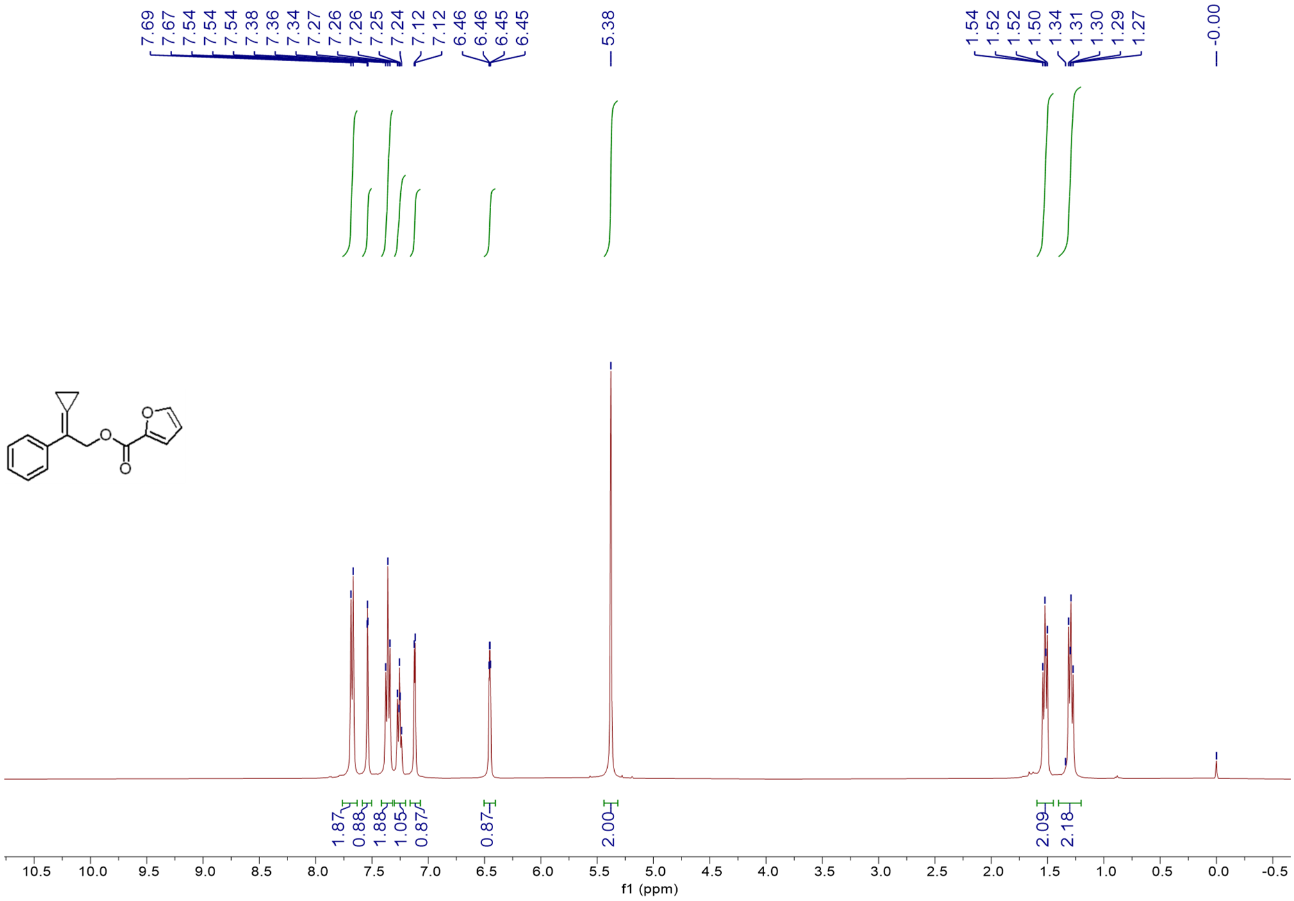
7.69 7.67 7.54 7.54 7.54 7.38 7.36 7.34 7.27 7.26 7.26 7.25 7.24 7.12 7.12 6.46 6.46 6.45 6.45
5.38
1.54 1.52 1.52 1.50 1.34 1.31 1.30 1.29 1.27
-0.00
1.87 0.88 1.88 1.05 0.87 0.87 2.00 2.09 2.18
O
O
O
f1 (ppm)

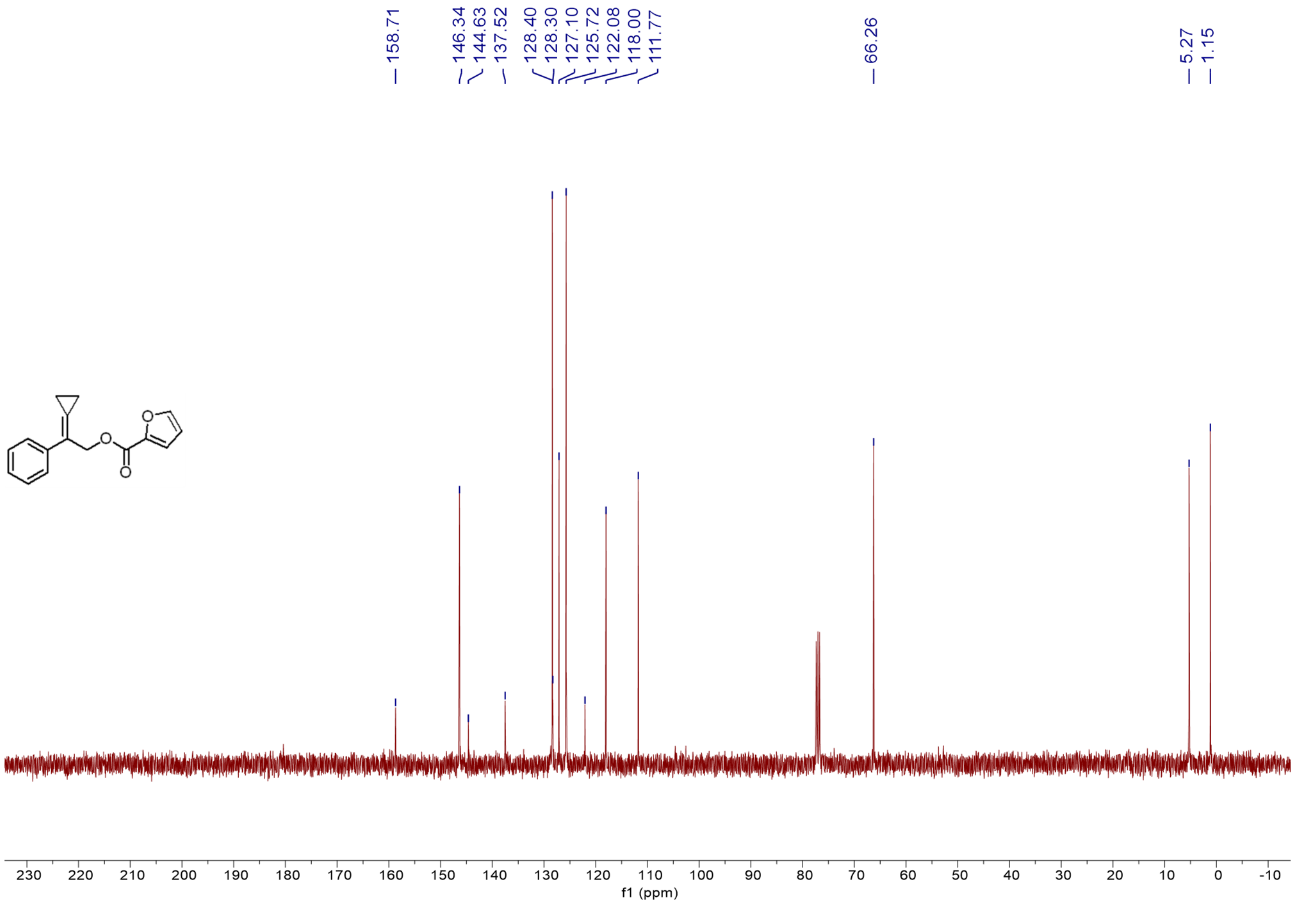
158.71
146.34 144.63 137.52
128.40 128.30 127.10 125.72 122.08 118.00 111.77
66.26
5.27 1.15
O
O
O
f1 (ppm)

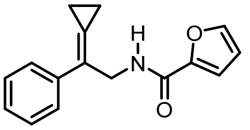

Compound 1ak: Yield: 304.0 mg, 60%; A white solid; M.p.: 100 - 103 ºC; ^{1}H NMR (400 MHz, $CDCl_3$) δ 7.69 - 7.62 (m, 2H), 7.35 (t, *J* = 7.8 Hz, 3H), 7.24 (t, *J* = 7.4 Hz, 1H), 7.10 (d, *J* = 3.4 Hz, 1H), 6.59 (s, 1H), 6.45 (dt, *J* = 3.4, 1.6 Hz, 1H), 4.65 (d, *J* = 5.6 Hz, 2H), 1.52 - 1.40 (m, 2H), 1.34 - 1.22 (m, 2H); ^{13}C NMR (100 MHz, $CDCl_3$) δ 158.2, 148.0, 143.8, 137.6, 128.5, 127.2, 125.7, 124.3, 123.3, 114.1, 112.0, 4.6, 1.1; IR (neat): ν 3301, 1652, 1594, 1526, 1497, 1747, 1014, 797, 756, 697 cm^{-1}; HRMS (ESI) Calcd. for $C_{16}H_{15}NO_2Na$ $[M+Na]^+$: 276.0995, Found: 276.0991.

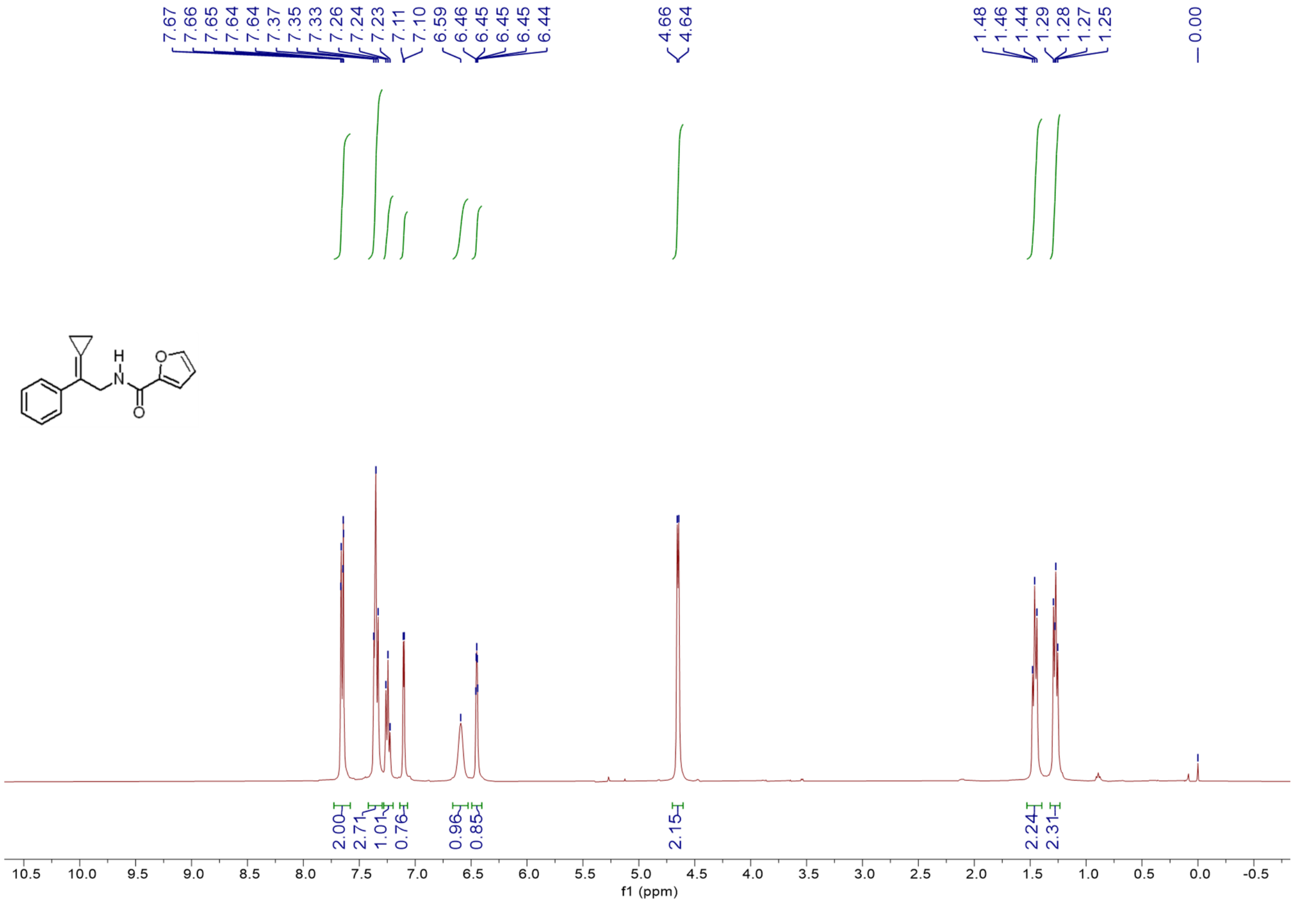
7.67
7.66
7.65
7.64
7.64
7.37
7.35
7.33
7.26
7.24
7.23
7.11
7.10
6.59
6.46
6.45
6.45
6.45
6.44
4.66
4.64
1.48
1.46
1.44
1.29
1.28
1.27
1.25
0.00
H
N
O
O
2.00
2.71
1.01
0.76
0.96
0.85
2.15
2.24
2.31
f1 (ppm)

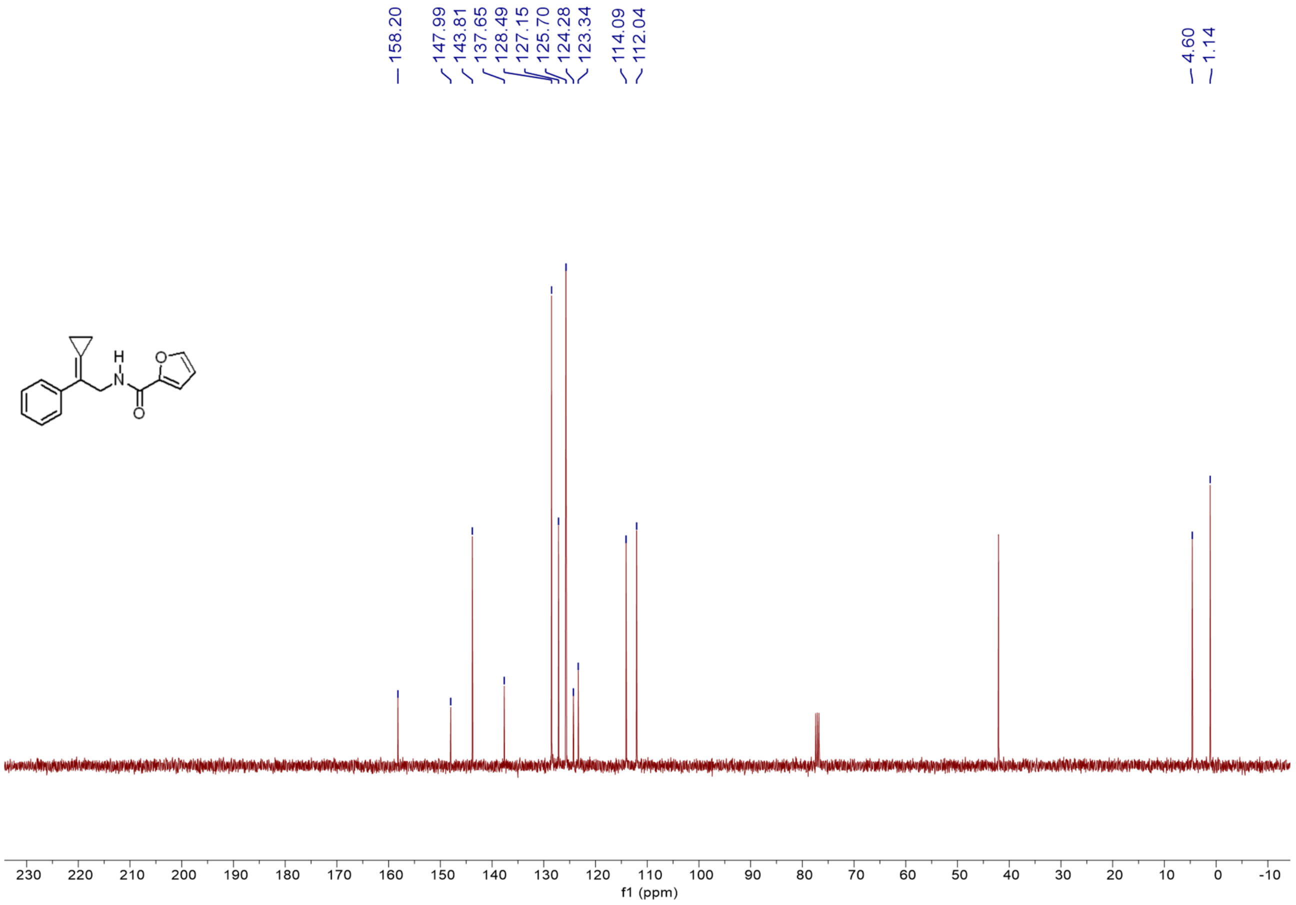
158.20
147.99
143.81
137.65
128.49
127.15
125.70
124.28
123.34
114.09
112.04
4.60
1.14
H
N
O
O
f1 (ppm)

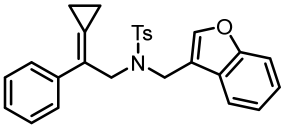

Compound 1al: Yield: 1029.0 mg, 50%; A white solid; M.p.: 146 - 148 ºC; ^{1}H NMR (400 MHz, $CDCl_3$) δ 7.61 (d, *J* = 7.0 Hz, 4H), 7.42 - 7.35 (m, 1H), 7.30 (t, *J* = 7.4 Hz, 2H), 7.24 (d, *J* = 7.4 Hz, 1H), 7.22 - 7.12 (m, 3H), 7.10 (d, *J* = 8.0 Hz, 2H), 6.27 (s, 1H), 4.55 (s, 2H), 4.37 (s, 2H), 2.31 (s, 3H), 1.35 (t, *J* = 8.0 Hz, 2H), 1.08 (t, *J* = 8.0 Hz, 2H); ^{13}C NMR (100 MHz, $CDCl_3$) δ 154.5, 152.7, 143.1, 137.4, 136.4, 129.2, 128.2, 128.0, 127.5, 127.3, 127.1, 126.3, 124.0, 122.7, 121.8, 120.8, 110.8, 105.6, 50.9, 43.1, 21.4, 5.0, 1.6; IR (neat): ν 2922, 1598, 1341, 1160, 1091, 1040, 907, 752, 709, 695 cm^{-1}; HRMS (ESI) Calcd. for $C_{27}H_{25}NO_3SNa$ $[M+Na]^+$: 466.1447, Found: 466.1453.

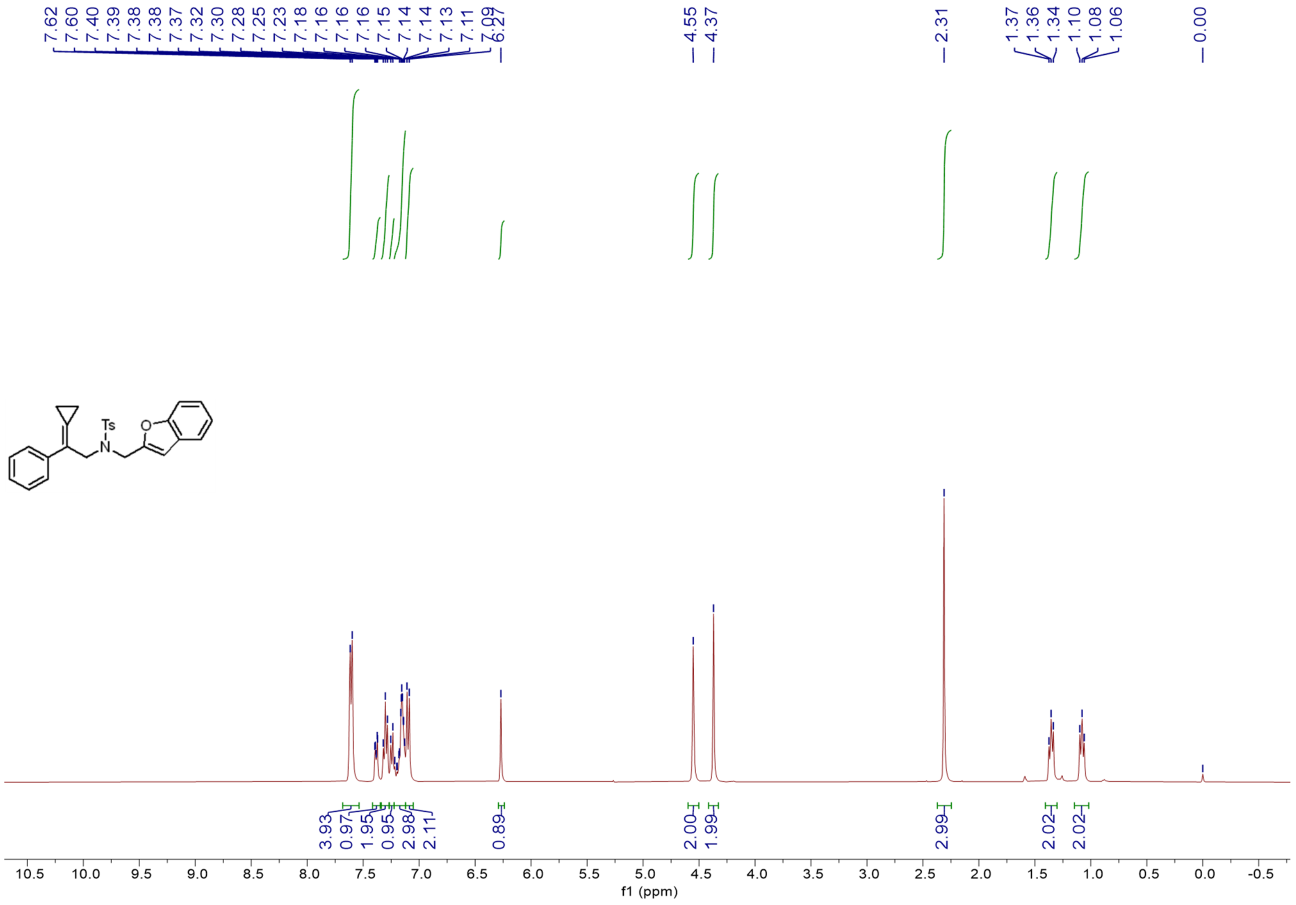
7.62 7.60 7.40 7.39 7.38 7.38 7.37 7.32 7.30 7.28 7.25 7.23 7.18 7.16 7.16 7.15 7.14 7.14 7.13 7.11 7.09 6.27
4.55 4.37
2.31
1.37 1.36 1.34 1.10 1.08 1.06
0.00
Ts
N
O
3.93 0.97 1.95 0.95 2.98 2.11
0.89
2.00 1.99
2.99
2.02 2.02
f1 (ppm)

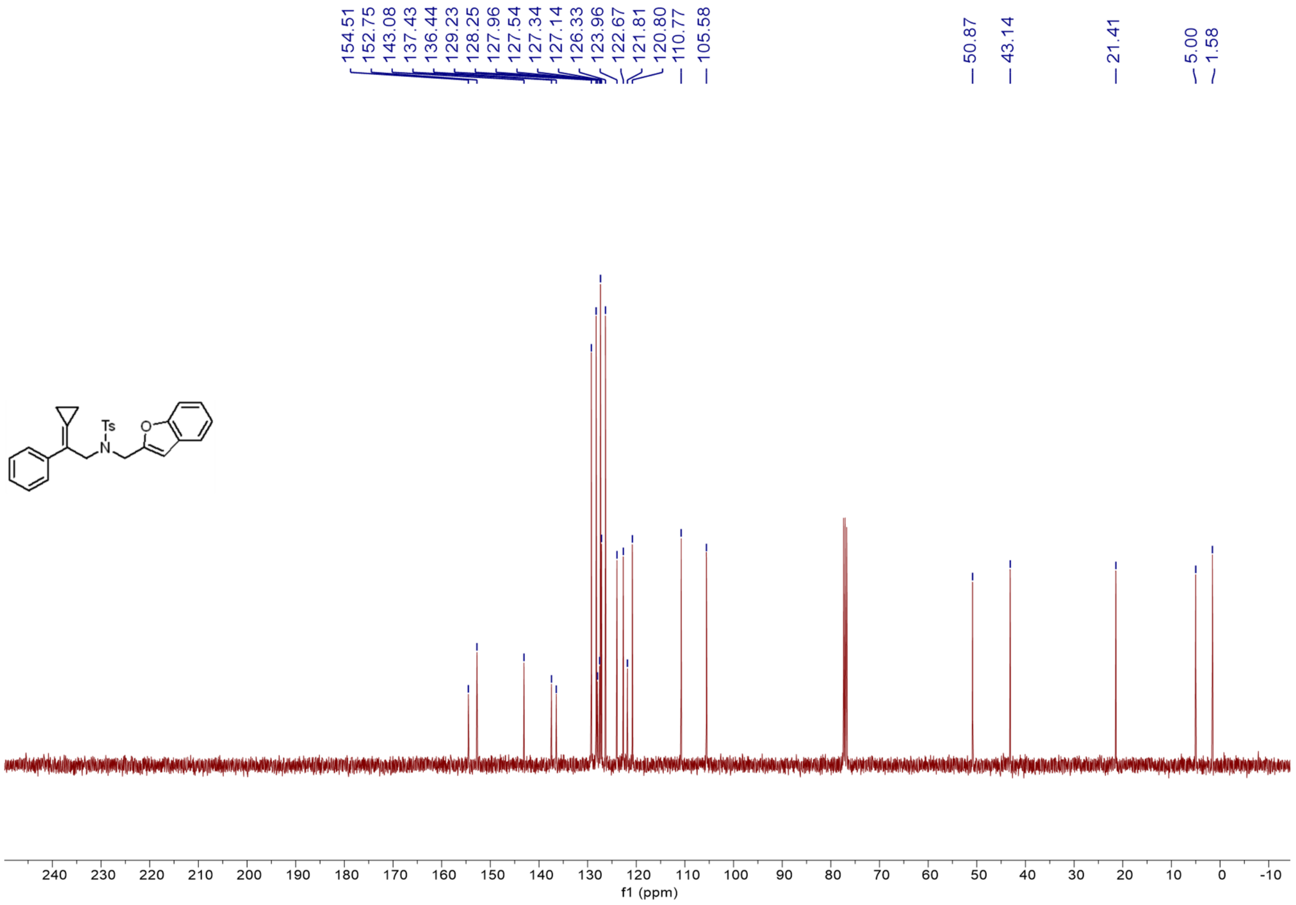
154.51 152.75 143.08 137.43 136.44 129.23 128.25 127.96 127.54 127.34 127.14 126.33 123.96 122.67 121.81 120.80 110.77 105.58
50.87 43.14
21.41
5.00 1.58
Ts
N
O
f1 (ppm)

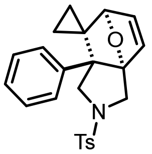

Compound 2a: Yield: 39.0 mg, 99%; A white solid; M.p.: > 200 ºC; ^{1}H NMR (400 MHz, $CDCl_3$) δ 7.78 (d, *J* = 8.0 Hz, 2H), 7.33 (d, *J* = 8.0 Hz, 2H), 7.31 - 7.16 (m, 5H), 6.67 (dd, *J* = 5.8, 1.8 Hz, 1H), 6.07 (d, *J* = 5.8 Hz, 1H), 4.20 (d, *J* = 1.8 Hz, 1H), 4.07 (d, *J* = 9.2 Hz, 1H), 3.79 -3.68 (m, 1H), 3.59 (d, *J* = 9.2 Hz, 1H), 2.44 (s, 3H), 0.82 - 0.74 (m, 1H), 0.70 - 0.62 (m, 1H), 0.61 - 0.53 (m, 1H), 0.51 - 0.42 (m, 1H); ^{13}C NMR (100 MHz, $CDCl_3$) δ 143.4, 138.3, 137.7, 134.0, 129.7, 128.5, 127.7, 127.4, 126.8, 99.1, 87.3, 58.5, 57.8, 49.0, 36.1, 21.6, 9.8, 8.6; IR (neat): ν 2924, 2163, 1971, 1334, 1261, 1160, 1097, 1026, 801, 794, cm^{-1}; HRMS (ESI) Calcd. for $C_{23}H_{23}NO_3SNa$ $[M+Na]^+$: 416.1291, Found: 416.1295.

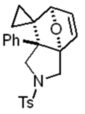
Ph
O
N
Ts

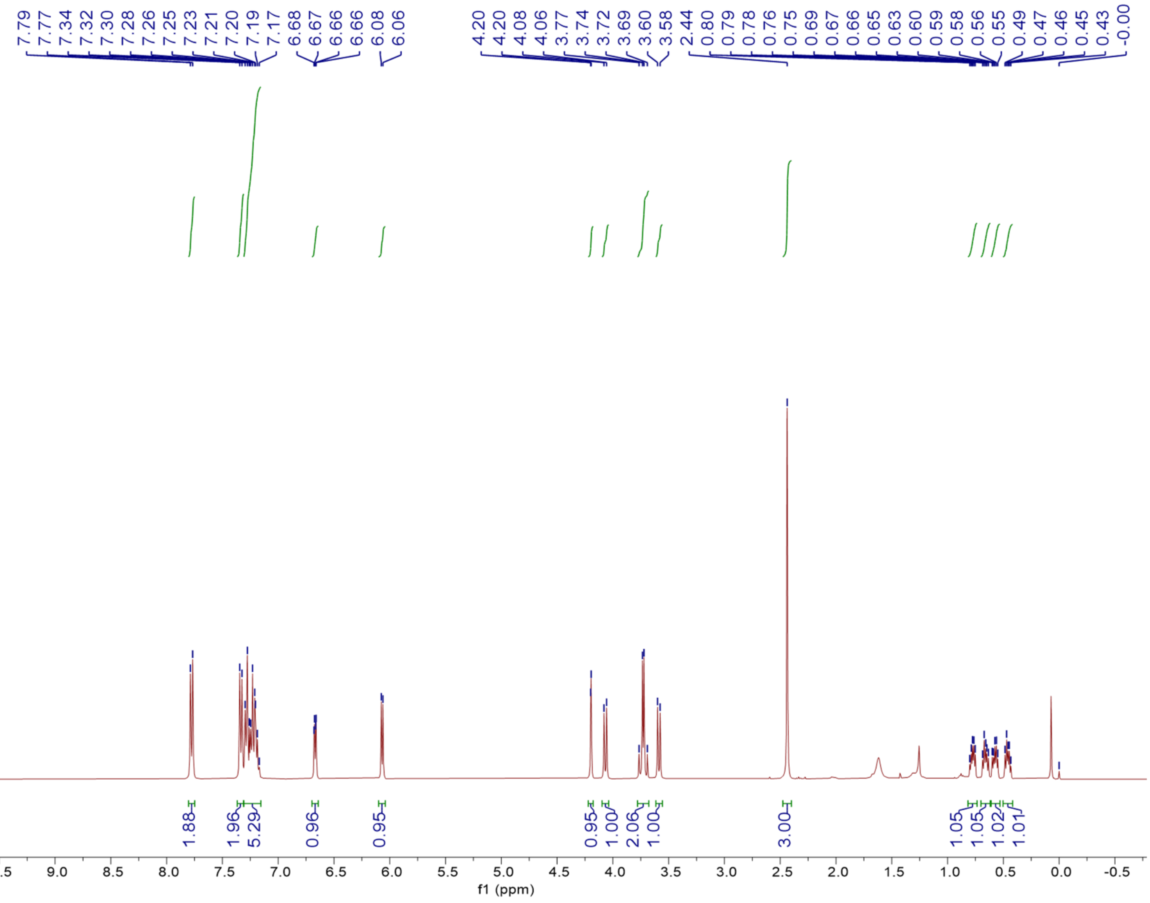
7.79 7.77 7.34 7.32 7.30 7.28 7.26 7.25 7.23 7.21 7.20 7.19 7.17 6.68 6.67 6.66 6.08 6.06
4.20 4.20 4.08 4.06 3.77 3.74 3.72 3.69 3.60 3.58 2.44 0.80 0.79 0.78 0.76 0.75 0.69 0.67 0.66 0.65 0.63 0.60 0.59 0.58 0.56 0.55 0.49 0.47 0.46 0.45 0.43 -0.00
1.88 1.96 5.29 0.96 0.95 0.95 1.00 2.06 1.00 3.00 1.05 1.05 1.02 1.01
10.5 10.0 9.5 9.0 8.5 8.0 7.5 7.0 6.5 6.0 5.5 5.0 4.5 4.0 3.5 3.0 2.5 2.0 1.5 1.0 0.5 0.0 -0.5
f1 (ppm)

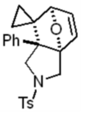
Ph
O
N
Ts

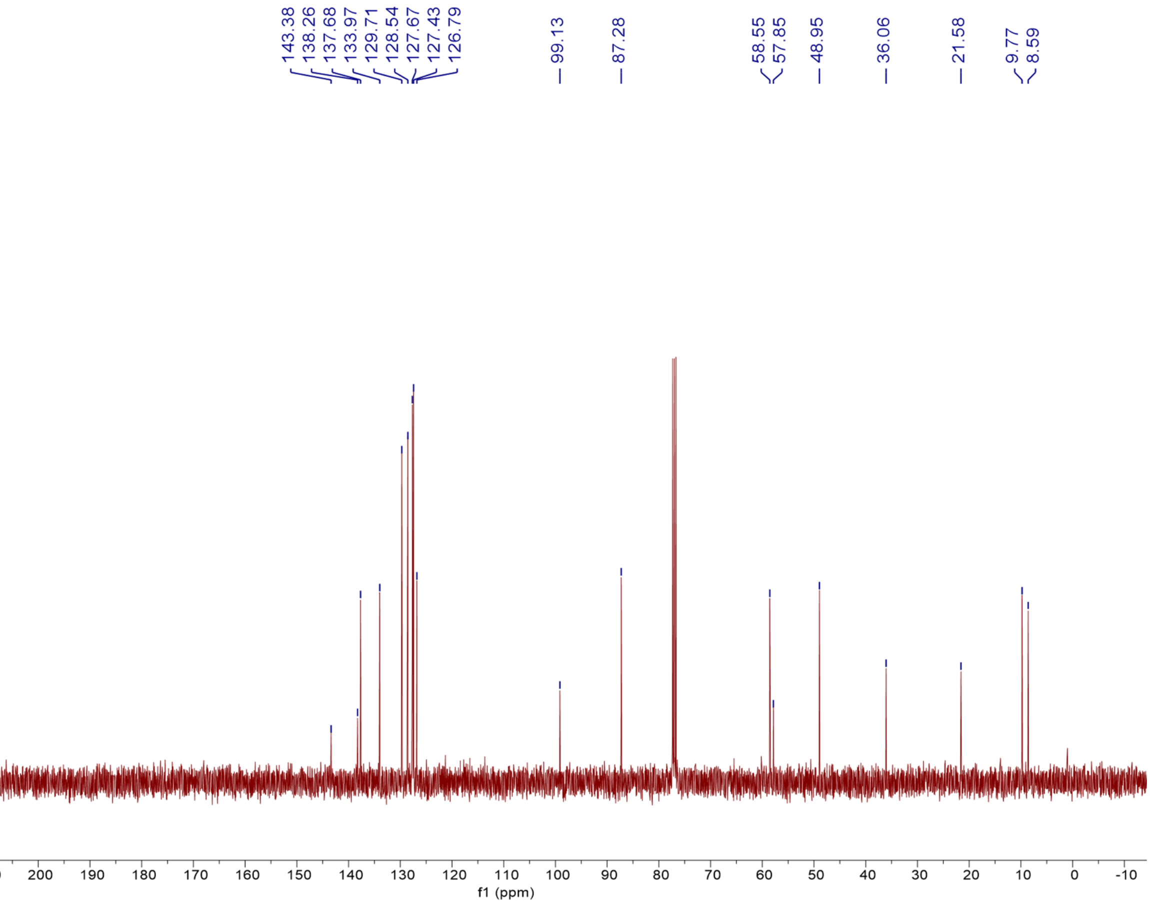
143.38 138.26 137.68 133.97 129.71 128.54 127.67 127.43 126.79
99.13
87.28
58.55 57.85
48.95
36.06
21.58
9.77 8.59
230 220 210 200 190 180 170 160 150 140 130 120 110 100 90 80 70 60 50 40 30 20 10 0 -10
f1 (ppm)

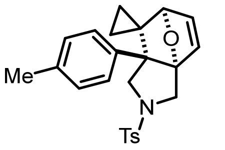

Compound 2b: Yield: 23.2 mg, 57%; A white solid; M.p.: >200 ºC; ^{1}H NMR (400 MHz, $CDCl_3$) δ 7.77 (d, *J* = 8.0 Hz, 2H), 7.33 (d, *J* = 8.0 Hz, 2H), 7.17 (d, *J* = 8.0 Hz, 2H), 7.03 (d, *J* = 8.0 Hz, 2H), 6.65 (dd, *J* = 5.8, 1.8 Hz, 1H), 6.07 (d, *J* = 5.8 Hz, 1H), 4.18 (d, *J* = 1.8 Hz, 1H), 4.04 (d, *J* = 9.0 Hz, 1H), 3.78 - 3.66 (m, 2H), 3.56 (d, *J* = 9.0 Hz, 1H), 2.44 (s, 3H), 2.29 (s, 3H), 0.81 - 0.71 (m, 1H), 0.69 - 0.60 (m, 1H), 0.60 - 0.52 (m, 0H), 0.49 - 0.41 (m, 1H); ^{13}C NMR (100 MHz, $CDCl_3$) δ 143.3, 137.6, 136.5, 135.2, 134.0, 129.7, 128.5, 128.4, 127.4, 99.1, 87.2, 58.6, 57.6, 49.0, 36.0, 21.6, 20.8, 9.7, 8.5; IR (neat): ν 2953, 1514, 1340, 1161, 1106, 1044, 986, 817, 742, 677 cm^{-1}; HRMS (ESI) Calcd. for $C_{24}H_{25}NO_3SNa$ $[M+Na]^+$: 430.1447, Found: 430.1440.

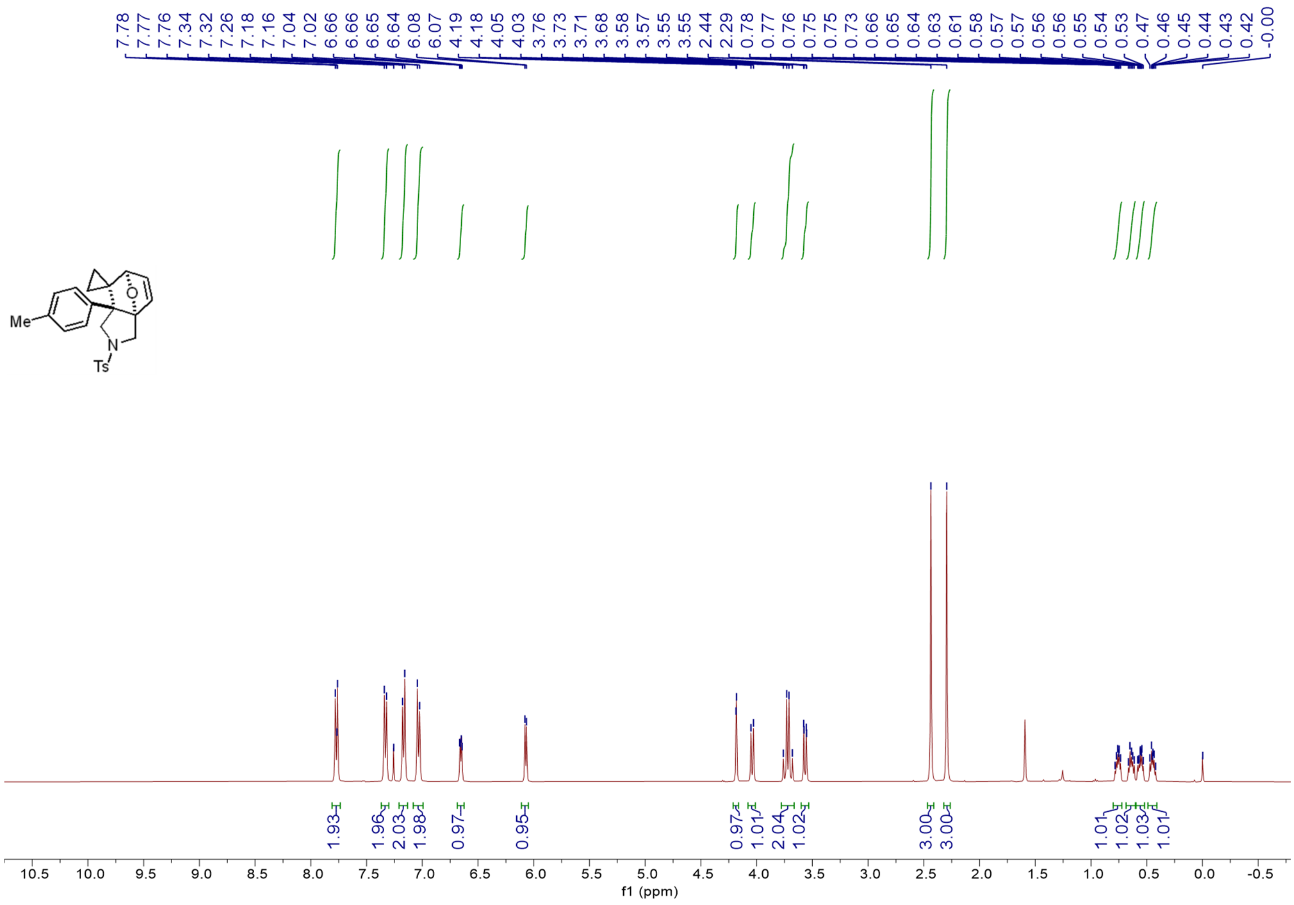
7.78
7.77
7.76
7.34
7.32
7.26
7.18
7.16
7.04
7.02
6.66
6.66
6.65
6.64
6.08
6.07
4.19
4.18
4.05
4.03
3.76
3.73
3.71
3.68
3.58
3.57
3.55
3.55
2.44
2.29
0.78
0.77
0.76
0.75
0.73
0.66
0.65
0.64
0.63
0.61
0.58
0.57
0.57
0.56
0.56
0.55
0.54
0.53
0.47
0.46
0.45
0.44
0.43
0.42
-0.00
Me
O
N
Ts
1.93
1.96
2.03
1.98
0.97
0.95
0.97
1.01
2.04
1.02
3.00
3.00
1.01
1.02
1.03
1.01
f1 (ppm)

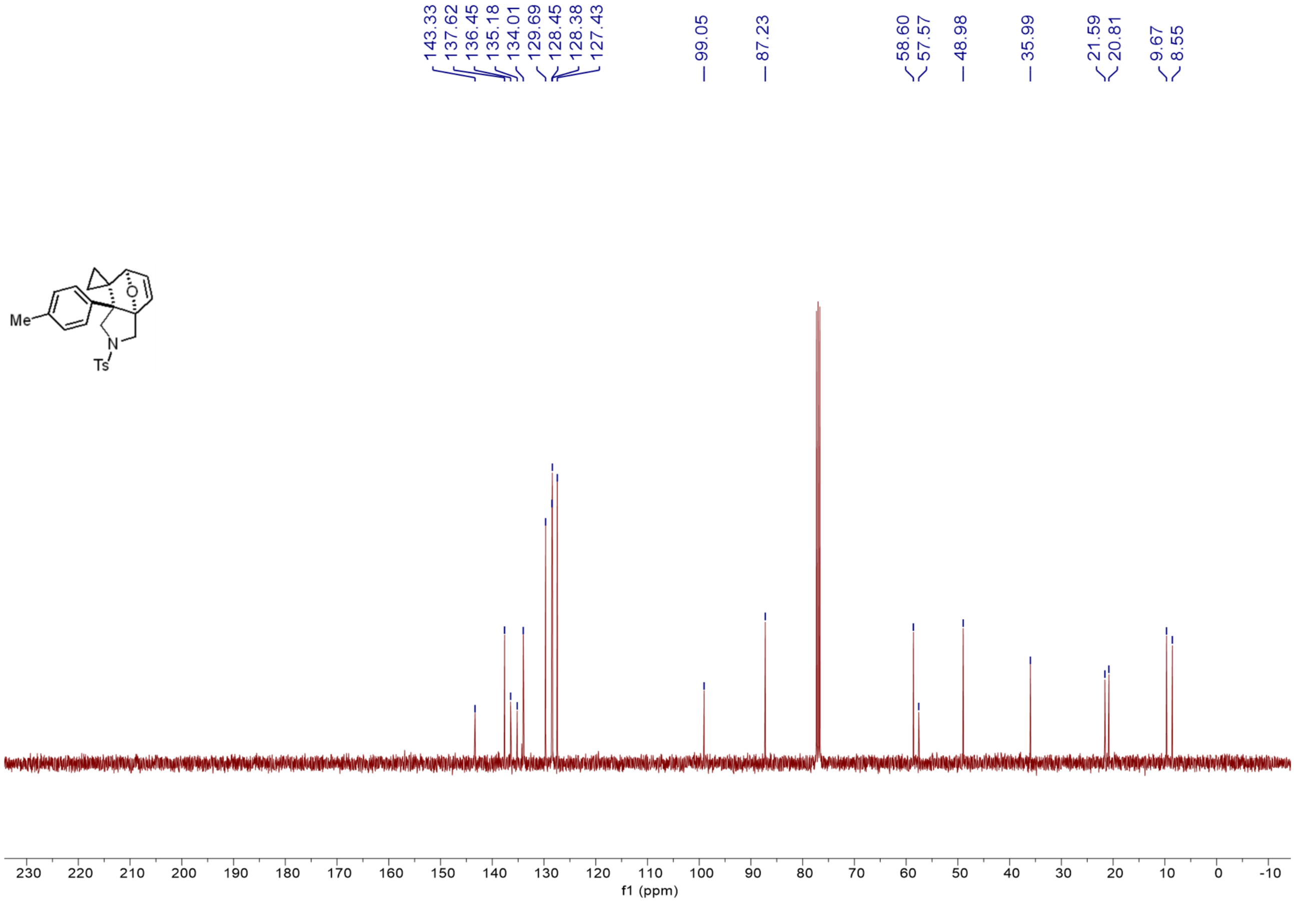
143.33
137.62
136.45
135.18
134.01
129.69
128.45
128.38
127.43
99.05
87.23
58.60
57.57
48.98
35.99
21.59
20.81
9.67
8.55
Me
O
N
Ts
f1 (ppm)

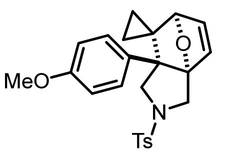

Compound 2c: Yield: 34.7 mg, 82%; A white solid; M.p.: >200 ºC; ^{1}H NMR (400 MHz, $CDCl_3$) δ 7.77 (d, J = 8.0 Hz, 2H), 7.34 (d, J = 8.0 Hz, 2H), 7.21 (d, J = 8.8 Hz, 2H), 6.77 (d, J = 8.8 Hz, 2H), 6.68 - 6.63 (m, 1H), 6.07 (d, J = 5.8 Hz, 1H), 4.18 (d, J = 1.8 Hz, 1H), 4.03 (d, J = 9.2 Hz, 1H), 3.81 - 3.65 (m, 5H), 3.56 (d, J = 9.2 Hz, 1H), 2.44 (s, 3H), 0.80 - 0.72 (m, 1H), 0.69 - 0.60 (m, 1H), 0.59 - 0.52 (m, 1H), 0.47 - 0.39 (m, 1H); ^{13}C NMR (100 MHz, $CDCl_3$) δ 158.3, 143.4, 137.6, 134.3, 134.1, 130.1, 129.73, 129.71, 127.4, 113.0, 99.0, 87.2, 58.6, 57.2, 55.2, 49.0, 36.0, 21.6, 9.6, 8.5; IR (neat): ν 2960, 1712, 1489, 1477, 1342, 1161, 1103, 1002, 814, 746, 666 cm^{-1}; HRMS (ESI) Calcd. for $C_{24}H_{25}NO_4SNa$ $[M+Na]^+$: 446.1397, Found: 446.1392.

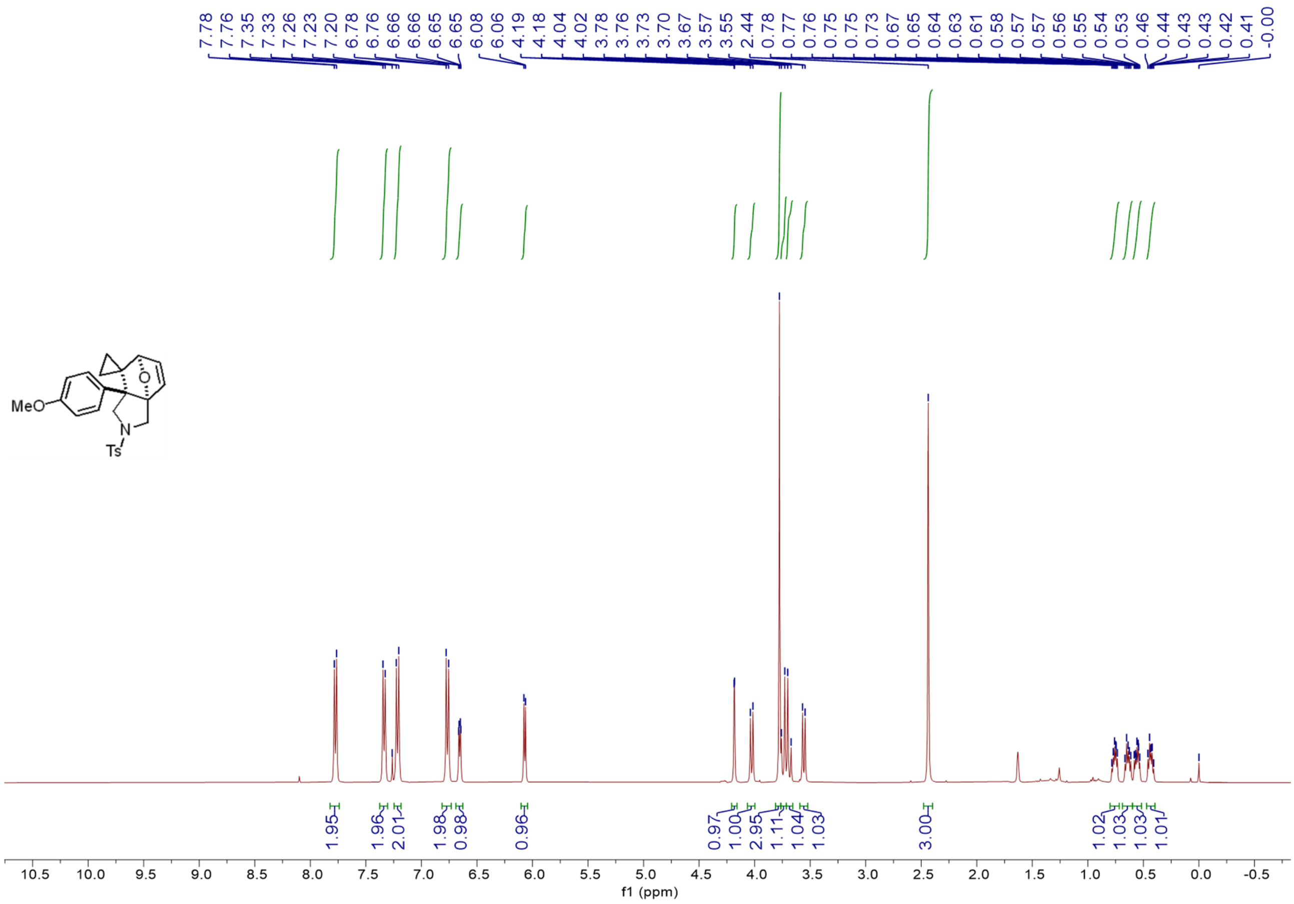
7.78 7.76 7.35 7.33 7.26 7.23 7.20 6.78 6.76 6.66 6.66 6.65 6.65 6.08 6.06 4.19 4.18 4.04 4.02 3.78 3.76 3.73 3.70 3.67 3.57 3.55 2.44 0.78 0.77 0.76 0.75 0.75 0.73 0.67 0.65 0.64 0.63 0.61 0.58 0.57 0.57 0.56 0.55 0.54 0.53 0.46 0.44 0.43 0.43 0.42 0.41 -0.00
MeO
O
N
Ts
1.95 1.96 2.01 1.98 0.98 0.96 0.97 1.00 2.95 1.11 1.04 1.03 3.00 1.02 1.03 1.03 1.01
f1 (ppm)

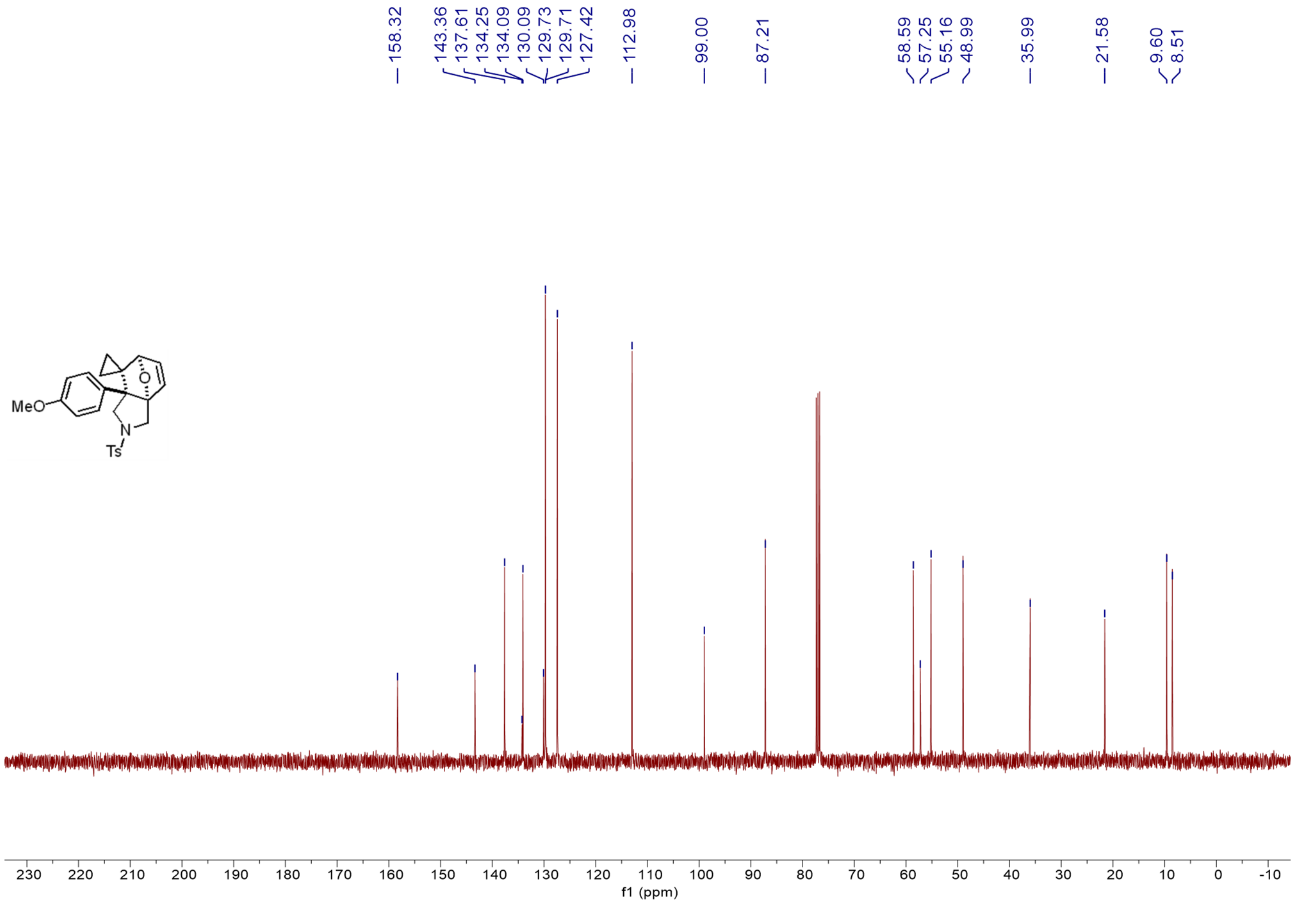
158.32 143.36 137.61 134.25 134.09 130.09 129.73 129.71 127.42 112.98 99.00 87.21 58.59 57.25 55.16 48.99 35.99 21.58 9.60 8.51
MeO
O
N
Ts
f1 (ppm)

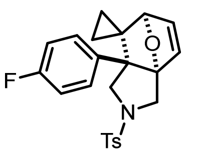

Compound 2d: Yield: 37.8 mg, 92%; A white solid; M.p.: 193 - 195 ºC; ^{1}H NMR (400 MHz, $CDCl_3$) δ 7.77 (d, J = 8.0 Hz, 2H), 7.34 (d, J = 8.0 Hz, 2H), 7.27 (dd, J = 8.4, 5.6 Hz, 2H), 6.91 (t, J = 8.4 Hz, 2H), 6.68 (dd, J = 5.8, 1.8 Hz, 1H), 6.06 (d, J = 5.8 Hz, 1H), 4.20 (d, J = 1.8 Hz, 1H), 4.02 (d, J = 9.2 Hz, 1H), 3.78 - 3.66 (m, 1H), 3.57 (d, J = 9.2 Hz, 1H), 2.44 (s, 3H), 0.83 - 0.74 (m, 1H), 0.71 - 0.63 (m, 1H), 0.61 - 0.54 (m, 1H), 0.45 - 0.37 (m, 1H); ^{13}C NMR (100 MHz, $CDCl_3$) δ 161.7 (d, J = 246.8 Hz), 143.5, 137.8, 133.9, 130.3 (d, J = 7.8 Hz), 129.8, 127.4, 114.5 (d, J = 21.0 Hz), 99.0, 87.3, 58.5, 57.4, 48.9, 36.1, 21.6, 9.8, 8.6; ^{19}F NMR (376 MHz, $CDCl_3$) δ -116.0. IR (neat): ν 2961, 2922, 1508, 1336, 1158, 1101, 1059, 1033, 819, 803, 677 cm^{-1}; HRMS (ESI) Calcd. for $C_{23}H_{22}NO_3SNaF$ $[M+Na]^+$: 434.1197, Found: 434.1203.

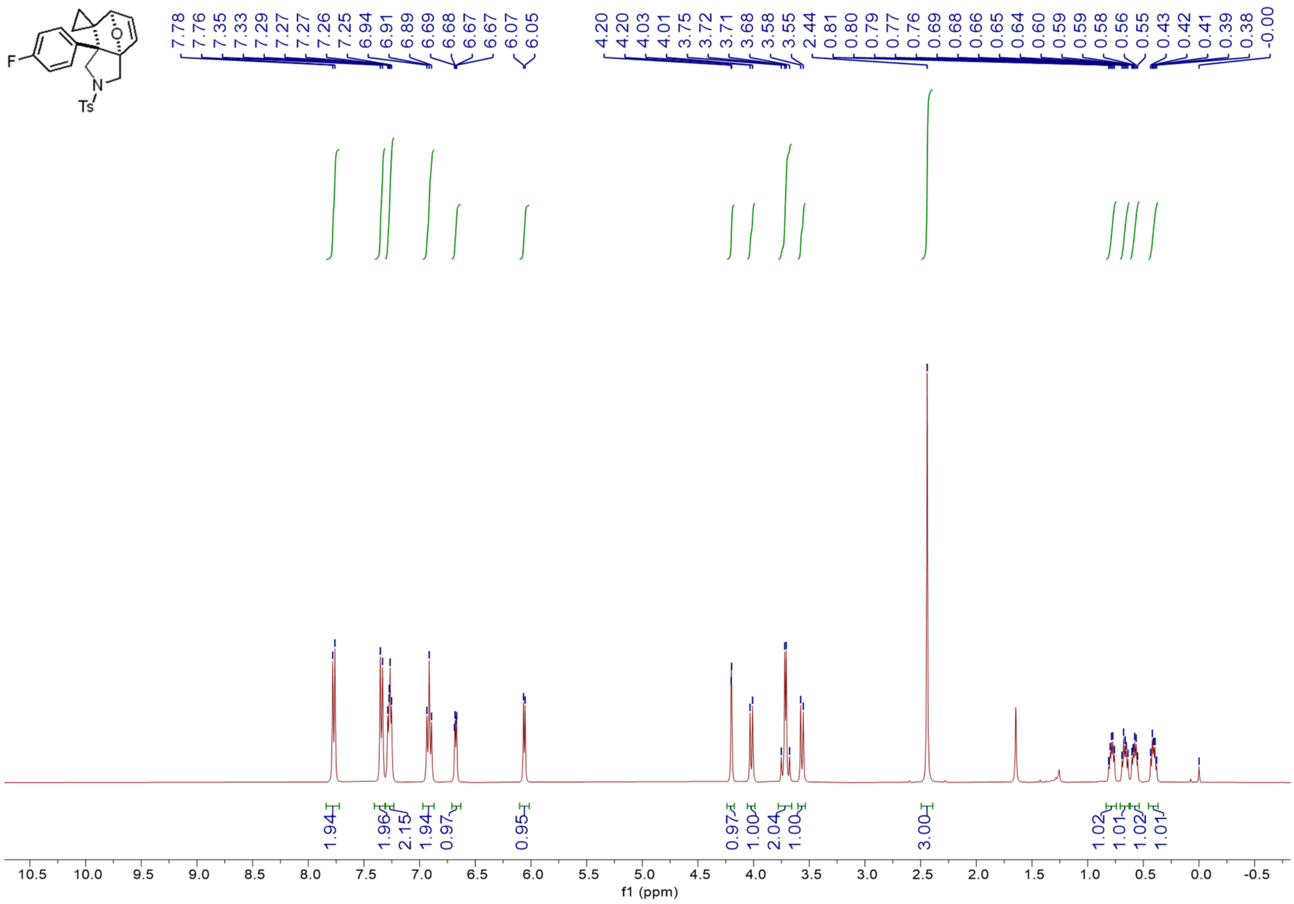
F
N
Ts
O
7.78 7.76 7.35 7.33 7.29 7.27 7.27 7.26 7.25 6.94 6.91 6.89 6.69 6.68 6.67 6.67 6.07 6.05
4.20 4.20 4.03 4.01 3.75 3.72 3.71 3.68 3.58 3.55 2.44 0.81 0.80 0.79 0.77 0.76 0.69 0.68 0.66 0.65 0.64 0.60 0.59 0.59 0.58 0.56 0.55 0.43 0.42 0.41 0.39 0.38 -0.00
1.94 1.96 2.15 1.94 0.97 0.95 0.97 1.00 2.04 1.00 3.00 1.02 1.01 1.02 1.01
10.5 10.0 9.5 9.0 8.5 8.0 7.5 7.0 6.5 6.0 5.5 5.0 4.5 4.0 3.5 3.0 2.5 2.0 1.5 1.0 0.5 0.0 -0.5
f1 (ppm)

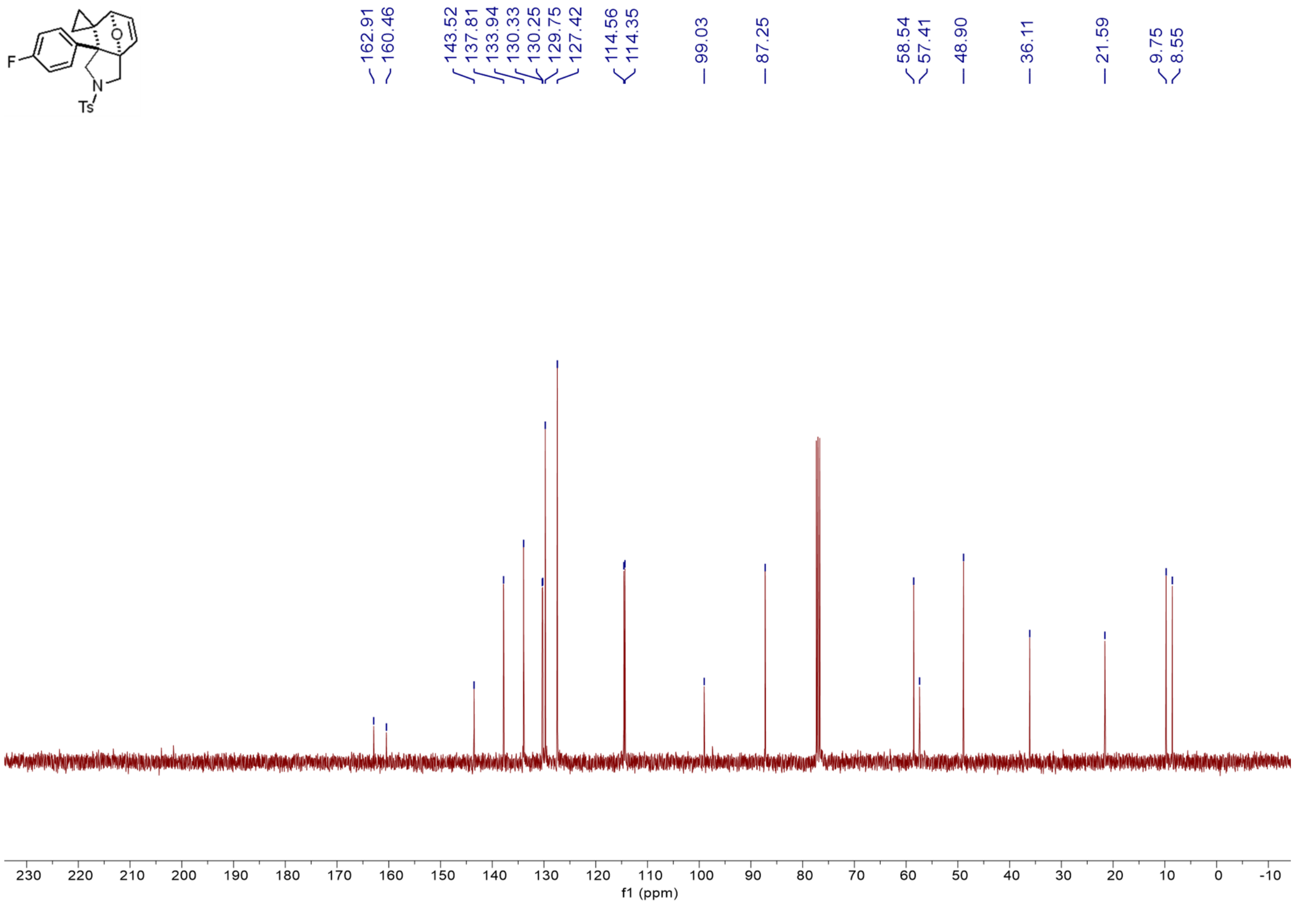
F
N
Ts
O
162.91 160.46
143.52 137.81 133.94 130.33 130.25 129.75 127.42
114.56 114.35
99.03
87.25
58.54 57.41
48.90
36.11
21.59
9.75 8.55
230 220 210 200 190 180 170 160 150 140 130 120 110 100 90 80 70 60 50 40 30 20 10 0 -10
f1 (ppm)

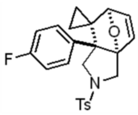
F
O
N
Ts

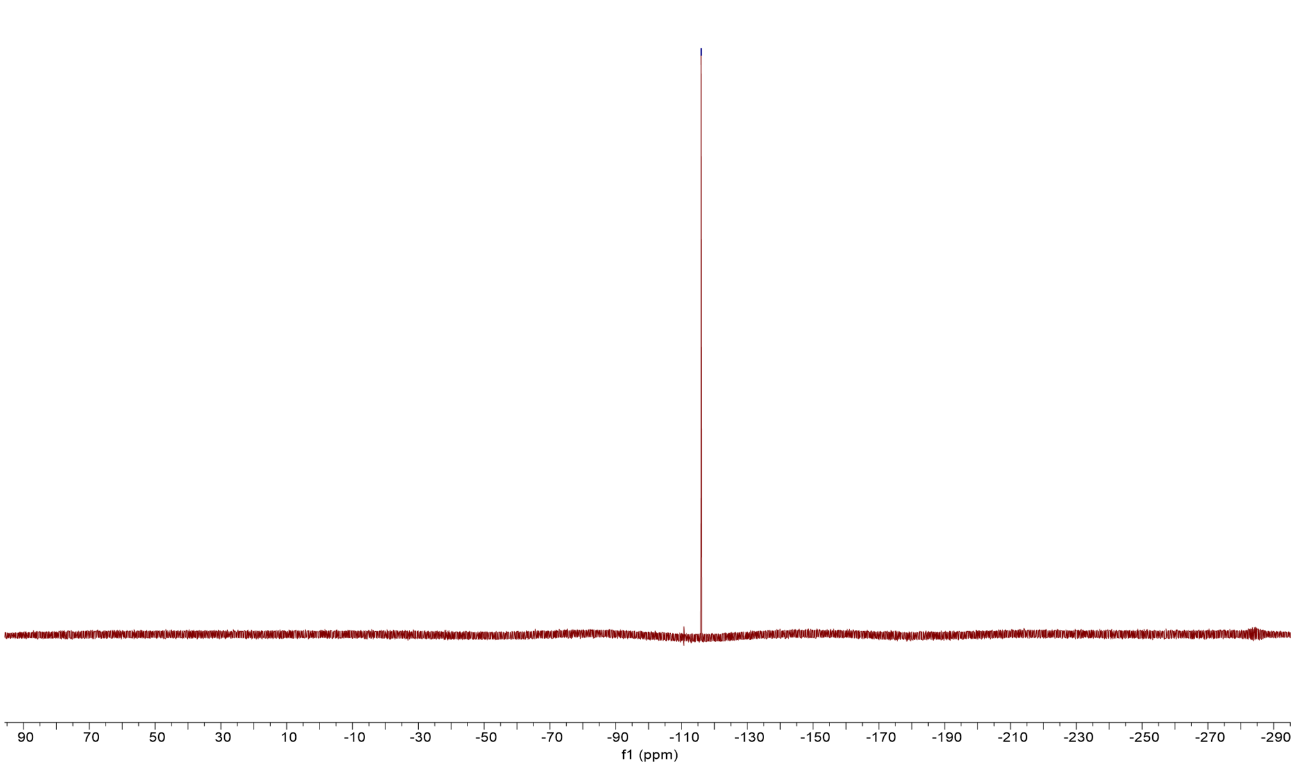
-115.97
90
70
50
30
10
-10
-30
-50
-70
-90
-110
-130
-150
-170
-190
-210
-230
-250
-270
-290
f1 (ppm)

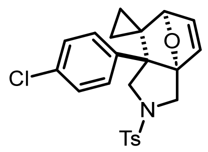

Compound 2e: Yield: 31.7 mg, 74%; A white solid; M.p.: 194 - 196 ºC; ^{1}H NMR (400 MHz, $CDCl_3$) δ 7.76 (d, *J* = 8.2 Hz, 3H), 7.34 (d, *J* = 8.2 Hz, 3H), 7.25 - 7.16 (m, 4H), 6.67 (dd, *J* = 5.8, 1.8 Hz, 1H), 6.06 (d, *J* = 5.8 Hz, 1H), 4.20 (d, *J* = 1.8 Hz, 1H), 4.01 (d, *J* = 9.4 Hz, 1H), 3.75 - 3.67 (m, 2H), 3.56 (d, *J* = 9.4 Hz, 1H), 2.44 (s, 3H), 0.83 - 0.75 (m, 1H), 0.70 - 0.62 (m, 1H), 0.62 - 0.54 (m, 1H), 0.45 - 0.36 (m, 1H); ^{13}C NMR (100 MHz, $CDCl_3$) δ 143.5, 137.9, 136.8, 133.9, 132.9, 130.0, 129.7, 127.8, 127.4, 99.1, 97.4, 87.3, 58.4, 57.6, 48.9, 36.1, 21.6, 9.8, 8.5; IR (neat): ν 2993, 1712, 1597, 1494, 1342, 1162, 1100, 818, 748, 671 cm^{-1}; HRMS (ESI) Calcd. for $C_{23}H_{22}NO_3SNaCl$ $[M+Na]^+$: 450.0901, Found: 450.0909.

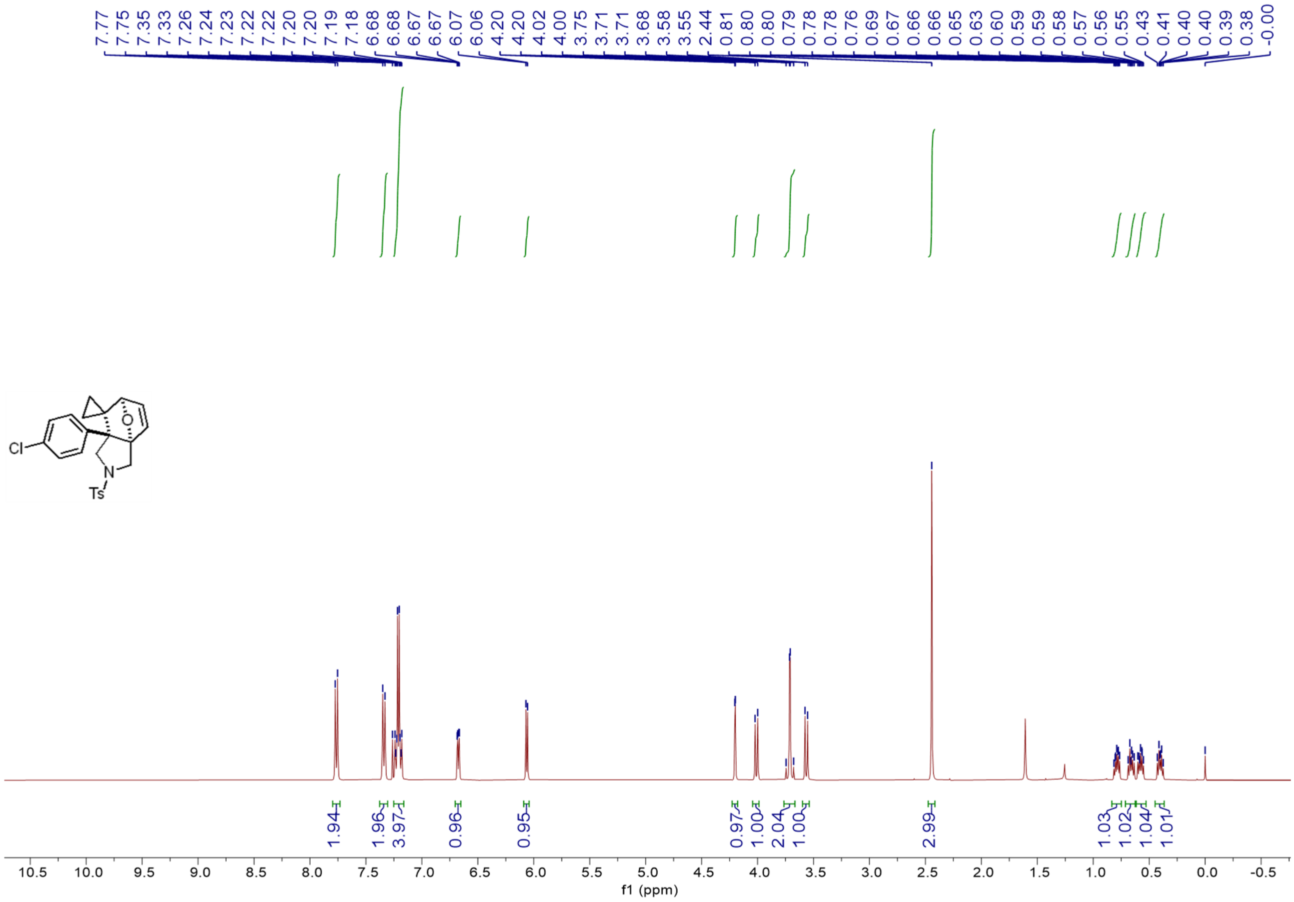
7.77
7.75
7.35
7.33
7.26
7.24
7.23
7.22
7.22
7.20
7.19
7.18
6.68
6.67
6.67
6.07
6.06
4.20
4.20
4.02
4.00
3.75
3.71
3.71
3.68
3.58
3.55
2.44
0.81
0.80
0.80
0.79
0.78
0.76
0.69
0.67
0.66
0.66
0.65
0.63
0.60
0.59
0.59
0.58
0.57
0.56
0.55
0.43
0.41
0.40
0.40
0.39
0.38
-0.00
Cl
N
Ts
O
1.94
1.96
3.97
0.96
0.95
0.97
1.00
2.04
1.00
2.99
1.03
1.02
1.04
1.01
f1 (ppm)

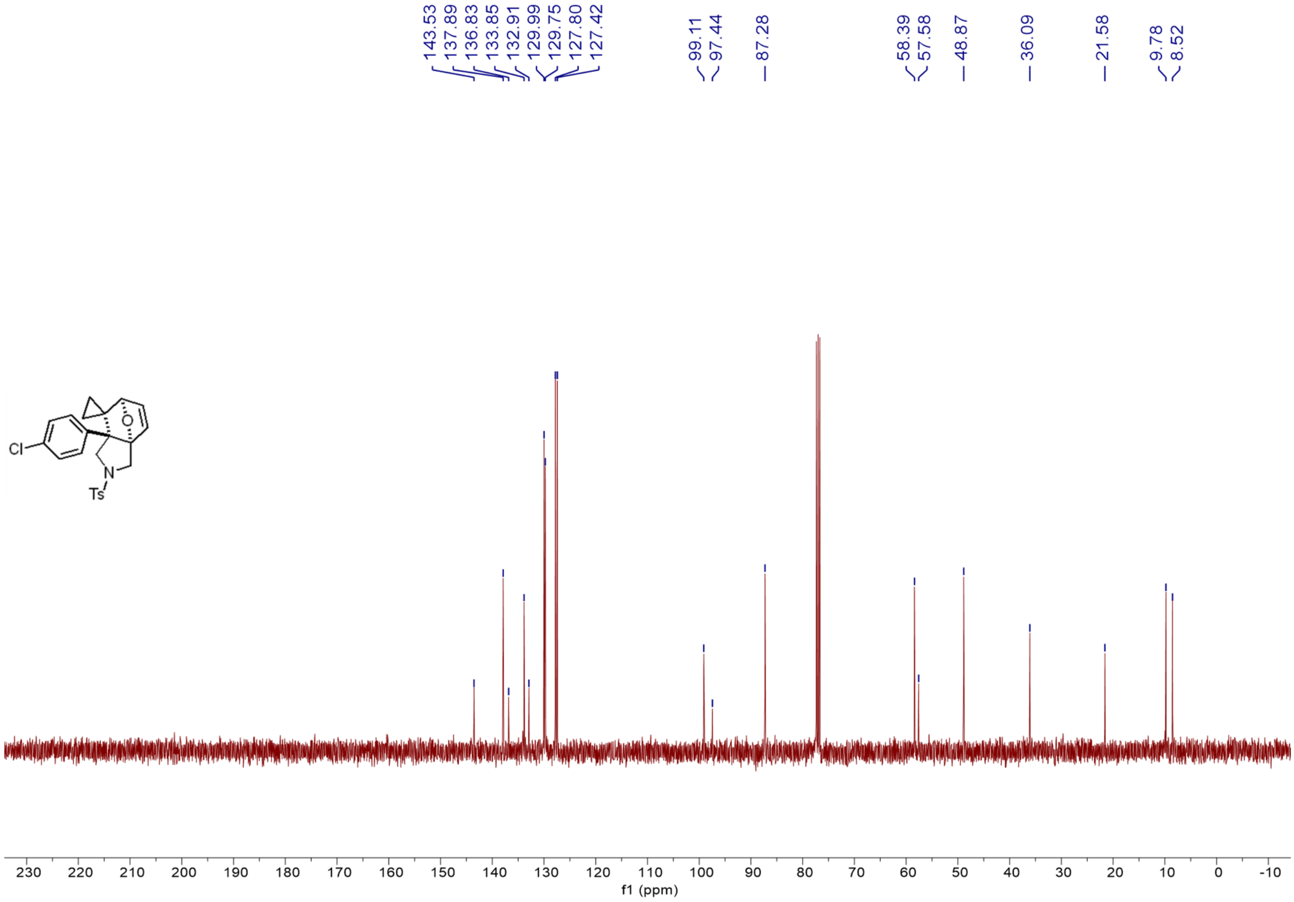
143.53
137.89
136.83
133.85
132.91
129.99
129.75
127.80
127.42
99.11
97.44
87.28
58.39
57.58
48.87
36.09
21.58
9.78
8.52
Cl
N
Ts
O
f1 (ppm)

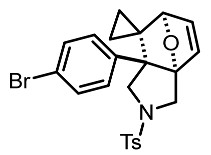

Compound 2f: Yield: 32.6 mg, 69%; A yellow solid; M.p.: > 200 ºC; ^{1}H NMR (400 MHz, $CDCl_3$) δ 7.76 (d, *J* = 8.0 Hz, 1H), 7.38 - 7.31 (m, 4H), 7.17 (d, *J* = 8.6 Hz, 2H), 6.67 (dd, *J* = 5.8, 1.8 Hz, 1H), 6.06 (d, *J* = 5.8 Hz, 1H), 4.20 (d, *J* = 1.8 Hz, 1H), 4.01 (d, *J* = 9.4 Hz, 1H), 3.81 - 3.65 (m, 2H), 3.56 (d, *J* = 9.4 Hz, 1H), 2.44 (s, 3H), 0.83 - 0.76 (m, 1H), 0.70 - 0.62 (m, 1H), 0.61 - 0.54 (m, 1H), 0.44 - 0.36 (m, 1H); ^{13}C NMR (100 MHz, $CDCl_3$) δ 143.6, 137.9, 137.4, 134.0, 133.8, 130.8, 130.4, 129.8, 127.4, 121.1, 99.1, 87.3, 58.3, 57.6, 48.9, 36.1, 21.6, 9.8, 8.5; IR (neat): ν 2961, 1712, 1473, 1343, 1161, 1100, 1040, 891, 801, 714 cm^{-1}; HRMS (ESI) Calcd. for $C_{23}H_{22}NO_3SNaBr$ $[M+Na]^+$: 494.0396, Found: 494.0397.

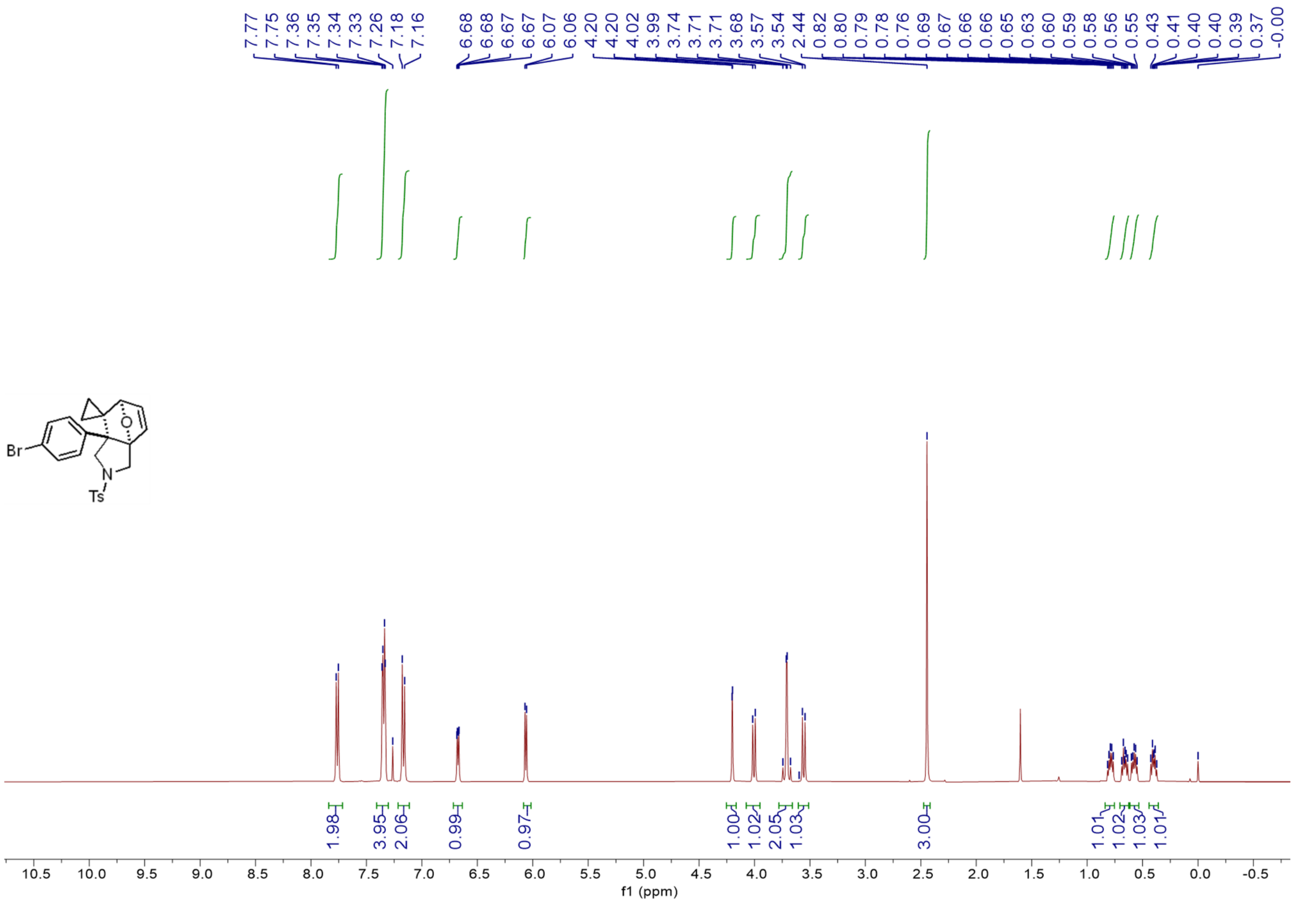
7.77 7.75 7.36 7.35 7.34 7.33 7.26 7.18 7.16 6.68 6.68 6.67 6.67 6.07 6.06 4.20 4.20 4.02 3.99 3.74 3.71 3.71 3.68 3.57 3.54 2.44 0.82 0.80 0.79 0.78 0.76 0.69 0.67 0.66 0.66 0.65 0.63 0.60 0.59 0.58 0.56 0.55 0.43 0.41 0.40 0.39 0.37 -0.00
Br
O
N
Ts
1.98 3.95 2.06 0.99 0.97 1.00 1.02 2.05 1.03 3.00 1.01 1.02 1.03 1.01
10.5 10.0 9.5 9.0 8.5 8.0 7.5 7.0 6.5 6.0 5.5 5.0 4.5 4.0 3.5 3.0 2.5 2.0 1.5 1.0 0.5 0.0 -0.5
f1 (ppm)

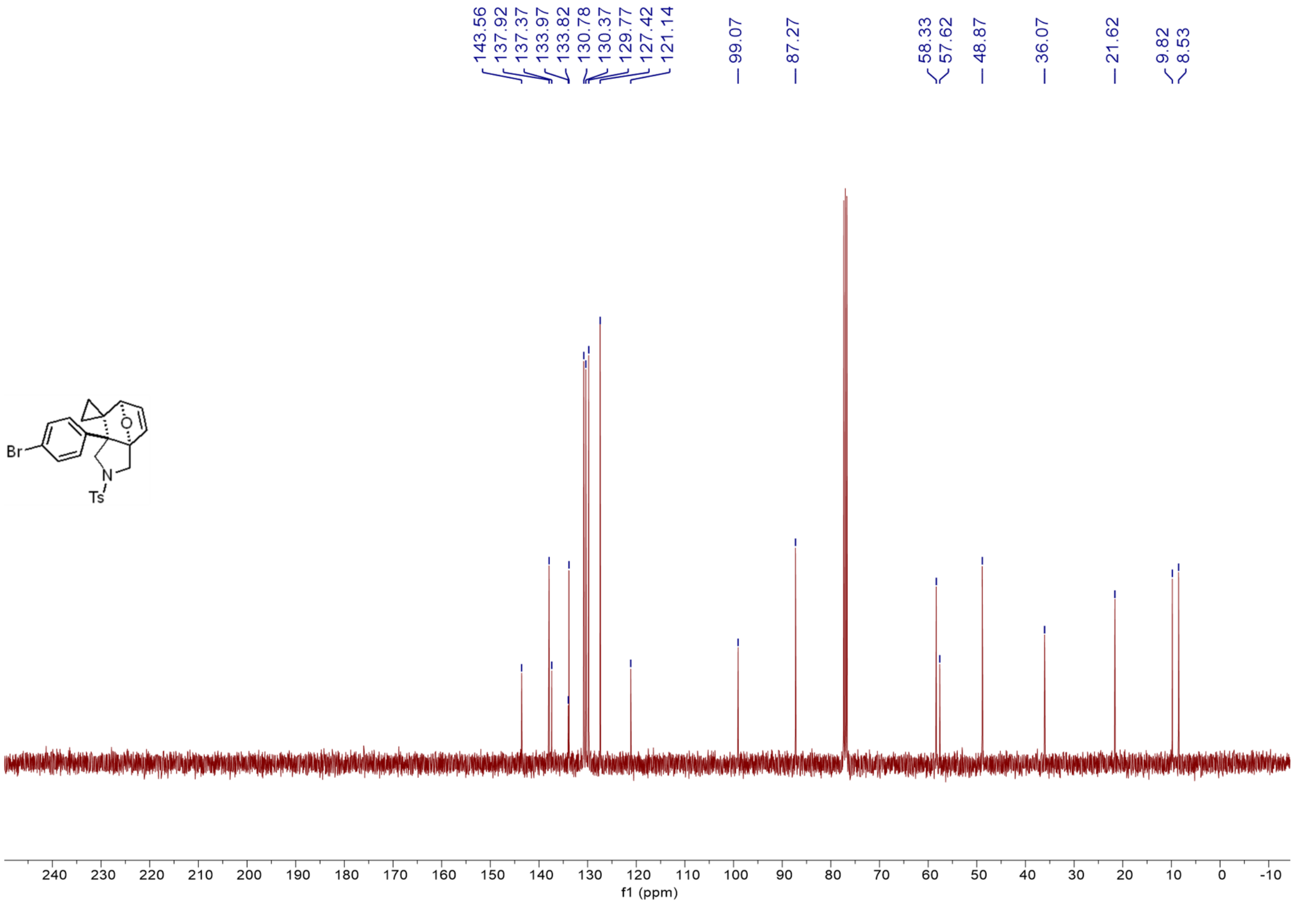
143.56 137.92 137.37 133.97 133.82 130.78 130.37 129.77 127.42 121.14 99.07 87.27 58.33 57.62 48.87 36.07 21.62 9.82 8.53
Br
O
N
Ts
240 230 220 210 200 190 180 170 160 150 140 130 120 110 100 90 80 70 60 50 40 30 20 10 0 -10
f1 (ppm)

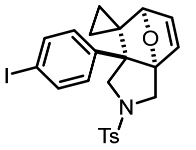

Compound 2g: Yield: 36.1 mg, 70%; A white solid; M.p.: 195 - 197 ºC; ^{1}H NMR (400 MHz, $CDCl_3$) δ 7.76 (d, *J* = 8.2 Hz, 2H), 7.55 (d, *J* = 8.8 Hz, 2H), 7.34 (d, *J* = 8.2 Hz, 2H), 7.03 (d, *J* = 8.8 Hz, 2H), 6.67 (dd, *J* = 5.8, 1.8 Hz, 1H), 6.06 (d, *J* = 5.8 Hz, 1H), 4.20 (d, *J* = 1.8 Hz, 1H), 3.99 (d, *J* = 9.4 Hz, 1H), 3.78 - 3.64 (m, 2H), 3.55 (d, *J* = 9.4 Hz, 1H), 2.44 (s, 3H), 0.83 - 0.75 (m, 1H), 0.70 - 0.62 (m, 1H), 0.61 - 0.53 (m, 1H), 0.44 - 0.36 (m, 1H); ^{13}C NMR (100 MHz, $CDCl_3$) δ 143.6, 138.1, 137.9, 136.8, 134.0, 133.8, 130.6, 129.8, 127.4, 99.1, 92.8, 87.3, 58.3, 57.7, 48.9, 36.1, 21.6, 9.8, 8.5; IR (neat): ν 2959, 1711, 1489, 1477, 1161, 1104, 1002, 814, 746, 666, 663 cm^{-1}; HRMS (ESI) Calcd. for $C_{23}H_{22}NO_3SNaI$ $[M+Na]^+$: 542.0257, Found: 542.0263.

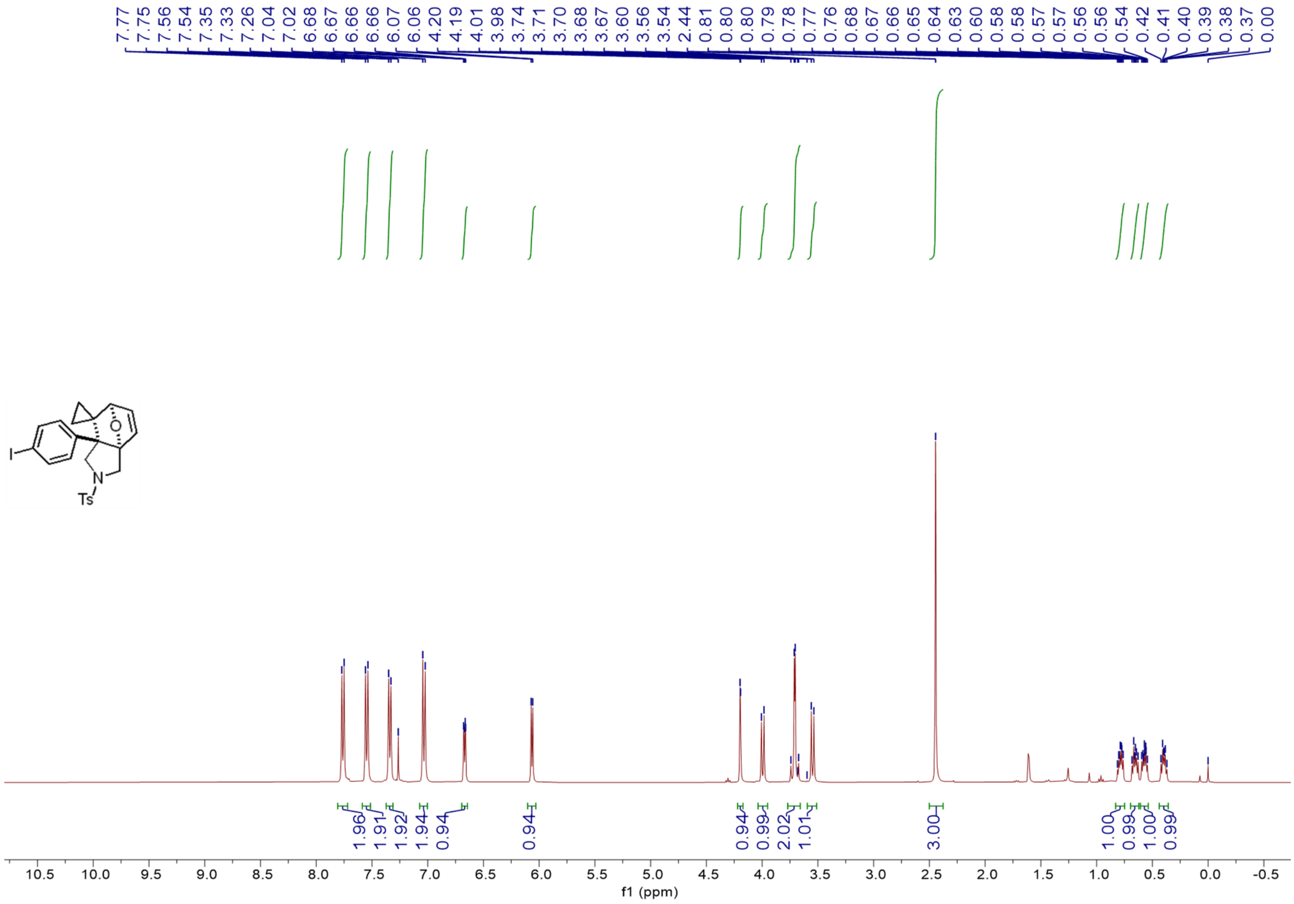
7.77 7.75 7.56 7.54 7.35 7.33 7.26 7.04 7.02 6.68 6.67 6.66 6.66 6.07 6.06 4.20 4.19 4.01 3.98 3.74 3.71 3.70 3.68 3.67 3.60 3.56 3.54 2.44 0.81 0.80 0.80 0.79 0.78 0.77 0.76 0.68 0.67 0.66 0.65 0.64 0.63 0.60 0.58 0.58 0.57 0.57 0.56 0.56 0.54 0.42 0.41 0.40 0.39 0.38 0.37 0.00
I
O
N
Ts
1.96 1.91 1.92 1.94 0.94 0.94 0.94 0.99 2.02 1.01 3.00 1.00 0.99 1.00 0.99
f1 (ppm)

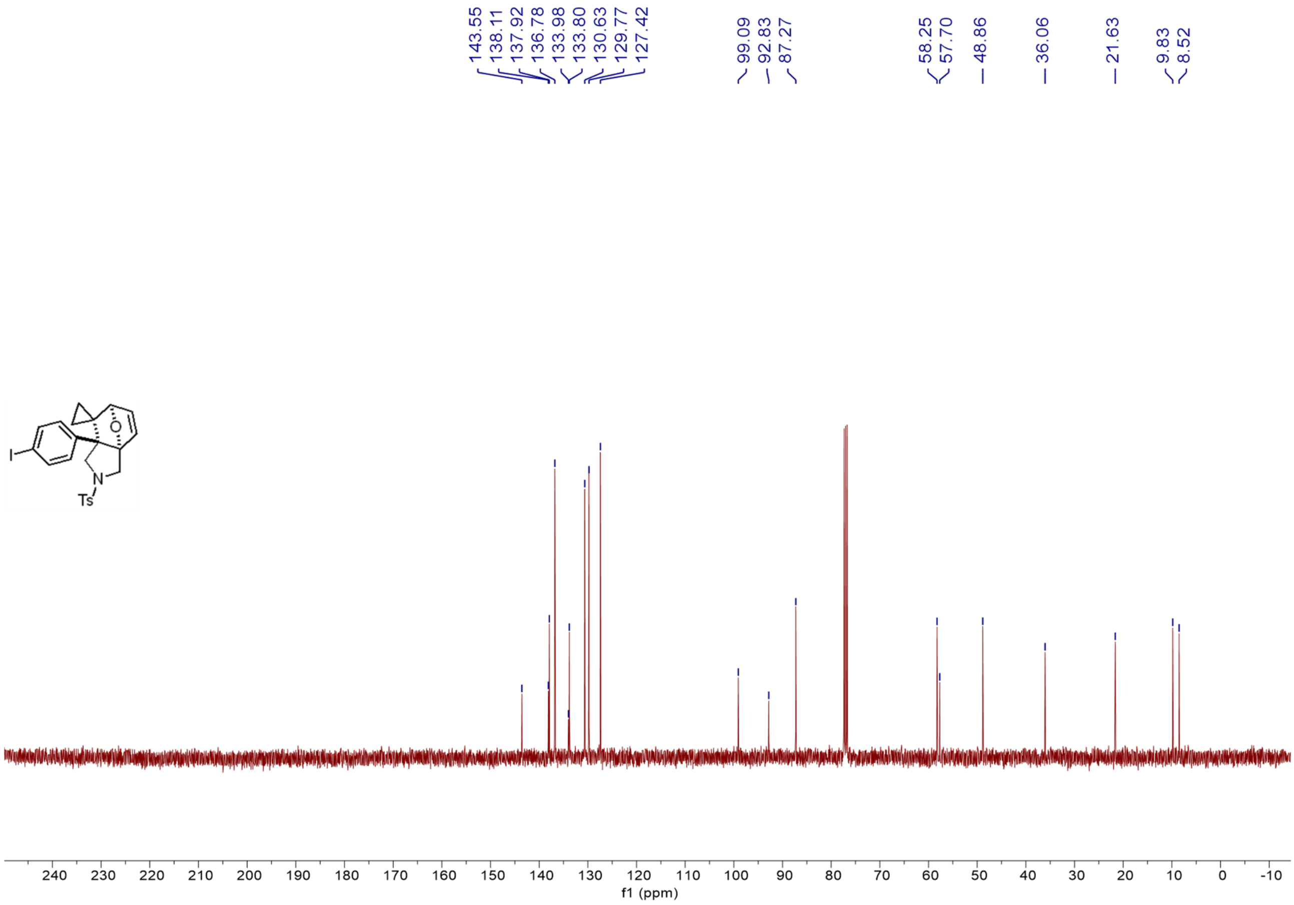
143.55 138.11 137.92 136.78 133.98 133.80 130.63 129.77 127.42 99.09 92.83 87.27 58.25 57.70 48.86 36.06 21.63 9.83 8.52
I
O
N
Ts
f1 (ppm)

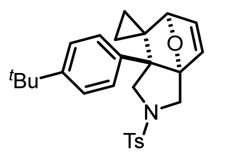

Compound 2h: Yield: 30.1 mg, 67%; A white solid; M.p.: 196 - 198 ºC; ^{1}H NMR (400 MHz, $CDCl_3$) δ 7.78 (d, *J* = 8.0 Hz, 2H), 7.34 (d, *J* = 8.0 Hz, 2H), 7.27 - 7.17 (m, 4H), 6.65 (dd, *J* = 5.8, 1.8 Hz, 1H), 6.09 (d, *J* = 5.8 Hz, 1H), 4.19 (d, *J* = 1.8 Hz, 1H), 4.06 (d, *J* = 9.2 Hz, 1H), 3.82 - 3.65 (m, 2H), 3.56 (d, *J* = 9.2 Hz, 1H), 2.44 (s, 3H), 1.29 (s, 9H), 0.80 - 0.71 (m, 1H), 0.68 - 0.61 (m, 1H), 0.59 - 0.52 (m, 1H), 0.51 - 0.44 (m, 1H); ^{13}C NMR (100 MHz, $CDCl_3$) δ 149.5, 143.3, 137.5, 135.1, 134.4, 134.1, 129.7, 128.2, 127.4, 124.5, 99.1, 87.2, 58.5, 57.5, 49.0, 36.0, 34.3, 31.2, 21.6, 9.7, 8.6; IR (neat): ν 2962, 1343, 1163, 1105, 984, 897, 816, 719, 673 cm^{-1}; HRMS (ESI) Calcd. for $C_{27}H_{32}NO_3SNa$ $[M+Na]^+$: 450.2097, Found: 450.2096.

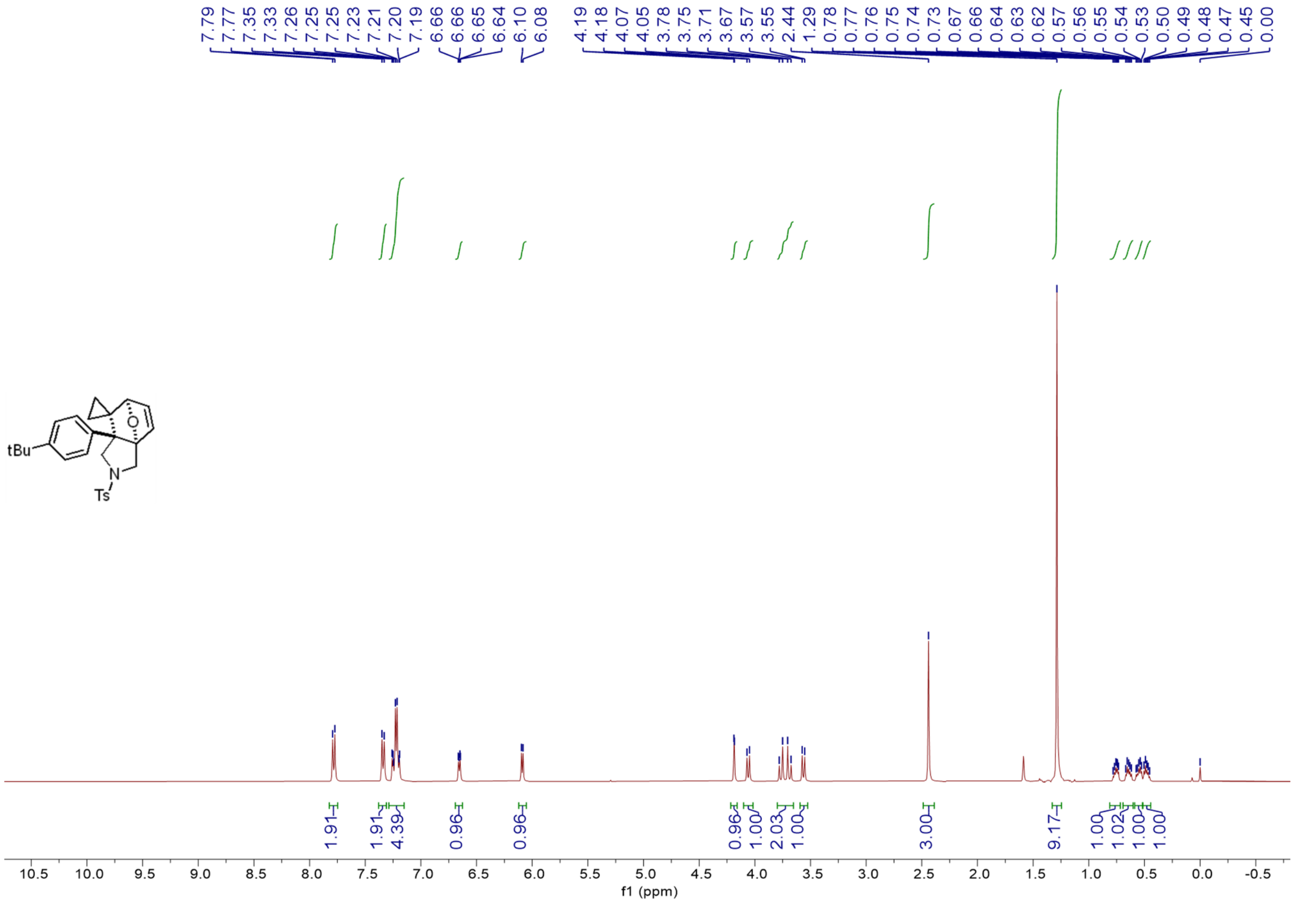
7.79
7.77
7.35
7.33
7.26
7.25
7.25
7.23
7.21
7.20
7.19
6.66
6.66
6.65
6.64
6.10
6.08
4.19
4.18
4.07
4.05
3.78
3.75
3.71
3.67
3.57
3.55
2.44
1.29
0.78
0.77
0.76
0.75
0.74
0.73
0.67
0.66
0.64
0.63
0.62
0.57
0.56
0.55
0.54
0.53
0.50
0.49
0.48
0.47
0.45
0.00
tBu
O
N
Ts
1.91
1.91
4.39
0.96
0.96
0.96
1.00
2.03
1.00
3.00
9.17
1.00
1.02
1.00
1.00
f1 (ppm)

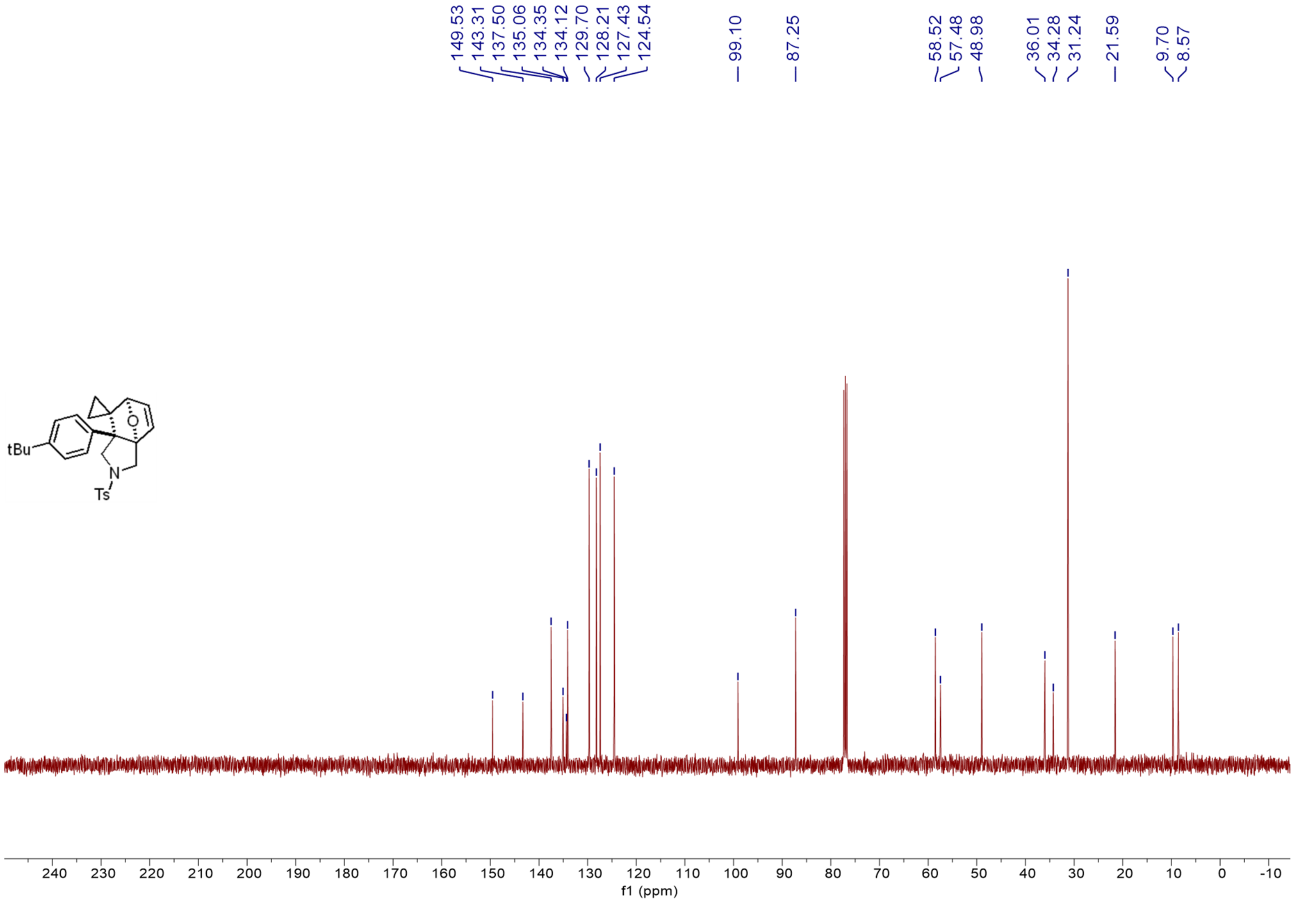
149.53
143.31
137.50
135.06
134.35
134.12
129.70
128.21
127.43
124.54
99.10
87.25
58.52
57.48
48.98
36.01
34.28
31.24
21.59
9.70
8.57
tBu
O
N
Ts
f1 (ppm)

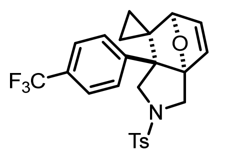

Compound 2i: Yield: 38.3 mg, 83%; A white solid; M.p.: > 200 ºC; ^{1}H NMR (400 MHz, $CDCl_3$) δ 7.78 (d, *J* = 8.0 Hz, 2H), 7.49 (d, *J* = 8.4 Hz, 2H), 7.43 (d, *J* = 8.4 Hz, 2H), 7.35 (d, *J* = 8.0 Hz, 2H), 6.71 (dd, *J* = 5.8, 1.8 Hz, 1H), 6.07 (d, *J* = 5.8 Hz, 1H), 4.23 (d, *J* = 1.8 Hz, 1H), 4.07 (d, *J* = 9.4 Hz, 1H), 3.76 - 3.68 (m, 2H), 3.59 (d, *J* = 9.4 Hz, 1H), 2.45 (s, 3H), 0.86 - 0.78 (m, 1H), 0.74 - 0.67 (m, 1H), 0.64 - 0.56 (m, 1H), 0.45 - 0.37 (m, 1H); ^{13}C NMR (100 MHz, $CDCl_3$) δ 143.7, 142.6, 138.1, 134.0, 133.8, 129.8, 129.1 (q, *J* = 32.5 Hz), 129.0, 127.5, 124.6 (q, *J* = 3.8 Hz), 124.1 (q, *J* = 272.0 Hz), 99.3, 87.4, 58.32, 58.1, 48.9, 36.3, 21.6, 9.9, 8.6; ^{19}F NMR (376 MHz, $CDCl_3$) δ -62.6; IR (neat): ν 2926, 1618, 1327, 1162, 1123, 1075, 1015, 748, 716, 666 cm^{-1}; HRMS (ESI) Calcd. for $C_{24}H_{23}NO_3SNaF_3$ $[M+Na]^+$: 462.1345, Found: 462.1353.

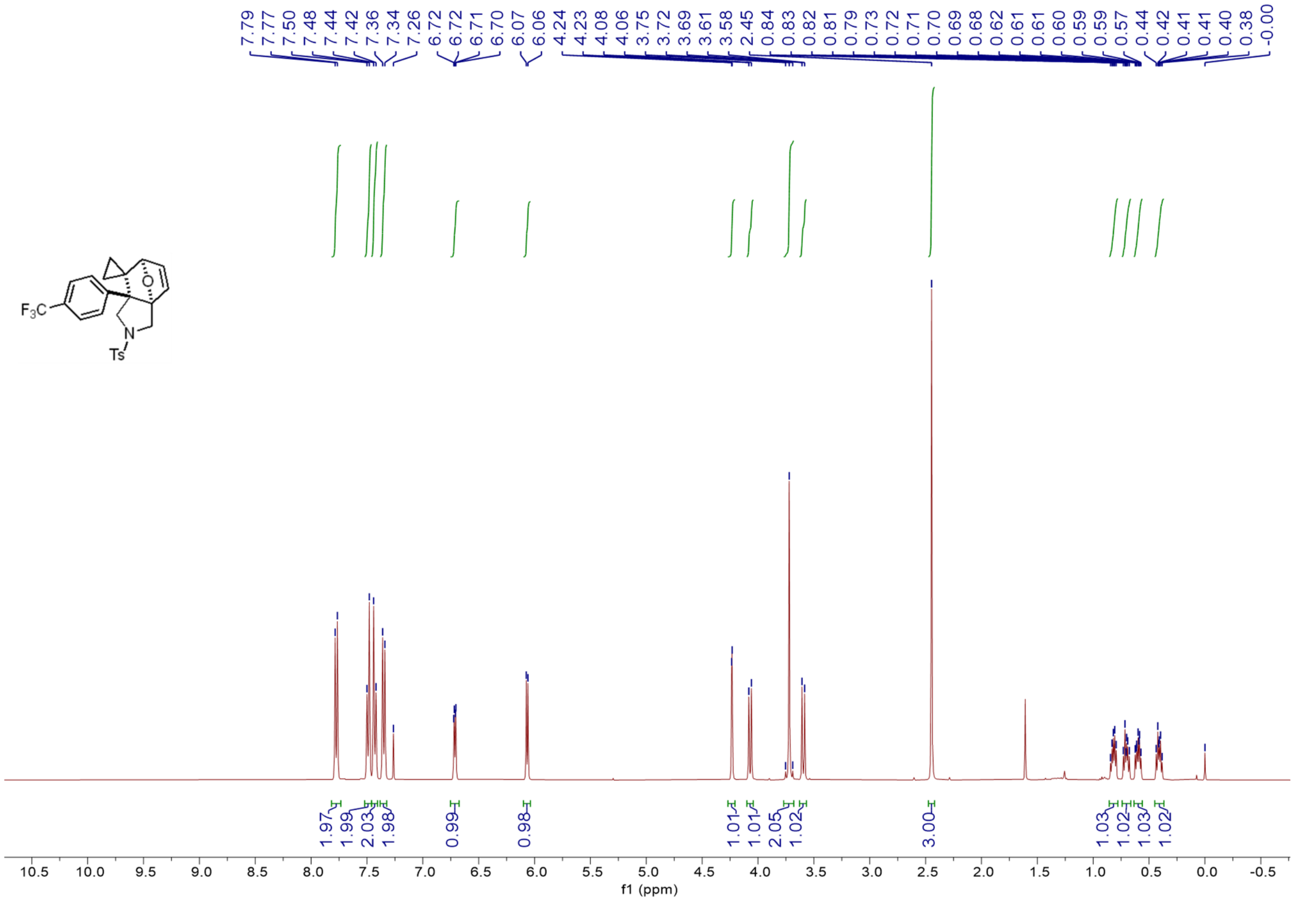
7.79 7.77 7.50 7.48 7.44 7.42 7.36 7.34 7.26 6.72 6.71 6.70 6.07 6.06 4.24 4.23 4.08 4.06 3.75 3.72 3.69 3.61 3.58 2.45 0.84 0.83 0.82 0.81 0.79 0.73 0.72 0.71 0.70 0.69 0.68 0.62 0.61 0.61 0.60 0.59 0.57 0.44 0.42 0.41 0.41 0.40 0.38 -0.00
F_3C
O
N
Ts
1.97 1.99 2.03 1.98 0.99 0.98 1.01 1.01 2.05 1.02 3.00 1.03 1.02 1.03 1.02
10.5 10.0 9.5 9.0 8.5 8.0 7.5 7.0 6.5 6.0 5.5 5.0 4.5 4.0 3.5 3.0 2.5 2.0 1.5 1.0 0.5 0.0 -0.5
f1 (ppm)

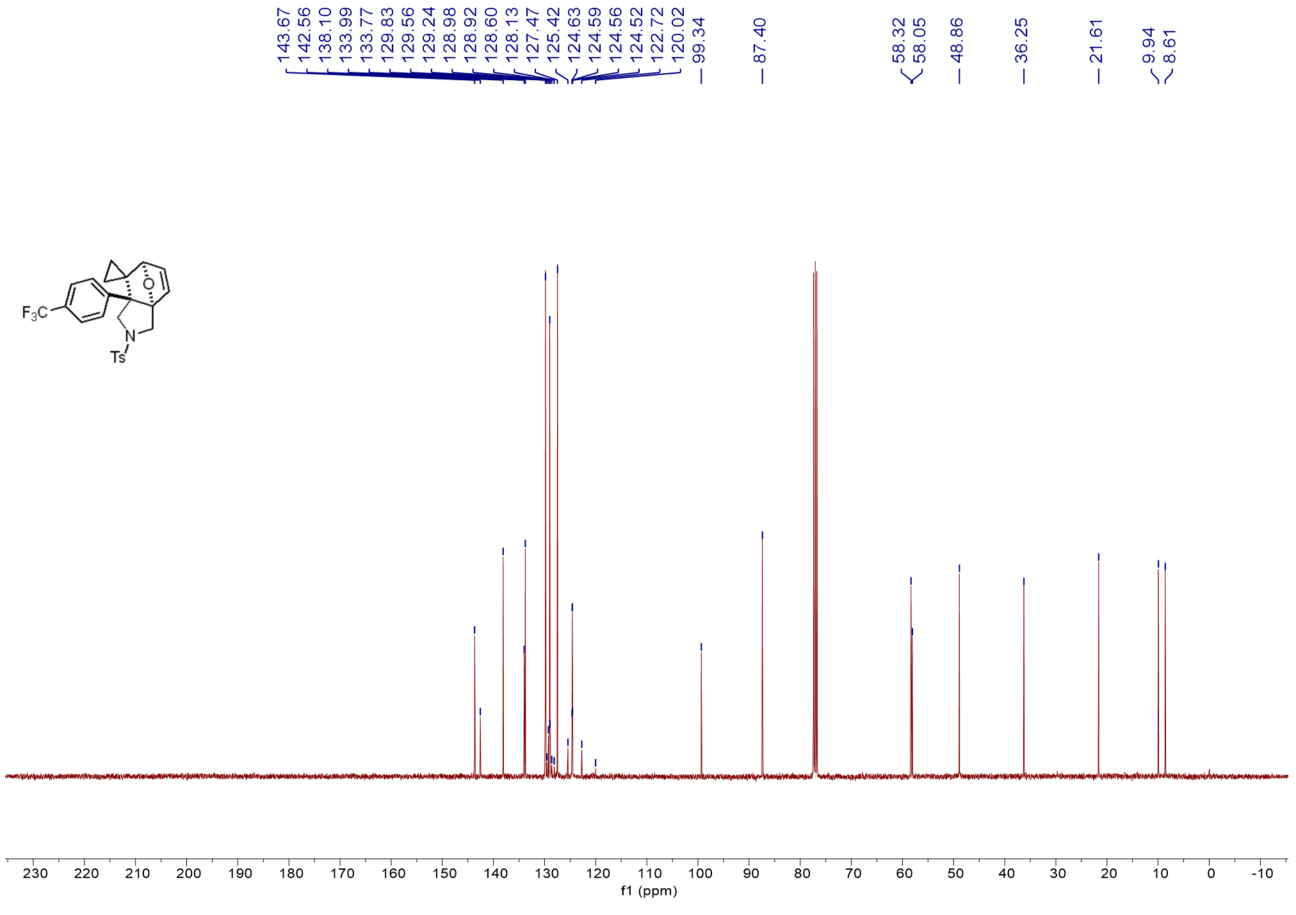
143.67 142.56 138.10 133.99 133.77 129.83 129.56 129.24 128.98 128.92 128.60 128.13 127.47 125.42 124.63 124.59 124.56 124.52 122.72 120.02 99.34 87.40 58.32 58.05 48.86 36.25 21.61 9.94 8.61
F_3C
O
N
Ts
230 220 210 200 190 180 170 160 150 140 130 120 110 100 90 80 70 60 50 40 30 20 10 0 -10
f1 (ppm)

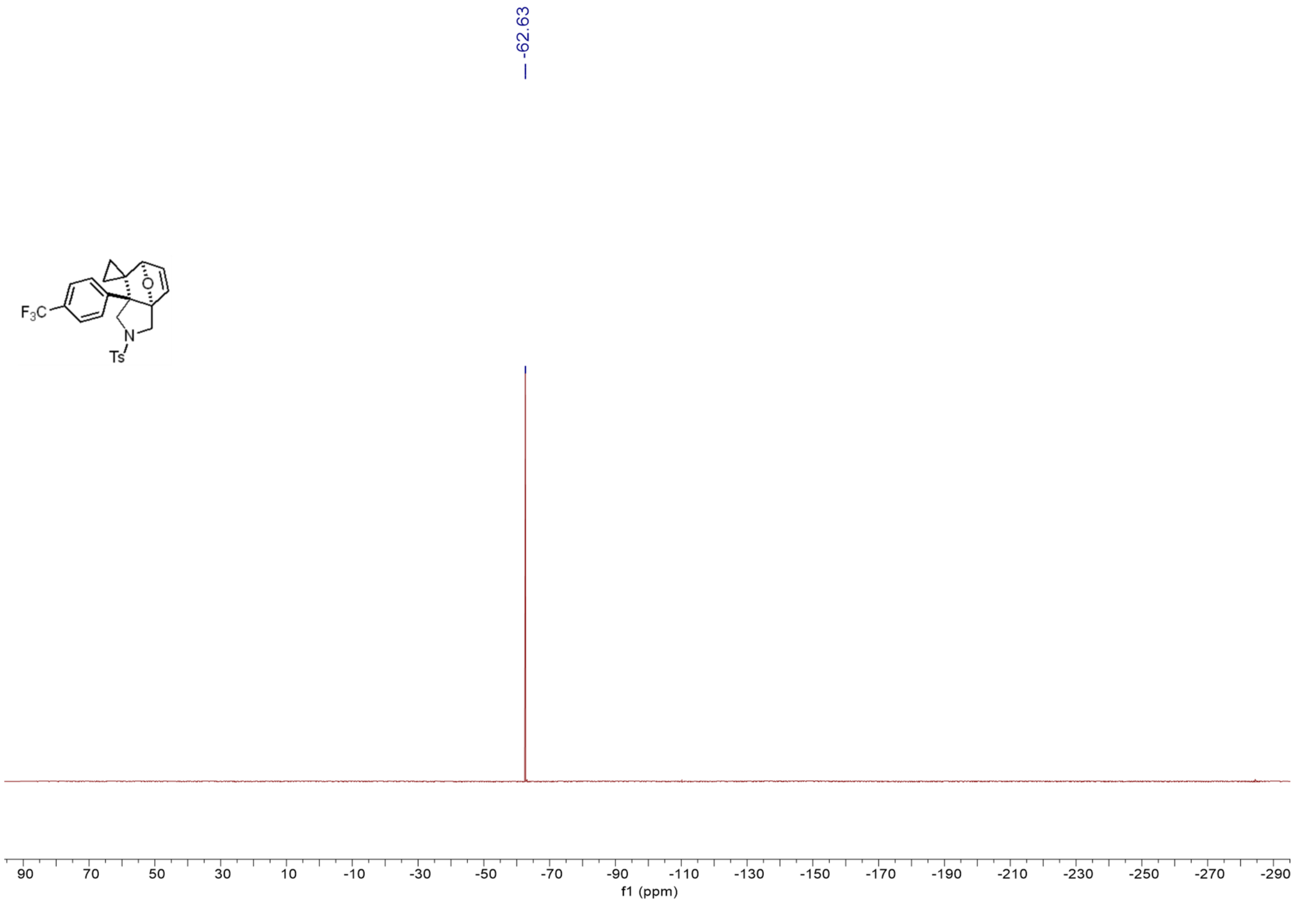
-62.63
F3C
O
N
Ts
90 70 50 30 10 -10 -30 -50 -70 -90 -110 -130 -150 -170 -190 -210 -230 -250 -270 -290
f1 (ppm)

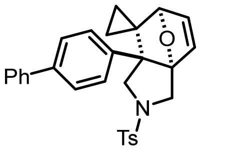

Compound 2j: Yield: 26.3 mg, 56%; A white solid; M.p.: > 200 ºC; ^{1}H NMR (400 MHz, $CDCl_3$) δ 7.79 (d, *J* = 7.8 Hz, 2H), 7.56 (d, *J* = 7.6 Hz, 2H), 7.45 (d, *J* = 7.8 Hz, 4H), 7.35 (d, *J* = 7.8 Hz, 5H), 6.72 - 6.68 (m, 1H), 6.13 (d, *J* = 5.8 Hz, 1H), 4.22 (d, *J* = 1.8 Hz, 1H), 4.11 (d, *J* = 9.2 Hz, 1H), 3.83 - 3.69 (m, 2H), 3.61 (d, *J* = 9.2 Hz, 1H), 2.43 (s, 3H), 0.84 - 0.76 (m, 1H), 0.74 - 0.65 (m, 1H), 0.64 - 0.57 (m, 1H), 0.55 - 0.47 (m, 1H); ^{13}C NMR (100 MHz, $CDCl_3$) δ 143.4, 140.4, 139.5, 137.8, 137.3, 134.2, 134.0, 129.7, 129.0, 128.8, 127.4, 127.3, 126.9, 126.3, 99.2, 87.3, 58.5, 57.7, 49.0, 36.1, 21.6, 9.8, 8.6; IR (neat): ν 2924, 1488, 1346, 1167, 1102, 761, 737, 677,655 cm^{-1}; HRMS (ESI) Calcd. for $C_{29}H_{28}NO_3SNa$ [M+Na]$^+$: 470.1784, Found: 470.1792.

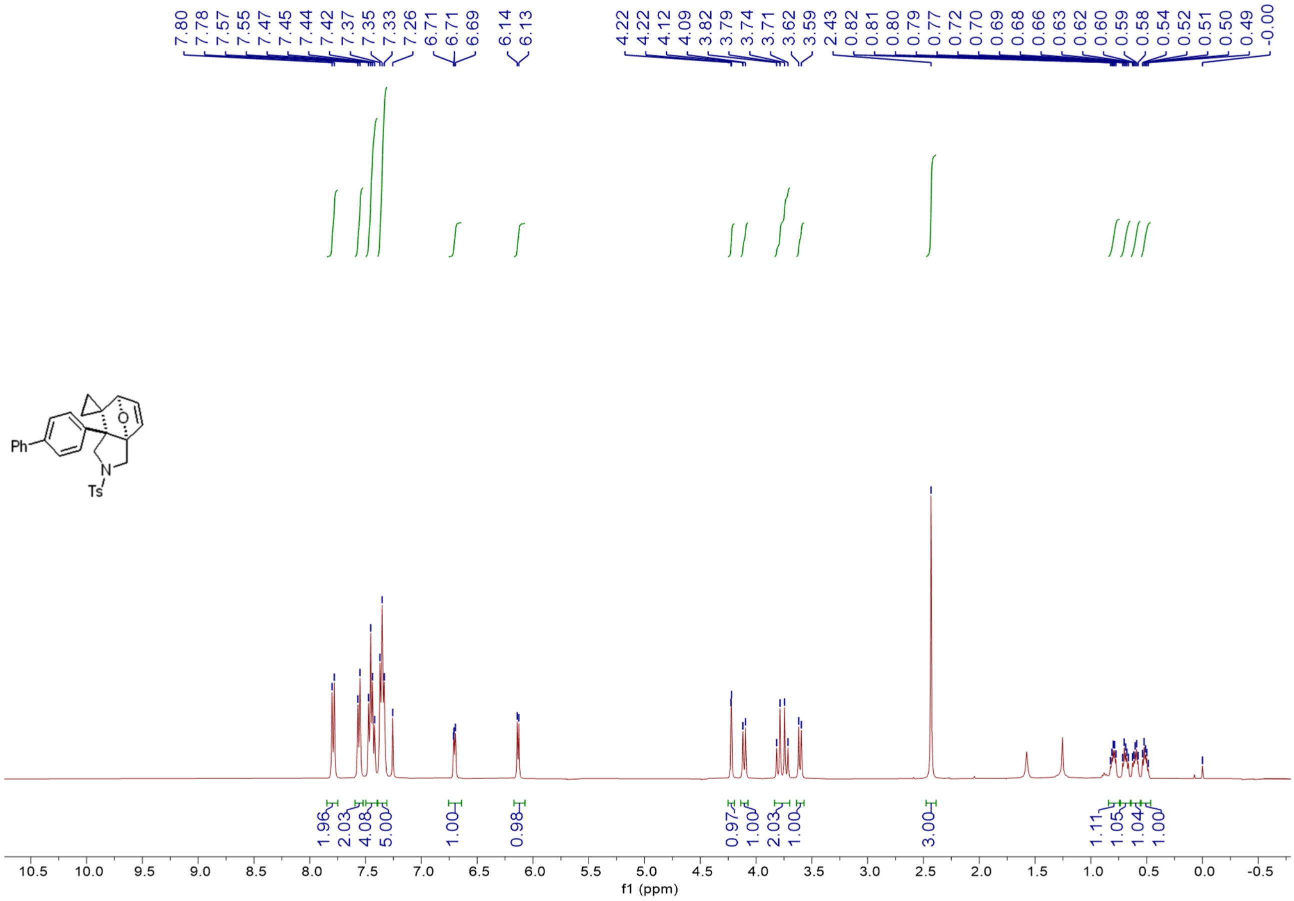
7.80
7.78
7.57
7.55
7.47
7.45
7.44
7.42
7.37
7.35
7.33
7.26
6.71
6.71
6.69
6.14
6.13
4.22
4.22
4.12
4.09
3.82
3.79
3.74
3.71
3.62
3.59
2.43
0.82
0.81
0.80
0.79
0.77
0.72
0.70
0.69
0.68
0.66
0.63
0.62
0.60
0.59
0.58
0.54
0.52
0.51
0.50
0.49
-0.00
Ph
O
N
Ts
1.96
2.03
4.08
5.00
1.00
0.98
0.97
1.00
2.03
1.00
3.00
1.11
1.05
1.04
1.00
f1 (ppm)

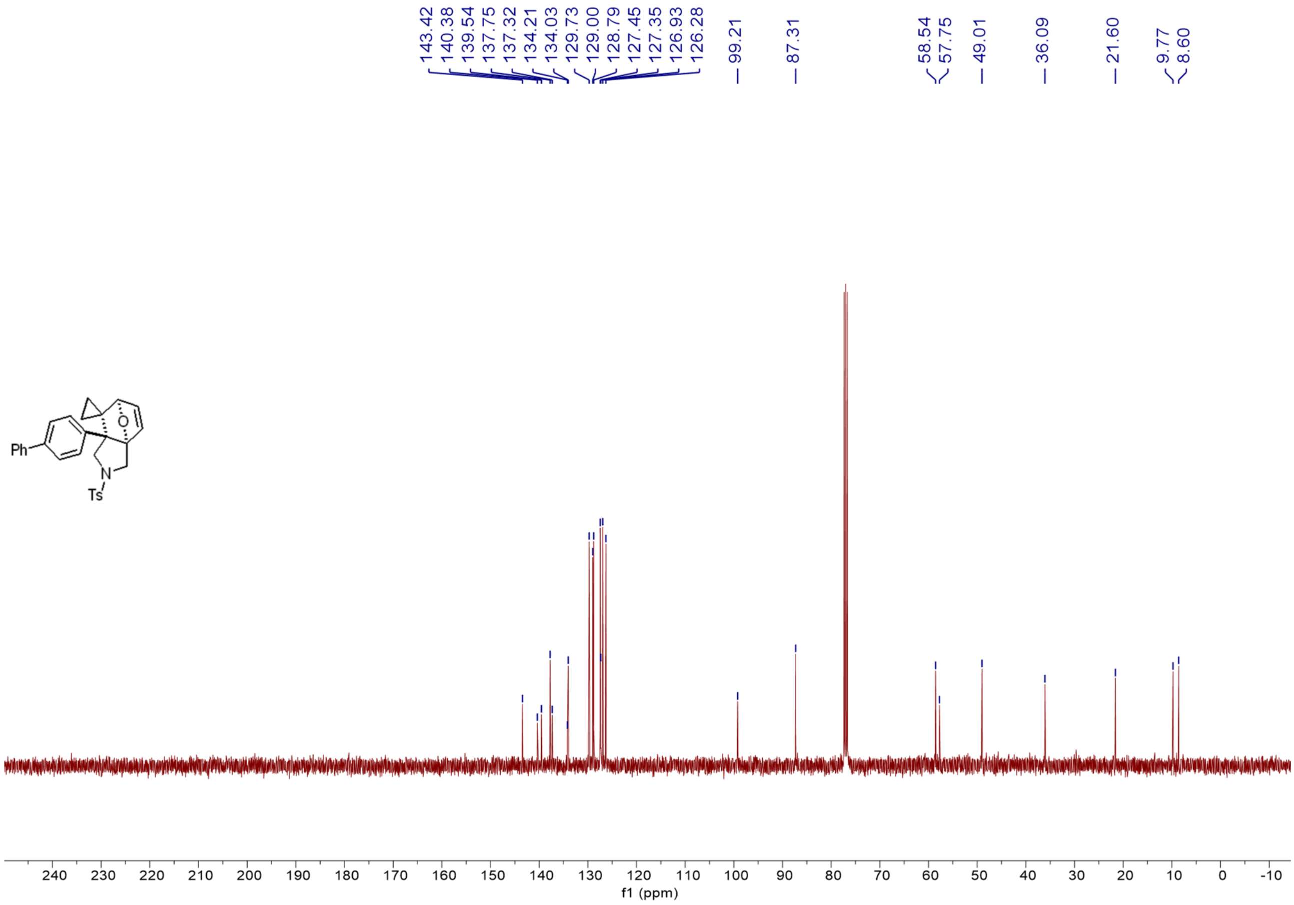
143.42
140.38
139.54
137.75
137.32
134.21
134.03
129.73
129.00
128.79
127.45
127.35
126.93
126.28
99.21
87.31
58.54
57.75
49.01
36.09
21.60
9.77
8.60
Ph
O
N
Ts
f1 (ppm)

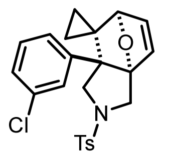

Compound 2k: Yield: 37.2 mg, 87%; A white solid; M.p.: 195 - 197 ºC; ^{1}H NMR (400 MHz, $CDCl_3$) δ 7.77 (d, *J* = 8.0 Hz, 2H), 7.34 (d, *J* = 8.0 Hz, 2H), 7.29 - 7.23 (m, 1H), 7.22 - 7.13 (m, 3H), 6.70 (d, *J* = 5.8 Hz, 1H), 6.10 (d, *J* = 5.8 Hz, 1H), 4.21 (s, 1H), 4.01 (d, *J* = 9.4 Hz, 1H), 3.73 (s, 2H), 3.58 (d, *J* = 9.4 Hz, 1H), 2.44 (s, 2H), 0.84 - 0.75 (m, 1H), 0.73 - 0.64 (m, 1H), 0.62 - 0.54 (m, 1H), 0.48 - 0.38 (m, 1H); ^{13}C NMR (100 MHz, $CDCl_3$) δ 143.5, 140.7, 138.0, 134.1, 133.7, 133.6, 129.8, 129.1, 128.9, 127.4, 127.1, 126.5, 99.1, 87.3, 58.4, 57.8, 48.9, 36.2, 21.6, 9.9, 8.5; IR (neat): ν 2992, 1712, 1595, 1475, 1343, 1162, 1102, 891, 806, 725, 676 cm^{-1}; HRMS (ESI) Calcd. for $C_{23}H_{22}NO_3SNaCl$ $[M+Na]^+$: 450.0901, Found: 450.0905.

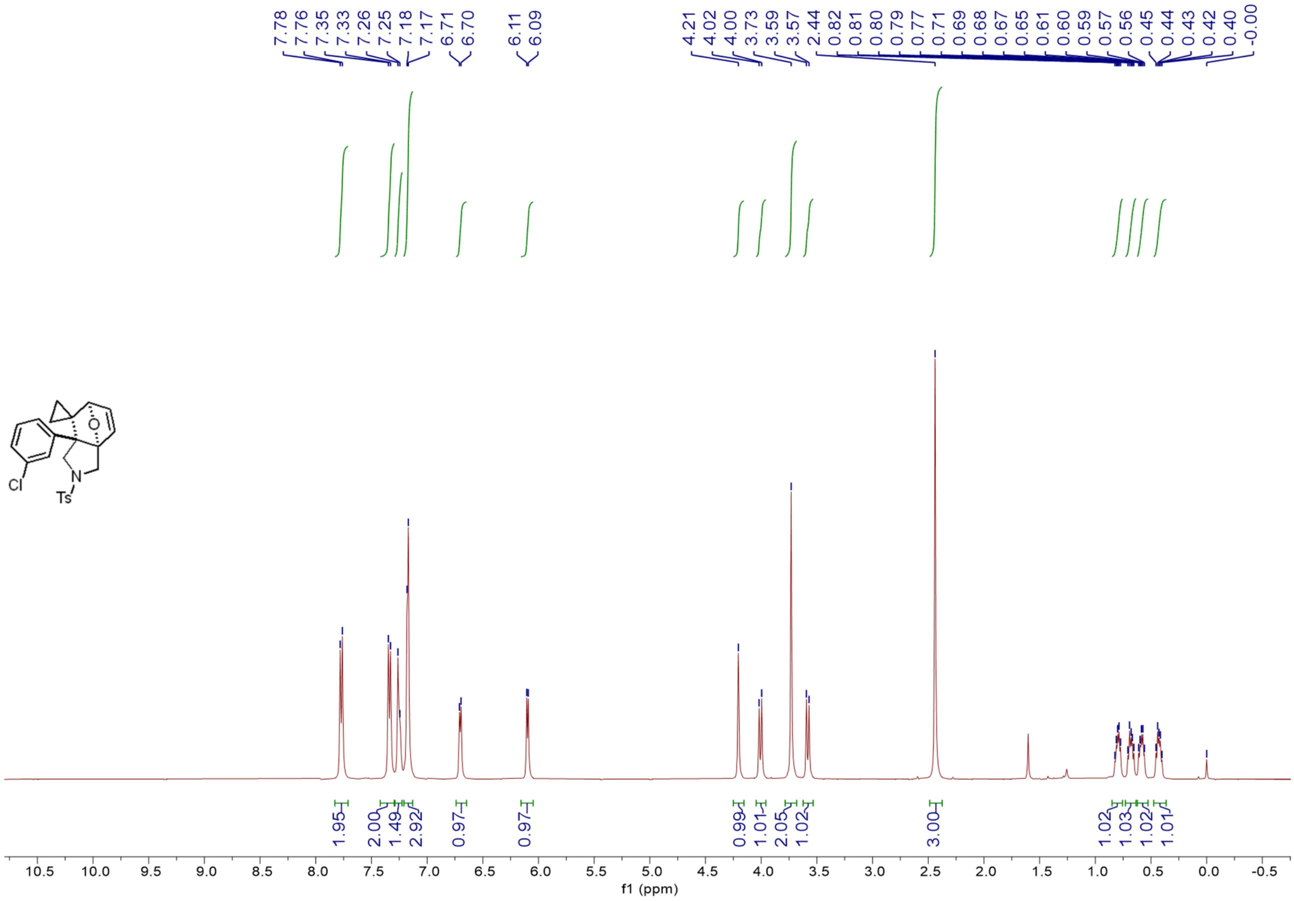

7.78
7.76
7.35
7.33
7.26
7.25
7.18
7.17
6.71
6.70
6.11
6.09
4.21
4.02
4.00
3.73
3.59
3.57
2.44
0.82
0.81
0.80
0.79
0.77
0.71
0.69
0.68
0.67
0.65
0.61
0.60
0.59
0.57
0.56
0.45
0.44
0.43
0.42
0.40
-0.00
Cl
N
Ts
O
1.95
2.00
1.49
2.92
0.97
0.97
0.99
1.01
2.05
1.02
3.00
1.02
1.03
1.02
1.01
f1 (ppm)

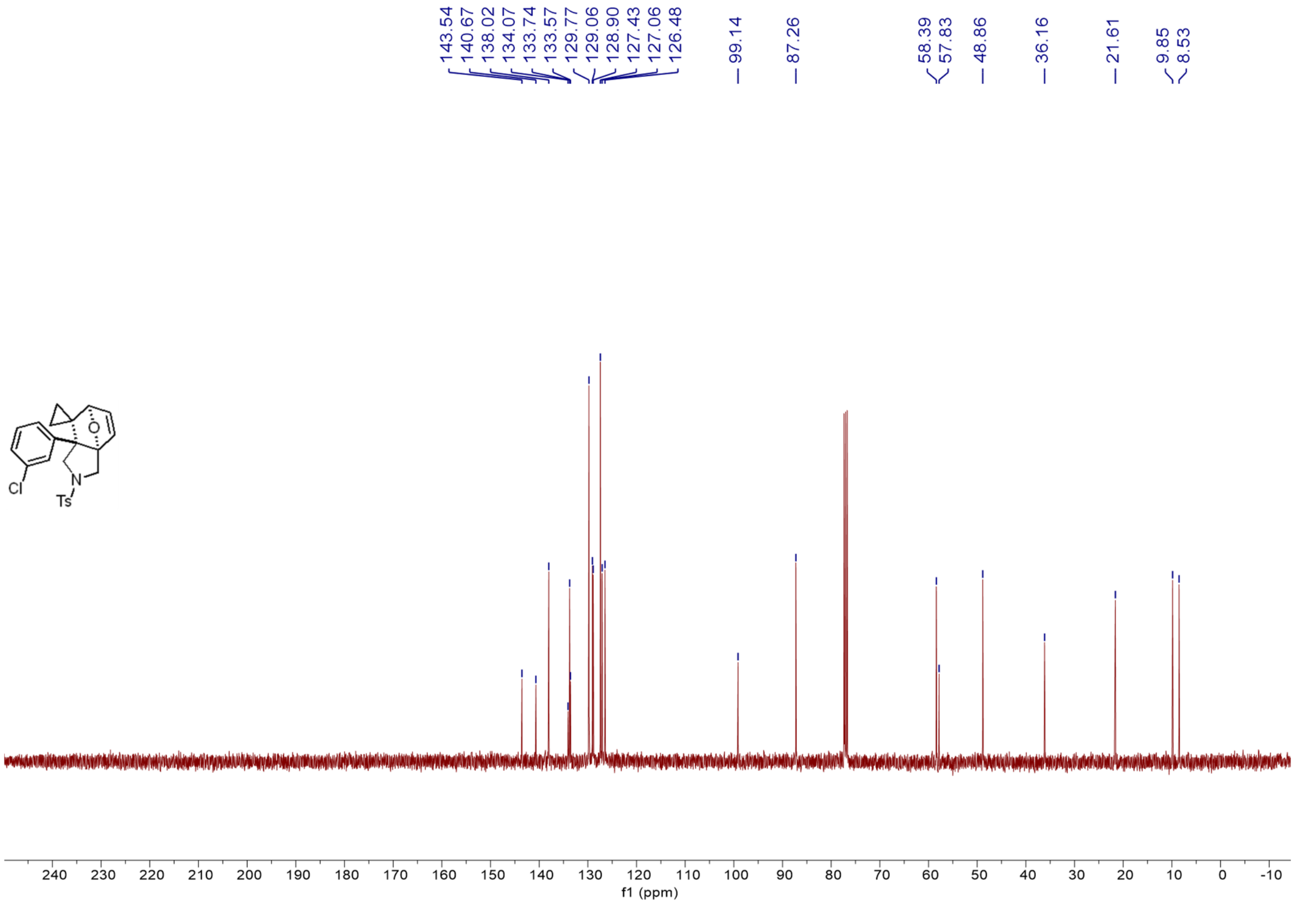

143.54
140.67
138.02
134.07
133.74
133.57
129.77
129.06
128.90
127.43
127.06
126.48
99.14
87.26
58.39
57.83
48.86
36.16
21.61
9.85
8.53
Cl
N
Ts
O
f1 (ppm)

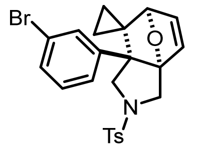

Compound 2l: Yield: 30.2 mg, 64%; A white solid; M.p.: 194 - 197 ºC; ^{1}H NMR (400 MHz, $CDCl_3$) δ 7.77 (d, *J* = 8.2 Hz, 2H), 7.38 - 7.28 (m, 5H), 7.12 (d, *J* = 8.2 Hz, 1H), 6.70 (dd, *J* = 5.8, 1.8 Hz, 1H), 6.10 (d, *J* = 5.8 Hz, 1H), 4.21 (d, *J* = 1.8 Hz, 1H), 4.00 (d, *J* = 9.4 Hz, 1H), 3.73 (s, 2H), 3.58 (d, *J* = 9.4 Hz, 1H), 2.44 (s, 3H), 0.83 - 0.76 (m, 1H), 0.72 - 0.64 (m, 1H), 0.62 - 0.54 (m, 1H), 0.47 - 0.38 (m, 1H); ^{13}C NMR (100 MHz, $CDCl_3$) δ 143.5, 140.9, 138.0, 134.1, 133.7, 131.8, 130.0, 129.8, 129.4, 127.4, 126.9, 121.8, 99.1, 87.3, 58.4, 57.8, 48.9, 36.2, 21.6, 9.9, 8.5; IR (neat): ν 2961, 1712, 1595, 1560, 1474, 1343, 1161, 1100, 1040, 801, 714, 761 cm^{-1}; HRMS (ESI) Calcd. for $C_{23}H_{22}NO_3SNaBr$ $[M+Na]^+$: 494.0396, Found: 494.0389.

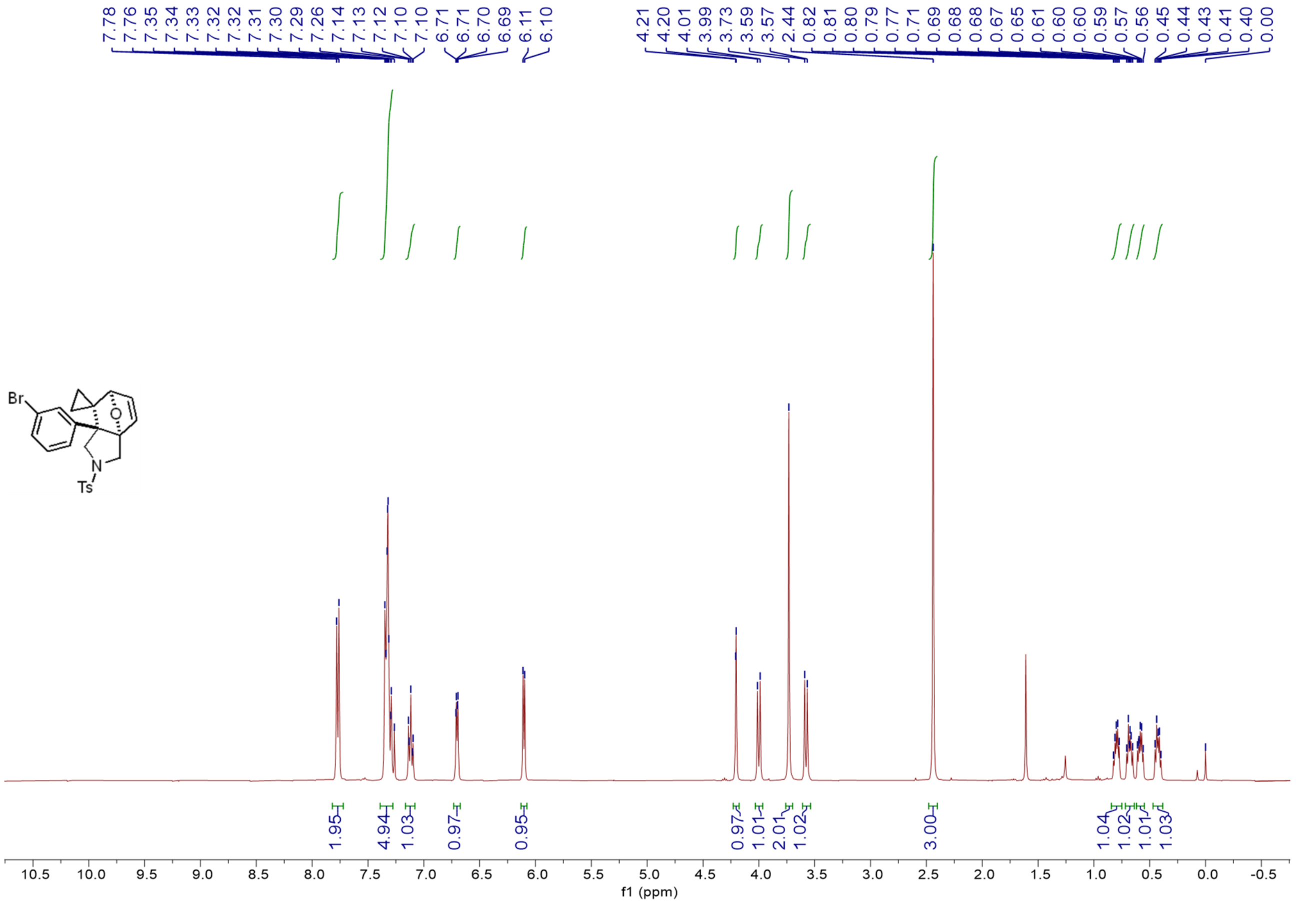
7.78 7.76 7.35 7.34 7.33 7.32 7.32 7.31 7.30 7.29 7.26 7.14 7.13 7.12 7.10 7.10 6.71 6.71 6.70 6.69 6.11 6.10
4.21 4.20 4.01 3.99 3.73 3.59 3.57 2.44 0.82 0.81 0.80 0.79 0.77 0.71 0.69 0.68 0.67 0.65 0.61 0.60 0.59 0.57 0.56 0.45 0.44 0.43 0.41 0.40 0.00
Br
O
N
Ts
1.95 4.94 1.03 0.97 0.95 0.97 1.01 2.01 1.02 3.00 1.04 1.02 1.01 1.03
f1 (ppm)

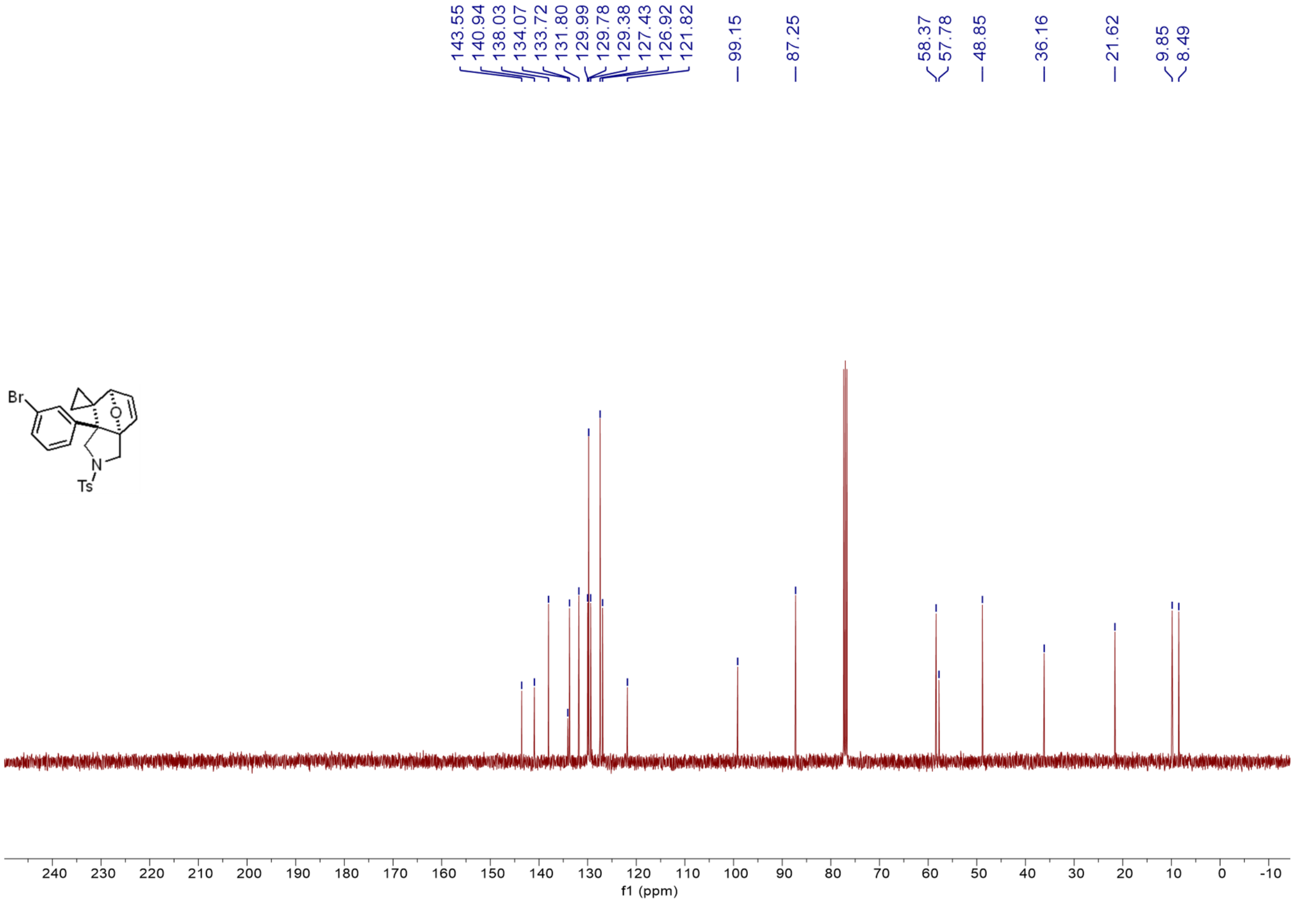
143.55 140.94 138.03 134.07 133.72 131.80 129.99 129.78 129.38 127.43 126.92 121.82 99.15 87.25 58.37 57.78 48.85 36.16 21.62 9.85 8.49
Br
O
N
Ts
f1 (ppm)

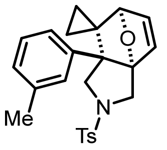

Compound 2m: Yield: 30.2 mg, 74%; A white solid; M.p.: > 200 ºC; ^{1}H NMR (400 MHz, $CDCl_3$) δ 7.77 (d, *J* = 8.0 Hz, 2H), 7.33 (d, *J* = 8.0 Hz, 2H), 7.15 - 7.09 (m, 1H), 7.08 - 7.03 (m, 2H), 7.02 - 6.98 (m, 1H), 6.66 (dd, *J* = 5.8, 1.8 Hz, 1H), 6.09 (d, *J* = 5.8 Hz, 1H), 4.19 (d, *J* = 1.8 Hz, 1H), 4.05 (d, *J* = 9.2 Hz, 1H), 3.80 - 3.70 (m, 2H), 3.59 (d, *J* = 9.2 Hz, 1H), 2.43 (s, 3H), 2.29 (s, 3H), 0.82 - 0.73 (m, 1H), 0.69 - 0.61 (m, 1H), 0.60 - 0.53 (m, 1H), 0.50 - 0.42 (m, 1H); ^{13}C NMR (100 MHz, $CDCl_3$) δ 143.3, 138.3, 137.7, 137.2, 134.4, 134.0, 129.7, 129.0, 127.5, 127.4, 125.8, 99.0, 87.2, 58.7, 57.8, 49.0, 36.0, 21.7, 21.6, 9.8, 8.6; IR (neat): ν 2924, 1600, 1342, 1161, 1106, 978, 893, 741, 707, 681 cm^{-1}; HRMS (ESI) Calcd. for $C_{24}H_{25}NO_3SNa$ $[M+Na]^+$: 430.1447, Found: 430.1442.

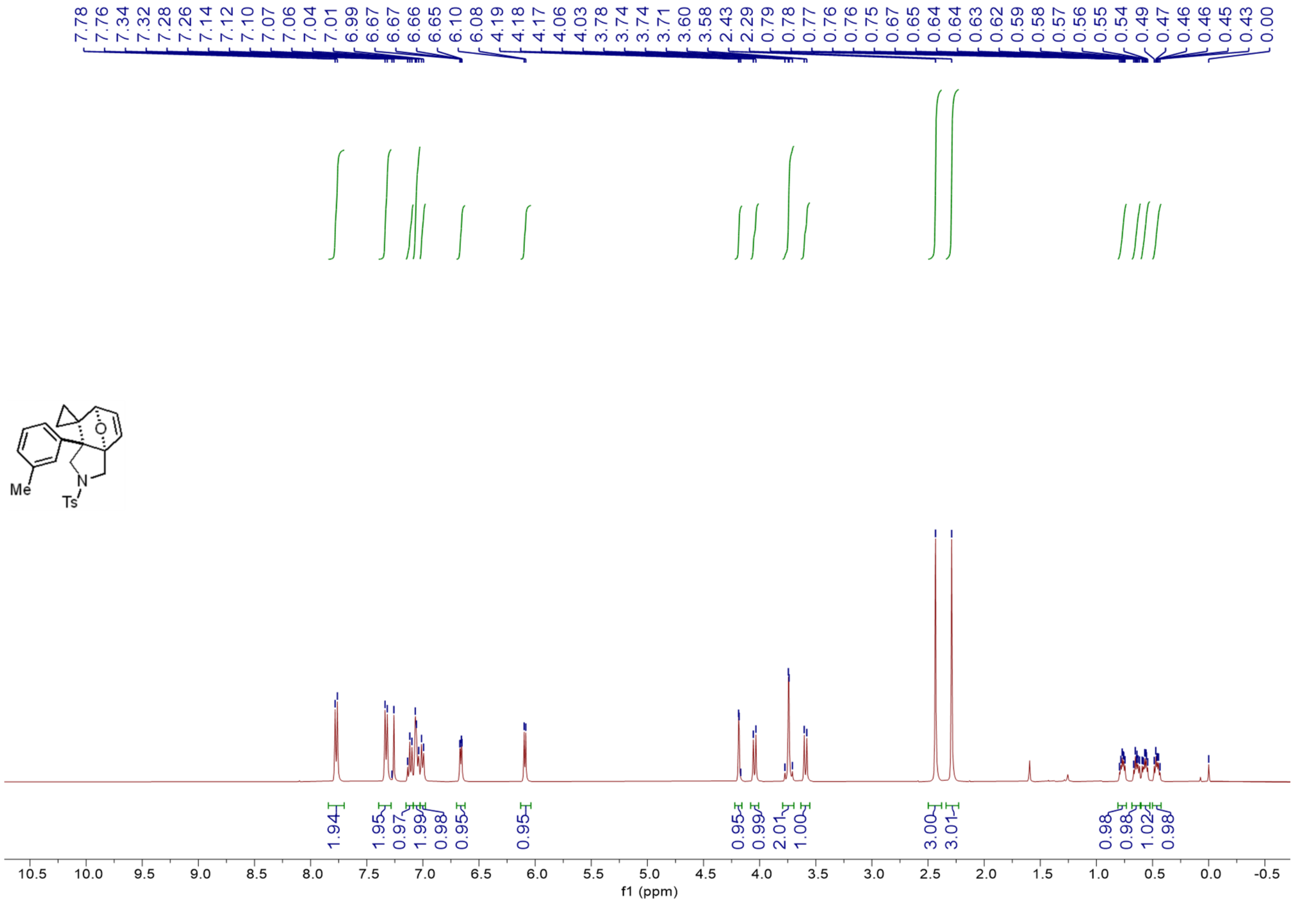
7.78 7.76 7.34 7.32 7.28 7.26 7.14 7.12 7.10 7.07 7.06 7.04 7.01 6.99 6.67 6.67 6.66 6.65 6.10 6.08 4.19 4.18 4.17 4.06 4.03 3.78 3.74 3.74 3.71 3.60 3.58 2.43 2.29 0.79 0.78 0.77 0.76 0.76 0.75 0.67 0.65 0.64 0.64 0.63 0.62 0.59 0.58 0.57 0.56 0.55 0.54 0.49 0.47 0.46 0.46 0.45 0.43 0.00
O
Me
N
Ts
1.94 1.95 0.97 1.99 0.98 0.95 0.95 0.95 0.99 2.01 1.00 3.00 3.01 0.98 0.98 1.02 0.98
10.5 10.0 9.5 9.0 8.5 8.0 7.5 7.0 6.5 6.0 5.5 5.0 4.5 4.0 3.5 3.0 2.5 2.0 1.5 1.0 0.5 0.0 -0.5
f1 (ppm)

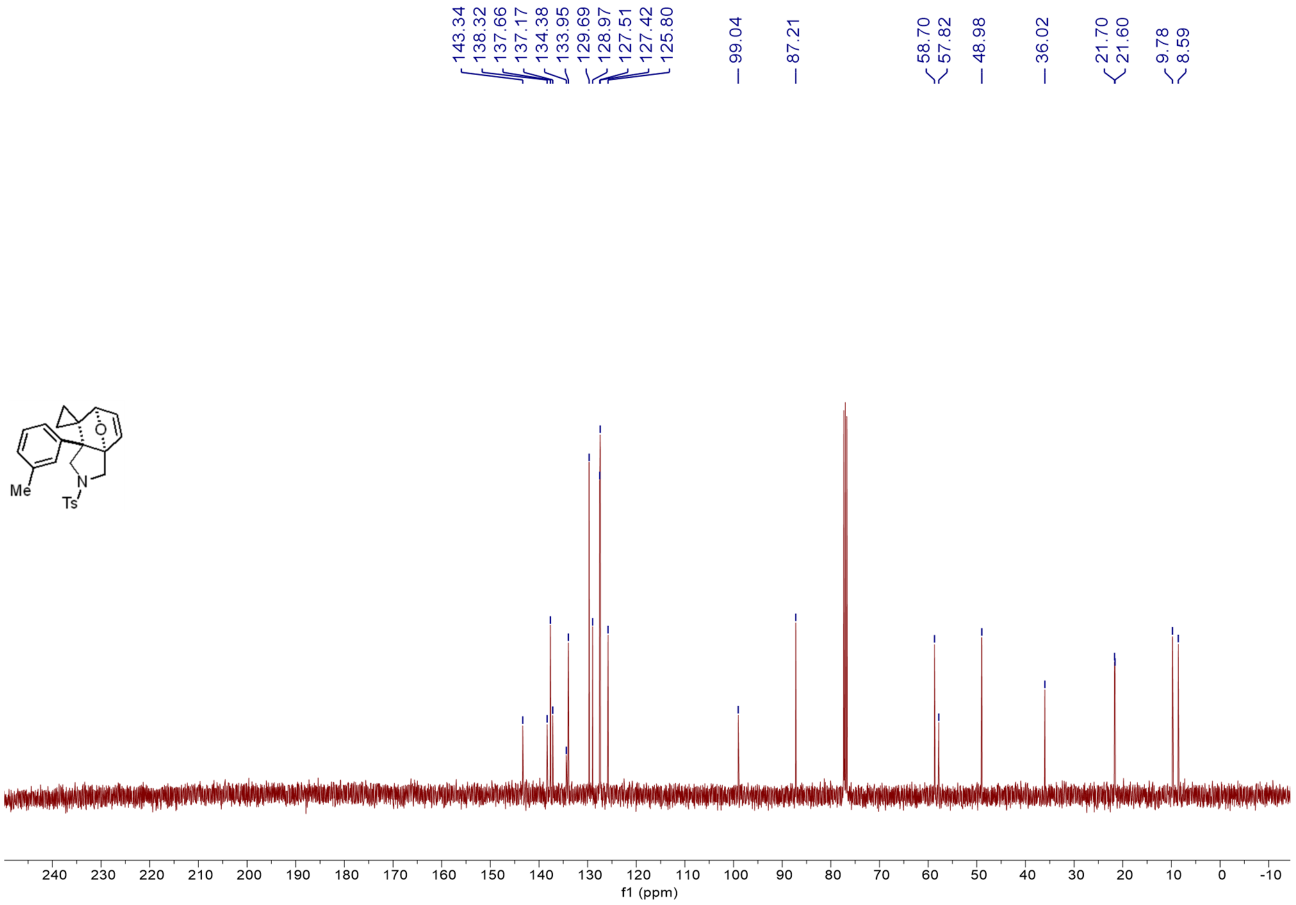
143.34 138.32 137.66 137.17 134.38 133.95 129.69 128.97 127.51 127.42 125.80 99.04 87.21 58.70 57.82 48.98 36.02 21.70 21.60 9.78 8.59
O
Me
N
Ts
240 230 220 210 200 190 180 170 160 150 140 130 120 110 100 90 80 70 60 50 40 30 20 10 0 -10
f1 (ppm)

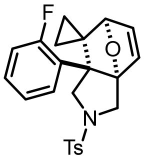

Compound 2n: Yield: 13.2 mg, 32%; A white solid; M.p.: > 200 °C; ^{1}H NMR (400 MHz, $CDCl_3$) δ 7.56 (d, *J* = 8.2 Hz, 2H), 7.18 - 7.11 (m, 3H), 6.98 - 6.81 (m, 3H), 6.74 (d, *J* = 5.8 Hz, 1H), 6.55 (dd, *J* = 5.8, 1.8 Hz, 1H), 4.12 (d, *J* = 1.8 Hz, 1H), 4.08 (d, *J* = 11.8 Hz, 1H), 3.87 (dd, *J* = 9.4, 1.8 Hz, 1H), 3.81 (d, *J* = 11.8 Hz, 1H), 3.67 (d, *J* = 9.4 Hz, 1H), 2.37 (s, 3H), 1.15 - 1.05 (m, 1H), 0.77 - 0.67 (m, 1H), 0.42 - 0.32 (m, 1H), 0.27 - 0.17 (m, 1H); ^{13}C NMR (100 MHz, $CDCl_3$) δ 161.4 (d, *J* = 245.3 Hz), 143.1, 139.3, 134.0, 133.3, 129.6, 128.6 (d, *J* = 9.0 Hz), 128.5 (d, *J* = 13.3 Hz), 127.6 (d, *J* = 5.0 Hz), 127.1, 124.0 (d, *J* = 3.2 Hz), 116.3 (d, *J* = 23.5 Hz), 98.2, 87.0, 60.9 (d, *J* = 4.9 Hz), 59.1(d, *J* = 2.6 Hz), 49.8, 33.7, 21.5, 9.2 (d, *J* = 10.1 Hz), 6.2 (d, *J* = 4.4 Hz); ^{19}F NMR (376 MHz, $CDCl_3$) δ -110.1; IR (neat): ν 2917, 1489, 1343, 1214, 1162, 1010, 901, 849, 759, 676 cm^{-1}; HRMS (ESI) Calcd. for $C_{23}H_{22}NO_3SNaF$ $[M+Na]^+$: 434.1197, Found: 434.1193.

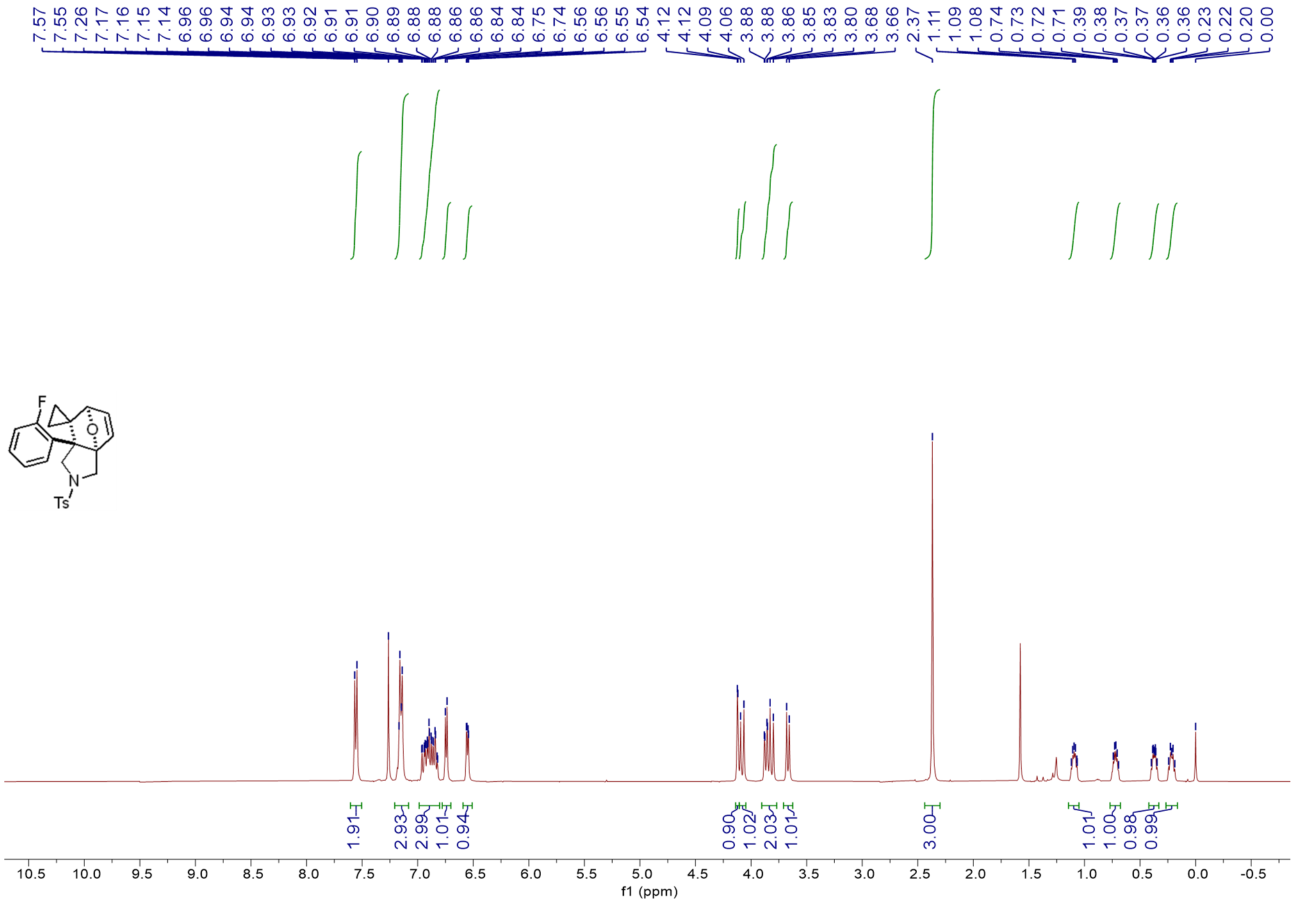
7.57 7.55 7.26 7.17 7.16 7.15 7.14 6.96 6.94 6.94 6.93 6.93 6.92 6.91 6.91 6.90 6.89 6.88 6.88 6.86 6.86 6.84 6.84 6.75 6.74 6.56 6.56 6.55 6.54 4.12 4.12 4.09 4.06 3.88 3.88 3.86 3.85 3.83 3.80 3.68 3.66 2.37 1.11 1.09 1.08 0.74 0.73 0.72 0.71 0.39 0.38 0.37 0.37 0.36 0.36 0.23 0.22 0.20 0.00
F
O
N
Ts
1.91 2.93 2.99 1.01 0.94 0.90 1.02 2.03 1.01 3.00 1.01 1.00 0.98 0.99
f1 (ppm)

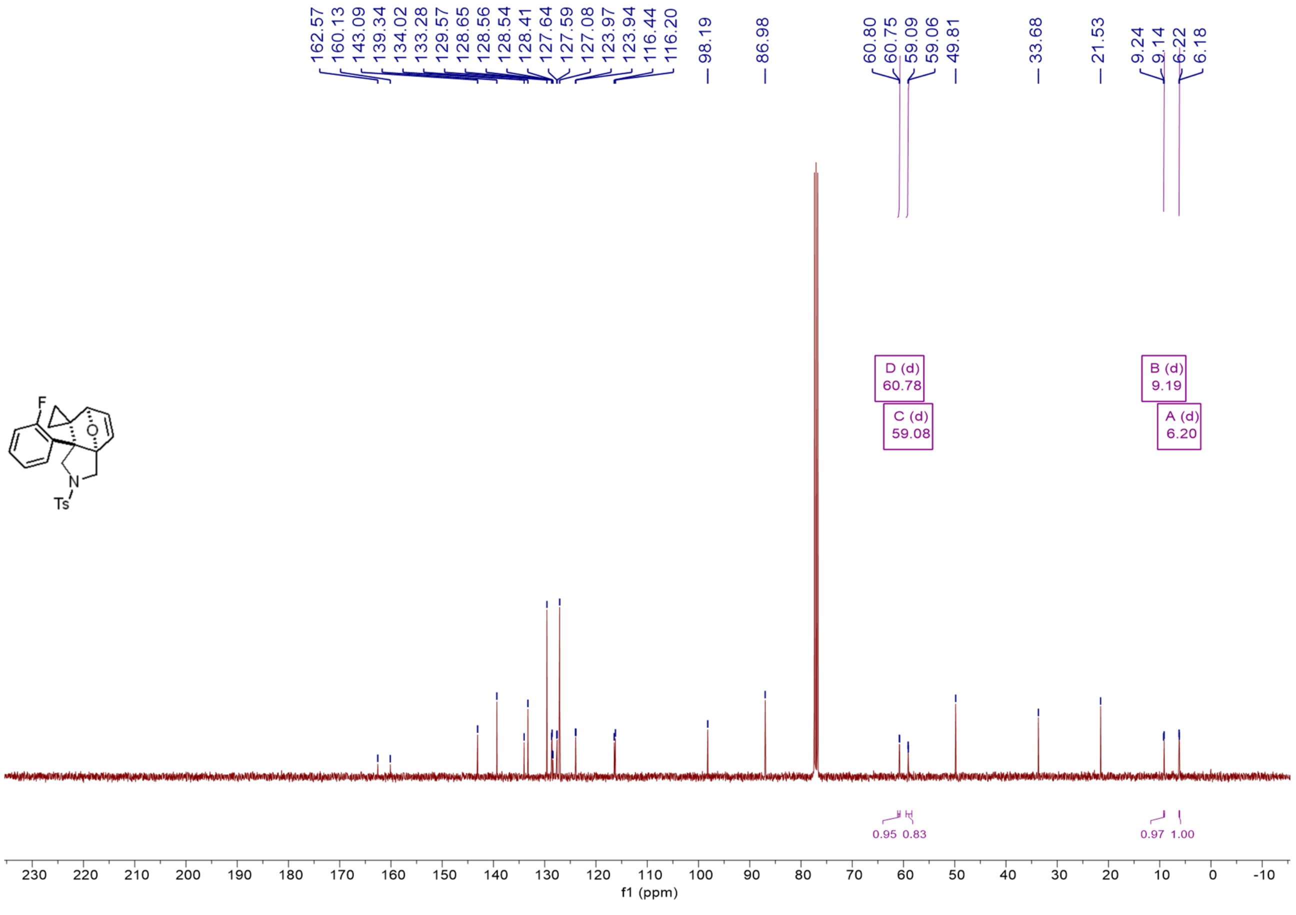
162.57 160.13 143.09 139.34 134.02 133.28 129.57 128.65 128.56 128.54 128.41 127.64 127.59 127.08 123.97 123.94 116.44 116.20 98.19 86.98 60.80 60.75 59.09 59.06 49.81 33.68 21.53 9.24 9.14 6.22 6.18
D (d) 60.78
C (d) 59.08
B (d) 9.19
A (d) 6.20
F
O
N
Ts
0.95 0.83 0.97 1.00
f1 (ppm)

-110.13

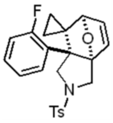

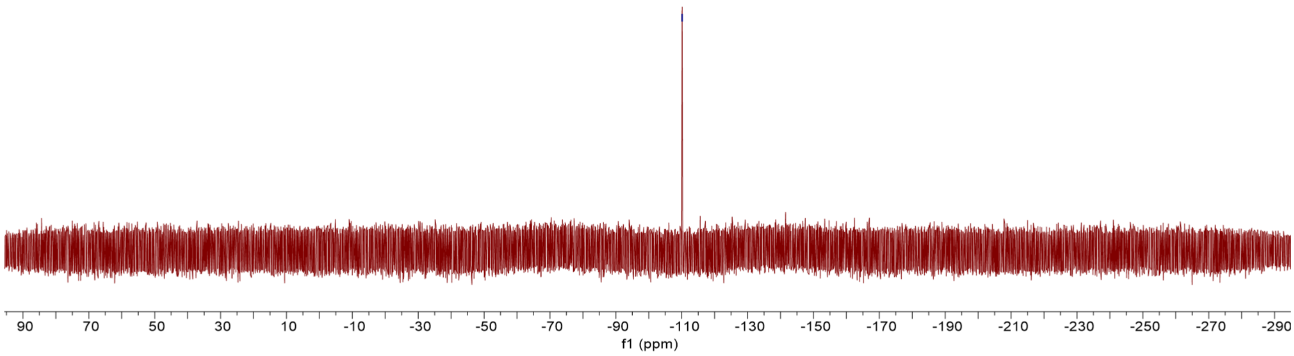

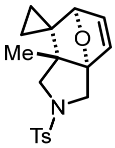

Compound 2o: Yield: 32.5 mg, 98%; A white solid; M.p.: 161 - 163 ºC; ^{1}H NMR (400 MHz, $CDCl_3$) δ 7.72 (d, *J* = 8.0 Hz, 2H), 7.31 (d, *J* = 8.0 Hz, 2H), 6.47 (dd, *J* = 5.8, 1.6 Hz, 1H), 6.34 (d, *J* = 5.8 Hz, 1H), 4.10 (s, 1H), 3.93 (d, *J* = 12.0 Hz, 1H), 3.65 (d, *J* = 12.0 Hz, 1H), 3.26 - 3.15 (m, 2H), 2.41 (s, 3H), 0.73 - 0.64 (m, 4H), 0.53 - 0.44 (m, 1H), 0.41 - 0.33 (m, 1H), 0.33 - 0.25 (m, 1H); ^{13}C NMR (100 MHz, $CDCl_3$) δ 143.4, 138.0, 134.2, 132.7, 129.7, 127.4, 97.9, 86.2, 59.3, 50.8, 49.3, 32.6, 21.6, 19.7, 7.4, 4.6; IR (neat): ν 2962, 2923, 2852, 1730, 1495, 1456, 1391, 856, 809, 754 cm^{-1}; HRMS (ESI) Calcd. for $C_{18}H_{22}NO_3S$ $[M+H]^+$: 332.1315, Found: 332.1314.

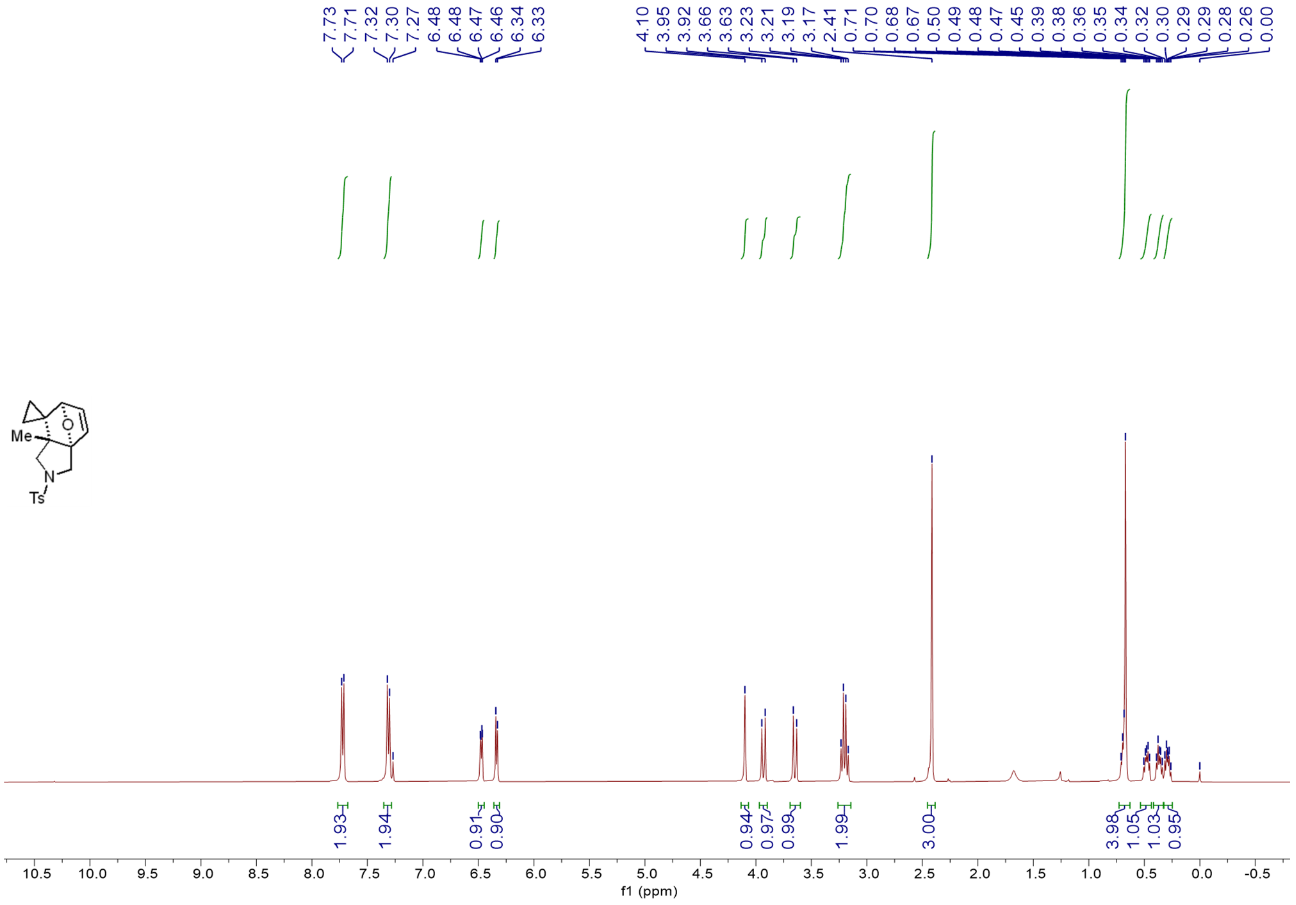
7.73 7.71 7.32 7.30 7.27 6.48 6.48 6.47 6.46 6.34 6.33
4.10 3.95 3.92 3.66 3.63 3.23 3.21 3.19 3.17 2.41 0.71 0.70 0.68 0.67 0.50 0.49 0.48 0.47 0.45 0.39 0.38 0.36 0.35 0.34 0.32 0.30 0.29 0.29 0.28 0.26 0.00
Me
O
N
Ts
1.93 1.94 0.91 0.90 0.94 0.97 0.99 1.99 3.00 3.98 1.05 1.03 0.95
f1 (ppm)

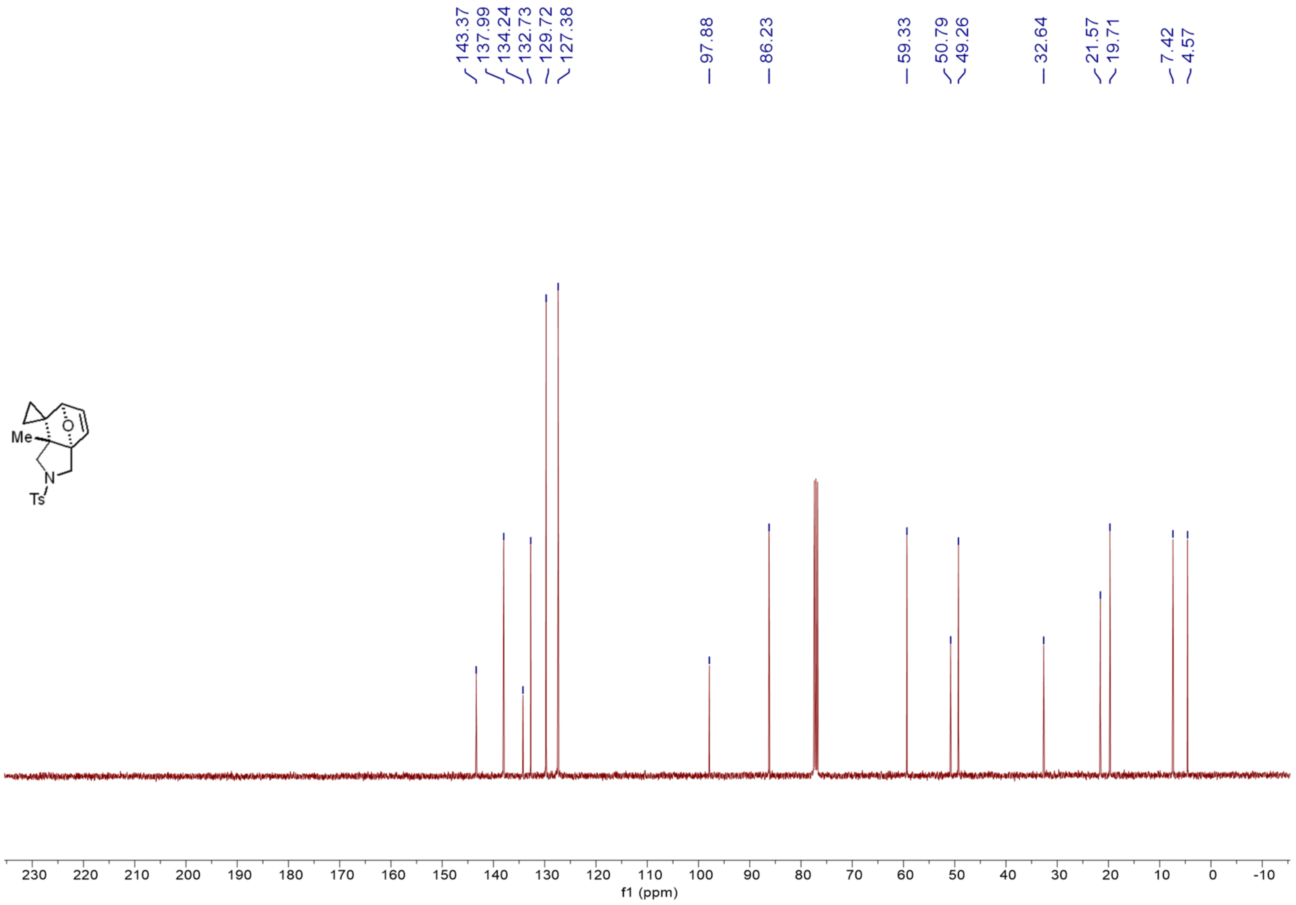
143.37 137.99 134.24 132.73 129.72 127.38
97.88
86.23
59.33 50.79 49.26
32.64
21.57 19.71
7.42 4.57
Me
O
N
Ts
f1 (ppm)

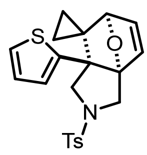

Compound 2p: Yield: 27.6 mg, 69%; A white solid; M.p.: 195 - 197 ºC; ^{1}H NMR (400 MHz, $CDCl_3$) δ 7.76 (d, *J* = 8.0 Hz, 2H), 7.33 (d, *J* = 8.0 Hz, 2H), 7.14 (dd, *J* = 5.0, 1.2 Hz, 1H), 6.93 - 6.83 (m, 2H), 6.67 (dd, *J* = 5.8, 1.8 Hz, 1H), 6.31 (d, *J* = 5.8 Hz, 1H), 4.21 (d, *J* = 1.8 Hz, 1H), 4.04 (d, *J* = 9.2 Hz, 1H), 3.86 (d, *J* = 12.0 Hz, 1H), 3.67 (d, *J* = 12.0 Hz, 1H), 3.56 (d, *J* = 9.2 Hz, 1H), 2.43 (s, 3H), 0.85 - 0.74 (m, 1H), 0.68 - 0.47 (m, 3H); ^{13}C NMR (100 MHz, $CDCl_3$) δ 143.4, 143.2, 138.4, 134.4, 134.3, 129.7, 127.4, 126.5, 126.2, 124.9, 98.8, 87.1, 59.4, 57.8, 48.7, 37.3, 21.6, 9.7, 8.3; IR (neat): ν 2991, 1711, 1598, 1342, 1160, 1109, 995, 897, 708, 670 cm^{-1}; HRMS (ESI) Calcd. for $C_{21}H_{21}NO_3S_2Na$ $[M+Na]^+$: 422.0855, Found: 422.0855.

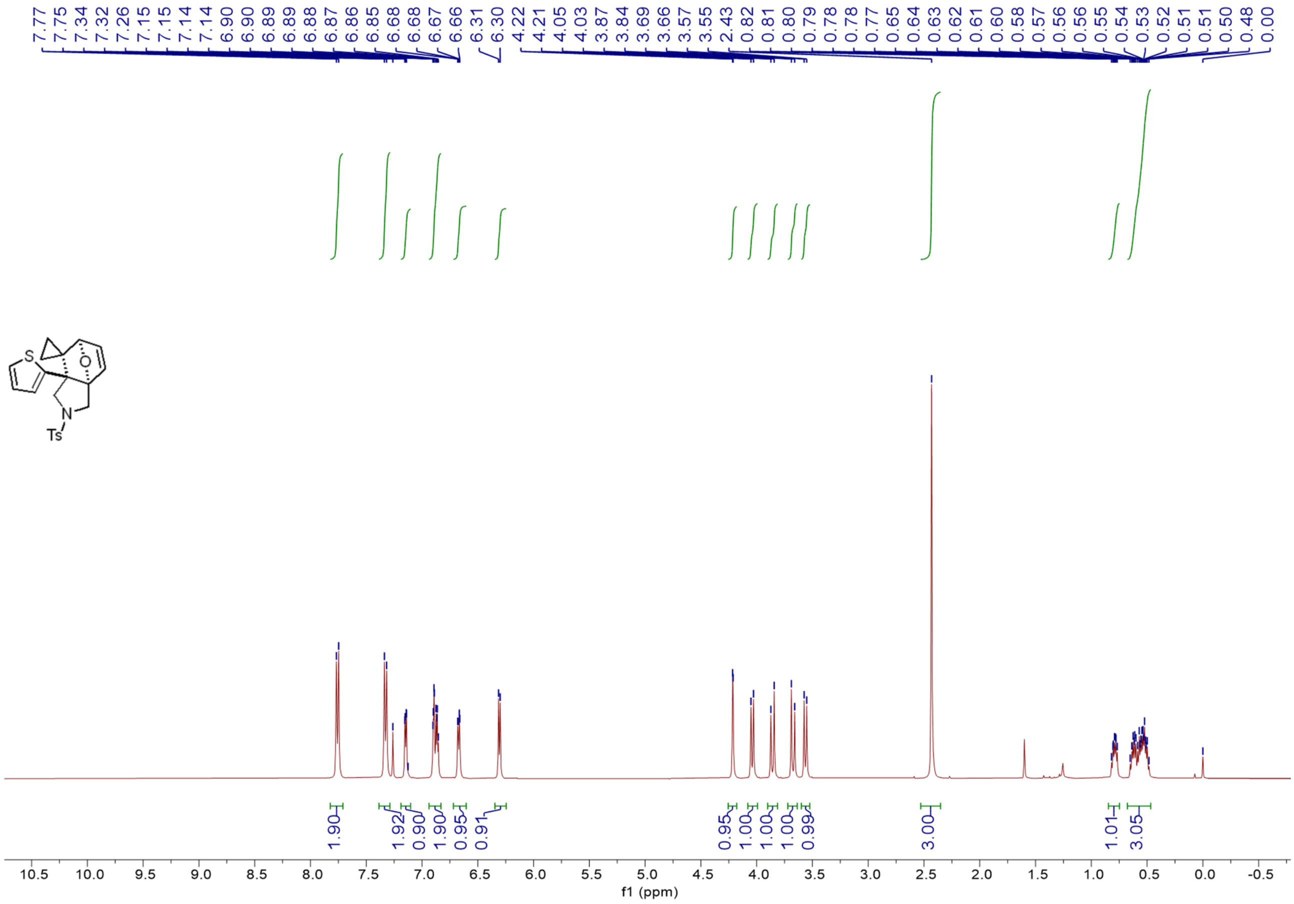
7.77 7.75 7.34 7.32 7.26 7.15 7.15 7.14 6.90 6.90 6.89 6.89 6.88 6.87 6.86 6.85 6.68 6.68 6.67 6.66 6.31 6.30 4.22 4.21 4.05 4.03 3.87 3.84 3.69 3.66 3.57 3.55 2.43 0.82 0.81 0.80 0.79 0.78 0.78 0.77 0.65 0.64 0.63 0.62 0.61 0.60 0.58 0.57 0.56 0.55 0.54 0.53 0.52 0.51 0.51 0.50 0.48 0.00
S
O
N
Ts
1.90 1.92 0.90 1.90 0.95 0.91 0.95 1.00 1.00 1.00 0.99 3.00 1.01 3.05
f1 (ppm)

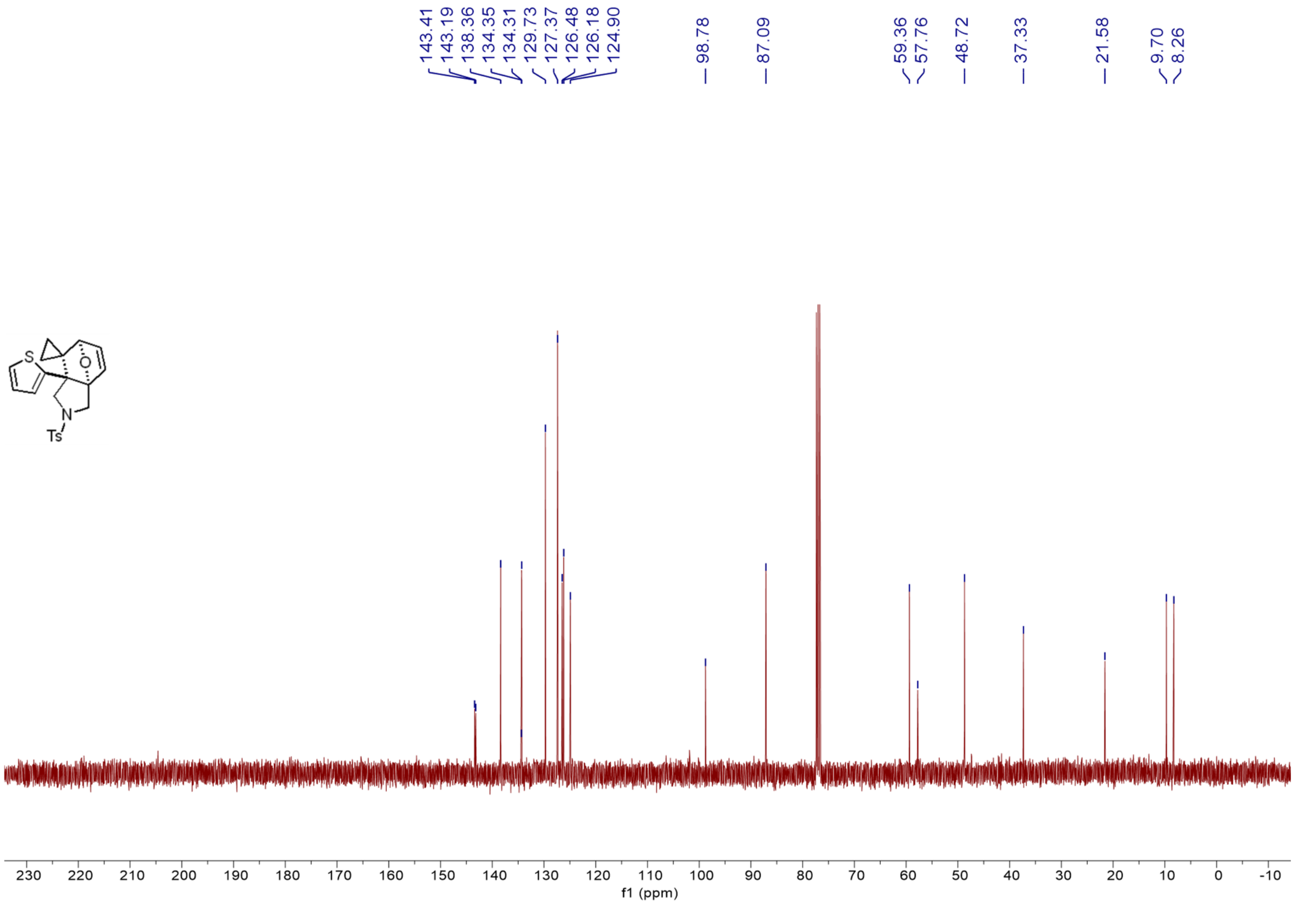
143.41 143.19 138.36 134.35 134.31 129.73 127.37 126.48 126.18 124.90 98.78 87.09 59.36 57.76 48.72 37.33 21.58 9.70 8.26
S
O
N
Ts
f1 (ppm)

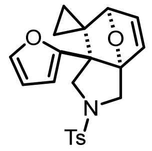

Compound 2q: Yield: 35.0 mg, 95%; A white solid; M.p.: > 200 °C; ^{1}H NMR (400 MHz, $CDCl_3$) δ 7.73 (d, *J* = 8.0 Hz, 2H), 7.31 (d, *J* = 8.0 Hz, 2H), 7.16 - 7.10 (m, 1H), 6.62 (dd, *J* = 5.8, 1.8 Hz, 1H), 6.30 (d, *J* = 5.8 Hz, 1H), 6.19 (dd, *J* = 3.4, 1.8 Hz, 1H), 6.02 (d, *J* = 3.4 Hz, 1H), 4.23 (d, *J* = 1.8 Hz, 1H), 3.98 (d, *J* = 9.2 Hz, 1H), 3.80 (d, *J* = 11.8 Hz, 1H), 3.73 (d, *J* = 11.8 Hz, 1H), 3.50 (d, *J* = 9.2 Hz, 1H), 2.43 (s, 3H), 0.80 - 0.72 (m, 1H), 0.65 - 0.56 (m, 1H), 0.45 (dt, *J* = 9.8, 6.0 Hz, 1H), 0.37 - 0.30 (m, 1H); ^{13}C NMR (100 MHz, $CDCl_3$) δ 153.1, 143.2, 141.8, 138.1, 134.6, 133.5, 129.6, 127.4, 110.1, 107.9, 98.4, 86.8, 57.1, 56.4, 49.0, 35.0, 21.6, 8.7, 6.8; IR (neat): ν 2990, 1598, 1342, 1161, 1115, 1006, 815, 742, 671 cm^{-1}; HRMS (ESI) Calcd. for $C_{21}H_{21}NO_4SNa$ $[M+Na]^+$: 406.1084, Found: 406.1083.

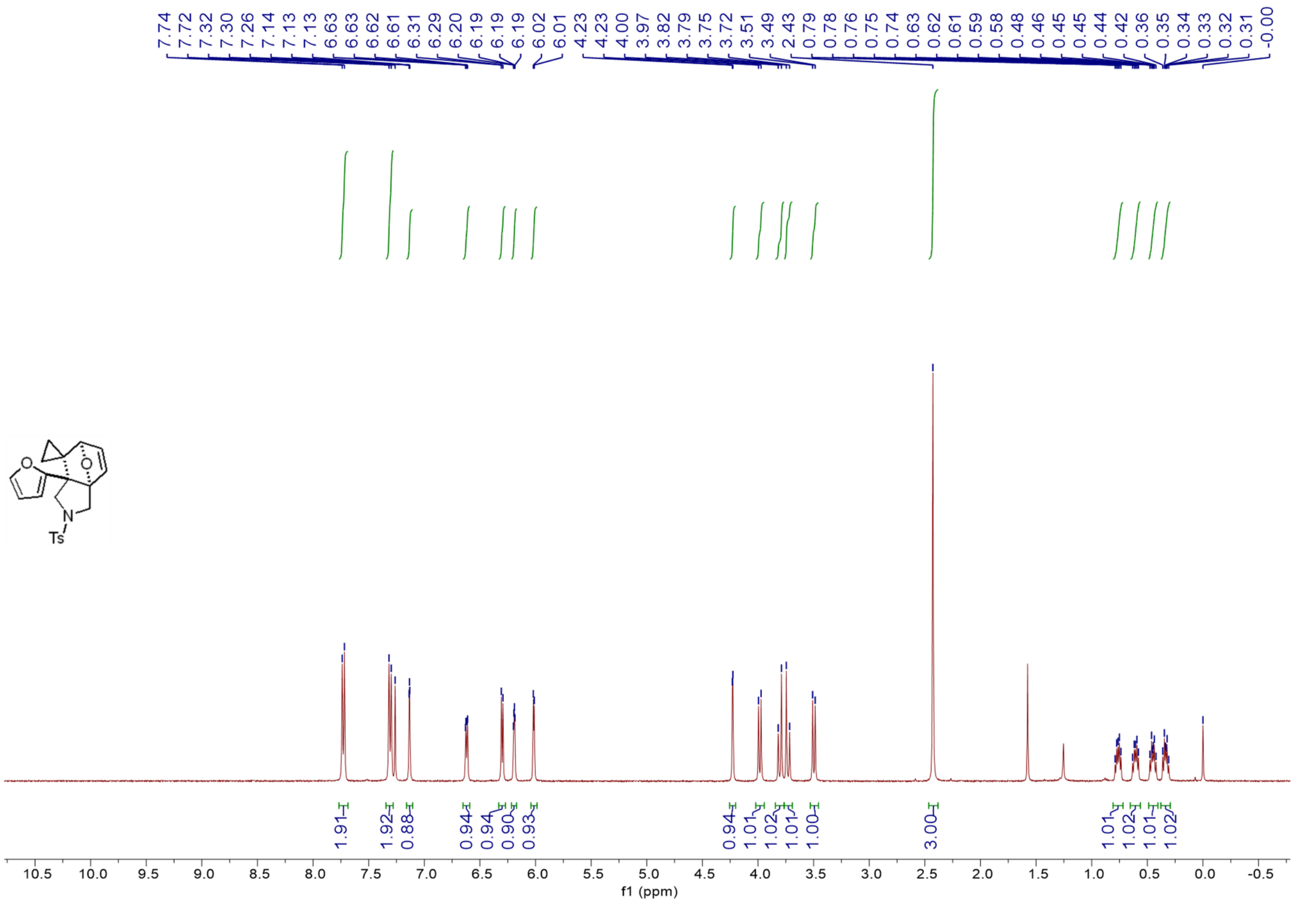
7.74 7.72 7.32 7.30 7.26 7.14 7.13 6.63 6.63 6.62 6.61 6.31 6.29 6.20 6.19 6.19 6.19 6.02 6.01 4.23 4.23 4.00 3.97 3.82 3.79 3.75 3.72 3.51 3.49 2.43 0.79 0.78 0.76 0.75 0.74 0.63 0.62 0.61 0.59 0.58 0.48 0.46 0.45 0.44 0.42 0.36 0.35 0.34 0.33 0.32 0.31 -0.00
O
N
Ts
1.91 1.92 0.88 0.94 0.94 0.90 0.93 0.94 1.01 1.02 1.01 1.00 3.00 1.01 1.02 1.01 1.02
10.5 10.0 9.5 9.0 8.5 8.0 7.5 7.0 6.5 6.0 5.5 5.0 4.5 4.0 3.5 3.0 2.5 2.0 1.5 1.0 0.5 0.0 -0.5
f1 (ppm)

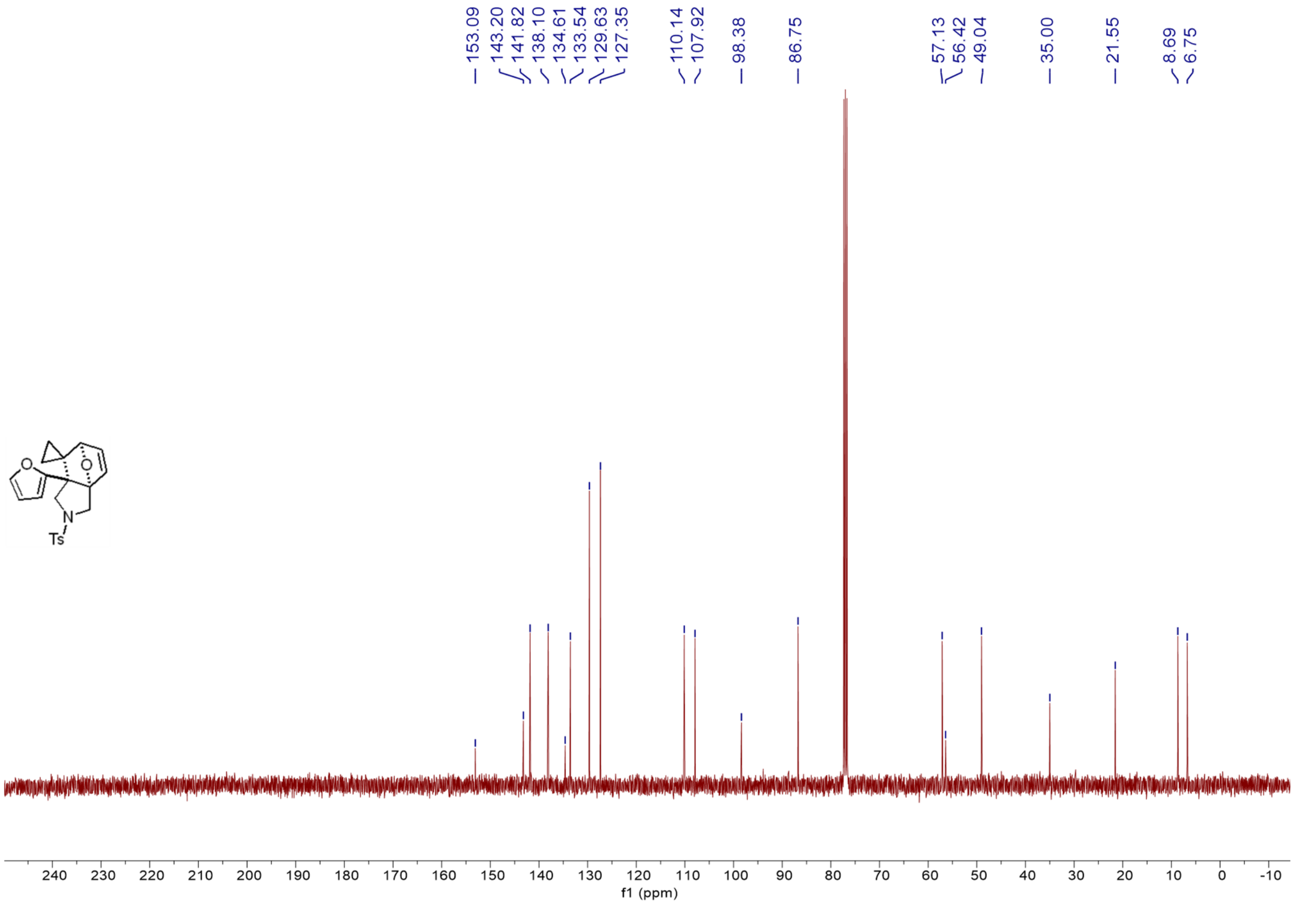
153.09 143.20 141.82 138.10 134.61 133.54 129.63 127.35 110.14 107.92 98.38 86.75 57.13 56.42 49.04 35.00 21.55 8.69 6.75
O
N
Ts
240 230 220 210 200 190 180 170 160 150 140 130 120 110 100 90 80 70 60 50 40 30 20 10 0 -10
f1 (ppm)

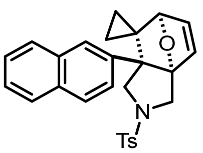

Compound 2r: Yield: 34.2 mg, 77%; A white solid; M.p.: > 200 °C; ^{1}H NMR (400 MHz, $CDCl_3$) δ 7.81 - 7.72 (m, 5H), 7.68 (d, *J* = 8.8 Hz, 1H), 7.48 - 7.42 (m, 2H), 7.36 - 7.28 (m, 3H), 6.73 (dd, *J* = 5.8, 1.8 Hz, 1H), 6.11 (d, *J* = 5.8 Hz, 1H), 4.25 (d, *J* = 1.8 Hz, 1H), 4.20 (d, *J* = 9.2 Hz, 1H), 3.78 (s, 2H), 3.70 (d, *J* = 9.2 Hz, 1H), 2.41 (s, 3H), 0.85 - 0.78 (m, 1H), 0.74 - 0.67 (m, 1H), 0.66 - 0.58 (m, 1H), 0.54 - 0.46 (m, 1H); ^{13}C NMR (100 MHz, $CDCl_3$) δ 143.4, 137.9, 135.8, 134.4, 134.0, 132.7, 132.0, 129.7, 128.3, 127.4, 127.3, 127.1, 126.9, 126.8, 126.2, 126.0, 99.3, 87.3, 58.8, 58.1, 49.0, 36.0, 21.6, 9.7, 8.6; IR (neat): ν 2991, 1710, 1598, 1342, 1161, 816, 748, 678 cm^{-1}; HRMS (ESI) Calcd. for $C_{27}H_{25}NO_3SNa$ $[M+Na]^+$: 466.1447, Found: 466.1450.

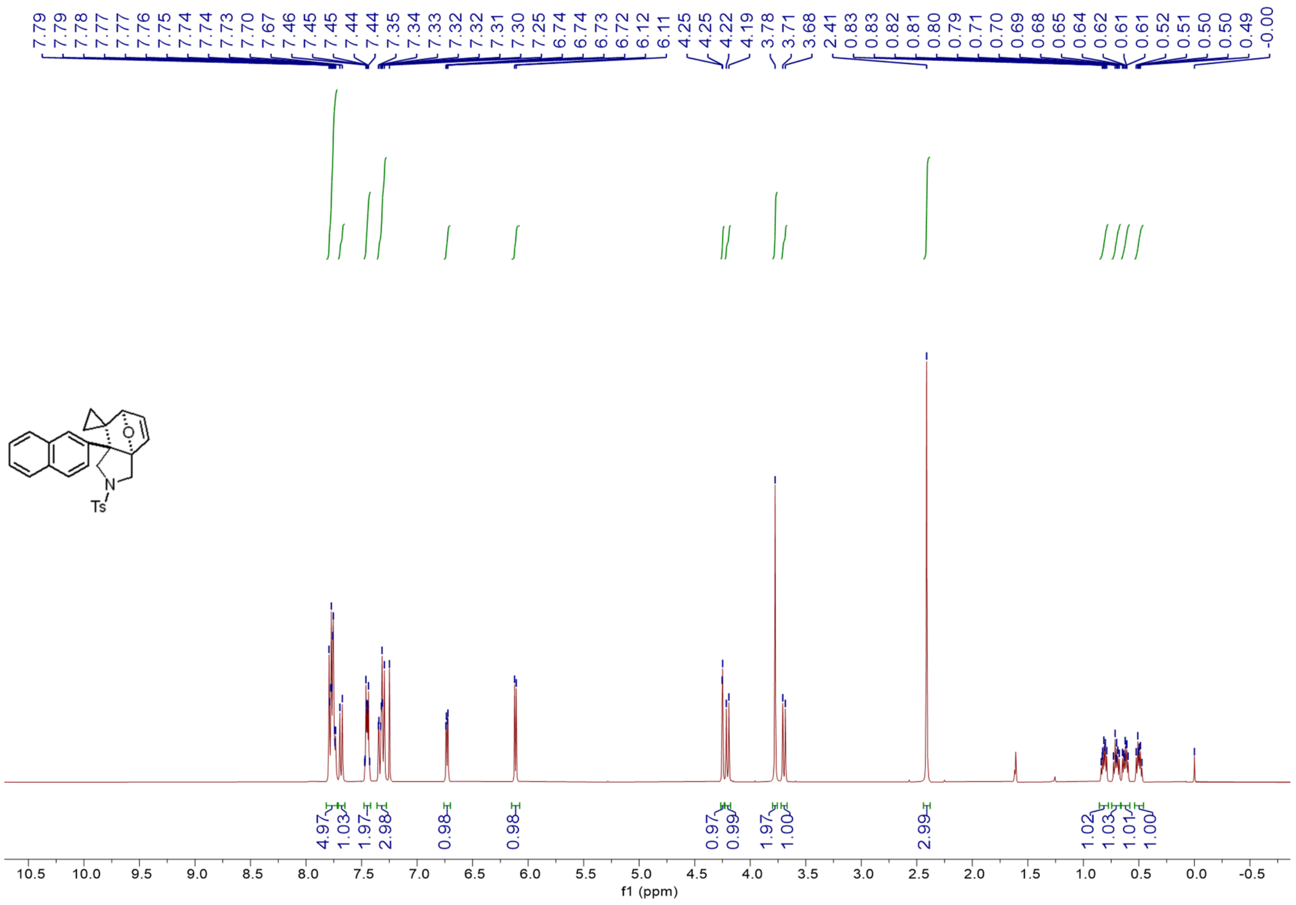
7.79 7.79 7.78 7.77 7.77 7.76 7.75 7.74 7.74 7.73 7.70 7.67 7.46 7.45 7.45 7.44 7.44 7.35 7.34 7.33 7.32 7.32 7.31 7.30 7.25 6.74 6.74 6.73 6.72 6.12 6.11 4.25 4.22 4.19 3.78 3.71 3.68 2.41 0.83 0.83 0.82 0.81 0.80 0.79 0.71 0.70 0.69 0.68 0.65 0.64 0.62 0.61 0.61 0.52 0.51 0.50 0.50 0.49 -0.00
O
N
Ts
4.97 1.03 1.97 2.98 0.98 0.98 0.97 0.99 1.97 1.00 2.99 1.02 1.03 1.01 1.00
10.5 10.0 9.5 9.0 8.5 8.0 7.5 7.0 6.5 6.0 5.5 5.0 4.5 4.0 3.5 3.0 2.5 2.0 1.5 1.0 0.5 0.0 -0.5
f1 (ppm)

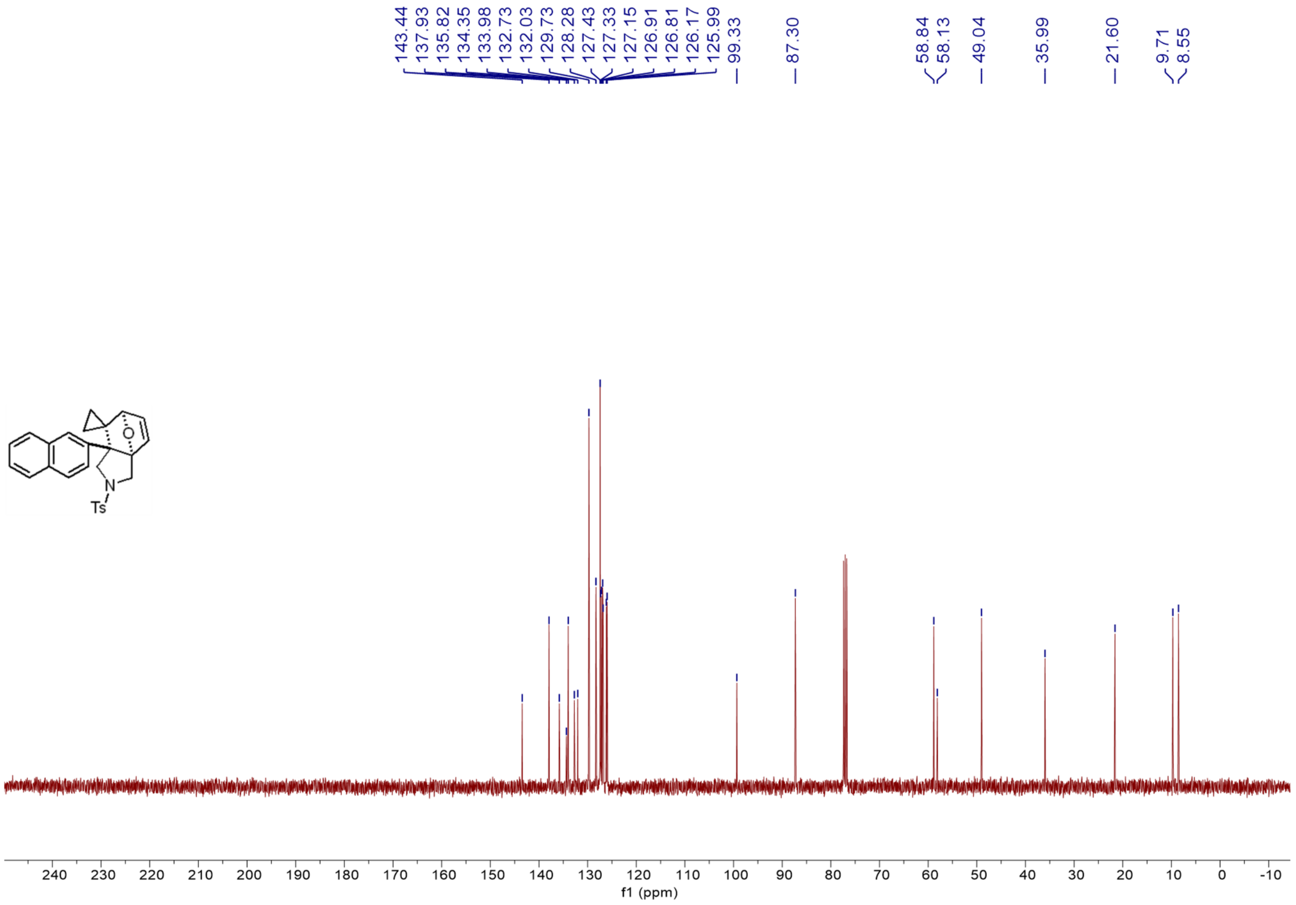
143.44 137.93 135.82 134.35 133.98 132.73 132.03 129.73 128.28 127.43 127.33 127.15 126.91 126.81 126.17 125.99 99.33 87.30 58.84 58.13 49.04 35.99 21.60 9.71 8.55
O
N
Ts
240 230 220 210 200 190 180 170 160 150 140 130 120 110 100 90 80 70 60 50 40 30 20 10 0 -10
f1 (ppm)

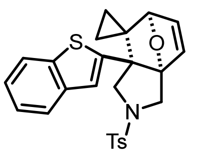

Compound 2s: Yield: 34.2 mg, 76%; A white solid; M.p.: > 200 °C; ^{1}H NMR (400 MHz, $CDCl_3$) δ 7.76 (d, *J* = 8.0 Hz, 2H), 7.70 (d, *J* = 7.6 Hz, 1H), 7.63 (d, *J* = 7.6 Hz, 1H), 7.33 - 7.23 (m, 4H), 7.07 (s, 1H), 6.72 (dd, *J* = 5.8, 1.8 Hz, 1H), 6.40 (d, *J* = 5.8 Hz, 1H), 4.25 (d, *J* = 1.8 Hz, 1H), 4.14 (d, *J* = 9.4 Hz, 1H), 3.90 (d, *J* = 12.0 Hz, 1H), 3.73 (d, *J* = 12.0 Hz, 1H), 3.62 (d, *J* = 9.4 Hz, 1H), 2.41 (s, 3H), 0.86 - 0.78 (m, 1H), 0.70 - 0.54 (m, 3H); ^{13}C NMR (100 MHz, $CDCl_3$) δ 144.0, 143.5, 139.7, 139.0, 138.6, 134.3, 129.7, 127.4, 124.2, 123.3, 123.2, 121.7, 99.1, 87.1, 59.0, 58.4, 48.8, 37.0, 21.6, 9.8, 8.4; IR (neat): ν 2994, 1711, 1458, 1344, 1162, 1009, 747, 670 cm^{-1}; HRMS (ESI) Calcd. for $C_{25}H_{23}NO_3S_2Na$ [M+Na]$^{+}$: 472.1012, Found: 472.1013.

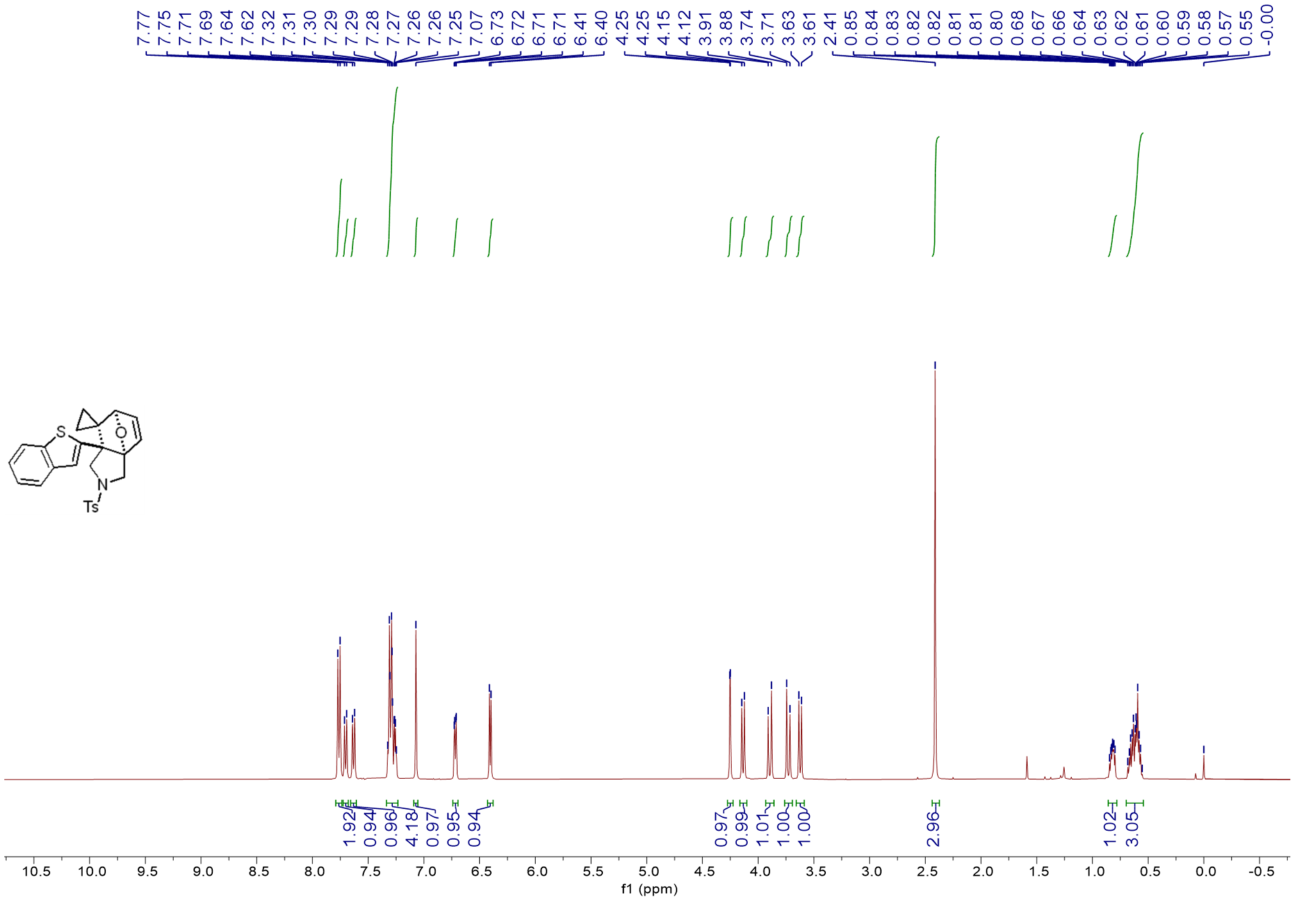
7.77 7.75 7.71 7.69 7.64 7.62 7.32 7.31 7.30 7.29 7.29 7.28 7.27 7.26 7.26 7.25 7.07 6.73 6.72 6.71 6.71 6.41 6.40 4.25 4.25 4.15 4.12 3.91 3.88 3.74 3.71 3.63 3.61 2.41 0.85 0.84 0.83 0.82 0.82 0.81 0.81 0.80 0.68 0.67 0.66 0.64 0.63 0.62 0.61 0.60 0.59 0.58 0.57 0.55 -0.00
S
O
N
Ts
1.92 0.94 0.96 4.18 0.97 0.95 0.94 0.97 0.99 1.01 1.00 1.00 2.96 1.02 3.05
10.5 10.0 9.5 9.0 8.5 8.0 7.5 7.0 6.5 6.0 5.5 5.0 4.5 4.0 3.5 3.0 2.5 2.0 1.5 1.0 0.5 0.0 -0.5
f1 (ppm)

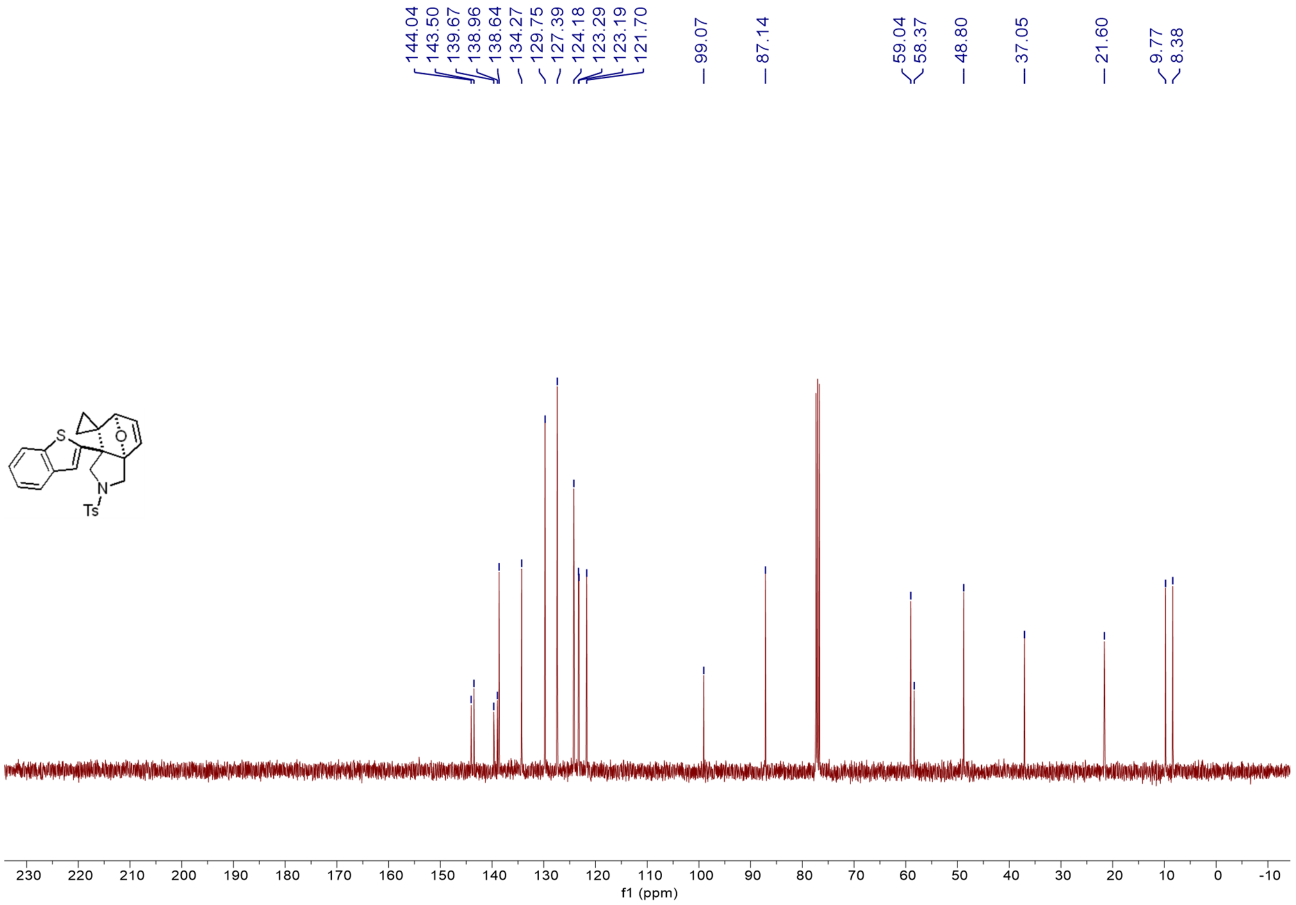
144.04 143.50 139.67 138.96 138.64 134.27 129.75 127.39 124.18 123.29 123.19 121.70 99.07 87.14 59.04 58.37 48.80 37.05 21.60 9.77 8.38
S
O
N
Ts
230 220 210 200 190 180 170 160 150 140 130 120 110 100 90 80 70 60 50 40 30 20 10 0 -10
f1 (ppm)

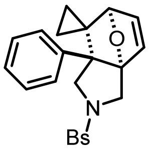

Compound 2t: Yield: 24.7 mg, 54%; A white solid; M.p.: > 200 °C; ^{1}H NMR (400 MHz, $CDCl_3$) δ 7.75 (d, J = 8.6 Hz, 2H), 7.68 (d, J = 8.6 Hz, 2H), 7.31 - 7.16 (m, 5H), 6.69 (dd, J = 5.8, 1.8 Hz, 1H), 6.08 (d, J = 5.8 Hz, 1H), 4.22 (d, J = 1.8 Hz, 1H), 4.08 (d, J = 9.2 Hz, 1H), 3.81 - 3.66 (m, 2H), 3.57 (d, J = 9.2 Hz, 1H), 0.85 - 0.75 (m, 1H), 0.72 - 0.63 (m, 1H), 0.62 - 0.53 (m, 1H), 0.52 - 0.43 (m, 1H); ^{13}C NMR (100 MHz, $CDCl_3$) δ 138.1, 137.9, 136.2, 133.7, 132.4, 128.9, 128.4, 127.8, 127.7, 126.9, 99.0, 87.2, 58.8, 57.9, 49.1, 36.1, 9.8, 8.6; IR (neat): ν 1574, 1471, 1389, 1349, 1162, 1068, 1009, 758, 739, 697 cm^{-1}; HRMS (ESI) Calcd. for $C_{22}H_{20}NO_3SNaBr$ $[M+Na]^+$: 480.0240, Found: 480.0246.

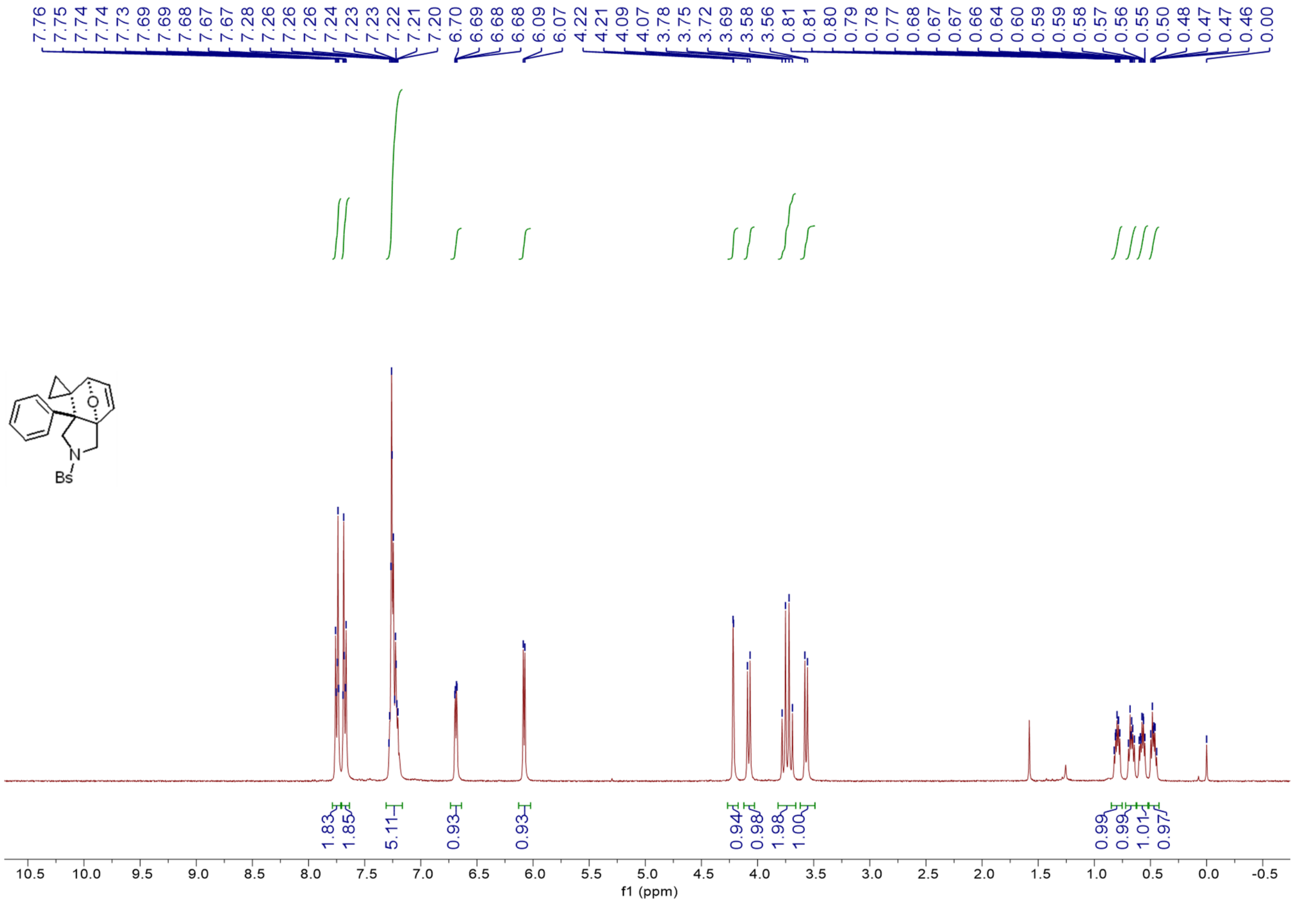
7.76 7.75 7.74 7.74 7.73 7.69 7.69 7.68 7.67 7.67 7.28 7.26 7.26 7.24 7.23 7.23 7.22 7.21 7.20 6.70 6.69 6.68 6.68 6.09 6.07 4.22 4.21 4.09 4.07 3.78 3.75 3.72 3.69 3.58 3.56 0.81 0.81 0.80 0.79 0.78 0.77 0.68 0.67 0.67 0.66 0.64 0.60 0.59 0.59 0.58 0.57 0.56 0.55 0.50 0.48 0.47 0.47 0.46 0.00
O
N
Bs
1.83 1.85 5.11 0.93 0.93 0.94 0.98 1.98 1.00 0.99 0.99 1.01 0.97
10.5 10.0 9.5 9.0 8.5 8.0 7.5 7.0 6.5 6.0 5.5 5.0 4.5 4.0 3.5 3.0 2.5 2.0 1.5 1.0 0.5 0.0 -0.5
f1 (ppm)

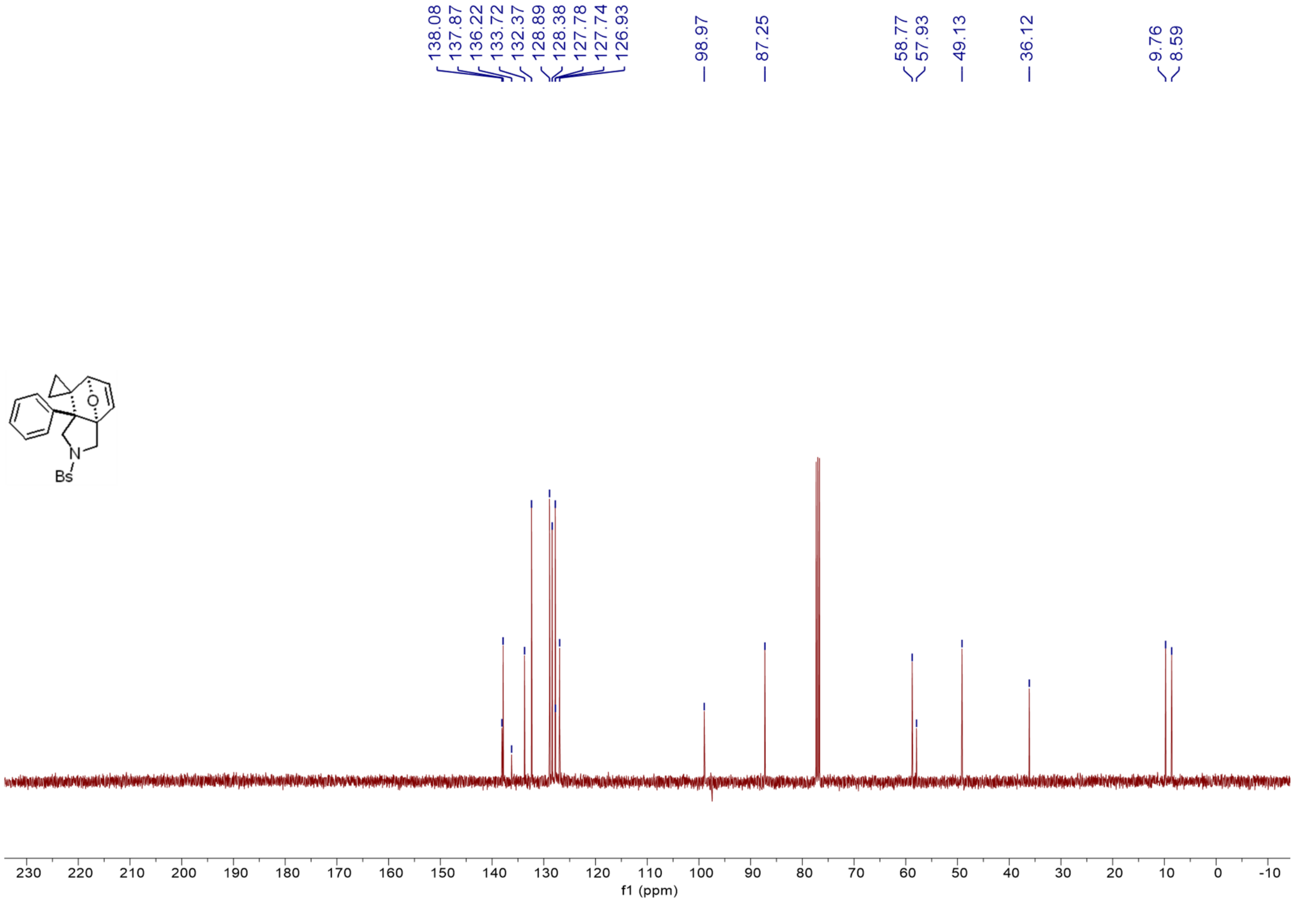
138.08 137.87 136.22 133.72 132.37 128.89 128.38 127.78 127.74 126.93 98.97 87.25 58.77 57.93 49.13 36.12 9.76 8.59
O
N
Bs
230 220 210 200 190 180 170 160 150 140 130 120 110 100 90 80 70 60 50 40 30 20 10 0 -10
f1 (ppm)

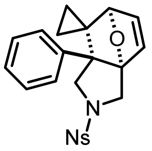

Compound 2u: Yield: 36.0 mg, 85%; A white solid; M.p.: > 200 ºC; ^{1}H NMR (400 MHz, $CDCl_3$) δ 8.39 (d, J = 9.0 Hz, 2H), 8.06 (d, J = 9.0 Hz, 1H), 7.30 - 7.18 (m, 5H), 6.69 (dd, J = 5.8, 1.8 Hz, 1H), 6.09 (d, J = 5.8 Hz, 1H), 4.19 (d, J = 1.8 Hz, 1H), 4.14 (d, J = 9.2 Hz, 1H), 3.81 (d, J = 12.2 Hz, 1H), 3.73 (d, J = 12.2 Hz, 1H), 3.58 (d, J = 9.2 Hz, 1H), 0.83 - 0.76 (m, 1H), 0.71 - 0.63 (m, 1H), 0.62 - 0.54 (m, 1H), 0.52 - 0.45 (m, 1H). ^{13}C NMR (100 MHz, $CDCl_3$) δ 150.1, 142.8, 138.1, 137.9, 133.4, 128.6, 128.2, 127.9, 127.1, 124.4, 98.8, 87.2, 59.1, 58.0, 49.3, 36.2, 9.7, 8.6; IR (neat): ν 2991, 1711, 1529, 1349, 1312, 1167, 1109, 855, 735, 688 cm^{-1}; HRMS (ESI) Calcd. for $C_{22}H_{20}N_2O_5SNa$ $[M+Na]^+$: 447.0985, Found: 447.0978.

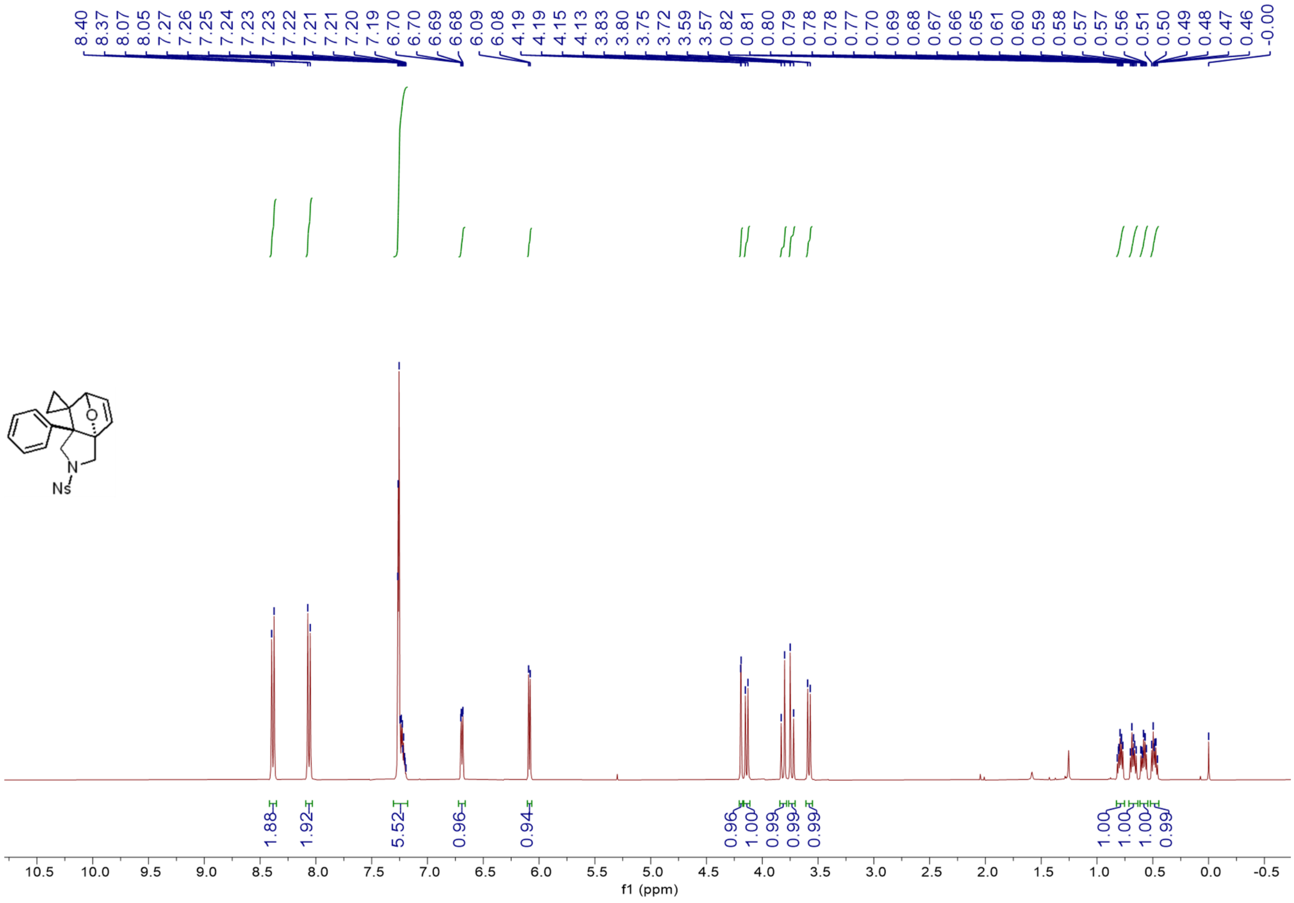
8.40
8.37
8.07
8.05
7.27
7.26
7.25
7.24
7.23
7.23
7.22
7.21
7.21
7.20
7.19
6.70
6.70
6.69
6.68
6.09
6.08
4.19
4.19
4.15
4.13
3.83
3.80
3.75
3.72
3.59
3.57
0.82
0.81
0.80
0.79
0.78
0.78
0.77
0.70
0.69
0.68
0.67
0.66
0.65
0.61
0.60
0.59
0.58
0.57
0.57
0.56
0.51
0.50
0.49
0.48
0.47
0.46
-0.00
Ns
N
O
1.88
1.92
5.52
0.96
0.94
0.96
1.00
0.99
0.99
0.99
1.00
1.00
1.00
0.99
f1 (ppm)

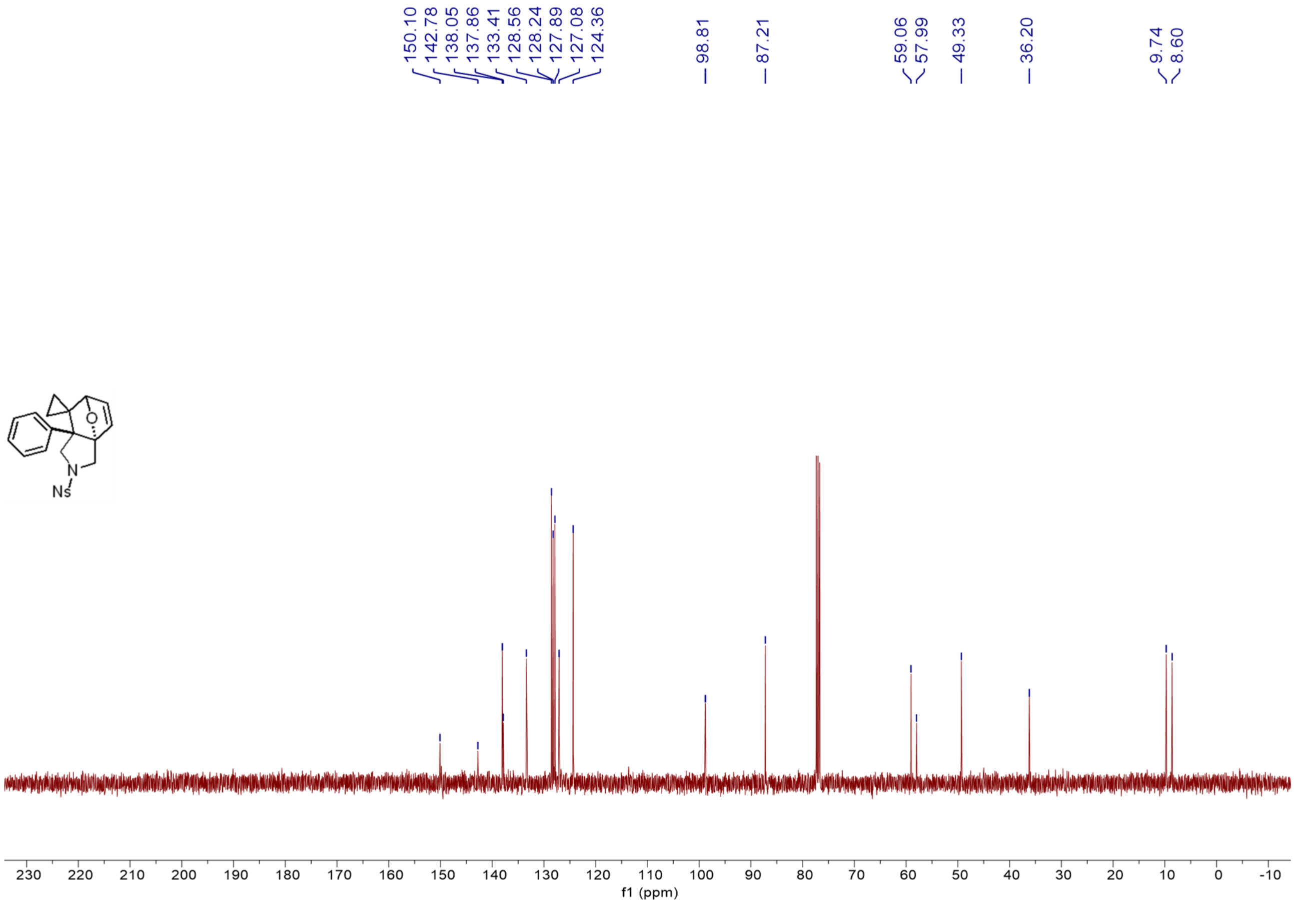
150.10
142.78
138.05
137.86
133.41
128.56
128.24
127.89
127.08
124.36
98.81
87.21
59.06
57.99
49.33
36.20
9.74
8.60
Ns
N
O
f1 (ppm)

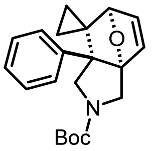

Compound 2v: Yield: 30.5 mg, 90%; A yellow oil; ^{1}H NMR (400 MHz, $CDCl_3$) (Mixture of two rotamers 54:46) δ 7.28 - 7.13 (m, 5H), 6.76 - 6.71 (m, 1H), 6.08 (t, *J* = 6.4 Hz, 1H), 4.37 (d, *J* = 1.8 Hz, 1H), 4.16 (d, *J* = 11.0 Hz, 0.54H), 4.03 (d, *J* = 11.0 Hz, 0.46H), 3.88 (d, *J* = 12.8 Hz, 0.46H), 3.84 - 3.76 (m, 1.5H), 3.62 (t, *J* = 11.8 Hz, 1H), 1.54 (s, 4H), 1.50 (s, 5H), 0.92 - 0.83 (m, 1H), 0.78 - 0.63 (m, 2H), 0.55 - 0.47 (m, 1H). ^{13}C NMR (100 MHz, $CDCl_3$) (Mixture of two rotamers) δ 150.1, 142.8, 138.1, 137.9, 133.4, 128.6, 128.2, 127.9, 127.1, 124.4, 98.8, 87.2, 59.1, 58.0, 49.3, 36.2, 9.7, 8.6; IR (neat): ν 2977, 1694, 1403, 1366, 1168, 1117, 841, 741, 703 cm^{-1}; HRMS (ESI) Calcd. for $C_{21}H_{25}NO_3Na$ $[M+Na]^+$: 362.1727, Found: 362.1720.

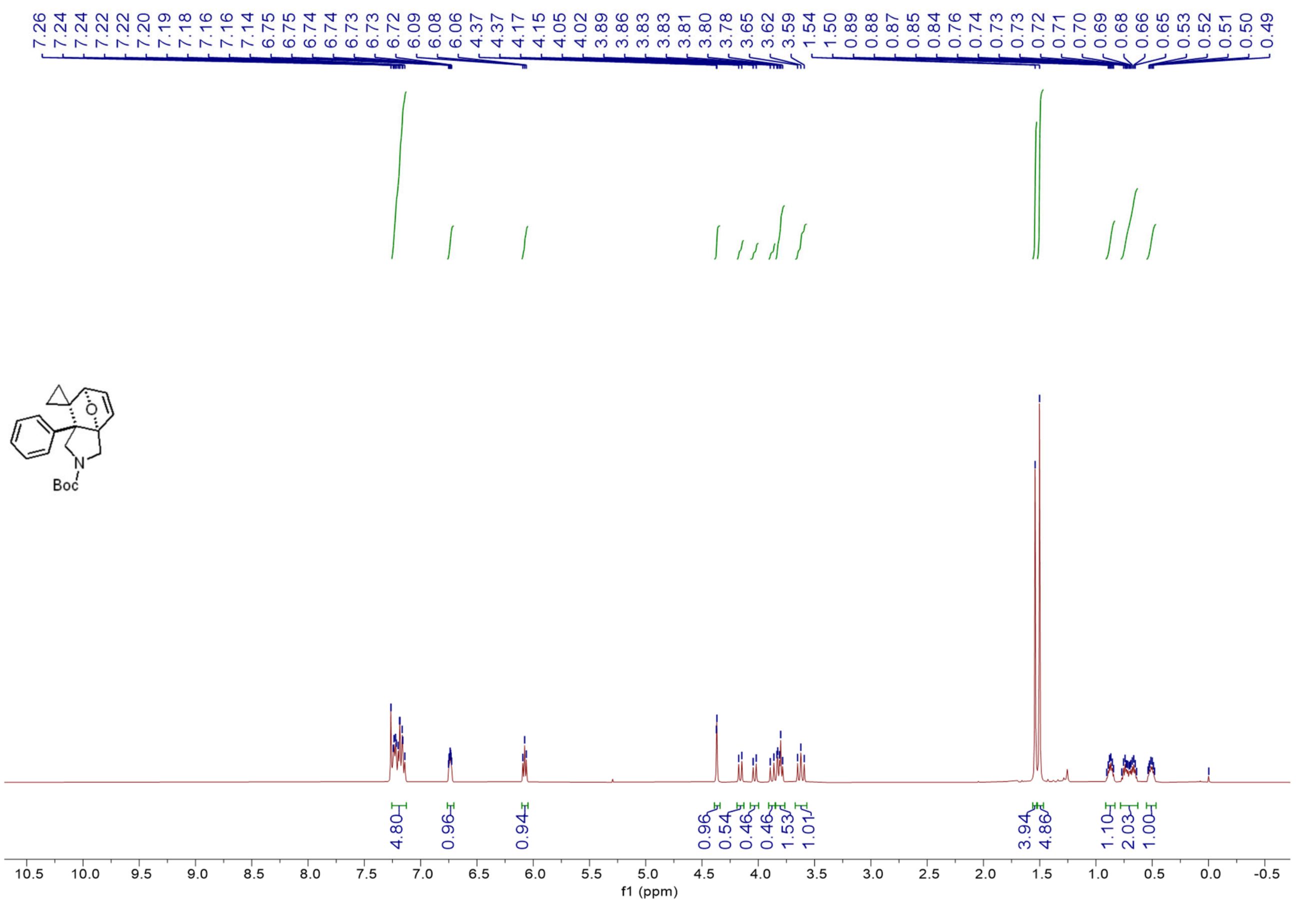
7.26 7.24 7.24 7.22 7.20 7.19 7.18 7.16 7.14 6.75 6.74 6.74 6.73 6.73 6.72 6.09 6.08 6.06 4.37 4.37 4.17 4.15 4.05 4.02 3.89 3.86 3.83 3.83 3.81 3.80 3.78 3.65 3.62 3.59 1.54 1.50 0.89 0.88 0.87 0.85 0.84 0.76 0.74 0.73 0.73 0.72 0.71 0.70 0.69 0.68 0.66 0.65 0.53 0.52 0.51 0.50 0.49
O
N
Boc
4.80
0.96
0.94
0.96
0.54
0.46
0.46
1.53
1.01
3.94
4.86
1.10
2.03
1.00
10.5 10.0 9.5 9.0 8.5 8.0 7.5 7.0 6.5 6.0 5.5 5.0 4.5 4.0 3.5 3.0 2.5 2.0 1.5 1.0 0.5 0.0 -0.5
f1 (ppm)
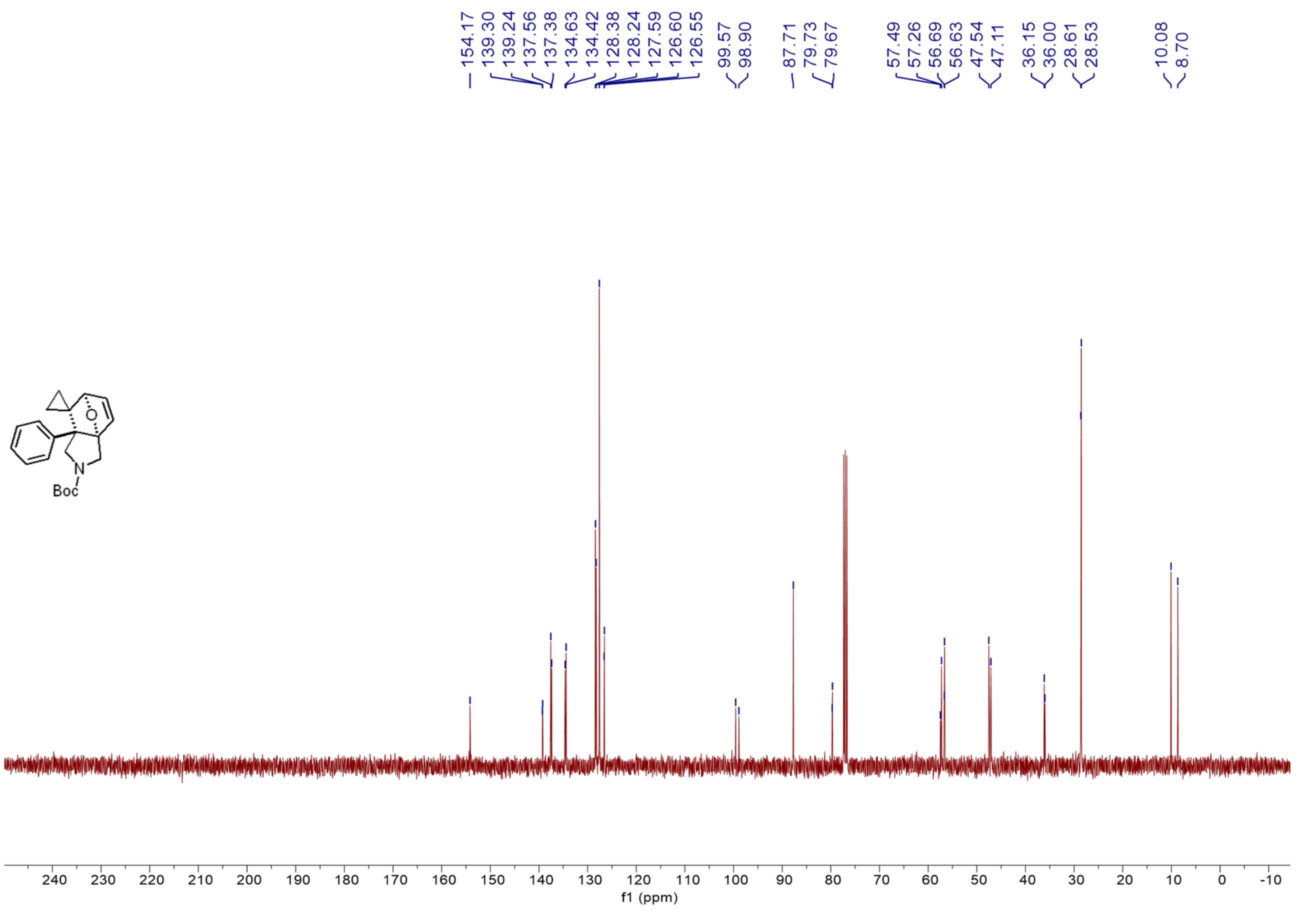
154.17 139.30 139.24 137.56 137.38 134.63 134.42 128.38 128.24 127.59 126.60 126.55 99.57 98.90 87.71 79.73 79.67 57.49 57.26 56.69 56.63 47.54 47.11 36.15 36.00 28.61 28.53 10.08 8.70
O
N
Boc
240 230 220 210 200 190 180 170 160 150 140 130 120 110 100 90 80 70 60 50 40 30 20 10 0 -10
f1 (ppm)

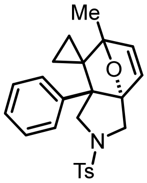

Compound 2x: Yield: 24.0 mg, 59%; A white solid; M.p.: > 200 ºC; ^{1}H NMR (400 MHz, $CDCl_3$) δ 7.75 (d, *J* = 8.2 Hz, 2H), 7.35 – 7.14 (m, 7H), 6.42 (d, *J* = 5.6 Hz, 1H), 6.07 (d, *J* = 5.6 Hz, 1H), 4.03 (d, *J* = 9.2 Hz, 1H), 3.75 - 3.63 (m, 2H), 3.56 (d, *J* = 9.2 Hz, 1H), 2.43 (s, 3H), 1.12 (s, 3H), 0.84 - 0.74 (m, 1H), 0.50 - 0.40 (m, 2H), 0.40 - 0.32 (m, 1H). ^{13}C NMR (100 MHz, $CDCl_3$) δ 143.3, 141.0, 138.3, 134.4, 134.1, 129.6, 128.5, 127.6, 127.4, 126.8, 96.7, 88.9, 59.9, 58.8, 49.1, 38.2, 21.5, 14.2, 8.4, 5.3; IR (neat): ν 2975, 1340, 1164, 1110, 1048, 997, 807, 747, 704, 667 cm^{-1}; HRMS (ESI) Calcd. for $C_{24}H_{25}NO_3SNa$ $[M+Na]^+$: 430.1447, Found: 430.1446.

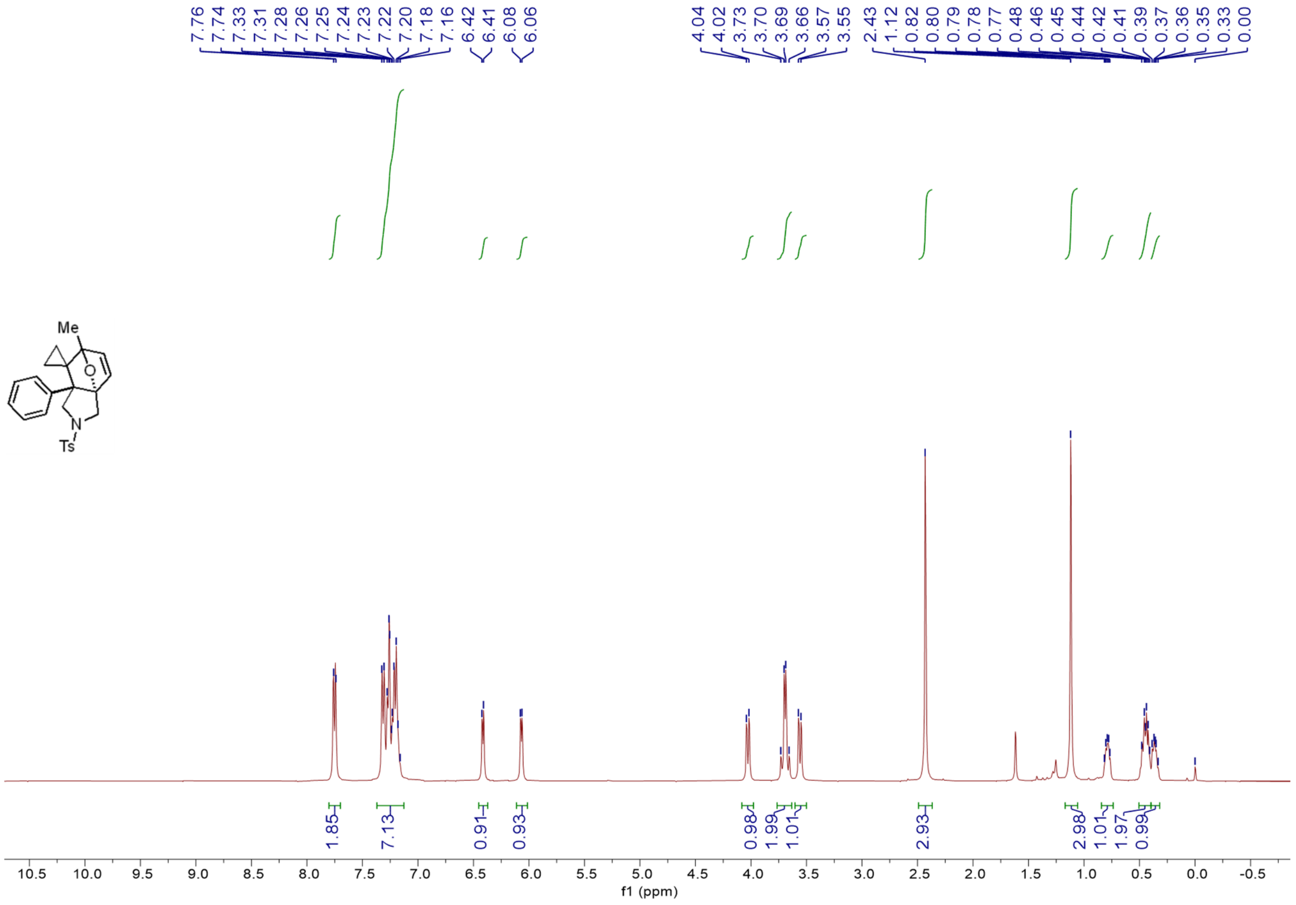

7.76
7.74
7.33
7.31
7.28
7.26
7.25
7.24
7.23
7.22
7.20
7.18
7.16
6.42
6.41
6.08
6.06
4.04
4.02
3.73
3.70
3.69
3.66
3.57
3.55
2.43
1.12
0.82
0.80
0.79
0.78
0.77
0.48
0.46
0.45
0.44
0.42
0.41
0.39
0.37
0.36
0.35
0.33
0.00
Me
O
N
Ts
1.85
7.13
0.91
0.93
0.98
1.99
1.01
2.93
2.98
1.01
1.97
0.99
f1 (ppm)

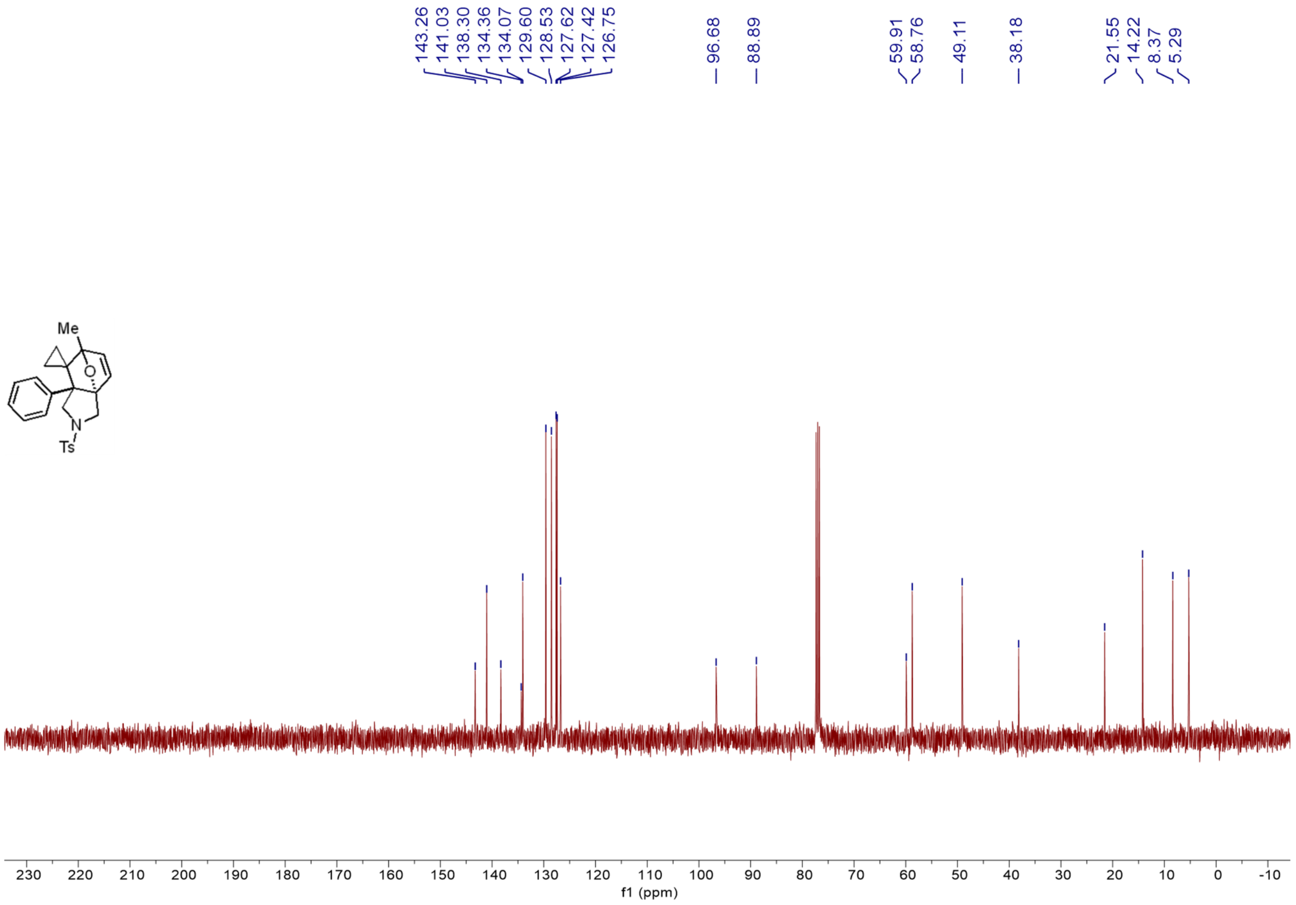

143.26
141.03
138.30
134.36
134.07
129.60
128.53
127.62
127.42
126.75
96.68
88.89
59.91
58.76
49.11
38.18
21.55
14.22
8.37
5.29
Me
O
N
Ts
f1 (ppm)

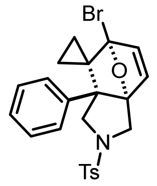

Compound 2y: Yield: 42.5 mg, 90%; A white solid; M.p.: > 200 ºC; ^{1}H NMR (400 MHz, CDCl$_3$) δ 7.75 (d, *J* = 8.2 Hz, 2H), 7.34 (d, *J* = 8.2 Hz, 2H), 7.30 - 7.21 (m, 5H), 6.62 (d, *J* = 5.6 Hz, 1H), 6.08 (d, *J* = 5.6 Hz, 1H), 4.09 (d, *J* = 9.4 Hz, 1H), 3.75 (s, 2H), 3.60 (d, *J* = 9.4 Hz, 1H), 2.44 (s, 3H), 1.29 - 1.19 (m, 1H), 0.76 - 0.67 (m, 1H), 0.57 - 1.45 (m, 2H). ^{13}C NMR (100 MHz, CDCl$_3$) δ 143.7, 141.7, 136.9, 134.4, 133.9, 129.8, 128.4, 128.0, 127.4, 127.3, 97.3, 96.7, 59.0, 58.7, 48.9, 40.1, 21.6, 8.5, 7.9; IR (neat): ν 2962, 1497, 1477, 1341, 1162, 1100, 1074, 1008, 758, 684 cm^{-1}; HRMS (ESI) Calcd. for $C_{23}H_{22}NO_3SNaBr$ $[M+Na]^+$: 494.0396, Found: 494.0394.

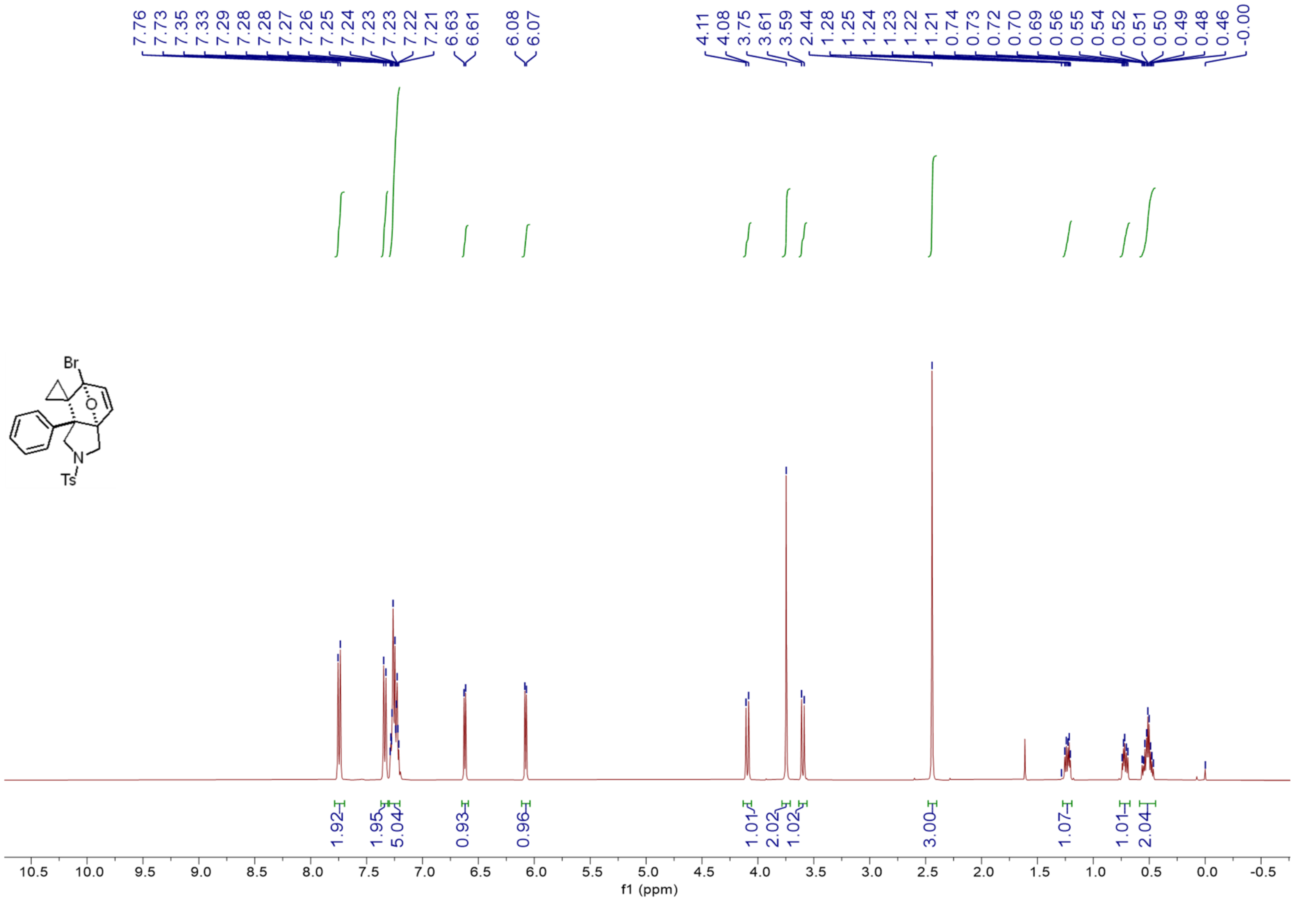
7.76
7.73
7.35
7.33
7.29
7.28
7.28
7.27
7.26
7.25
7.24
7.23
7.23
7.22
7.21
6.63
6.61
6.08
6.07
4.11
4.08
3.75
3.61
3.59
2.44
1.28
1.25
1.24
1.23
1.22
1.21
0.74
0.73
0.72
0.70
0.69
0.56
0.55
0.54
0.52
0.51
0.50
0.49
0.48
0.46
-0.00
Br
O
N
Ts
1.92
1.95
5.04
0.93
0.96
1.01
2.02
1.02
3.00
1.07
1.01
2.04
10.5 10.0 9.5 9.0 8.5 8.0 7.5 7.0 6.5 6.0 5.5 5.0 4.5 4.0 3.5 3.0 2.5 2.0 1.5 1.0 0.5 0.0 -0.5
f1 (ppm)

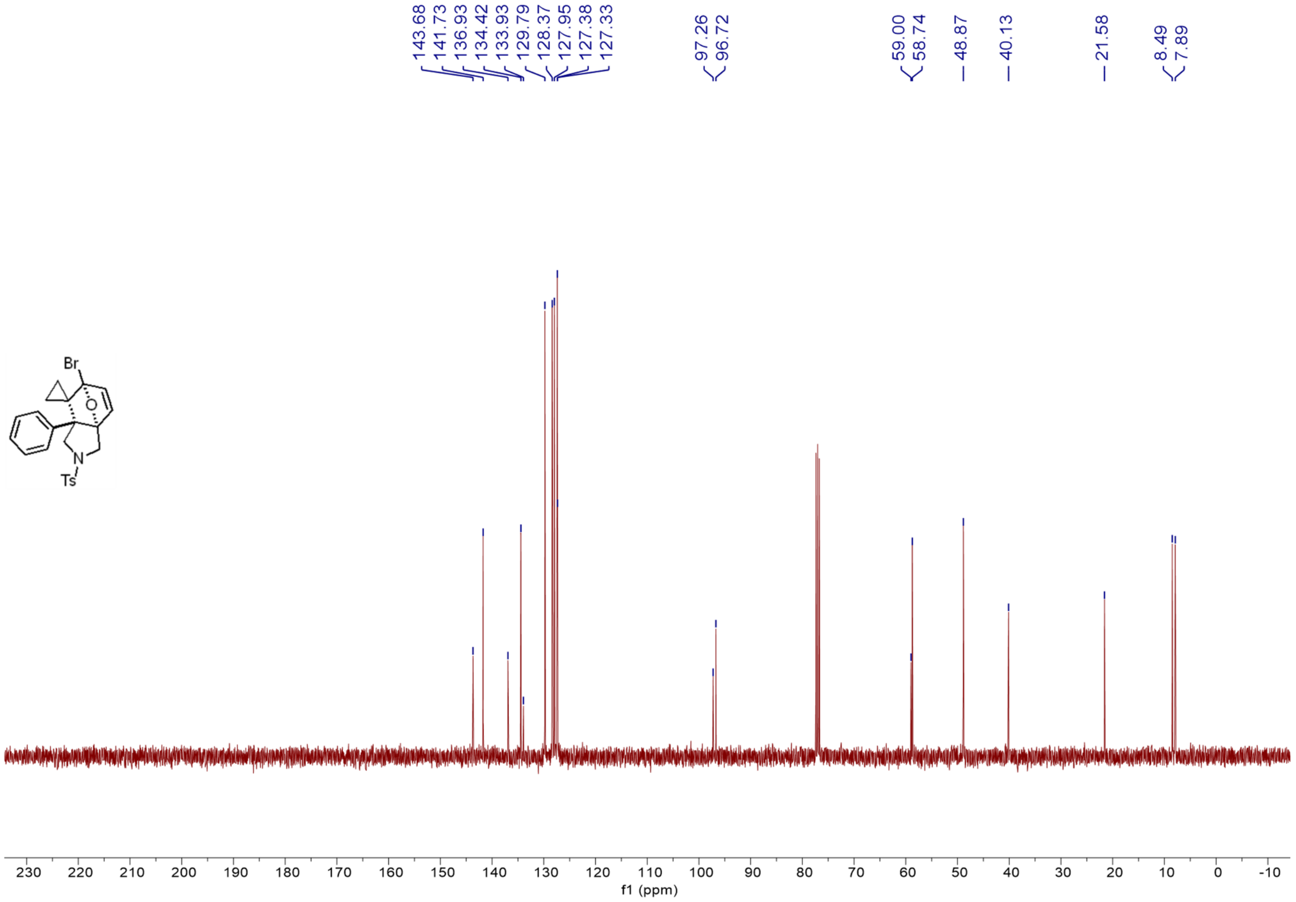
143.68
141.73
136.93
134.42
133.93
129.79
128.37
127.95
127.38
127.33
97.26
96.72
59.00
58.74
48.87
40.13
21.58
8.49
7.89
Br
O
N
Ts
230 220 210 200 190 180 170 160 150 140 130 120 110 100 90 80 70 60 50 40 30 20 10 0 -10
f1 (ppm)

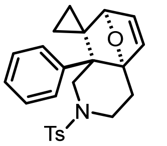

Compound 2z: Yield: 23.6 mg, 58%; A white solid; M.p.: > 200 °C; ^{1}H NMR (400 MHz, $CDCl_3$) δ 7.66 (d, *J* = 8.0 Hz, 2H), 7.62 - 7.55 (m, 2H), 7.35 (d, *J* = 8.0 Hz, 2H), 7.29 - 7.23 (m, 2H), 7.21 - 7.14 (m, 1H), 6.69 (dd, *J* = 5.8, 1.8 Hz, 1H), 6.14 (d, *J* = 5.8 Hz, 1H), 4.18 (d, *J* = 1.8 Hz, 1H), 4.03 (dd, *J* = 12.0, 1.8 Hz, 1H), 3.79 (dd, *J* = 9.8, 4.8 Hz, 1H), 2.94 (d, *J* = 12.0 Hz, 1H), 2.71 - 2.61 (m, 1H), 2.46 (s, 3H), 2.23 (td, *J* = 13.6, 5.0 Hz, 1H), 2.09 (dt, *J* = 14.4, 2.7 Hz, 1H), 0.65 - 0.54 (m, 2H), 0.53 - 0.45 (m, 1H), 0.29 - 0.20 (m, 1H). ^{13}C NMR (100 MHz, $CDCl_3$) δ 143.6, 139.8, 137.7, 137.6, 132.6, 130.1, 129.8, 127.8, 127.4, 126.3, 89.7, 86.2, 54.6, 47.7, 42.1, 37.6, 26.7, 8.4, 7.9; IR (neat): ν 2991, 2964, 1338, 1261, 1159, 1090, 1027, 800, 734 cm^{-1}; HRMS (ESI) Calcd. for $C_{24}H_{26}NO_3S$ $[M+H]^+$: 408.1628, Found: 408.1623.

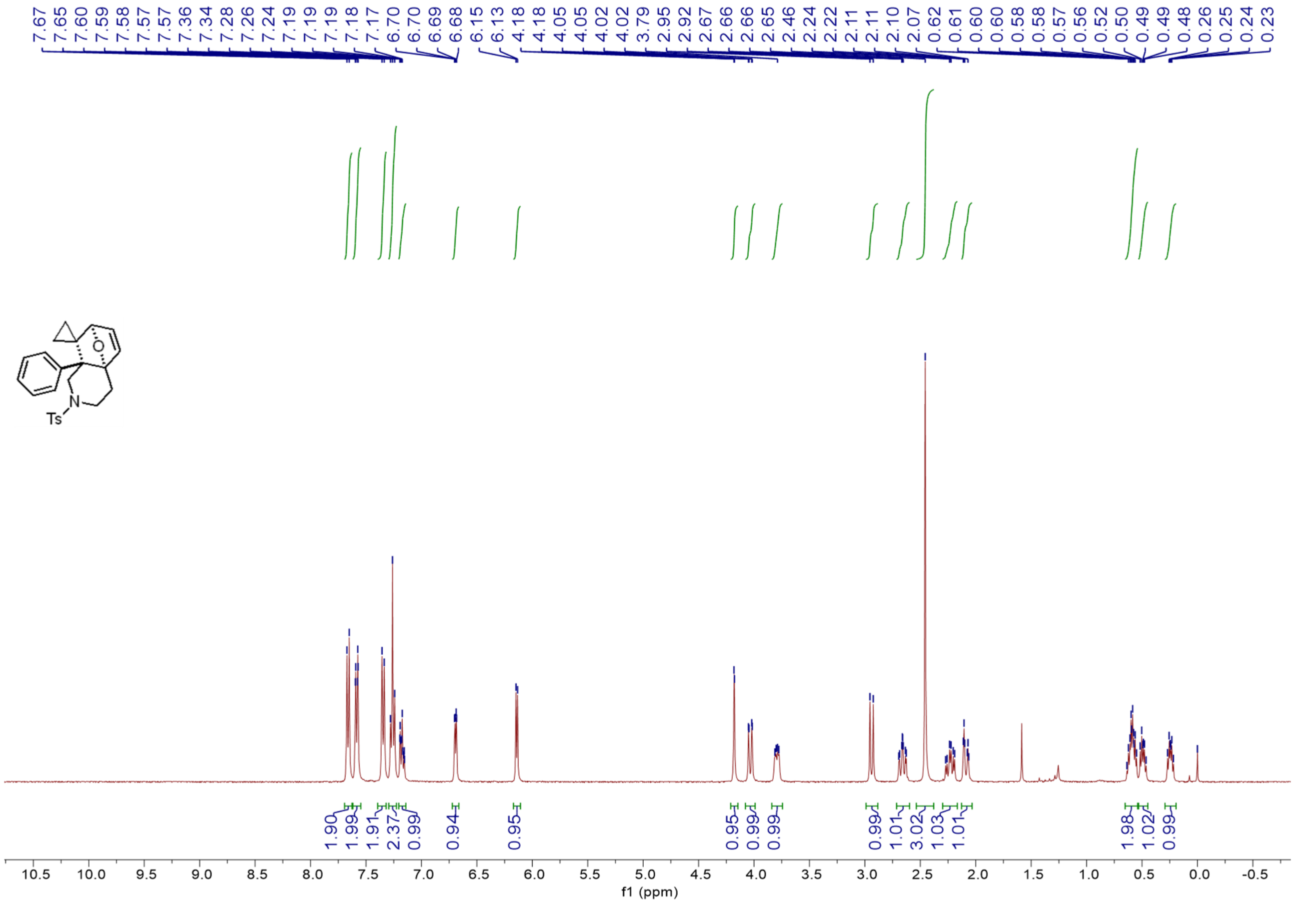
7.67 7.65 7.60 7.59 7.58 7.57 7.57 7.36 7.34 7.28 7.26 7.24 7.19 7.19 7.18 7.17 6.70 6.70 6.69 6.68 6.15 6.13 4.18 4.18 4.05 4.05 4.02 4.02 3.79 2.95 2.92 2.67 2.66 2.66 2.65 2.46 2.24 2.22 2.11 2.11 2.10 2.07 0.62 0.61 0.60 0.58 0.58 0.57 0.56 0.52 0.50 0.49 0.49 0.48 0.26 0.25 0.24 0.23
O
N
Ts
1.90 1.99 1.91 2.37 0.99 0.94 0.95 0.95 0.99 0.99 0.99 1.01 3.02 1.03 1.01 1.98 1.02 0.99
10.5 10.0 9.5 9.0 8.5 8.0 7.5 7.0 6.5 6.0 5.5 5.0 4.5 4.0 3.5 3.0 2.5 2.0 1.5 1.0 0.5 0.0 -0.5
f1 (ppm)

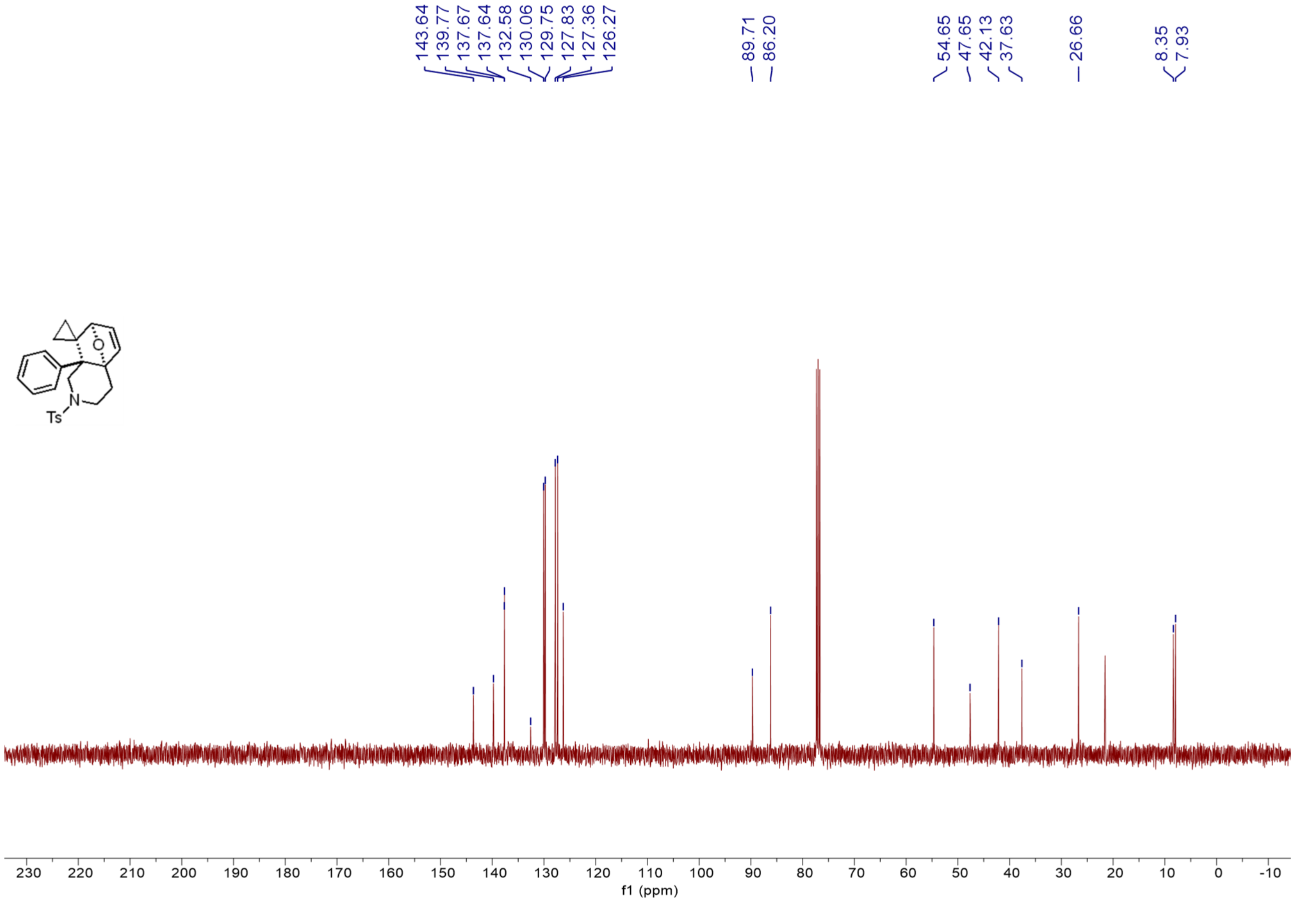
143.64 139.77 137.67 137.64 132.58 130.06 129.75 127.83 127.36 126.27
89.71 86.20
54.65 47.65 42.13 37.63
26.66
8.35 7.93
O
N
Ts
230 220 210 200 190 180 170 160 150 140 130 120 110 100 90 80 70 60 50 40 30 20 10 0 -10
f1 (ppm)

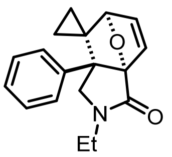

Compound 2aa: Yield: 27.6 mg, 98%; A white solid; M.p.: 165 - 167 ºC; ^{1}H NMR (400 MHz, $CDCl_3$) δ 7.29 - 7.16 (m, 3H), 7.10 - 7.04 (m, 2H), 6.75 (dd, *J* = 5.8, 1.8 Hz, 1H), 6.36 (d, *J* = 5.8 Hz, 1H), 4.46 (d, *J* = 1.8 Hz, 1H), 4.08 (d, *J* = 9.8 Hz, 1H), 3.69 - 3.56 (m, 2H), 3.41 (dq, *J* = 14.2, 7.2 Hz, 1H), 1.24 (d, *J* = 7.2 Hz, 3H), 1.00 - 0.91 (m, 1H), 0.78 - 0.70 (m, 2H), 0.64 - 0.54 (m, 1H). ^{13}C NMR (100 MHz, $CDCl_3$) δ 166.2, 137.5, 136.9, 132.2, 127.4, 126.7, 125.8, 96.4, 88.2, 57.1, 54.3, 36.8, 34.5, 11.5, 8.8, 7.2; IR (neat): ν 2979, 1697, 1489, 1260, 1047, 939, 999, 887, 726, 713 cm^{-1}; HRMS (ESI) Calcd. for $C_{18}H_{20}NO_2$ $[M+H]^+$: 282.1489, Found: 282.1488.

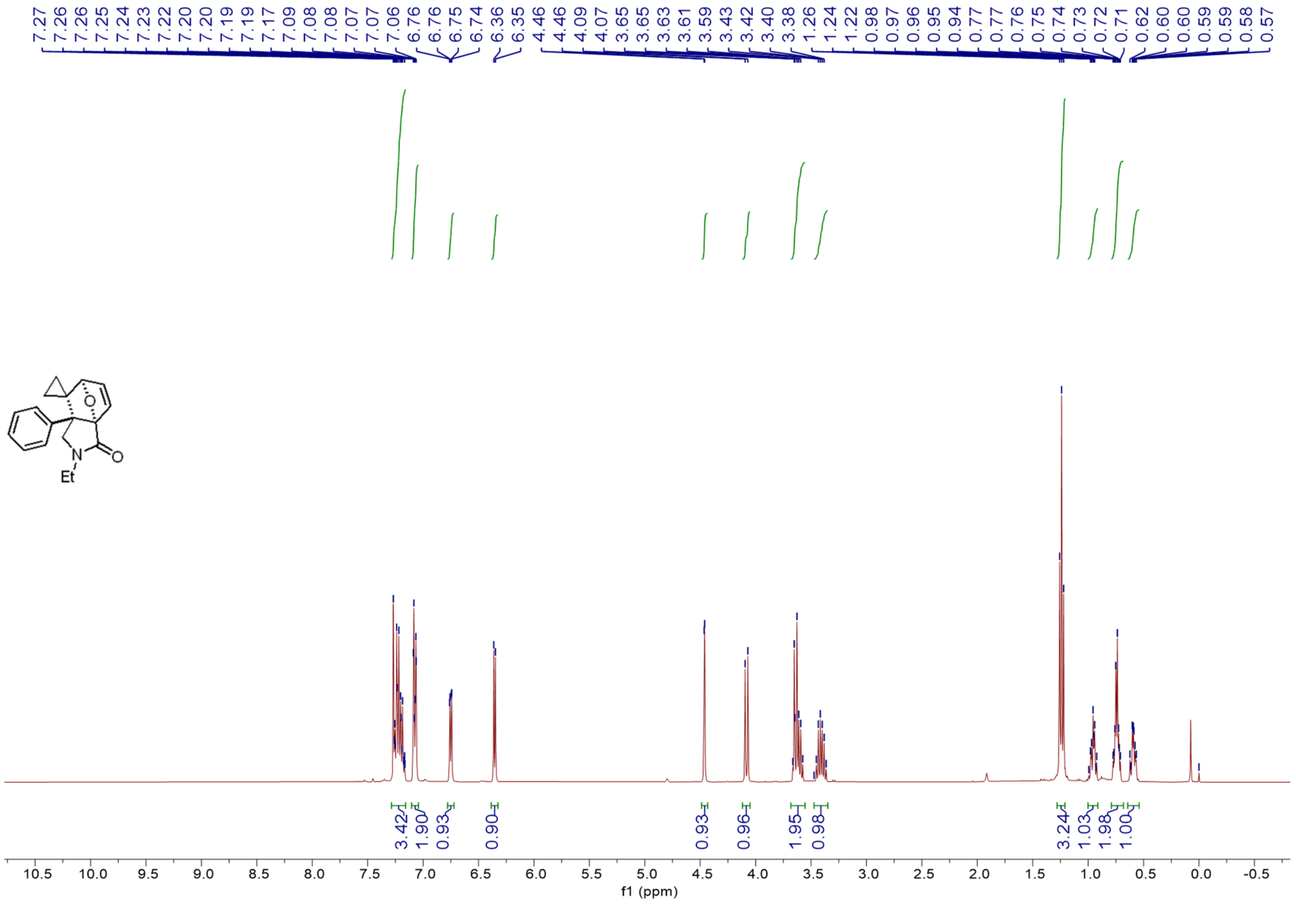
7.27 7.26 7.26 7.25 7.24 7.23 7.22 7.20 7.20 7.19 7.19 7.17 7.09 7.08 7.08 7.07 7.07 7.06 6.76 6.76 6.75 6.74 6.36 6.35 4.46 4.09 4.07 3.65 3.65 3.63 3.61 3.59 3.43 3.42 3.40 3.38 1.26 1.24 1.22 0.98 0.97 0.96 0.95 0.94 0.77 0.76 0.75 0.74 0.73 0.72 0.71 0.62 0.60 0.60 0.59 0.59 0.58 0.57
O
N
O
Et
3.42
1.90
0.93
0.90
0.93
0.96
1.95
0.98
3.24
1.03
1.98
1.00
10.5 10.0 9.5 9.0 8.5 8.0 7.5 7.0 6.5 6.0 5.5 5.0 4.5 4.0 3.5 3.0 2.5 2.0 1.5 1.0 0.5 0.0 -0.5
f1 (ppm)

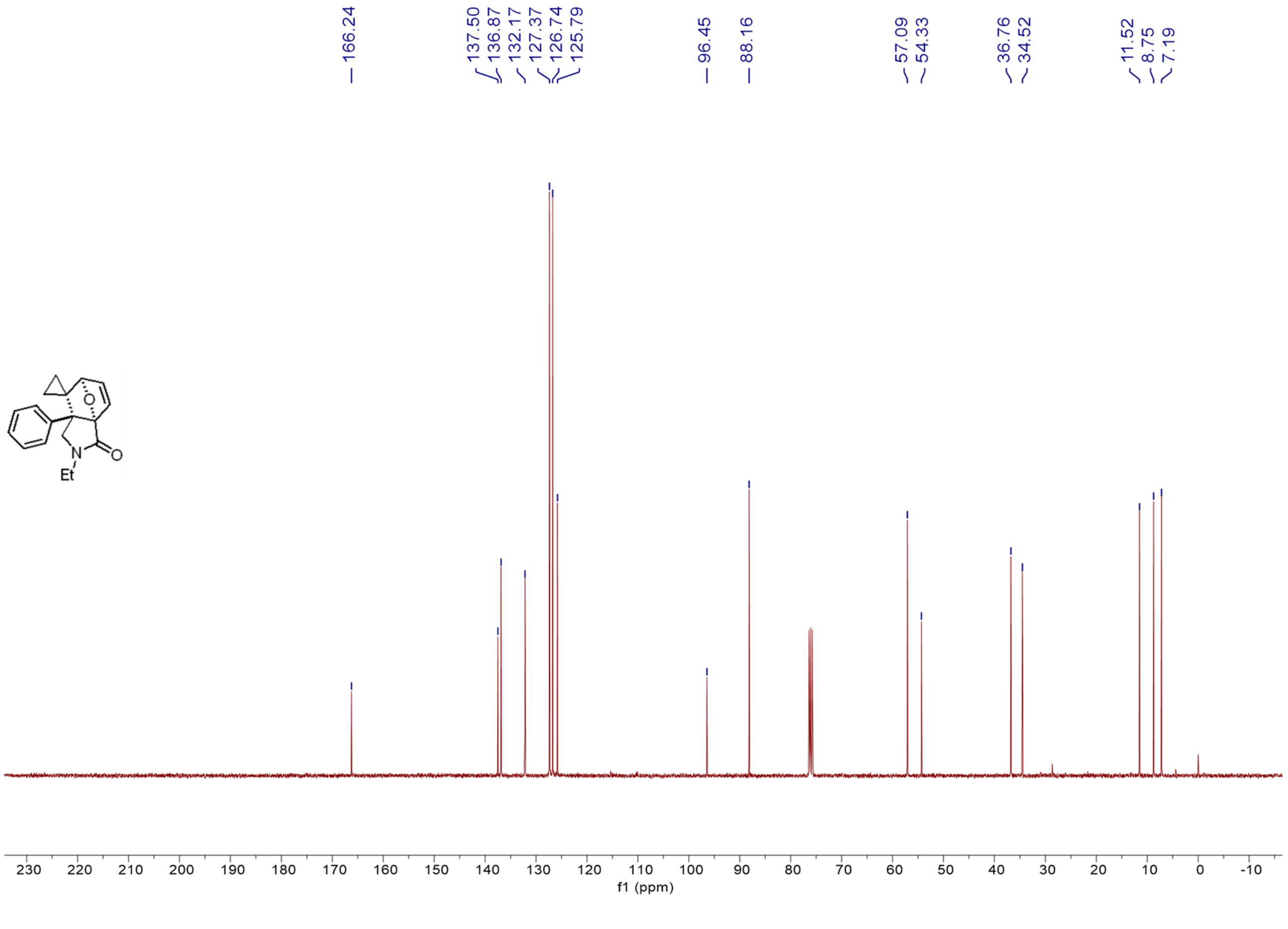
166.24
137.50 136.87 132.17 127.37 126.74 125.79
96.45
88.16
57.09 54.33
36.76 34.52
11.52 8.75 7.19
O
N
O
Et
230 220 210 200 190 180 170 160 150 140 130 120 110 100 90 80 70 60 50 40 30 20 10 0 -10
f1 (ppm)

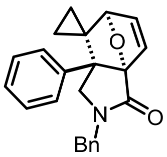

Compound 2ab: Yield: 33.3 mg, 97%; A yellow oil; ^{1}H NMR (400 MHz, $CDCl_3$) δ 7.38 - 7.27 (m, 5H), 7.18 - 7.07 (m, 3H), 6.89 - 6.82 (m, 2H), 6.76 (dd, *J* = 5.8, 1.8 Hz, 1H), 6.42 (d, *J* = 5.8 Hz, 1H), 4.70 (d, *J* = 14.6 Hz, 1H), 4.52 (d, *J* = 14.6 Hz, 1H), 4.47 (d, *J* = 1.8 Hz, 1H), 3.96 (d, *J* = 9.8 Hz, 1H), 3.52 (d, *J* = 9.8 Hz, 1H), 0.95 - 0.86 (m, 1H), 0.72 - 0.62 (m, 2H), 0.56 - 0.46 (m, 1H). ^{13}C NMR (100 MHz, $CDCl_3$) δ 166.6, 137.3, 137.0, 134.7, 132.1, 127.7, 127.5, 127.4, 126.8, 126.6, 125.7, 96.2, 88.2, 57.0, 54.5, 46.0, 34.4, 8.7, 7.1; IR (neat): ν 2917, 1702, 1495, 1442, 1260, 1046, 939, 767, 748, 701 cm^{-1}; HRMS (ESI) Calcd. for $C_{23}H_{22}NO_2$ $[M+H]^+$: 344.1645, Found: 344.1645.

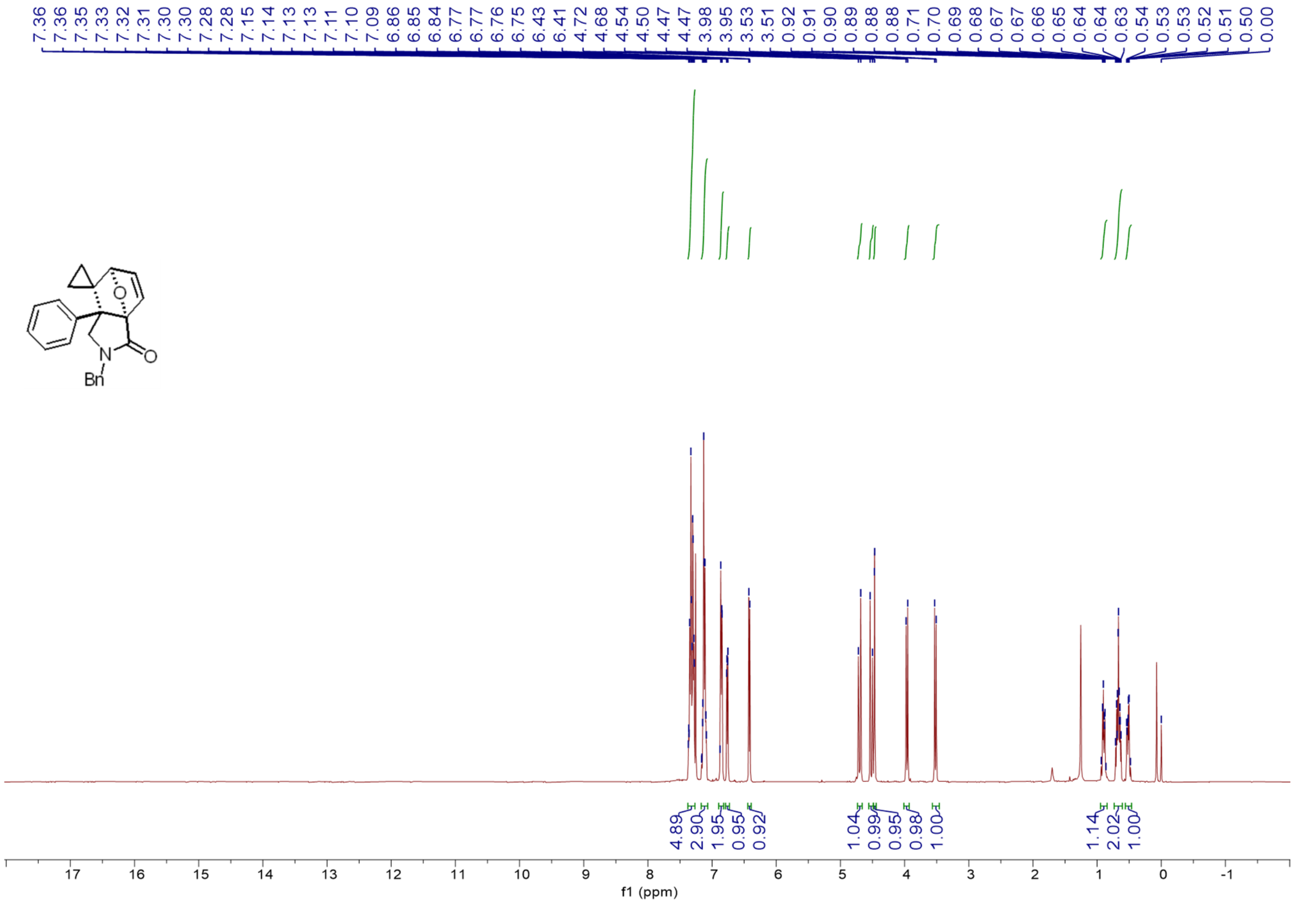
7.36
7.36
7.35
7.33
7.32
7.31
7.30
7.30
7.28
7.28
7.15
7.14
7.13
7.13
7.11
7.10
7.09
6.86
6.85
6.84
6.77
6.77
6.76
6.75
6.43
6.41
4.72
4.68
4.54
4.50
4.47
4.47
3.98
3.95
3.53
3.51
0.92
0.91
0.90
0.89
0.88
0.88
0.71
0.70
0.69
0.68
0.67
0.67
0.66
0.65
0.64
0.64
0.63
0.54
0.53
0.53
0.52
0.51
0.50
0.00
O
N
O
Bn
4.89
2.90
1.95
0.95
0.92
1.04
0.99
0.95
0.98
1.00
1.14
2.02
1.00
f1 (ppm)

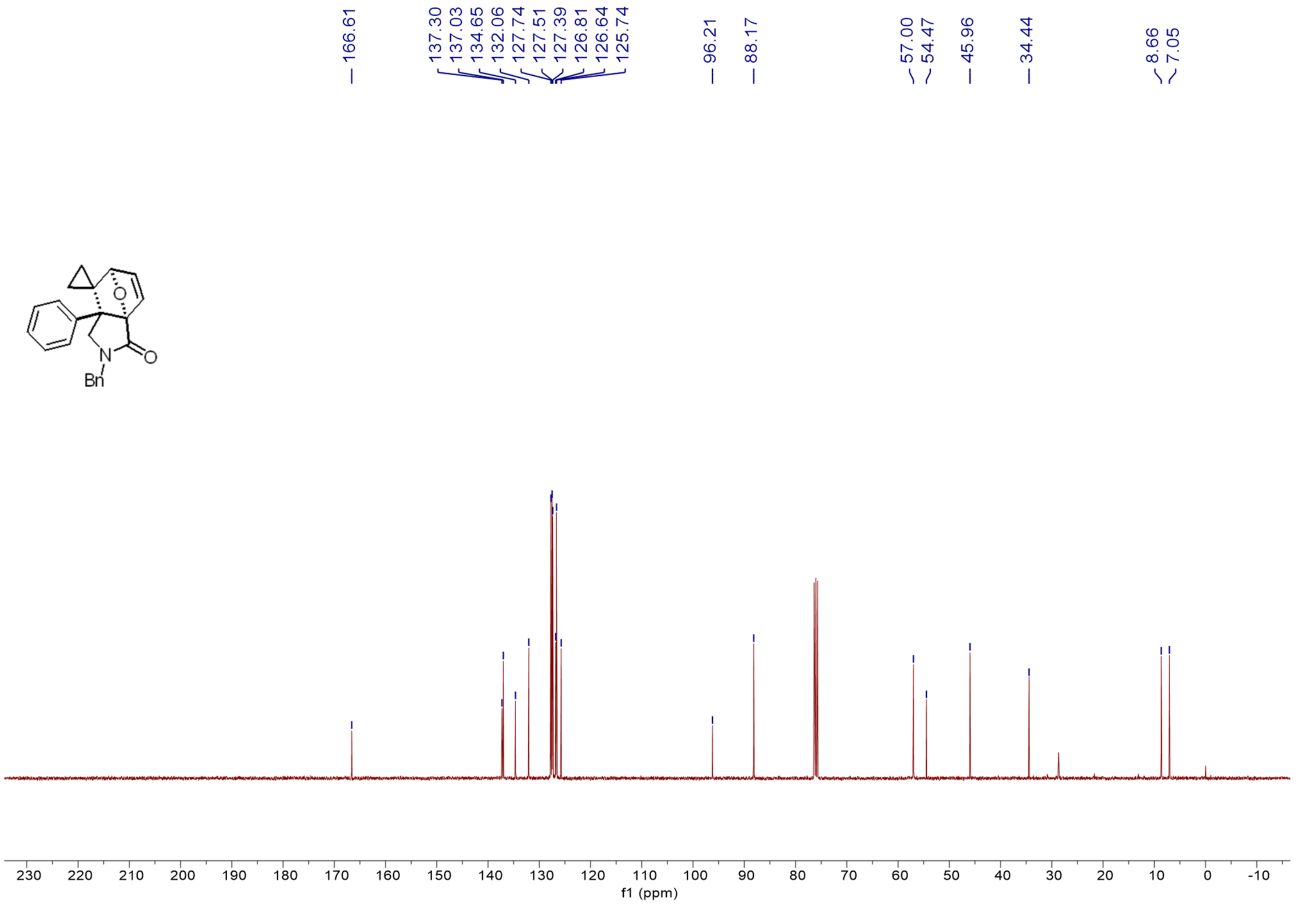
166.61
137.30
137.03
134.65
132.06
127.74
127.51
127.39
126.81
126.64
125.74
96.21
88.17
57.00
54.47
45.96
34.44
8.66
7.05
O
N
O
Bn
f1 (ppm)

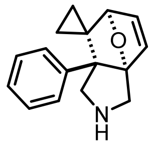

Compound 3v: Yield: 301.5 mg, 42%; A yellow oil; ^{1}H NMR (400 MHz, $CDCl_3$) δ 7.33 - 7.21 (m, 4H), 7.17 (t, *J* = 7.2 Hz, 1H), 6.70 (d, *J* = 6.0 Hz, 0H), 6.18 (d, *J* = 6.0 Hz, 1H), 4.32 (s, 1H), 3.59 (d, *J* = 10.2 Hz, 1H), 3.49 - 3.38 (m, 2H), 3.28 (d, *J* = 12.8 Hz, 1H), 2.42 (s, 1H), 0.89 - 0.79 (m, 1H), 0.72 - 0.60 (m, 2H), 0.57 - 0.46 (m, 1H). ^{13}C NMR (100 MHz, $CDCl_3$) δ 140.7, 137.2, 135.2, 128.6, 127.4, 126.2, 102.1, 87.4, 59.3, 58.3, 47.4, 36.5, 10.0, 8.4; IR (neat): ν 2962, 2868, 1704, 1260, 1052, 1028, 1000, 894, 797, 743, 702 cm^{-1}; HRMS (ESI) Calcd. for $C_{18}H_{18}NO$ $[M+H]^+$: 240.1383, Found: 240.1382.

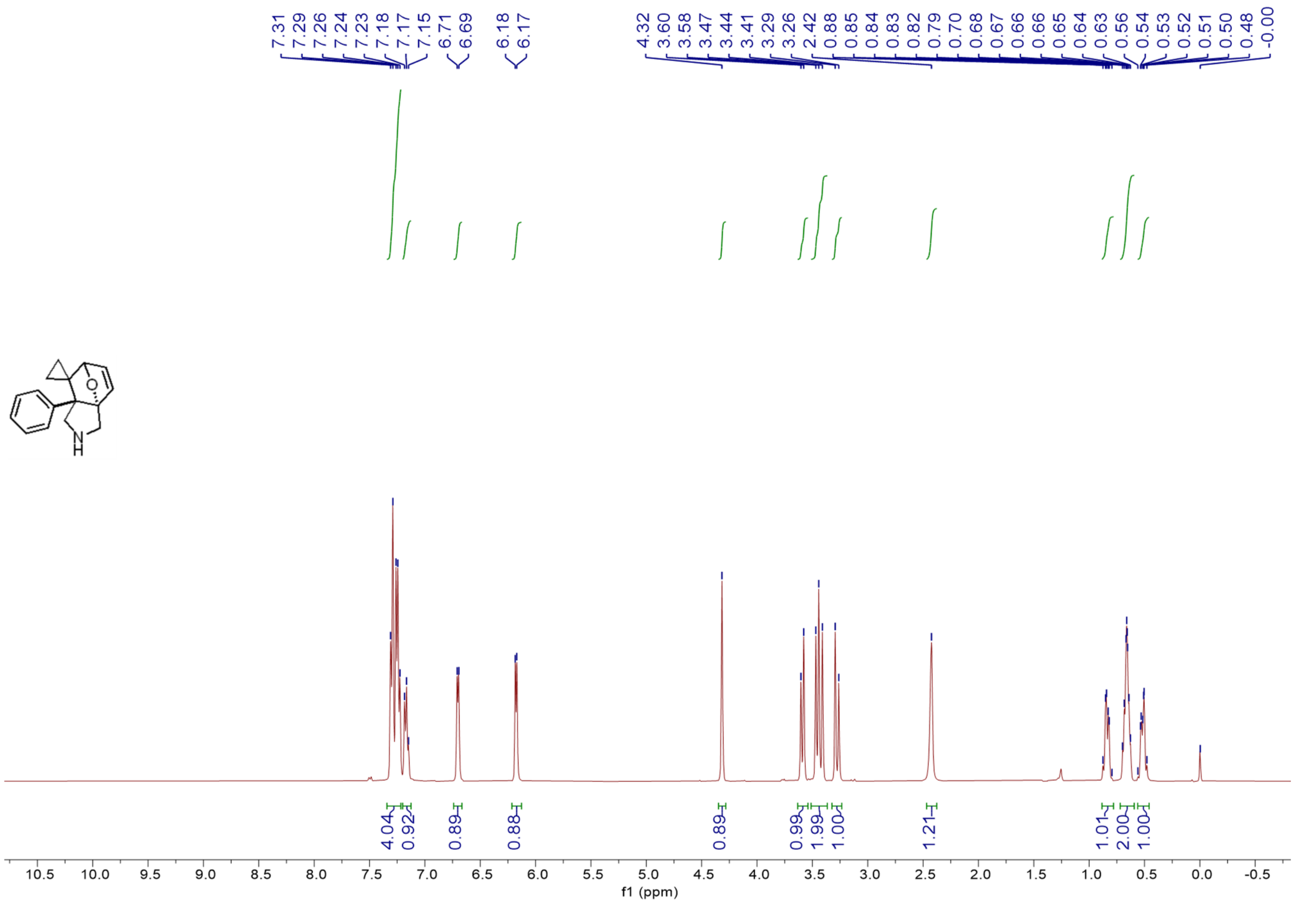
7.31 7.29 7.26 7.24 7.23 7.18 7.17 7.15 6.71 6.69 6.18 6.17
4.32 3.60 3.58 3.47 3.44 3.41 3.29 3.26 2.42 0.88 0.85 0.84 0.83 0.82 0.79 0.70 0.68 0.67 0.66 0.66 0.65 0.64 0.63 0.56 0.54 0.53 0.52 0.51 0.50 0.48 -0.00
O
N
H
4.04 0.92 0.89 0.88 0.89 0.99 1.99 1.00 1.21 1.01 2.00 1.00
f1 (ppm)

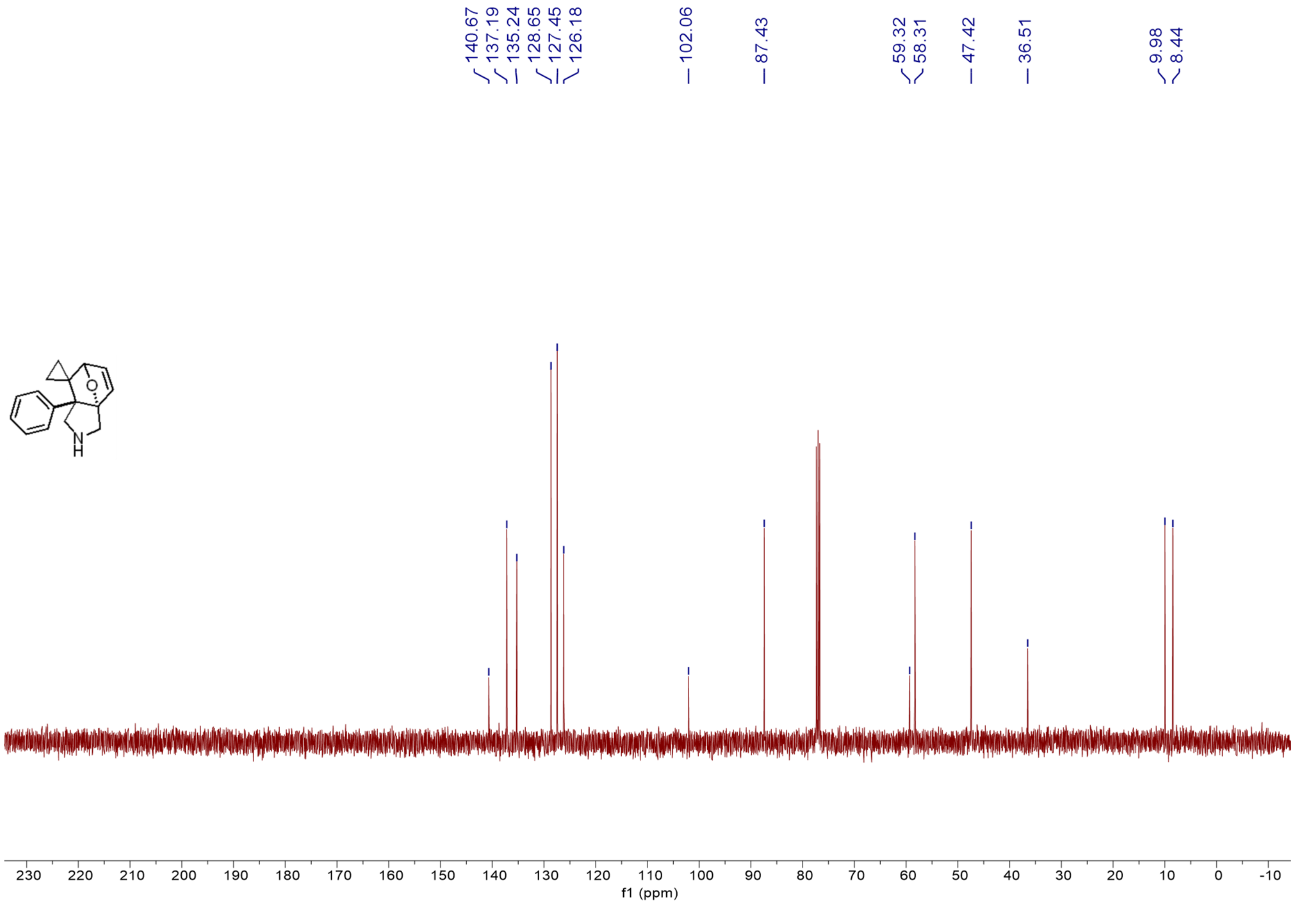
140.67 137.19 135.24 128.65 127.45 126.18 102.06 87.43 59.32 58.31 47.42 36.51 9.98 8.44
O
N
H
f1 (ppm)

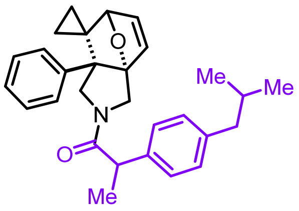

Compound 4a: Yield: 19.1 mg, 45%; A yellow oil; ^{1}H NMR (400 MHz, $CDCl_3$) (mixture of diastereomers) δ 7.32 (d, *J* = 7.8 Hz, 1H), 7.28 - 7.05 (m,5H), 7.02 (q, *J* = 7.2 Hz, 1.5H), 6.86 (t, *J* = 7.2 Hz, 1.5H), 6.75 - 6.66 (m, 1H), 6.51 (d, *J* = 7.8 Hz, 1H), 6.08 (d, *J* = 5.6 Hz, 0.2H), 6.02 (d, *J* = 5.6 Hz, 0.4H), 5.89 (d, *J* = 5.6 Hz, 0.3H), 4.54 (d, *J* = 12.2 Hz, 0.1H), 4.44 - 4.30 (m, 1.2H), 4.24 (s, 0.1H), 4.14 - 3.66 (m, 3.7H), 3.98 - 3.85 (m, 0.5H), 3.85 - 3.74 (m, 0.3H), 3.70 (t, *J* = 6.8 Hz, 0.5H), 3.59 (d, *J* = 13.8 Hz, 0.5H), 3.32 (d, *J* = 12.2 Hz, 0.3H), 2.47 (dd, *J* = 7.2, 2.8 Hz, 1H), 2.42 (dd, *J* = 7.2, 2.8 Hz, 1H), 1.94 - 1.78 (m, 1H), 1.58 - 1.45 (m, 3H), 1.44 - 1.32 (m, 0.4H), 0.96 - 0.80 (m, 7.2H), 0.77 - 0.66 (m, 1.7H), 0.64 - 0.56 (m, 0.5H), 0.55 - 0.41 (m, 1.2H). ^{13}C NMR (100 MHz, $CDCl_3$) δ 172.24, 172.15, 140.5, 140.2, 138.7, 138.5, 138.3, 138.1, 137.5, 134.4, 134.1, 129.7, 129.6, 129.53, 129.46, 128.3, 128.04, 128.00, 127.8, 127.7, 127.6, 127.4, 127.33, 127.28, 127.2, 127.1, 126.45, 126.40, 99.4, 97.9, 87.8, 87.7, 57.9, 57.0, 56.9, 55.8, 47.6, 47.2, 45.1, 45.0, 44.7, 44.2, 36.5, 36.3, 36.04, 36.01, 30.22, 30.18, 22.5, 22.4, 22.4, 20.4, 20.1, 10.2, 10.1, 8.8, 8.7; IR (neat): ν 2955, 2928, 2868, 1643, 1426, 1022, 844, 741, 703 cm^{-1}; HRMS (ESI) Calcd. for $C_{29}H_{33}NO_2Na$ $[M+H]^+$: 450.2404, Found: 450.2405.

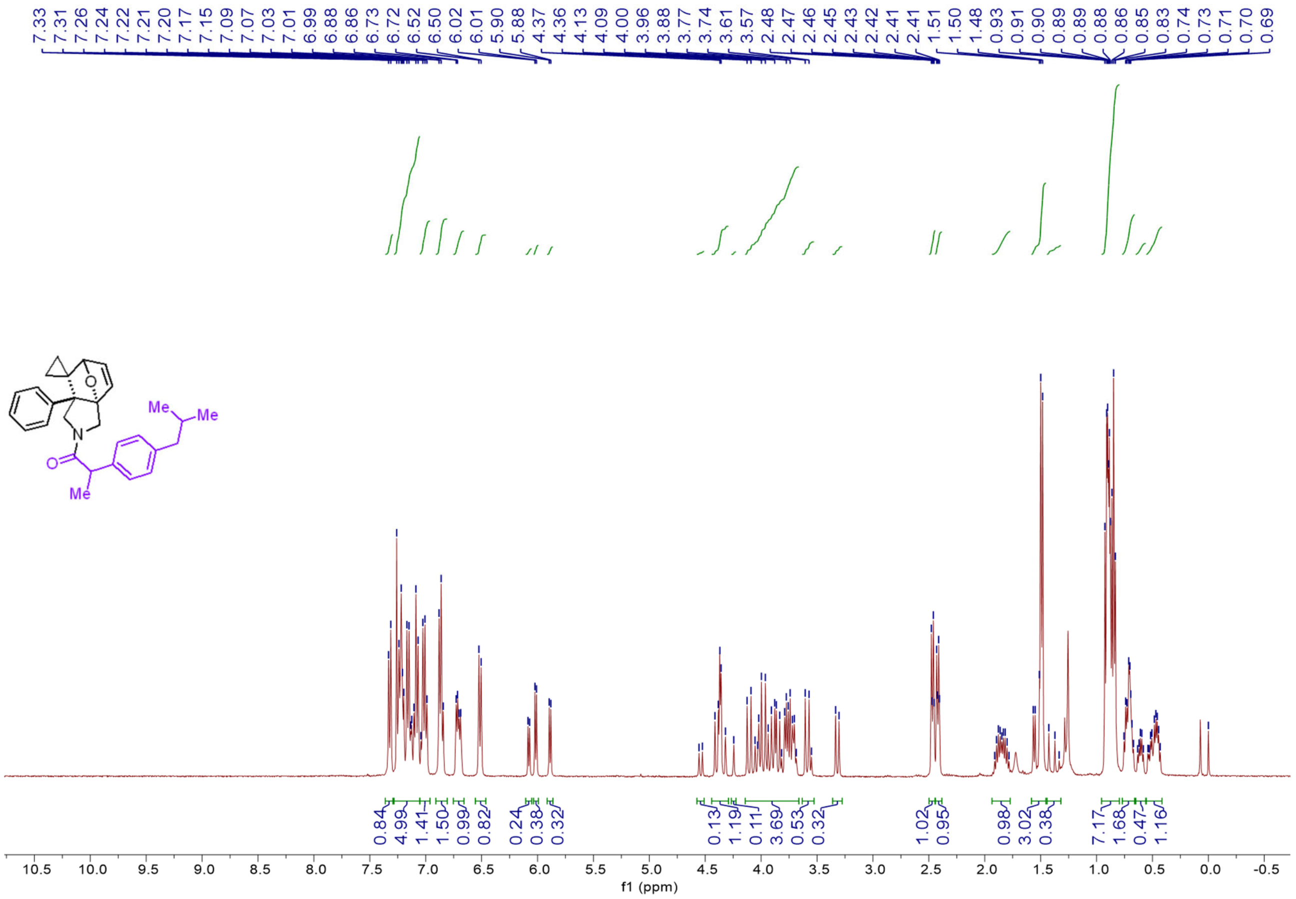

7.33 7.31 7.26 7.24 7.22 7.21 7.20 7.17 7.15 7.09 7.07 7.03 7.01 6.99 6.88 6.86 6.73 6.72 6.52 6.50 6.02 6.01 5.90 5.88 4.37 4.36 4.13 4.09 4.00 3.96 3.88 3.77 3.74 3.61 3.57 2.48 2.47 2.46 2.45 2.43 2.42 2.41 1.51 1.50 1.48 0.93 0.91 0.90 0.89 0.88 0.86 0.85 0.83 0.74 0.73 0.71 0.70 0.69
Me
Me
O
N
O
Me
0.84 4.99 1.41 1.50 0.99 0.82 0.24 0.38 0.32 0.13 1.19 0.11 3.69 0.53 0.32 1.02 0.95 0.98 3.02 0.38 7.17 1.68 0.47 1.16
10.5 10.0 9.5 9.0 8.5 8.0 7.5 7.0 6.5 6.0 5.5 5.0 4.5 4.0 3.5 3.0 2.5 2.0 1.5 1.0 0.5 0.0 -0.5
f1 (ppm)

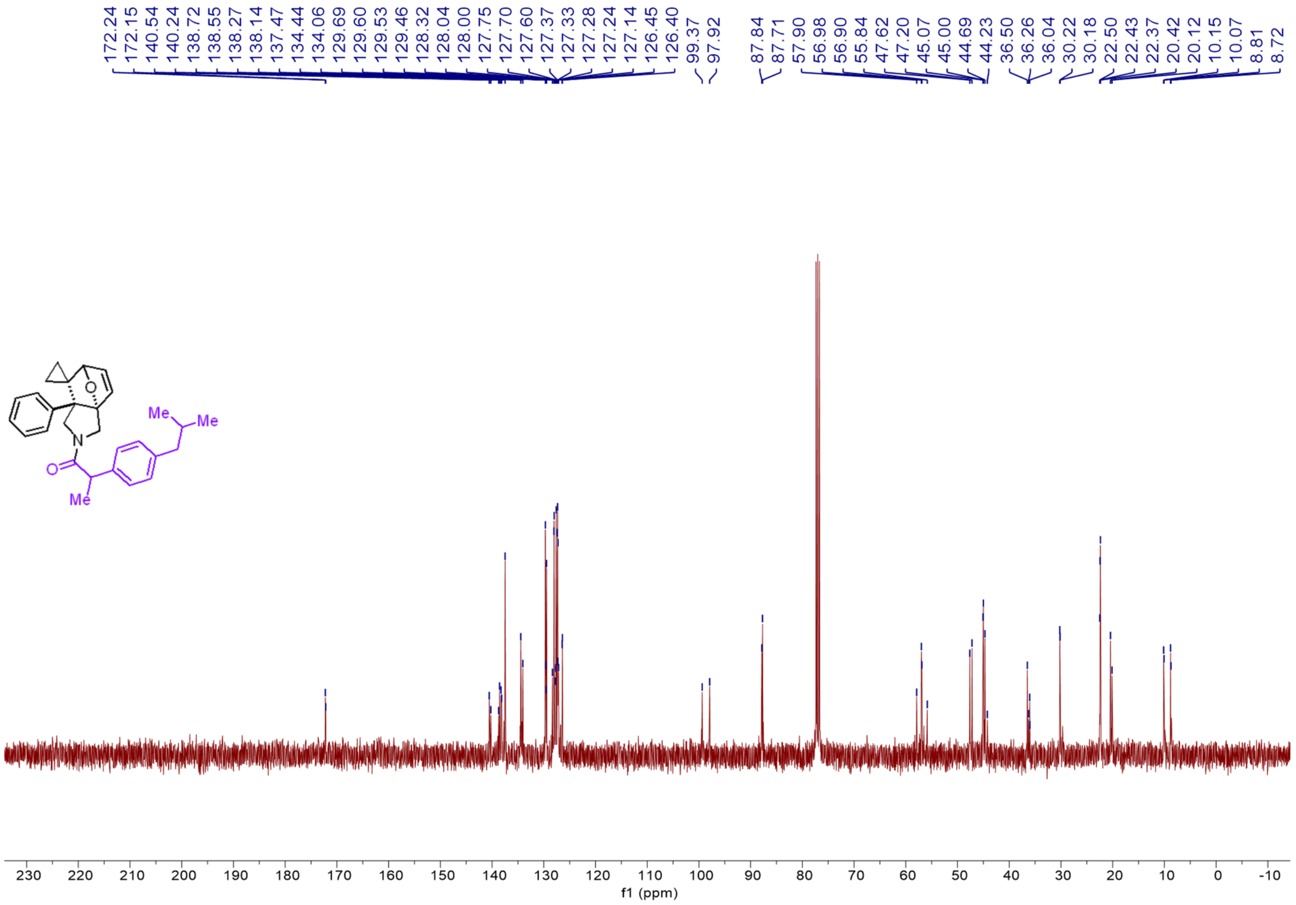

172.24 172.15 140.54 140.24 138.72 138.55 138.27 138.14 137.47 134.44 134.06 129.69 129.60 129.53 129.46 128.32 128.04 128.00 127.75 127.70 127.60 127.37 127.33 127.28 127.24 127.14 126.45 126.40 99.37 97.92 87.84 87.71 57.90 56.98 56.90 55.84 47.62 47.20 45.07 45.00 44.69 44.23 36.50 36.26 36.04 30.22 30.18 22.50 22.43 22.37 20.42 20.12 10.15 10.07 8.81 8.72
Me
Me
O
N
O
Me
230 220 210 200 190 180 170 160 150 140 130 120 110 100 90 80 70 60 50 40 30 20 10 0 -10
f1 (ppm)

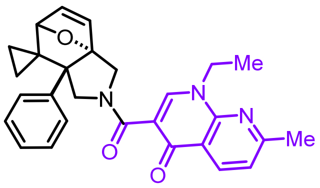

Compound 4b: Yield: 24.1 mg, 53%; A yellow oil; ^{1}H NMR (400 MHz, $CDCl_3$) (mixture of diastereomers) δ 8.65 (d, *J* = 8.2 Hz, 0.3H), 8.57 (d, *J* = 8.2 Hz, 0.6H), 8.24 (s, 0.6H), 8.17 (s, 0.3H), 7.49 (d, *J* = 7.8 Hz, 0.6H), 7.37 - 7.28 (m, 3.4H), 7.26 - 7.17 (m, 2H), 6.76 (dd, *J* = 5.8, 1.8 Hz, 0.3H), 6.72 (dd, *J* = 5.8, 1.8 Hz, 0.6H), 6.14 (d, *J* = 5.8 Hz, 0.3H), 6.00 (d, *J* = 5.8 Hz, 0.6H), 4.62 - 4.28 (m, 5H), 4.11 (d, *J* = 12.4 Hz, 0.6H), 3.99 (d, *J* = 10.8 Hz, 0.3H), 3.88 (d, *J* = 14.0 Hz, 0.3H), 3.70 (d, *J* = 13.0 Hz, 0.6H), 2.68 - 2.65 (m, 3H), 1.56 - 1.46 (m, 3H), 0.93 - 0.44 (m, 4H). ^{13}C NMR (100 MHz, $CDCl_3$) δ 173.9, 173.7, 166.1, 165.9, 162.6, 148.6, 145.5, 144.7, 139.2, 139.2, 137.4, 137.2, 136.5, 136.2, 134.6, 134.4, 128.7, 128.5, 127.7, 127.6, 126.7, 126.6, 120.7, 120.6, 120.34, 120.32, 119.9, 99.5, 98.2, 87.83, 87.75, 67.9, 58.2, 58.0, 57.9, 56.2, 48.4, 47.9, 46.3, 46.2, 36.5, 36.4, 25.6, 25.1, 15.2, 15.1, 10.3, 10.2, 9.0, 8.8; IR (neat): ν 3481, 3065, 2929, 1709, 1641, 1525, 1452, 1251, 1051, 842, 742, 699 cm^{-1}; HRMS (ESI) Calcd. for $C_{28}H_{28}N_3O_3$ $[M+H]^+$: 454.2125, Found: 454.2125.

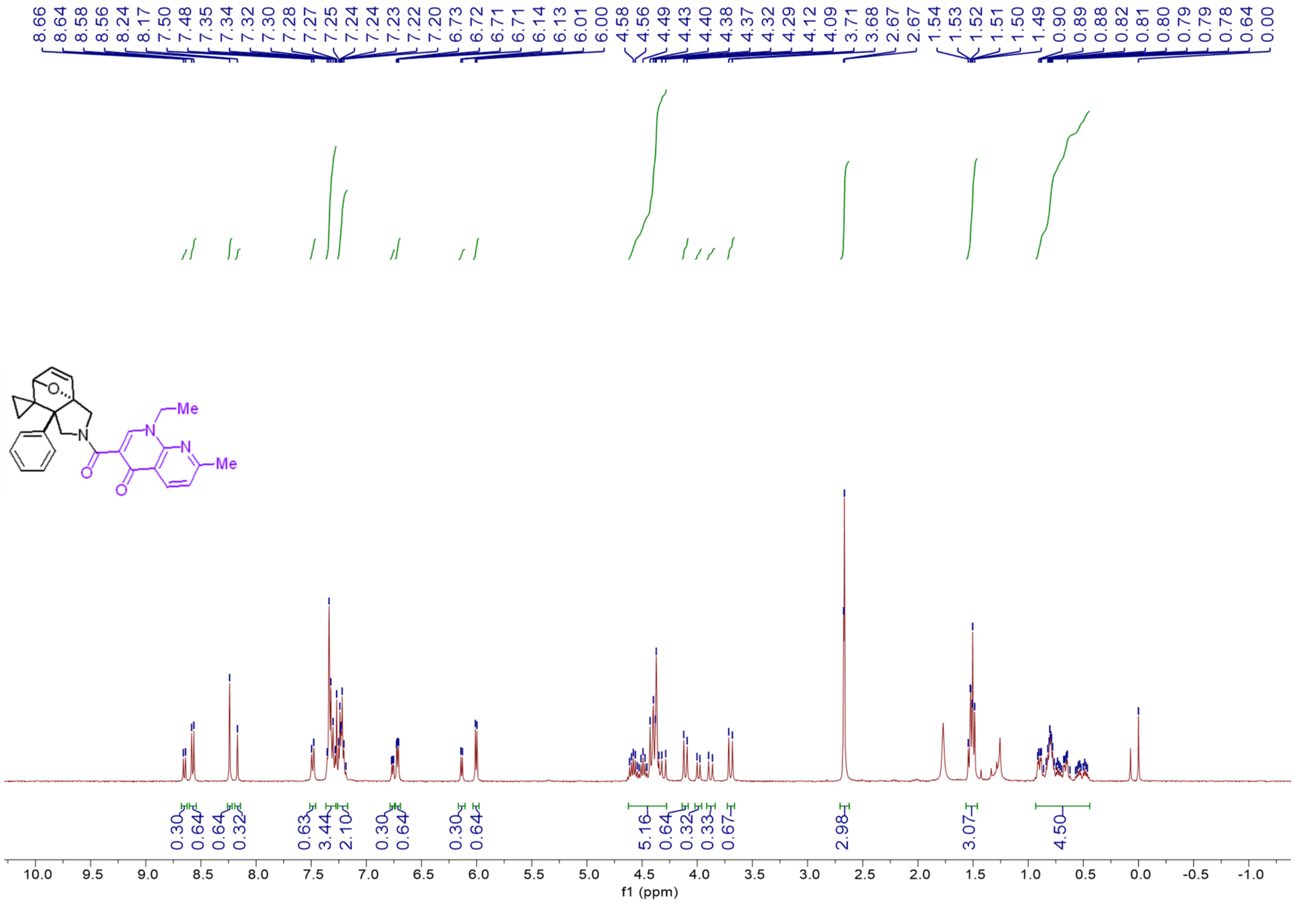
8.66 8.64 8.58 8.56 8.24 8.17 7.50 7.48 7.35 7.34 7.32 7.30 7.28 7.27 7.25 7.24 7.24 7.23 7.22 7.20 6.73 6.72 6.71 6.71 6.14 6.13 6.01 6.00 4.58 4.56 4.49 4.43 4.40 4.38 4.37 4.32 4.29 4.12 4.09 3.71 3.68 2.67 1.54 1.53 1.52 1.51 1.50 1.49 0.90 0.89 0.88 0.82 0.81 0.80 0.79 0.79 0.78 0.64 0.00
O
N
Me
N
N
Me
O
O
0.30 0.64 0.64 0.32 0.63 3.44 2.10 0.30 0.64 0.30 0.64 5.16 0.64 0.32 0.33 0.67 2.98 3.07 4.50
10.0 9.5 9.0 8.5 8.0 7.5 7.0 6.5 6.0 5.5 5.0 4.5 4.0 3.5 3.0 2.5 2.0 1.5 1.0 0.5 0.0 -0.5 -1.0
f1 (ppm)

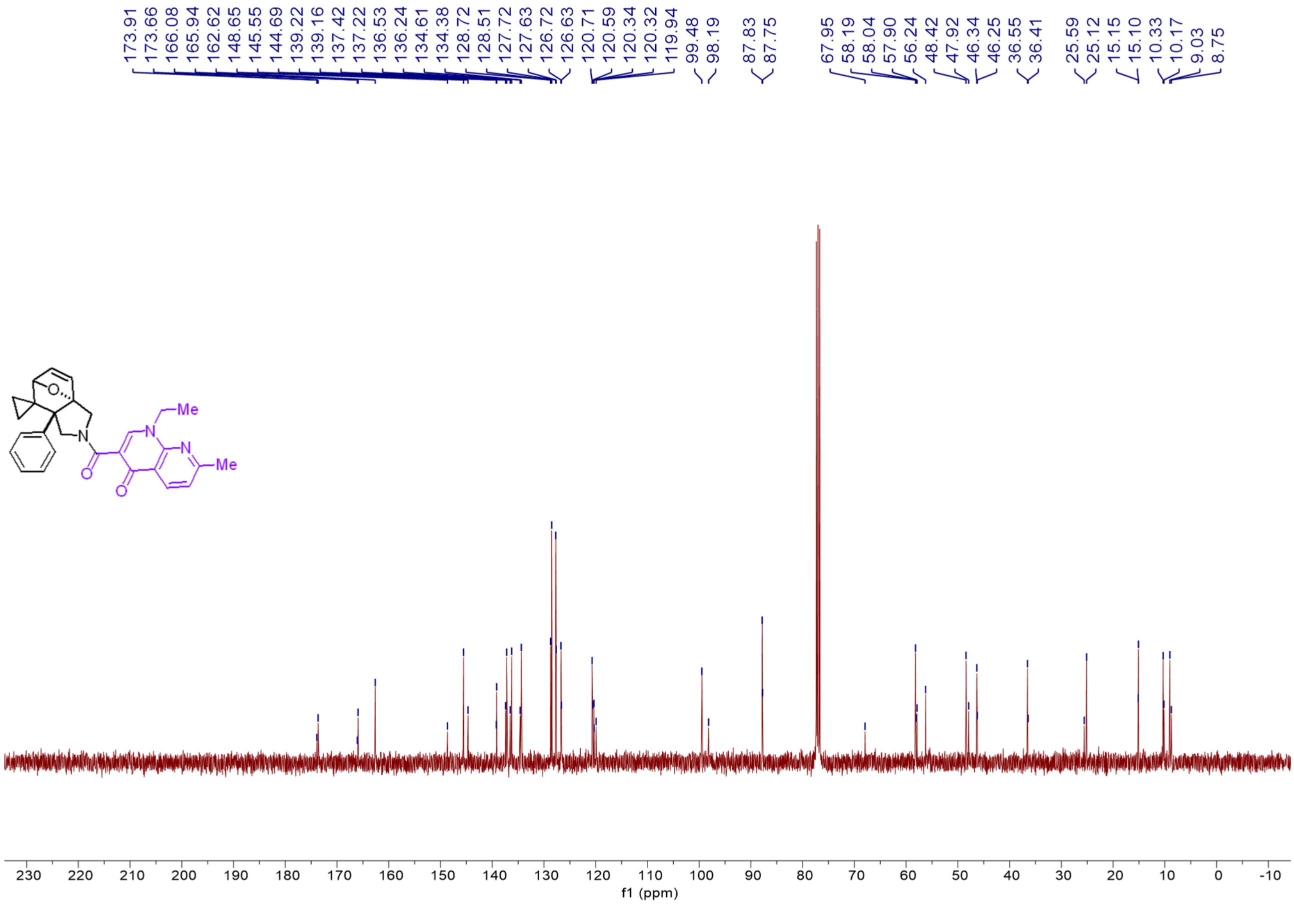
173.91 173.66 166.08 165.94 162.62 148.65 145.55 144.69 139.22 139.16 137.42 137.22 136.53 136.24 134.61 134.38 128.72 128.51 127.72 127.63 126.72 126.63 120.71 120.59 120.34 120.32 119.94 99.48 98.19 87.83 87.75 67.95 58.19 58.04 57.90 56.24 48.42 47.92 46.34 46.25 36.55 36.41 25.59 25.12 15.15 15.10 10.33 10.17 9.03 8.75
O
N
Me
N
N
Me
O
O
230 220 210 200 190 180 170 160 150 140 130 120 110 100 90 80 70 60 50 40 30 20 10 0 -10
f1 (ppm)

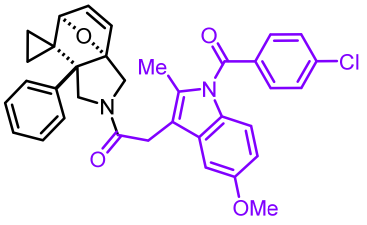

Compound 4c: Yield: 50.0 mg, 91%; A yellow oil; ^{1}H NMR (400 MHz, $CDCl_3$) (mixture of diastereomers) δ 7.60 (dd, *J* = 8.2, 6.6 Hz, 2H), 7.42 (dd, *J* = 8.2, 6.6 Hz, 2H), 7.18 (dd, *J* = 6.4, 2.6 Hz, 1H), 7.14 - 7.08 (m, 1H), 7.07 - 6.97 (m, 3H), 6.91 - 6.82 (m, 2H), 6.77 - 6.72 (m, 1H), 6.71 (dd, *J* = 2.6, 1.2 Hz, 0.5H), 6.68 (dd, *J* = 2.6, 1.2 Hz, 0.4H), 6.07 (d, *J* = 5.8 Hz, 0.4H), 6.03 (d, *J* = 5.8 Hz, 0.5H), 4.44 (d, *J* = 12.1 Hz, 0.5H), 4.40 - 4.37 (m, 1H), 4.15 - 4.08 (m, 1H), 4.04 (d, *J* = 10.1 Hz, 0.5H), 3.96 - 3.88 (m, 1H), 3.88 - 3.77 (m, 4H), 3.76 - 3.64 (m, 2H), 2.43 (s, 1.3H), 2.39 (s, 1.6H), 0.94 - 0.84 (m, 1H), 0.80 - 0.65 (m, 1.6H), 0.61 - 0.47 (m, 1.5H) ^{13}C NMR (100 MHz, $CDCl_3$) δ 168.6, 168.5, 168.25, 168.22, 156.2, 156.1, 139.21, 139.19, 138.8, 138.7, 137.8, 137.6, 135.3, 135.1, 134.3, 133.93, 133.88, 131.20, 131.17, 130.8, 130.7, 130.6, 129.07, 129.05, 128.1, 127.8, 127.7, 127.6, 126.8, 126.7, 114.93, 114.89, 112.7, 112.5, 112.11, 112.06, 101.6, 101.4, 99.5, 98.0, 87.8, 87.6, 58.2, 57.9, 57.0, 56.0, 55.73, 55.69, 47.9, 47.5, 36.3, 36.2, 32.1, 13.51, 13.46, 10.1, 10.1, 8.8, 8.7; IR (neat): ν 2991, 1641, 1356, 1315, 1221, 1088, 1014, 841, 742, 703 cm^{-1}; HRMS (ESI) Calcd. for $C_{35}H_{32}N2O_4Cl$ $[M+H]^+$: 579.2045, Found: 579.2053.

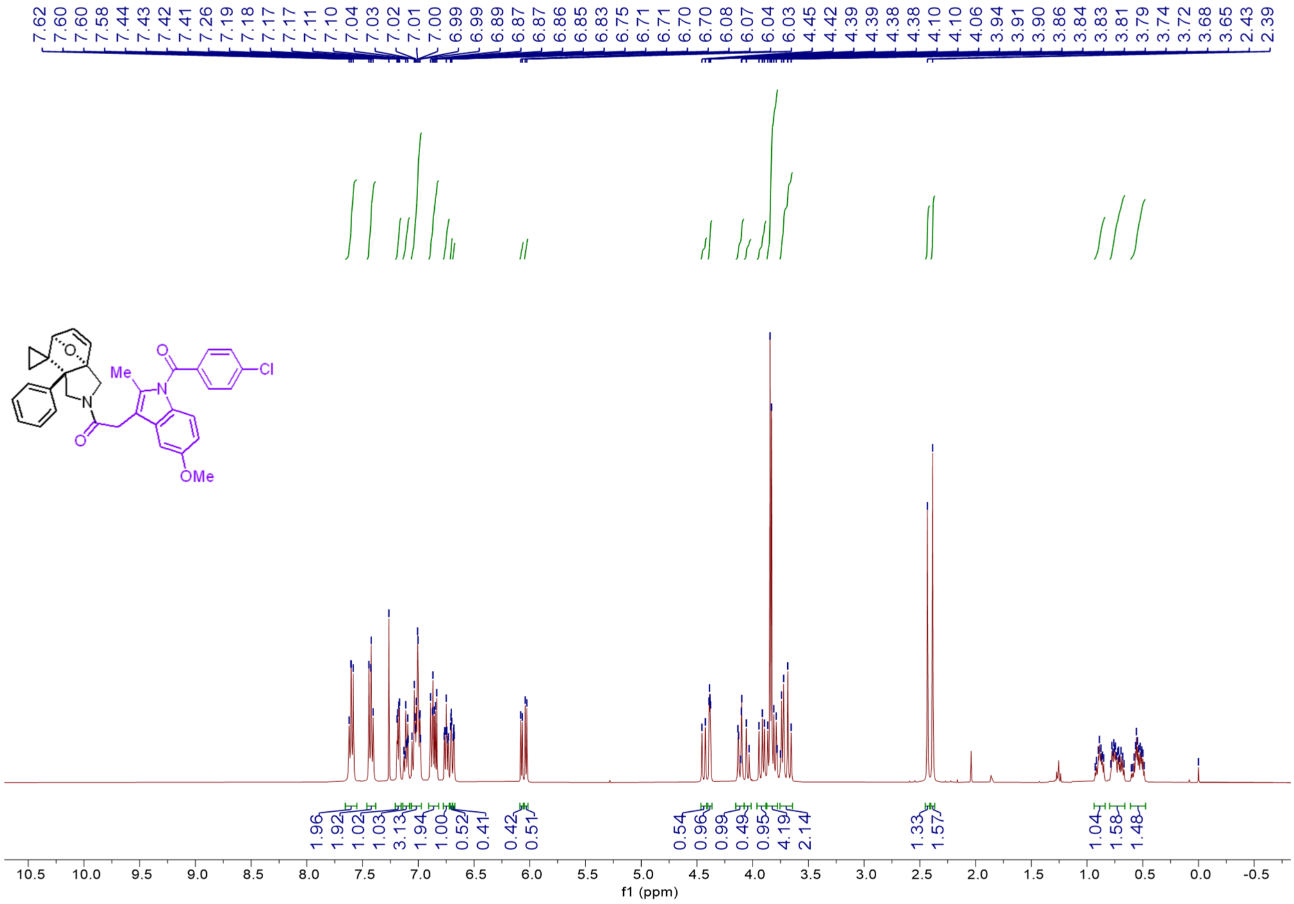
7.62 7.60 7.60 7.58 7.44 7.43 7.42 7.41 7.26 7.19 7.18 7.17 7.11 7.10 7.04 7.03 7.02 7.01 7.00 6.99 6.89 6.87 6.87 6.86 6.85 6.83 6.75 6.71 6.71 6.70 6.70 6.08 6.07 6.04 6.03 4.45 4.42 4.39 4.39 4.38 4.38 4.10 4.10 4.06 3.94 3.91 3.90 3.86 3.84 3.83 3.81 3.79 3.74 3.72 3.68 3.65 2.43 2.39
Me
O
N
Cl
OMe
1.96 1.92 1.02 1.03 3.13 1.94 1.00 0.52 0.41 0.42 0.51 0.54 0.96 0.99 0.49 0.95 4.19 2.14 1.33 1.57 1.04 1.58 1.48
10.5 10.0 9.5 9.0 8.5 8.0 7.5 7.0 6.5 6.0 5.5 5.0 4.5 4.0 3.5 3.0 2.5 2.0 1.5 1.0 0.5 0.0 -0.5
f1 (ppm)

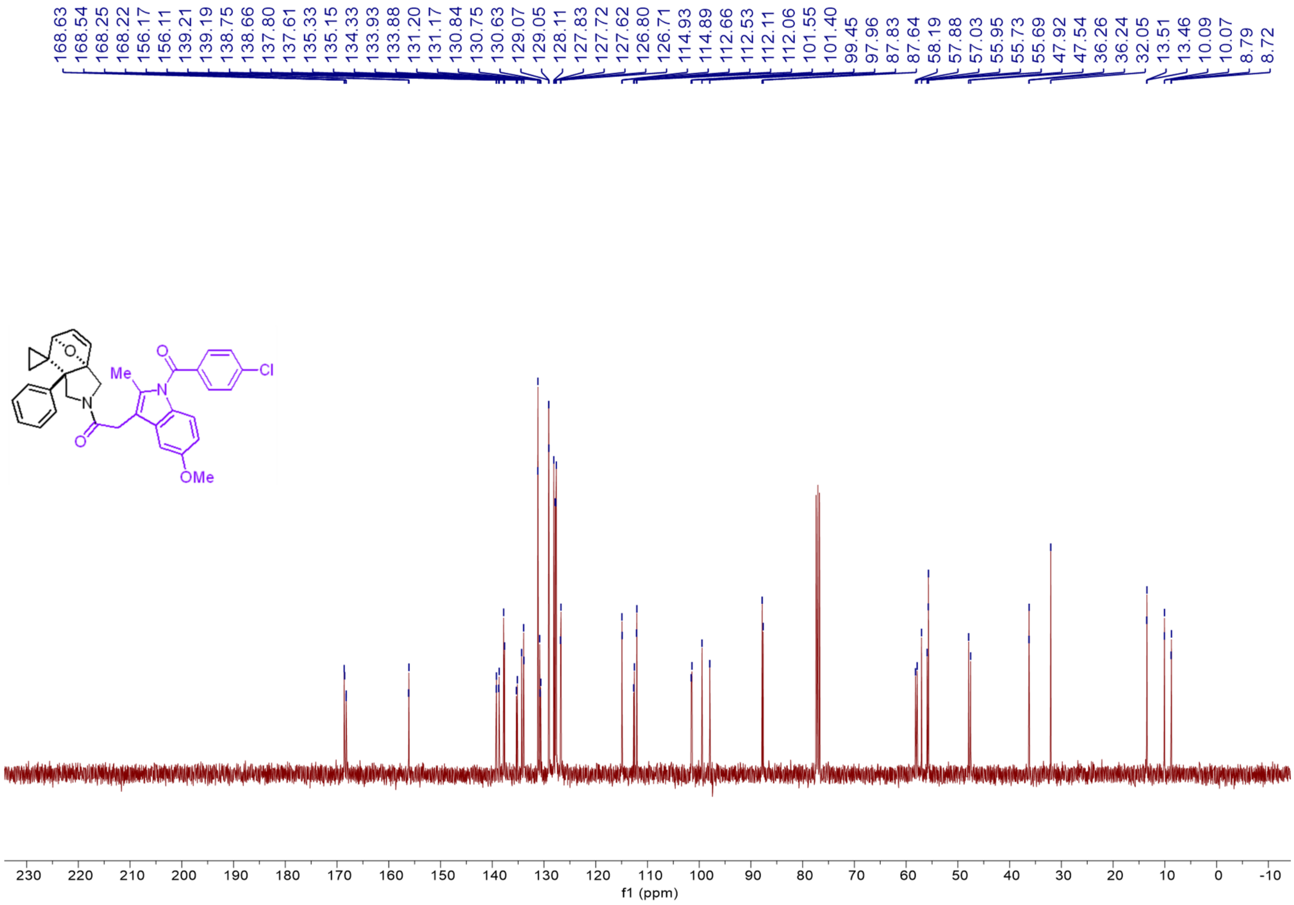
168.63 168.54 168.25 168.22 156.17 156.11 139.21 139.19 138.75 138.66 137.80 137.61 135.33 135.15 134.33 133.93 133.88 131.20 131.17 130.84 130.75 130.63 129.07 129.05 128.11 127.83 127.72 127.62 126.80 126.71 114.93 114.89 112.66 112.53 112.11 112.06 101.55 101.40 99.45 97.96 87.83 87.64 58.19 57.88 57.03 55.95 55.73 55.69 47.92 47.54 36.26 36.24 32.05 13.51 13.46 10.09 10.07 8.79 8.72
Me
O
N
Cl
OMe
230 220 210 200 190 180 170 160 150 140 130 120 110 100 90 80 70 60 50 40 30 20 10 0 -10
f1 (ppm)

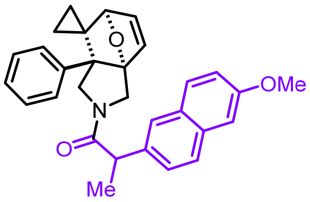

Compound 4d: Yield: 40.8 mg, 90%; A yellow oil; ^{1}H NMR (400 MHz, $CDCl_3$) (mixture of diastereomers) δ 7.81 (d, *J* = 1.8 Hz, 0.4H), 7.78 - 7.65 (m, 2.5H), 7.50 (dd, *J* = 8.4, 1.8 Hz, 0.4H), 7.47 - 7.40 (m, 0.6H), 7.26 - 7.06 (m, 3H), 6.99 - 6.80 (m, 2.3H), 6.72 - 6.64 (m, 1H), 6.53 (t, *J* = 7.8 Hz, 0.7H), 6.39 (d, *J* = 7.8 Hz, 0.7H), 6.08 (d, *J* = 5.8 Hz, 0.2H), 6.06 (d, *J* = 5.8 Hz, 0.4H), 6.02 (d, *J* = 5.8 Hz, 0.4H), 5.83 (d, *J* = 5.8 Hz, 0.4H), 4.56 (d, *J* = 12.2 Hz, 0.1H), 4.46 (d, *J* = 12.2 Hz, 0.4H), 4.35 (dd, *J* = 4.8, 1.8 Hz, 0.7H), 4.26 (d, *J* = 1.8 Hz, 0.1H), 4.20 (d, *J* = 1.8 Hz, 0.1H), 4.18 - 3.99 (m, 1.2H), 3.97 - 3.70 (m, 5.4H), 3.67 - 3.59 (m, 0.5H), 3.28 (d, *J* = 12.2 Hz, 0.5H), 1.63 (d, *J* = 6.8 Hz, 0.3H), 1.61 - 1.53 (m, 2.6H), 0.91 - 0.78 (m, 0.9H), 0.76 - 0.64 (m, 1.6H), 0.61 - 0.41 (m, 1.1H), 0.40 - 0.32 (m, 0.4H); ^{13}C NMR (100 MHz, $CDCl_3$) δ 172.34, 172.25, 172.2, 172.0, 157.7, 157.6, 157.5, 139.2, 138.9, 138.8, 138.4, 137.7, 137.6, 137.50, 137.45, 136.31, 136.29, 136.12, 136.09, 134.5, 134.4, 134.1, 134.0, 133.7, 133.62, 133.58, 133.5, 129.3, 129.2, 129.12, 129.09, 128.3, 128.0, 127.96, 127.9, 127.8, 127.73, 127.67, 127.6, 127.44, 127.35, 127.2, 126.9, 126.8, 126.5, 126.41, 126.37, 126.3, 126.2, 126.0, 125.9, 125.7, 119.1, 119.0, 118.9, 105.6, 99.43, 99.40, 98.0, 97.9, 87.8, 87.7, 87.5, 58.2, 58.0, 57.1, 57.0, 56.6, 55.9, 55.3, 47.7, 47.6, 47.3, 45.5, 45.1, 45.0, 44.7, 36.5, 36.3, 36.1, 36.0, 20.4, 20.3, 20.1, 10.12, 10.09, 10.0, 9.9, 8.8, 8.7, 8.6; IR (neat): ν 2986, 1710, 1636, 1604, 1424, 1213, 1028, 893, 843, 741, 703 cm^{-1}; HRMS (ESI) Calcd. for $C_{30}H_{30}NO_3$ $[M+H]^+$: 352.2220, Found: 452.2219.

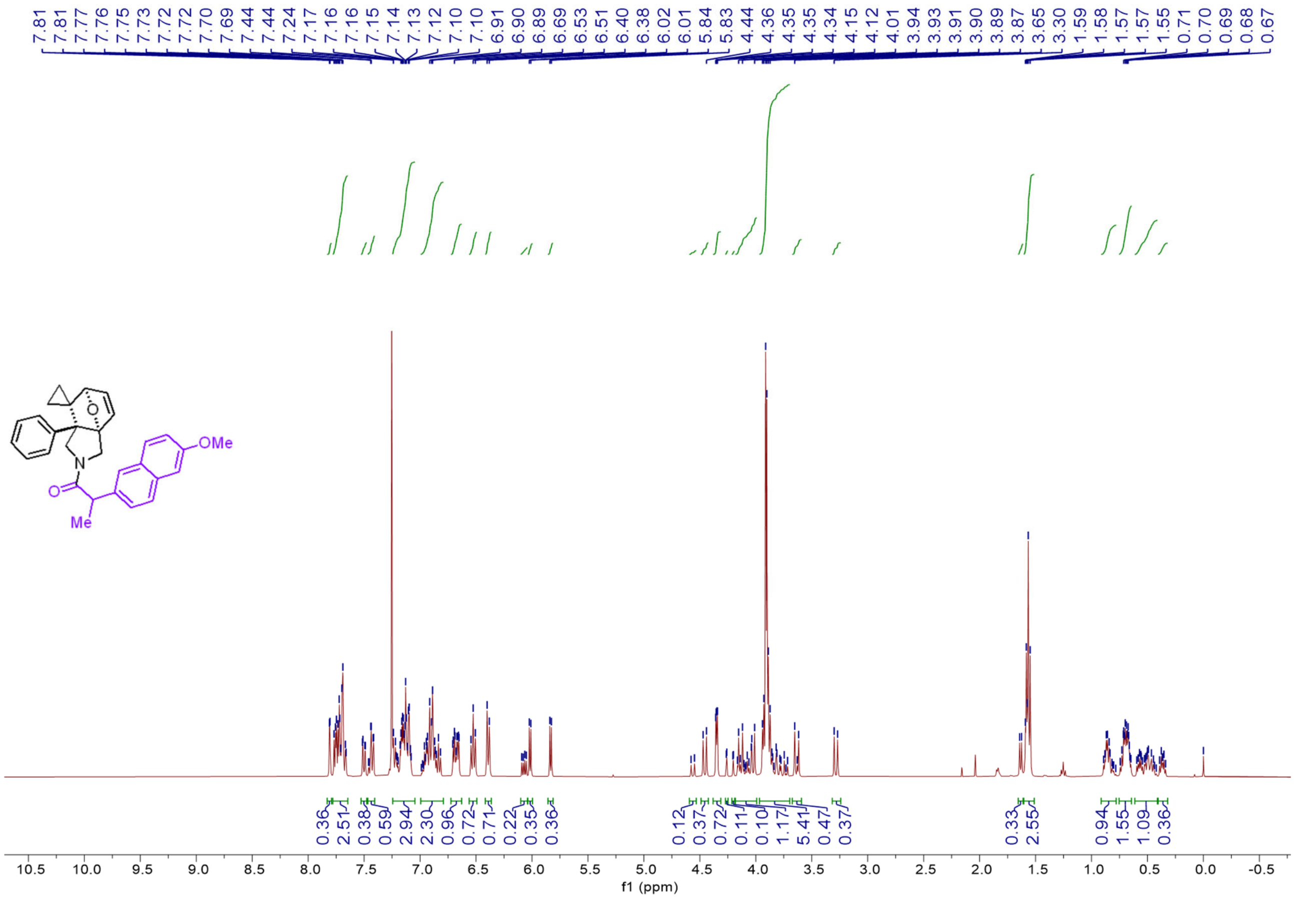
7.81 7.81 7.77 7.76 7.75 7.73 7.72 7.72 7.70 7.69 7.44 7.44 7.24 7.17 7.16 7.16 7.15 7.14 7.13 7.12 7.10 7.10 6.91 6.90 6.89 6.69 6.53 6.51 6.40 6.38 6.02 6.01 5.84 5.83 4.44 4.36 4.35 4.35 4.34 4.15 4.12 4.01 3.94 3.93 3.91 3.90 3.89 3.87 3.65 3.30 1.59 1.58 1.57 1.57 1.55 0.71 0.70 0.69 0.68 0.67
OMe
O
N
Me
0.36 2.51 0.38 0.59 2.94 2.30 0.96 0.72 0.71 0.22 0.35 0.36
0.12 0.37 0.72 0.11 0.10 1.17 5.41 0.47 0.37
0.33 2.55
0.94 1.55 1.09 0.36
10.5 10.0 9.5 9.0 8.5 8.0 7.5 7.0 6.5 6.0 5.5 5.0 4.5 4.0 3.5 3.0 2.5 2.0 1.5 1.0 0.5 0.0 -0.5
f1 (ppm)

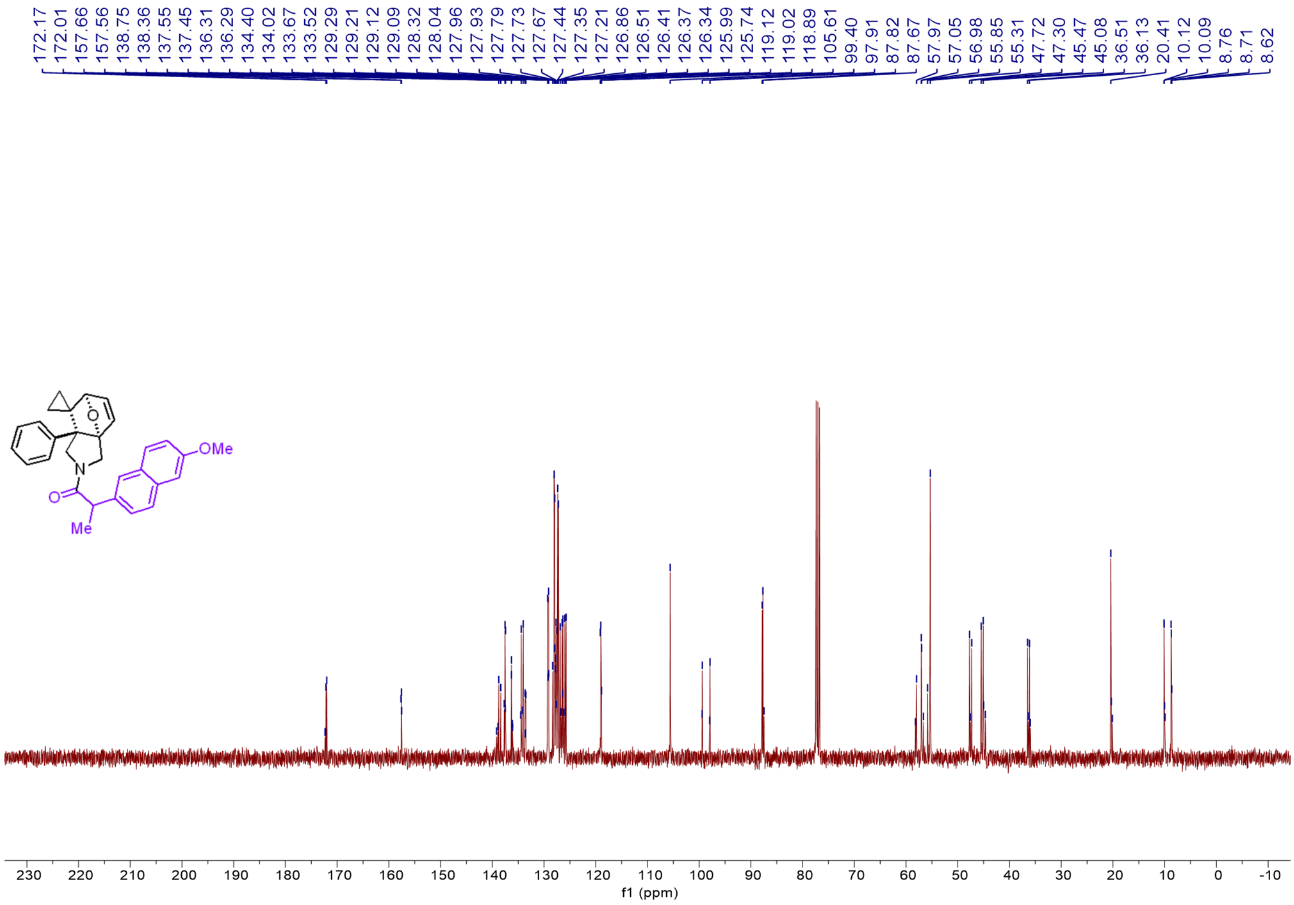
172.17 172.01 157.66 157.56 138.75 138.36 137.55 137.45 136.31 136.29 134.40 134.02 133.67 133.52 129.29 129.21 129.12 129.09 128.32 128.04 127.96 127.93 127.79 127.73 127.67 127.44 127.35 127.21 126.86 126.51 126.41 126.37 126.34 125.99 125.74 119.12 119.02 118.89 105.61 99.40 97.91 87.82 87.67 57.97 57.05 56.98 55.85 55.31 47.72 47.30 45.47 45.08 36.51 36.13 20.41 10.12 10.09 8.76 8.71 8.62
OMe
O
N
Me
230 220 210 200 190 180 170 160 150 140 130 120 110 100 90 80 70 60 50 40 30 20 10 0 -10
f1 (ppm)

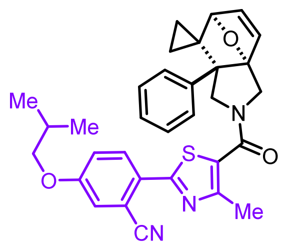

Compound 4e: Yield: 41.7 mg, 78%; A yellow oil; ^{1}H NMR (400 MHz, $CDCl_3$) (mixture of diastereomers) δ 8.20 - 7.99 (m, 2H), 7.33 - 7.17 (m, 5H), 7.10 - 6.94 (m, 2H), 6.79 (d, J = 5.8 Hz, 1H), 6.22 (d, J = 5.8 Hz, 0.5H), 6.08 (d, J = 5.8 Hz, 0.5H), 4.53 (d, J = 12.4 Hz, 0.5H), 4.43 (s, 1H), 4.19 - 4.05 (m, 2H), 4.03 - 3.79 (m, 3.5H), 2.63 (s, 1H), 2.59 (s, 1H), 2.21 (q, J = 7.0 Hz, 1H), 1.09 (d, J = 7.0 Hz, 2H), 0.97 - 0.88 (m, 1H), 0.83 - 0.69 (m, 1.5H), 0.66 - 0.52 (m, 1H), 0.51 - 0.42 (m, 0.5H); ^{13}C NMR (100 MHz, $CDCl_3$) δ 164.8, 164.7, 162.2, 161.8, 155.2, 154.8, 138.6, 138.4, 138.1, 138.0, 134.2, 133.7, 132.4, 131.9, 131.8, 128.2, 127.9, 127.8, 127.0, 126.0, 124.4, 124.3, 115.5, 112.6, 102.9, 99.0, 98.3, 87.81, 87.75, 75.6, 60.1, 58.2, 57.4, 56.4, 49.9, 47.9, 36.6, 36.2, 28.1, 19.0, 16.9, 10.1, 10.0, 8.7, 8.5; IR (neat): ν 2959, 2929, 1627, 1445, 1410, 1278, 1012, 741, 702 cm^{-1}; HRMS (ESI) Calcd. for $C_{32}H_{32}N_3O_3$ $[M+H]^+$: 538.2159, Found: 538.2156.

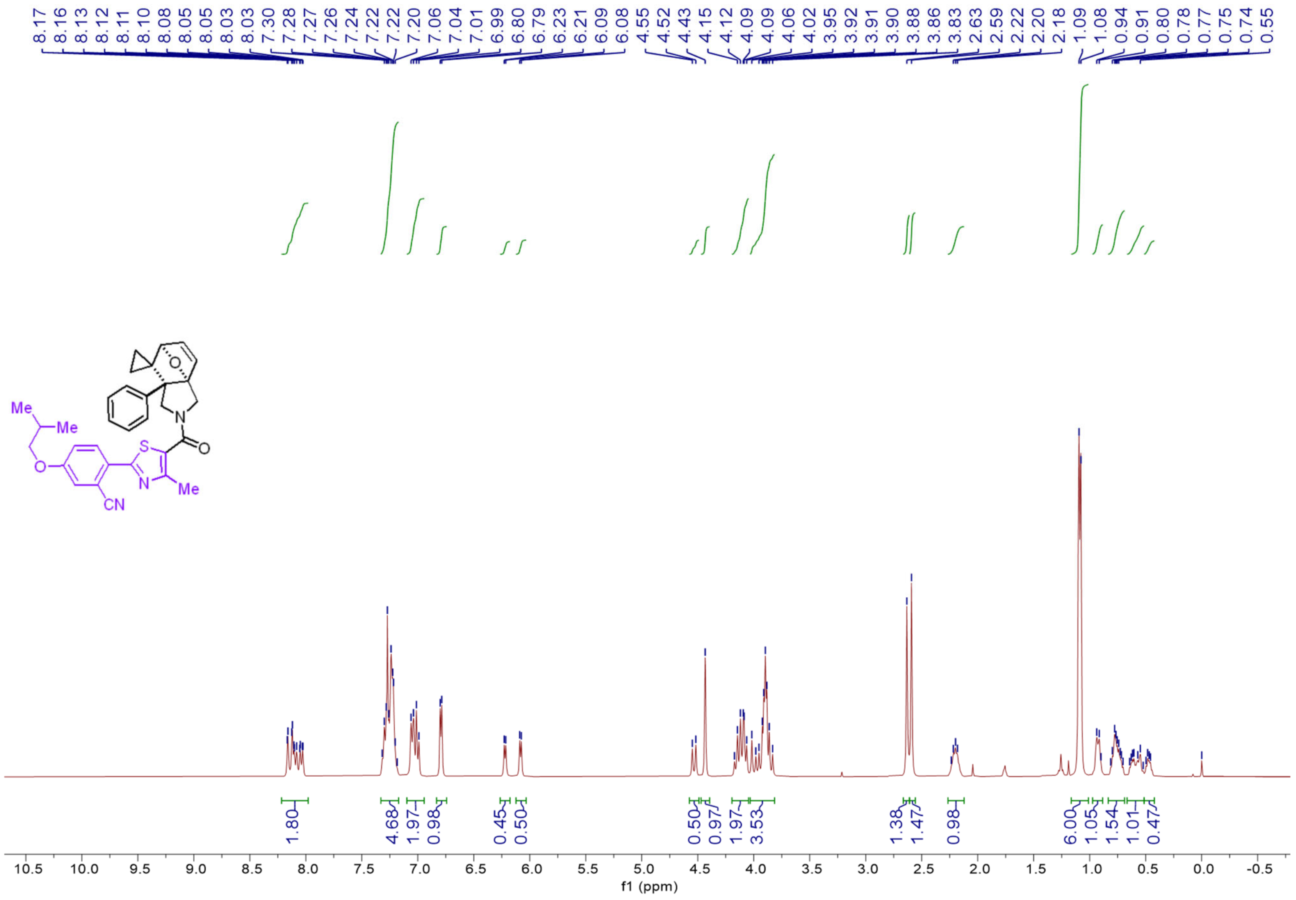

8.17 8.16 8.13 8.12 8.11 8.10 8.08 8.05 8.05 8.03 8.03 7.30 7.28 7.27 7.26 7.24 7.22 7.22 7.20 7.06 7.04 7.01 6.99 6.80 6.79 6.23 6.21 6.09 6.08 4.55 4.52 4.43 4.15 4.12 4.09 4.09 4.06 4.02 3.95 3.92 3.91 3.90 3.88 3.86 3.83 2.63 2.59 2.22 2.20 2.18 1.09 1.08 0.94 0.91 0.80 0.78 0.77 0.75 0.74 0.55
1.80 4.68 1.97 0.98 0.45 0.50 0.50 0.97 1.97 3.53 1.38 1.47 0.98 6.00 1.05 1.54 1.01 0.47
Me
Me
O
S
N
Me
CN
O
N
O
f1 (ppm)

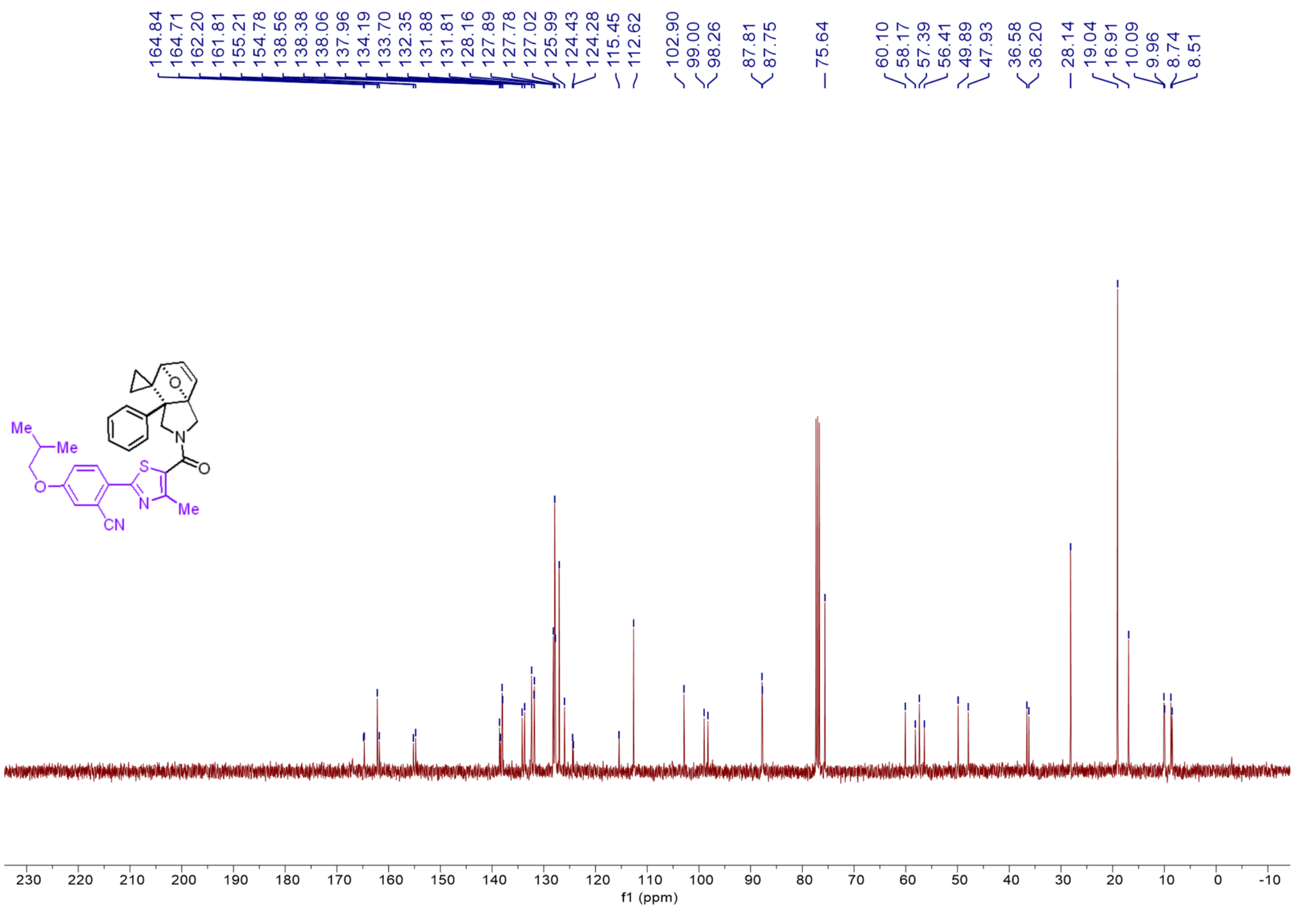

164.84 164.71 162.20 161.81 155.21 154.78 138.56 138.38 138.06 137.96 134.19 133.70 132.35 131.88 131.81 128.16 127.89 127.78 127.02 125.99 124.43 124.28 115.45 112.62 102.90 99.00 98.26 87.81 87.75 75.64 60.10 58.17 57.39 56.41 49.89 47.93 36.58 36.20 28.14 19.04 16.91 10.09 9.96 8.74 8.51
Me
Me
O
S
N
Me
CN
O
N
O
f1 (ppm)

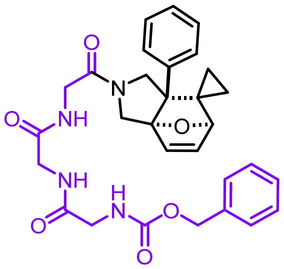

Compound 4f: Yield: 43.0 mg, 79%; A yellow oil; ^{1}H NMR (400 MHz, $CDCl_3$) (mixture of diastereomers) δ 7.66 -7.42 (m, 2H), 7.32 - 7.15 (m, 8H), 7.10 - 7.03 (m, 2H), 6.73 (dt, J = 6.0, 1.8 Hz, 1H), 6.13 (s, 1H), 6.06 (t, J = 6.0 Hz, 1H), 5.07 (s, 2H), 4.44 - 4.18 (m, 2H), 4.13 - 3.88 (m, 7H), 3.88 - 3.59 (m, 2H), 0.94 - 0.79 (m, 1H), 0.78 - 0.59 (m, 2H), 0.58 - 0.43 (m, 1H); ^{13}C NMR (100 MHz, $CDCl_3$) δ 170.0, 169.3, 169.2, 166.8, 166.7, 156.8, 138.5, 138.4, 138.0, 137.9, 136.3, 134.0, 133.7, 128.5, 128.1, 128.0, 127.93, 127.87, 127.8, 127.0, 126.9, 99.2, 97.7, 87.7, 87.6, 67.0, 58.2, 57.1, 56.9, 56.1, 47.5, 47.0, 44.4, 42.78, 42.76, 42.1, 42.0, 36.4, 36.3, 10.1, 10.0, 8.8, 8.7; IR (neat): ν 3312, 3065, 2929, 1641, 1525, 1452, 1251, 1051, 894, 842, 742, 699 cm^{-1}; HRMS (ESI) Calcd. for $C_{30}H_{33}N_4O_6$ $[M+H]^+$: 545.2395, Found: 545.2393.

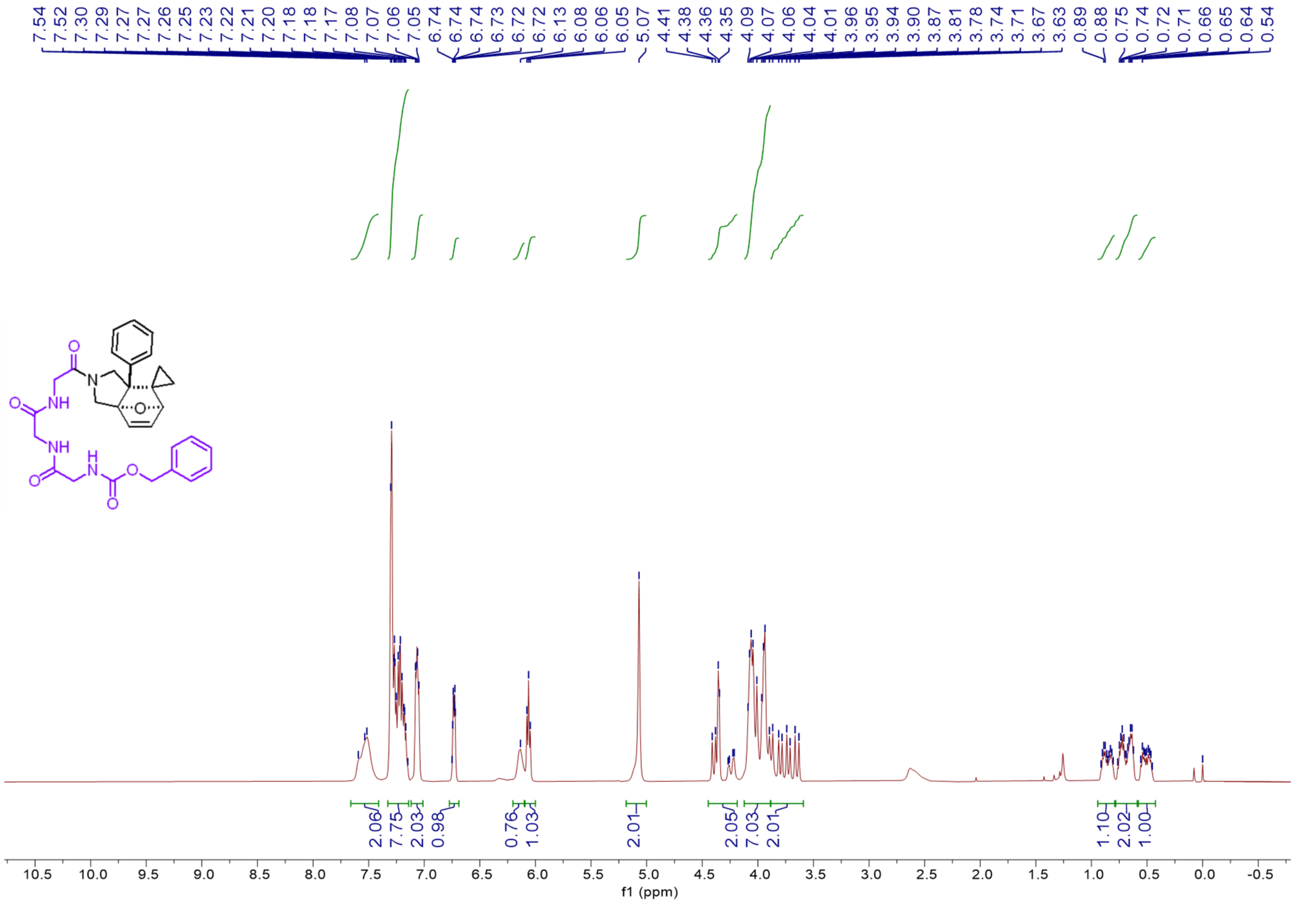
7.54 7.52 7.30 7.29 7.27 7.27 7.26 7.25 7.23 7.22 7.21 7.20 7.18 7.18 7.17 7.08 7.07 7.06 7.05 6.74 6.74 6.73 6.72 6.72 6.13 6.08 6.06 6.05 5.07 4.41 4.38 4.36 4.35 4.09 4.07 4.06 4.04 4.01 3.96 3.95 3.94 3.90 3.87 3.81 3.78 3.74 3.71 3.67 3.63 0.89 0.88 0.75 0.74 0.72 0.71 0.66 0.65 0.64 0.54
O
N
NH
O
NH
H
N
O
O
O
2.06 7.75 2.03 0.98 0.76 1.03 2.01 2.05 7.03 2.01 1.10 2.02 1.00
10.5 10.0 9.5 9.0 8.5 8.0 7.5 7.0 6.5 6.0 5.5 5.0 4.5 4.0 3.5 3.0 2.5 2.0 1.5 1.0 0.5 0.0 -0.5
f1 (ppm)

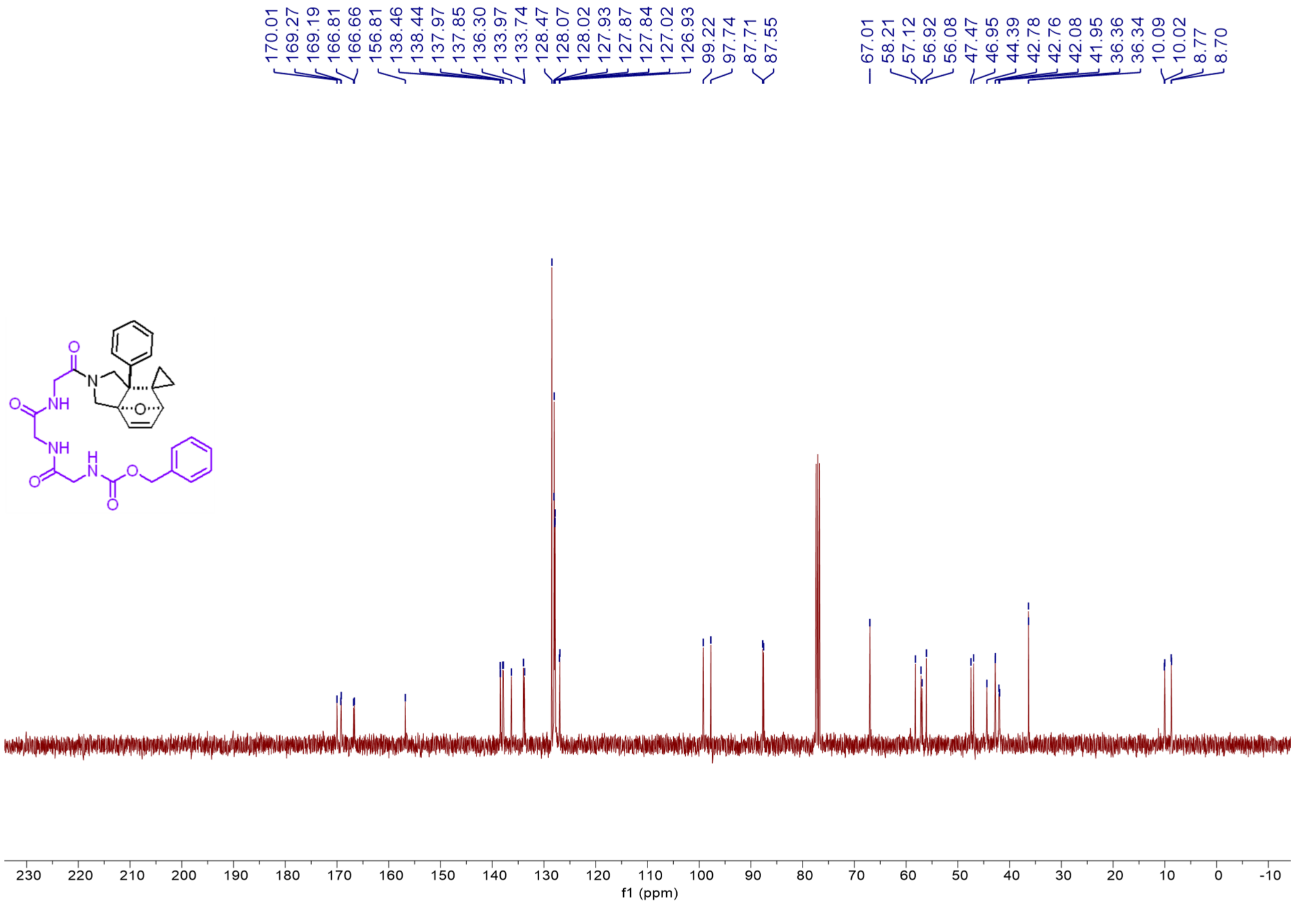
170.01 169.27 169.19 166.81 166.66 156.81 138.46 138.44 137.97 137.85 136.30 133.97 133.74 128.47 128.07 128.02 127.93 127.87 127.84 127.02 126.93 99.22 97.74 87.71 87.55
67.01 58.21 57.12 56.92 56.08 47.47 46.95 44.39 42.78 42.76 42.08 41.95 36.36 36.34 10.09 10.02 8.77 8.70
O
N
NH
O
NH
H
N
O
O
O
230 220 210 200 190 180 170 160 150 140 130 120 110 100 90 80 70 60 50 40 30 20 10 0 -10
f1 (ppm)

(5) X-ray Crystal Data of 2a.

Single crystals of **2a** were grown in dichloromethane and hexanes. Hexanes (2.0 mL) were added to **2a** (20.0 mg in a 4.0 mL vial) followed by five drops of dichloromethane. The 4.0 mL vial was capped and placed at room temperature in the experimental cabinet for seven days or longer if necessary, whereupon crystals were formed.

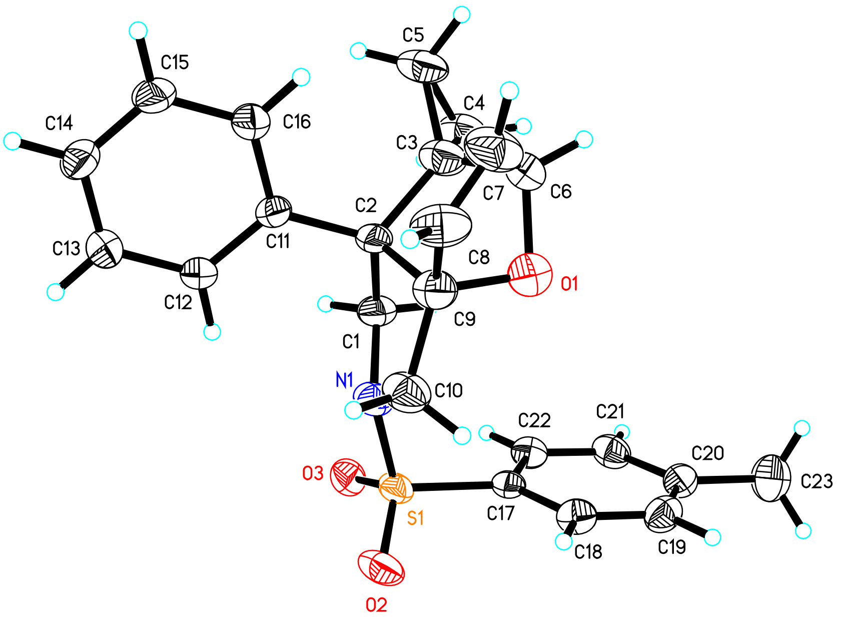

The crystal data of **2a** have been deposited in CCDC with number 2282257. Empirical formula: $C_{23}H_{23}NO_3S$; Formula weight: 393.48; Crystal Color, Habit: colorless; Crystal dimensions: 0.170 x 0.140 x 0.120 mm^3; Crystal system: Monoclinic; Lattice parameters: a = 9.9390(15) Å, b = 16.582(3) Å, c = 12.5722(19) Å, α = 90°, β = 106.558(4)°, γ = 90°, V = 1986.1(5) Å^3; Space group: P 21/c; Z = 4; D_{calc} = 1.316 g/cm^3; F_{000} = 832; Final R indices [I>2sigma(I)] R1 = 0.1070, wR2 = 0.2411. The thermal ellipsoids were set at a 30% probability level.

(6) Computational details

Table S2. The total energies, enthalpies, and free energies of all species.[a]

sub	E_{tot}	H_{298}	G_{298}
**1a**	-1568.909642	-1568.267209	-1568.55414
**Ts$_a$**	-1568.872283	-1568.231302	-1568.512384
**2a**	-1568.927595	-1568.284082	-1568.564525
**1ac**	-1570.166505	-1569.501749	-1569.787456
**Ts$_{ac}$**	-1570.113521	-1569.449459	-1569.730817
**2ac**	-1570.168067	-1569.502416	-1569.782388
**1ad**	-1491.529943	-1490.931303	-1491.204493
**Ts$_{ad}$**	-1491.488206	-1490.891506	-1491.160119
**2ad**	-1491.536018	-1490.936736	-1491.203695
**1ae**	-1608.042699	-1607.572375	-1607.863031
**Ts$_{ae}$**	-1608.201399	-1607.526239	-1607.811851
**2ae**	-1608.243604	-1607.566021	-1607.851165

[a] Calculated at t ωB97X-D/6-311G(d,p), SMD ($CHCl_3$) level, supplemented by single-point energy corrections at the ωB97X-D/def2-TZVPP, SMD ($CHCl_3$) level.

1a

opt=recalc=4 freq wb97xd/6-311g(d,p) scrf=(smd,solvent=chloroform)

0 1

C	1.95227400	-1.71253200	-0.40859100
C	0.52508700	-1.31562400	-0.52192900
C	-0.45904800	-1.73419100	0.56086400
C	0.08037800	-0.62769000	-1.56196300
N	-1.27154300	-0.64184700	1.12082300
C	0.35812000	0.10896900	-2.79503500
C	-1.05489500	0.02812400	-2.20438300
C	-2.69137500	-0.95082000	1.35836100
C	-3.46904900	-0.94756000	0.09216200
C	-3.99668300	-1.93666800	-0.67158800
C	-4.64596900	-1.29330300	-1.77435900
C	-4.45845200	0.03433700	-1.59001200
O	-3.75048900	0.26162400	-0.45825500
C	2.70911700	-1.97220600	-1.55779100
C	4.05287000	-2.30796500	-1.46996000
C	4.67346400	-2.39901600	-0.22839500
C	3.93288000	-2.15434800	0.92152400
C	2.58660700	-1.81943700	0.83433800
C	0.31987100	1.54635600	1.07613000
C	1.70329400	1.57292700	1.02356200
C	2.32667000	2.45104000	0.14481200
C	1.58294600	3.30052800	-0.67079400
C	0.18627400	3.26059100	-0.58416500
C	-0.45054300	2.39060200	0.28230900
C	2.25260700	4.24971900	-1.62537000

S	-0.51121900	0.40927800	2.15959800
O	-1.55217300	1.13459200	2.87067000
O	0.50832600	-0.29283100	2.92797400
H	-1.17552900	-2.42394400	0.11087700
H	0.04395000	-2.28099300	1.36180300
H	0.55940600	-0.44351900	-3.70789700
H	0.88742700	1.05352900	-2.70563400
H	-1.46360600	0.91763400	-1.73854500
H	-1.78747100	-0.59061000	-2.71551500
H	-2.79226400	-1.92997700	1.83646600
H	-3.09628300	-0.20294200	2.03882500
H	-3.93323600	-2.99520100	-0.47034000
H	-5.17833300	-1.75896100	-2.58899000
H	-4.75628100	0.90930800	-2.14494800
H	2.23358700	-1.92096800	-2.52997100
H	4.61574900	-2.50808900	-2.37539900
H	5.72273800	-2.66379700	-0.15855300
H	4.40340800	-2.22120900	1.89655500
H	2.03515700	-1.61343400	1.74442500
H	2.28708500	0.91162500	1.65042600
H	3.40981800	2.46706400	0.09244900
H	-0.40739800	3.91804400	-1.21093200
H	-1.53263700	2.35855800	0.33559200
H	1.84446900	4.14054100	-2.63404700
H	2.08728600	5.28665500	-1.31727500
H	3.32928200	4.07707700	-1.67018300

Ts_a

opt=(calcfc,ts,noeigen) freq ωb97xd/6-311g(d,p) scrf=(smd,solvent=chloroform)

#p ωb97xd/6-311g(d,p) opt=(ts,calcfc,noeigen,nofreeze,maxcyc=256) geom=check guess=read scrf=(smd,solvent=chloroform)

#p ωb97xd/6-311g(d,p) freq geom=check guess=read scrf=(smd,solvent=chloroform)

0 1

S	1.45614500	-1.93385300	0.16179700
O	-0.40513000	2.06658700	1.23655800
O	1.60240500	-2.67324500	1.40329800
O	1.20570600	-2.63500000	-1.08456200
N	0.19562500	-0.88441900	0.38726400
C	-0.19739200	-0.04282700	-0.76243700
H	-0.14894700	-0.67724300	-1.65060800
H	0.50709300	0.78528000	-0.89665300
C	-1.58530600	0.51668300	-0.57923200
C	-1.78588500	1.88103100	-0.79633300
C	-1.19551800	2.80714800	-1.80091200
C	-2.70534700	2.65644400	-1.65815700
C	-1.54567400	2.78187700	0.97286500
H	-1.38362200	3.82245600	0.72402200
C	-2.58194400	2.25731600	1.77620900
H	-3.56277500	2.69027400	1.89802700
C	-2.14851500	1.00875300	2.17632700
H	-2.71220200	0.23060100	2.66740900
C	-0.85951700	0.86649900	1.66382000
C	0.13735900	-0.22852800	1.71529400
H	-0.14487500	-0.96898100	2.46116700
H	1.10882600	0.20258400	1.98812600
C	-2.73424700	-0.40834900	-0.52746600
C	-2.57487300	-1.79391400	-0.68849800
H	-1.59052400	-2.21413600	-0.84898200

C	-3.66432900	-2.65297200	-0.64002100
H	-3.50630800	-3.71802800	-0.77281500
C	-4.94602500	-2.16057900	-0.42014000
H	-5.79472900	-2.83436100	-0.37938300
C	-5.12352600	-0.79125200	-0.25867400
H	-6.11682800	-0.38796200	-0.09101600
C	-4.03726500	0.07135400	-0.31669100
H	-4.20181600	1.13386600	-0.19500700
C	2.90764500	-0.92739000	-0.05628800
C	3.68351400	-0.59064700	1.04625700
H	3.45451000	-1.00258800	2.02154400
C	4.76779500	0.26022600	0.87550700
H	5.37886900	0.51975200	1.73331800
C	5.08387600	0.78170400	-0.37856500
C	4.28770200	0.42524100	-1.47095300
H	4.52438400	0.81601500	-2.45512900
C	3.20300600	-0.42313900	-1.32018000
H	2.60080300	-0.70409300	-2.17570400
C	6.26862500	1.68789300	-0.56701500
H	7.07729200	1.15794400	-1.08004700
H	6.65392600	2.04428500	0.38988400
H	6.00614800	2.55487800	-1.17860200
H	-3.24758700	3.49131300	-1.22440200
H	-3.24564500	2.12411500	-2.43456400
H	-0.74620200	3.74073900	-1.47762200
H	-0.72628000	2.36372900	-2.67322400

2a

#opt freq wb97xd/6-311g(d,p) scrf=(smd,solvent=chloroform)

0 1

S	-1.44126000	-2.19571400	0.12008500
O	-0.22422600	1.57333200	-0.86879500
O	-1.59647100	-3.06820400	-1.02971100
O	-1.33611800	-2.74532300	1.45978100
N	-0.06610300	-1.32435100	-0.13192500
C	0.38856600	-0.42047300	0.94502400
H	0.76591400	-0.99782800	1.78795800
H	-0.42813400	0.21887700	1.28821300
C	1.45695700	0.45295200	0.26965200
C	1.38512100	1.92635300	0.72026400
C	1.00956700	2.33170900	2.11726600
H	0.28642300	3.13154600	2.23497400
H	0.95409700	1.55444500	2.87153600
C	2.37356300	2.66123100	1.57008500
H	2.59000400	3.69208200	1.30865900
H	3.21620200	2.10980900	1.96956000
C	0.68266200	2.60520600	-0.47821200
H	0.15808100	3.52929100	-0.24538800
C	1.64993300	2.61684500	-1.65080800
H	2.24176200	3.46419800	-1.96871100
C	1.69885700	1.36033800	-2.08652500
H	2.34072600	0.92372000	-2.83822000
C	0.77696300	0.59810800	-1.16307100
C	0.17072400	-0.74201600	-1.47051800
H	0.85972200	-1.37327500	-2.03435400
H	-0.74920900	-0.61707600	-2.04492800
C	2.82914000	-0.20301700	0.21147600
C	2.98397200	-1.57800800	0.41489600

H	2.12492000	-2.19842900	0.63836300
C	4.23017300	-2.18616900	0.31201100
H	4.31521800	-3.25502400	0.47489500
C	5.35557800	-1.43508200	0.00117000
H	6.32793000	-1.90867400	-0.07706300
C	5.21888800	-0.06852000	-0.21082200
H	6.08657600	0.53413400	-0.45681100
C	3.97304500	0.53692700	-0.10795800
H	3.89336900	1.60120900	-0.28227500
C	-2.78009100	-1.02480100	0.10982600
C	-3.40293000	-0.70533200	-1.09386200
H	-3.14025300	-1.23402200	-2.00204300
C	-4.37859600	0.27746900	-1.10827100
H	-4.87083300	0.52464100	-2.04315700
C	-4.74160200	0.95281700	0.06025500
C	-4.10926800	0.60725600	1.25386800
H	-4.38754600	1.11139500	2.17312000
C	-3.12953500	-0.37608200	1.28809400
H	-2.65527000	-0.64792500	2.22315700
C	-5.79754200	2.02236100	0.01857700
H	-5.97825100	2.44352800	1.00907000
H	-6.74188200	1.62112600	-0.36026300
H	-5.49704700	2.83527900	-0.64888900

1ac

p opt freq wb97xd/6-311g(d,p) scrf=(smd,solvent=chloroform)

0 1

C	2.03324400	-1.70339200	-0.37135800

C	0.65996500	-1.17376300	-0.61174200
C	-0.42070000	-1.65645500	0.34462900
C	0.36166000	-0.38423700	-1.65939400
N	-1.27623300	-0.61161700	0.95110200
C	1.39199600	0.28722700	-2.52666600
C	-1.05276200	-0.04064400	-2.03889400
C	-2.63033300	-1.08124600	1.30836200
C	-3.51230200	-1.12826000	0.11455900
C	-4.01914600	-2.14638700	-0.62512800
C	-4.77034500	-1.54054900	-1.68340800
C	-4.65824900	-0.20435400	-1.49828300
O	-3.90499900	0.06225600	-0.40519500
C	2.75904600	-2.26906400	-1.42701100
C	4.03384500	-2.78459700	-1.23116300
C	4.61175700	-2.75831700	0.03291800
C	3.89531700	-2.22308800	1.09706800
C	2.61920700	-1.70910500	0.89958900
C	0.22741900	1.65735200	1.00023700
C	1.61141200	1.74299200	0.95394700
C	2.19803100	2.71117700	0.15283800
C	1.42024800	3.59486400	-0.59689000
C	0.02956200	3.49560200	-0.51606300
C	-0.57376600	2.53335900	0.27900100
C	2.07028500	4.60946900	-1.49567700
S	-0.55732200	0.44053600	2.03030100
O	-1.63309100	1.09900900	2.75519100
O	0.48249400	-0.23617100	2.79522100
H	-1.11446000	-2.29852400	-0.20510500
H	0.02130200	-2.27351200	1.13239900
H	1.40581100	-0.14381700	-3.53441000

H	2.40031800	0.23891100	-2.11698700
H	-1.38774900	0.87638700	-1.54692500
H	-1.75955400	-0.83016600	-1.78243800
H	-2.58217900	-2.07798800	1.75826300
H	-3.05023100	-0.39857400	2.04544000
H	-3.86848800	-3.19924800	-0.44135900
H	-5.31650400	-2.03526100	-2.47132700
H	-5.04617200	0.65220800	-2.02549100
H	2.31036700	-2.31661100	-2.41302400
H	4.57184900	-3.21788200	-2.06753000
H	5.60607100	-3.16213100	0.18908000
H	4.32920200	-2.20603200	2.09115700
H	2.07255300	-1.30496100	1.74346400
H	2.22125100	1.06232000	1.53340800
H	3.27983500	2.77921800	0.10949100
H	-0.59067600	4.18180200	-1.08280200
H	-1.65383900	2.46506200	0.33669300
H	2.36851600	4.14298900	-2.44060500
H	1.38939900	5.43025900	-1.72919100
H	2.97066400	5.02509200	-1.03745200
H	-1.11109400	0.12916800	-3.11825100
H	1.11701500	1.34176300	-2.63957600

Ts_{ac}

opt=(calcfc,ts,noeigen) freq ωb97xd/6-311g(d,p) scrf=(smd,solvent=chloroform)

#p ωb97xd/6-311g(d,p) opt=(ts,calcfc,recalc=4,noeigen,nofreeze,maxcyc=256) geom=check guess=read scrf=(smd,solvent=chloroform)

#p ωb97xd/6-311g(d,p) freq geom=check guess=read scrf=(smd,solvent=chloroform)

0 1

S	1.36366600	-1.95624700	0.21020900
O	-0.22675900	2.15746400	0.91323300
O	1.45815400	-2.66451800	1.47424500
O	1.13315400	-2.68120000	-1.02637300
N	0.12588100	-0.87600400	0.37036400
C	-0.22555100	-0.05665600	-0.80065500
H	-0.21379200	-0.69663900	-1.68297000
H	0.52805500	0.71673400	-0.95185600
C	-1.58742900	0.56415700	-0.55271800
C	-1.81799700	1.89257000	-1.02519700
C	-0.79119700	2.51300100	-1.97094300
C	-3.21269900	2.36060500	-1.39925600
C	-1.28356300	2.98952600	0.65012000
H	-1.01338100	3.96331400	0.26324800
C	-2.29606000	2.70470200	1.59597300
H	-3.18731800	3.29221300	1.75752100
C	-2.00318400	1.45769800	2.09426100
H	-2.60449600	0.83337400	2.73655400
C	-0.82273500	1.05089200	1.44211800
C	0.05332200	-0.13364700	1.64927700
H	-0.35943100	-0.78183500	2.42042000
H	1.04394000	0.21313400	1.96765600
C	-2.70011200	-0.43933400	-0.41028800
C	-2.57728900	-1.71052100	-0.99210500
H	-1.67756700	-1.98752600	-1.52510300
C	-3.58656100	-2.66035900	-0.89938400
H	-3.44508800	-3.62934600	-1.36595900
C	-4.75974900	-2.37986900	-0.21200000
H	-5.54731200	-3.12134100	-0.13613000

C	-4.90037700	-1.13344600	0.38388000
H	-5.80270300	-0.89139000	0.93563200
C	-3.89004800	-0.18523600	0.28563500
H	-4.03455500	0.76215900	0.78194600
C	2.84507400	-0.98969900	0.00925200
C	3.60724600	-0.65719700	1.12343300
H	3.34817000	-1.05170400	2.09842600
C	4.71273500	0.16684700	0.96493000
H	5.31204300	0.42401000	1.83188000
C	5.06537400	0.66715000	-0.28899700
C	4.28288800	0.31705900	-1.39217200
H	4.54618900	0.69223200	-2.37554800
C	3.17625000	-0.50620700	-1.25319200
H	2.58429100	-0.78233200	-2.11749600
C	6.28124500	1.53521800	-0.45729900
H	6.13927700	2.26616000	-1.25634600
H	7.15077600	0.92377900	-0.71988500
H	6.51825200	2.06999700	0.46464500
H	-3.98166100	2.09306400	-0.68123500
H	-3.50328500	1.93900000	-2.36935800
H	0.21750300	2.55030700	-1.55857800
H	-0.74884100	1.95011800	-2.91098100
H	-3.22171700	3.44953400	-1.50104500
H	-1.08104600	3.53749200	-2.21820900

2ac

#p opt freq wb97xd/6-311g(d,p) scrf=(smd,solvent=chloroform)

0 1

S	1.39093100	-2.09663400	0.06766600
O	0.03407300	1.93158700	0.88295600
O	1.53740200	-2.87323900	1.28555600
O	1.17786100	-2.75217700	-1.20948400
N	0.10593300	-1.09411100	0.30432500
C	-0.32678700	-0.22978600	-0.80868700
H	-0.64413000	-0.84460800	-1.64842000
H	0.48442200	0.43092900	-1.12071100
C	-1.48351500	0.56635800	-0.16524900
C	-1.68276700	2.05173100	-0.68335500
C	-0.91406800	2.36723700	-1.97037100
H	0.16744000	2.33954800	-1.82401000
H	-1.17204700	1.66699300	-2.77072800
C	-3.13892200	2.48542500	-0.86801700
H	-3.78316300	2.15162900	-0.05313100
H	-3.55561100	2.10362500	-1.80254500
C	-1.01578600	2.82268800	0.51436900
H	-0.62330000	3.80245600	0.24682600
C	-1.91831000	2.74647100	1.73227600
H	-2.58414100	3.52546900	2.07642800
C	-1.78585800	1.50805500	2.19549600
H	-2.30578700	1.03476000	3.01571800
C	-0.81616500	0.83718800	1.24248900
C	0.00160300	-0.38193100	1.59345200
H	-0.48658300	-1.01617800	2.33327000
H	0.97291500	-0.05979600	1.98096200
C	-2.72178400	-0.31535100	-0.19659100
C	-3.32799100	-0.56274300	-1.43319700
H	-2.91978900	-0.10339900	-2.32778500
C	-4.43641100	-1.38831500	-1.54294600

H	-4.88729800	-1.55721200	-2.51476200
C	-4.96275600	-2.00306300	-0.41092300
H	-5.82760100	-2.65209200	-0.49146800
C	-4.35913500	-1.78789200	0.81904300
H	-4.74499300	-2.27511200	1.70783600
C	-3.24879000	-0.95404900	0.92309100
H	-2.79322100	-0.82827100	1.89623200
C	2.81791200	-1.04506400	-0.08970600
C	3.55205800	-0.70475700	1.04298100
H	3.30861000	-1.14382900	2.00279400
C	4.61251700	0.17879400	0.91954800
H	5.19264700	0.43951800	1.79865900
C	4.94980800	0.73439900	-0.31737500
C	4.20133400	0.37283400	-1.43750900
H	4.45718300	0.78369600	-2.40827800
C	3.13634500	-0.51144700	-1.33317500
H	2.57170500	-0.79863300	-2.21185900
C	6.09504200	1.70234700	-0.42611900
H	6.31889000	1.94046900	-1.46745300
H	6.99765800	1.29262300	0.03501000
H	5.85823800	2.63646100	0.09250400
H	-1.17491100	3.37265300	-2.31474300
H	-3.18369600	3.57907700	-0.91262800

1ad

#p opt freq ωb97xd/6-311g(d,p) scrf=(smd,solvent=chloroform)

0 1

C	0.10481600	1.72727800	-1.17731300

C	-1.33818800	1.40552600	-1.34873100
C	-1.70281300	0.03393800	-1.87387700
C	-2.27420000	2.31167400	-1.07528100
N	-1.46574900	-1.05639800	-0.92131500
C	-2.54254100	-1.40537500	0.00760500
C	-2.90469700	-0.29248800	0.92758100
C	-4.07593200	0.34513700	1.16995300
C	-3.79023800	1.34912900	2.15069800
C	-2.46906400	1.23647900	2.42219400
O	-1.92044100	0.23314500	1.69541400
C	0.57936200	2.22906000	0.03599600
C	1.90669300	2.61589700	0.17252300
C	2.78020500	2.50527300	-0.90310700
C	2.32284900	1.98752600	-2.10969200
C	0.99832300	1.59147400	-2.24300300
C	1.09000100	-1.45214100	0.05085000
C	2.32552200	-1.06902500	-0.44748800
C	3.30894800	-0.65013700	0.43781300
C	3.07037700	-0.60013800	1.80982600
C	1.81675700	-0.99494900	2.28390900
C	0.82780300	-1.42767200	1.41633200
C	4.12689700	-0.09568500	2.75323600
S	-0.15892300	-2.05181600	-1.06860500
O	-0.56294800	-3.36339400	-0.58661000
O	0.35526100	-1.90582600	-2.41953700
H	-2.76499800	0.00834000	-2.13184300
H	-1.14514500	-0.18670500	-2.78457300
H	-3.43928700	-1.68192400	-0.55671500
H	-2.23687300	-2.28695600	0.57177000
H	-5.02209400	0.13919900	0.69336100

H	-4.47590700	2.05895300	2.58660400
H	-1.79977400	1.75961800	3.08586600
H	-0.10287900	2.31155000	0.87458000
H	2.25953000	3.00219600	1.12256500
H	3.81559700	2.81056600	-0.79855000
H	2.99930000	1.89158200	-2.95205600
H	0.65196300	1.19599500	-3.19179300
H	2.51277600	-1.09501600	-1.51293400
H	4.27609100	-0.34808800	0.05031000
H	1.61415600	-0.96925300	3.34949000
H	-0.13506900	-1.73342100	1.80536600
H	4.07477800	0.99549000	2.83248100
H	3.99617900	-0.50729700	3.75624400
H	5.12860100	-0.35237100	2.40138400
H	-2.01543600	3.30881200	-0.73429200
H	-3.32876300	2.08647900	-1.19465600

Ts$_{ad}$

opt=(calcfc,ts,noeigen) freq ωb97xd/6-311g(d,p) scrf=(smd,solvent=chloroform)

#p ωb97xd/6-311g(d,p) opt=(ts, recalc=4,calcfc,noeigen,nofreeze,maxcyc=256) geom=check guess=read scrf=(smd,solvent=chloroform)

#p ωb97xd/6-311g(d,p) freq geom=check guess=read scrf=(smd,solvent=chloroform)

0 1

S	1.21082300	-1.79032700	0.43362200
O	-0.34388300	2.40552400	0.45273600
O	1.26413100	-2.25188200	1.80951700
O	0.99334600	-2.73156200	-0.65007400
N	-0.01005900	-0.67900000	0.35995000

C	-0.33519300	-0.10767500	-0.96184400
H	-0.36034200	-0.93637700	-1.67250900
H	0.44377700	0.59272400	-1.28415600
C	-1.66044800	0.61515100	-0.91796100
C	-1.74020000	1.88628400	-1.51289100
C	-1.37696200	3.17054000	-0.02703800
H	-1.07455600	4.08982700	-0.50966300
C	-2.47760200	3.00082700	0.85000900
H	-3.38863000	3.57944700	0.83164300
C	-2.22162800	1.84545100	1.55284800
H	-2.88654900	1.29780000	2.20289500
C	-0.97038100	1.39582800	1.11162400
C	-0.12027500	0.24852700	1.50988900
H	-0.56415200	-0.27937000	2.35229300
H	0.86355800	0.62989400	1.80849900
C	-2.89086700	-0.17895300	-0.67530800
C	-2.84539900	-1.56886800	-0.49282800
H	-1.89849900	-2.09163900	-0.52436000
C	-4.00126300	-2.30332400	-0.26026700
H	-3.92884200	-3.37750900	-0.12714800
C	-5.23875000	-1.67463100	-0.19455900
H	-6.13956600	-2.24914600	-0.00940900
C	-5.30397700	-0.29740500	-0.37327100
H	-6.26076000	0.21225100	-0.32867000
C	-4.15064800	0.43651100	-0.61370200
H	-4.23815000	1.50772100	-0.74716200
C	2.71358400	-0.90022000	0.09246300
C	3.43817700	-0.34826600	1.14198900
H	3.13602600	-0.52583600	2.16696600
C	4.56584200	0.41111500	0.85937400

H	5.13669400	0.83813300	1.67690800
C	4.97676200	0.62920300	-0.45539000
C	4.23091600	0.06081300	-1.49195400
H	4.54162000	0.21398900	-2.52021900
C	3.10326300	-0.70029700	-1.22929100
H	2.54025600	-1.14886000	-2.03883800
C	6.21041600	1.43288400	-0.75958700
H	6.03333400	2.12802900	-1.58408400
H	7.03168000	0.77346100	-1.05768300
H	6.53809700	2.00468500	0.11052200
H	-0.88365400	2.21805100	-2.09182800
H	-2.68506100	2.22334500	-1.92288100

2ad

#p opt freq ωb97xd/6-311g(d,p) scrf=(smd,solvent=chloroform)

0 1

S	1.22969300	-2.08701600	0.18512800
O	0.18037500	1.82580200	0.40738100
O	1.32107100	-2.72002100	1.48830400
O	1.13612700	-2.88625300	-1.02334300
N	-0.11475400	-1.13591200	0.21697000
C	-0.50547200	-0.44232300	-1.02919700
H	-0.91340400	-1.15526200	-1.74483800
H	0.35627500	0.05938900	-1.47718300
C	-1.52704000	0.61185900	-0.57143900
C	-1.35783300	1.96541200	-1.30682600
C	-0.64496300	2.81487300	-0.20851200
H	-0.04982100	3.64851200	-0.57202800

C	-1.65202500	3.09842500	0.89236200
H	-2.20303800	4.02087800	1.01585600
C	-1.79421800	1.95244500	1.55438600
H	-2.48955100	1.70245300	2.34293400
C	-0.88673400	0.98188500	0.83659400
C	-0.37222500	-0.30612400	1.41340900
H	-1.11950500	-0.78823400	2.04645900
H	0.52914700	-0.12617400	2.00192800
C	-2.94259900	0.06086300	-0.45462500
C	-3.16626300	-1.31014300	-0.28631700
H	-2.33025700	-1.99892100	-0.26263600
C	-4.45053700	-1.81797400	-0.12910600
H	-4.58837200	-2.88615400	-0.00173100
C	-5.54825500	-0.96752300	-0.13432400
H	-6.55081600	-1.36303600	-0.01494500
C	-5.34432500	0.39731600	-0.29359000
H	-6.18913400	1.07760900	-0.29813800
C	-4.05926000	0.90255900	-0.44971800
H	-3.93794000	1.97261300	-0.55983800
C	2.61452600	-0.98519800	0.00726700
C	3.21138200	-0.44531500	1.14255800
H	2.89641000	-0.76231800	2.12927700
C	4.23178100	0.48028400	0.99347300
H	4.70485400	0.89655300	1.87660900
C	4.66404200	0.88088000	-0.27300900
C	4.05675000	0.31575400	-1.39470200
H	4.39198200	0.60124800	-2.38614900
C	3.03356100	-0.61287200	-1.26494800
H	2.58045700	-1.05938800	-2.14157300
C	5.74949100	1.91097800	-0.41745000

H	6.20629200	1.87263500	-1.40821600
H	6.53233200	1.76796100	0.33104800
H	5.33961100	2.91639200	-0.27623700
H	-2.29125100	2.41238700	-1.64270200
H	-0.70537800	1.86192300	-2.17519000

1ae

#p opt freq ωb97xd/6-311g(d,p) scrf=(smd,solvent=chloroform)

1

C	-0.42366200	1.65097500	-0.70157800
C	-1.58090200	0.74113100	-0.92051400
C	-1.38149800	-0.48457700	-1.77824000
C	-2.77744500	1.04772200	-0.42292700
N	-0.59509200	-1.55117400	-1.14295600
C	-4.16773200	0.46203400	-0.58113200
C	-1.26755100	-2.46036600	-0.21953500
C	-1.70913800	-1.83082400	1.05892500
C	-2.88376400	-1.87335400	1.73517100
C	-2.68851700	-1.10267100	2.92729300
C	-1.41074000	-0.65940800	2.87641500
O	-0.80063100	-1.10626800	1.75186700
C	-0.03895000	2.03268500	0.58502500
C	0.98293800	2.95403400	0.77927400
C	1.63962400	3.50998000	-0.31233500
C	1.27725000	3.12583400	-1.59866900
C	0.26034600	2.19924800	-1.79069400
C	2.03034900	-1.04392300	-0.42344500
C	2.76633200	0.06348500	-0.81643500

C	3.60069000	0.67992600	0.10484200
C	3.69882700	0.21305900	1.41463300
C	2.95400800	-0.91079100	1.77986800
C	2.12602500	-1.54617400	0.86921500
C	4.56195300	0.92468800	2.41923600
S	0.96227100	-1.85239200	-1.59580600
O	1.18400900	-3.28256200	-1.44838000
O	1.16698700	-1.21710600	-2.88631600
H	-2.34963400	-0.92019600	-2.03974000
H	-0.88448600	-0.22499000	-2.71301900
H	-4.35549700	-0.34642700	0.13048000
H	-4.45706100	0.12718200	-1.57991400
H	-2.15208100	-2.88095400	-0.70912700
H	-0.59552600	-3.29531800	-0.01560700
H	-3.78102700	-2.38354200	1.42051600
H	-3.40523100	-0.90554500	3.70931000
H	-0.80608500	-0.05437000	3.53240600
H	-0.55051300	1.60048900	1.43683500
H	1.26756600	3.23691100	1.78712000
H	2.43430300	4.23259900	-0.16193900
H	1.78610100	3.55059600	-2.45733500
H	-0.01846400	1.91431200	-2.79991600
H	2.68879900	0.43587200	-1.82914200
H	4.17796800	1.54588900	-0.20041800
H	3.02785000	-1.29780400	2.79076800
H	1.56408300	-2.42239300	1.16591800
H	3.99541300	1.72782200	2.90276400
H	4.90735900	0.24545000	3.20153900
H	5.43444900	1.37788000	1.94354600
C	-4.73622500	1.82288600	-0.09496800

H	-5.10343000	2.43457600	-0.92078500
H	-5.49715300	1.77963000	0.68523600
C	-3.30833600	2.23729200	0.35071200
H	-2.94458100	3.22186000	0.04698700
H	-3.16002500	2.12708800	1.42891400

Ts$_{ae}$

opt=(calcfc,ts,noeigen) freq ωb97xd/6-311g(d,p) scrf=(smd,solvent=chloroform)

#p ωb97xd/6-311g(d,p) opt=(ts,recalc=4calcfc,noeigen,nofreeze,maxcyc=256) geom=check guess=read scrf=(smd,solvent=chloroform)

#p ωb97xd/6-311g(d,p) freq geom=check guess=read scrf=(smd,solvent=chloroform)

0 1

S	1.58439100	-2.05582000	-0.10687600
O	-0.23857200	1.71684700	1.40037100
O	1.76537000	-2.96470500	1.01106600
O	1.37162300	-2.57117200	-1.44761500
N	0.27503300	-1.11729900	0.25940900
C	-0.17503000	-0.14974000	-0.75848800
H	-0.18816800	-0.66580700	-1.72091800
H	0.53113600	0.68300700	-0.83252100
C	-1.54182100	0.38314100	-0.39801900
C	-1.75063300	1.76972700	-0.57550200
C	-0.85057400	2.68820300	-1.42474500
H	0.03161400	3.12140600	-0.94725600
H	-0.53203400	2.19323300	-2.34716000
C	-2.99446900	2.51830800	-1.07838300
H	-3.72123500	2.84489500	-0.33228700
H	-3.52480300	1.95532300	-1.85208200

C	-1.34359000	2.51897400	1.26868600
H	-1.12755900	3.56701100	1.10730500
C	-2.35951500	1.98836700	2.10006000
H	-3.29464300	2.47293000	2.33628700
C	-1.99478400	0.68651000	2.35287700
H	-2.57852900	-0.09448200	2.81530500
C	-0.76433500	0.50039300	1.70358400
C	0.17474600	-0.64738300	1.66069400
H	-0.18862600	-1.46087800	2.28648700
H	1.14842300	-0.31540300	2.03977600
C	-2.67643000	-0.57222100	-0.40022100
C	-2.51168200	-1.90204300	-0.81815300
H	-1.54051000	-2.25878900	-1.13516900
C	-3.57663400	-2.79312800	-0.82796000
H	-3.41157900	-3.81247900	-1.16044400
C	-4.84066500	-2.39094000	-0.41306200
H	-5.67060400	-3.08883200	-0.41920200
C	-5.02335100	-1.07991500	0.00993400
H	-6.00140700	-0.74560200	0.33994600
C	-3.96065300	-0.18606900	0.01328400
H	-4.13394300	0.82286300	0.35975000
C	2.98437400	-0.95790700	-0.15721100
C	3.73821500	-0.75107600	0.99204200
H	3.52769500	-1.31640900	1.89179100
C	4.77596100	0.17084100	0.96298600
H	5.36895500	0.33028600	1.85714200
C	5.06817700	0.89117500	-0.19512700
C	4.29616000	0.66195800	-1.33731700
H	4.51502900	1.20771400	-2.24917100
C	3.25722800	-0.25491200	-1.32760900

H	2.67407600	-0.43550300	-2.22249700
C	6.20732600	1.87169200	-0.23095100
H	7.07483200	1.42933600	-0.73116200
H	6.51406200	2.16309200	0.77532800
H	5.93395900	2.77299500	-0.78489500
C	-2.06725400	3.61449400	-1.65250200
H	-2.03807600	4.50556500	-1.02193700
H	-2.25552900	3.92028400	-2.68222800

2ae

#p opt freq ωb97xd/6-311g(d,p) scrf=(smd,solvent=chloroform)

0 1

S	-1.56228600	-2.13074700	0.20448000
O	-0.16838300	1.62385000	-1.06050200
O	-1.73784100	-3.07965900	-0.88006500
O	-1.40087500	-2.59023600	1.57259300
N	-0.21680400	-1.25330100	-0.15322800
C	0.25128500	-0.25579600	0.82461300
H	0.51713100	-0.73276500	1.76505900
H	-0.52795400	0.48388900	1.01610500
C	1.42421400	0.41377600	0.08932900
C	1.57513100	1.93812300	0.45071400
C	1.06627800	2.36979400	1.86062300
H	0.55726600	3.33584900	1.80355700
H	0.43250300	1.68230100	2.42040900
C	2.95392500	2.53185300	0.84648700
H	3.03830900	3.55426400	0.46548900
H	3.85765900	1.98750700	0.58208600
C	0.85436000	2.57768600	-0.78079800
H	0.43339900	3.56308000	-0.59184000
C	1.74855600	2.42416600	-1.99778000
H	2.39307300	3.18881000	-2.40895500
C	1.64878500	1.14515500	-2.35200700
H	2.18321100	0.60519500	-3.12031500
C	0.71593300	0.53421800	-1.33136600
C	-0.03631000	-0.75464800	-1.53378200
H	0.54082400	-1.47265500	-2.11762400

H	-0.98422100	-0.55595300	-2.04049100
C	2.67718500	-0.46238700	0.08281100
C	2.72486800	-1.67718900	0.77526300
H	1.86786700	-2.03036700	1.33166200
C	3.85535800	-2.48618200	0.75270100
H	3.84844100	-3.42087100	1.30262300
C	4.97724400	-2.10942000	0.02856300
H	5.85907400	-2.74015200	0.00963500
C	4.94890000	-0.91340200	-0.67656100
H	5.81181600	-0.59940900	-1.25393000
C	3.81830500	-0.10685400	-0.64860800
H	3.83325900	0.81385900	-1.21301400
C	-2.92798500	-0.99130200	0.16720600
C	-3.62490500	-0.79305500	-1.02022600
H	-3.39342000	-1.38847500	-1.89496600
C	-4.63676500	0.15421500	-1.05916500
H	-5.18902600	0.30380300	-1.98090800
C	-4.95968900	0.91218600	0.06808500
C	-4.24967800	0.68843200	1.24888300
H	-4.49877300	1.25631900	2.13902900
C	-3.23491400	-0.25547800	1.30702400
H	-2.70217000	-0.43458200	2.23305000
C	-6.03666800	1.95908500	0.00391900
H	-6.46912700	2.14556100	0.98897800
H	-6.83808900	1.66236200	-0.67618600
H	-5.62491900	2.90480100	-0.36358900
C	2.52751400	2.51234800	2.32492100
H	2.87807200	1.60824800	2.82874600
H	2.78122800	3.38084900	2.93500700

(7) References.

1) Liu, Z.-Y.; Zhang, M. and Wang, X.-C. Hydrosilylation-Promoted Furan Diels–Alder Cycloadditions with Stereoselectivity Controlled by the Silyl Group. *J. Am. Chem. Soc.* **2022**, *142*, 581-588. https://doi.org/10.1021/jacs.9b11909.
